# Temporal Events Detector for Pregnancy Care (TED-PC): A rule-based algorithm to infer gestational age and delivery date from electronic health records of pregnant women with and without COVID-19

**DOI:** 10.1371/journal.pone.0276923

**Published:** 2022-10-31

**Authors:** Tianchu Lyu, Chen Liang, Jihong Liu, Berry Campbell, Peiyin Hung, Yi-Wen Shih, Nadia Ghumman, Xiaoming Li, on behalf of the National COVID Cohort Collaborative Consortium

**Affiliations:** 1 Department of Health Services Policy and Management, Arnold School of Public Health, University of South Carolina, Columbia, South Carolina, United States of America; 2 Department of Epidemiology & Biostatistics, Arnold School of Public Health, University of South Carolina, Columbia, South Carolina, United States of America; 3 Department of Obstetrics and Gynecology, School of Medicine, University of South Carolina, Columbia, South Carolina, United States of America; 4 Department of Health Promotion Education and Behaviors, Arnold School of Public Health, University of South Carolina, Columbia, South Carolina, United States of America; Kyung Hee University School of Medicine, REPUBLIC OF KOREA

## Abstract

**Objective:**

Identifying the time of SARS-CoV-2 viral infection relative to specific gestational weeks is critical for delineating the role of viral infection timing in adverse pregnancy outcomes. However, this task is difficult when it comes to Electronic Health Records (EHR). In combating the COVID-19 pandemic for maternal health, we sought to develop and validate a clinical information extraction algorithm to detect the time of clinical events relative to gestational weeks.

**Materials and methods:**

We used EHR from the National COVID Cohort Collaborative (N3C), in which the EHR are normalized by the Observational Medical Outcomes Partnership (OMOP) Common Data Model (CDM). We performed EHR phenotyping, resulting in 270,897 pregnant women (June 1^st^, 2018 to May 31^st^, 2021). We developed a rule-based algorithm and performed a multi-level evaluation to test content validity and clinical validity, and extreme length of gestation (<150 or >300).

**Results:**

The algorithm identified 296,194 pregnancies (16,659 COVID-19, 174,744 without COVID-19) in 270,897 pregnant women. For inferring gestational age, 95% cases (n = 40) have moderate-high accuracy (Cohen’s Kappa = 0.62); 100% cases (n = 40) have moderate-high granularity of temporal information (Cohen’s Kappa = 1). For inferring delivery dates, the accuracy is 100% (Cohen’s Kappa = 1). The accuracy of gestational age detection for the extreme length of gestation is 93.3% (Cohen’s Kappa = 1). Mothers with COVID-19 showed higher prevalence in obesity or overweight (35.1% vs. 29.5%), diabetes (17.8% vs. 17.0%), chronic obstructive pulmonary disease (0.2% vs. 0.1%), respiratory distress syndrome or acute respiratory failure (1.8% vs. 0.2%).

**Discussion:**

We explored the characteristics of pregnant women by different gestational weeks of SARS-CoV-2 infection with our algorithm. TED-PC is the first to infer the exact gestational week linked with every clinical event from EHR and detect the timing of SARS-CoV-2 infection in pregnant women.

**Conclusion:**

The algorithm shows excellent clinical validity in inferring gestational age and delivery dates, which supports multiple EHR cohorts on N3C studying the impact of COVID-19 on pregnancy.

## Introduction

Recent findings suggested Coronavirus Disease 2019 (COVID-19) to be associated with an increased risk for adverse pregnancy outcomes and neonatal complications [1, 2]. However, there has been limited knowledge pertaining to the timing of SARS-CoV-2 infection during the pregnancy (e.g., infection in a specific trimester, gestational age [GA], or labor and delivery) and its association with pregnant women’s real-time clinical presentation [3]. Timing of viral infection is important because fetuses are more vulnerable to maternal complications and/or viral infection during certain gestational stages [4, 5]. Nevertheless, detecting the timing of viral infection poses substantial challenges to the quality of data and clinical information extraction methodology.

Pregnancy care consists of antenatal, labor and delivery, and postpartum care [6]. Because pregnancy care spans up to 21 months, it involves exceedingly rich clinical data. A complete episode of pregnancy care often involves multiple encounters with multiple health providers, clinical sites, and diverse clinical information systems, meaning that a vast number of clinical events are generated at an unprecedented granularity and data quality. Over decades, comprehensive clinical data for pregnancy care have not been widely available until recent advances in the use of normalized multi-system electronic health records (EHR), such as Observational Health Data Sciences and Informatics (OHDSI)’s Atlas [7], National Institutes of Health (NIH)’s All of Us, and NIH’s National COVID Cohort Collaborative (N3C) [8]. which have provided growing Real-World Evidence (RWE) to support pregnancy research during the COVID-19 pandemic [9–12].

One of the unique characteristics of EHR in pregnancy care is the complex temporal relations of clinical events. To better understand the impacts of SARS-CoV-2 infection or the COVID-19 pandemic on pregnancy health, it is important to know the length of pregnancy and the timing of pregnancy-related complications or events in relation to the time of pregnancy (i.e., GA). Both mothers and fetuses experience crucial physiological changes and clinical complications during pregnancy, which generates a substantial number of clinical events in the EHR. Accurate identification of temporal relations of clinical events across the entire episode of pregnancy care is a fundamental step for clinical decision-making as well as downstream EHR data mining. GA is also the prerequisite for tracing the timing of SARS-CoV-2 infection and COVID-19 vaccination for pregnant women. Although EHR data have unique advantages in preserving temporal information of these clinical events, clinical information extraction methods tailored for pregnancy care have been scarce [13].

Identifying GA from EHR is challenging because GA is a concept of temporal relativity. When it comes to clinical information extraction, this challenge is manifold. First, controlled vocabularies (e.g., ICD, SNOMED-CT, LOINC, RxNorm) along with the relational EHR database architecture are designed to preserve some temporal information of clinical events, yet the information suffers from a low level of granularity (e.g., LOINC 95656–5: Gestational age <30 weeks, LOINC 49085–4: First and Second trimester integrated maternal screen panel), unreliable data entries (e.g., laboratory test results sometimes have delayed time stamps), and incomplete data (e.g., laboratory test results sometime are missing due to many laboratory results are photocopies). Second, approximately 80% of the EHR data consist of unstructured data (i.e., clinical notes), with which a considerable amount of temporal information is in the form of free text that cannot be directly used for quantitative analysis [14]. Third, EHR data for pregnant women are distinct from most other medical specialties in that pregnancy care has scheduled routine visits that are involved with antenatal care, labor and delivery hospitalization, and postpartum care. Incomplete and/or inconsistent data are common because patients are often engaged with different providers, health care systems, and clinical visits with chief complaints not related to pregnancy but would generate data relevant to pregnancy [13]. For example, antenatal care and other medical care during the pregnancy could be at locations different from labor and delivery hospitals. Missing critical information from one clinical site would require researchers to infer such information using data from other sites or other visits. Health records of individual visits span the inpatient and various outpatient visits but may not contain explicit and consistent temporal information (e.g., last menstrual period [LMP] [15], estimated date of delivery [DOD], and GA).

Current biomedical informatics methods for extracting and inferring temporal relations of clinical events include rule-based methods, machine learning, natural language processing (NLP), ontology-based methods, and temporal reasoning [14, 16–23]. Most of these methods utilized unstructured clinical notes in combination with structured EHR data, which is comprehensive for generic temporal information extraction. However, methods focusing on temporal events among pregnant women’s EHR are limited. Among studies that extract or infer DOD and GA, LMP and imaging/lab test results are commonly used data; chart review is a commonly used method [17, 20, 22, 23]. Using LMP data requires the providers to accurately document LMP in the EHR, yet in the real world, many EHR datasets have a lot of missing values in LMP. The use of ultrasound test results or other laboratory test results requires the EHR to comprehensively document both laboratory orders and testing results, yet testing results are often missing in real-world EHR datasets. Additionally, using laboratory data alone for inferring GA may not be accurate due to the individual physiological variation among pregnant women. A recent study utilized comprehensive ICD codes of diagnoses and procedures to infer delivery dates [24]. While the study focused on full-term pregnancy with comprehensive medical records during the labor and delivery hospitalization, comprehensive methods remain needed for early-stage pregnancy (e.g., extreme preterm, very preterm) and those who have part of the pregnancy care data and conflicting data documented in EHR. Particularly, there are no published methods for extracting temporal relations of clinical events for pregnant women with COVID-19. This is a critical knowledge gap because temporal relations of clinical events are suggestive of the exact time of viral infection, acute phase of COVID-19, and vaccinations.

To identify temporal relations of clinical events imperative for pregnant women with COVID-19, we developed a rule-based clinical information extraction algorithm, namely Temporal Events Detector for Pregnancy Care (TED-PC), which infers GA and DOD using both structured EHR data and annotated clinical notes. The algorithm is designed to capture temporal information to be used for inferring GA and DOD, respectively, so that the complete temporal relations in a pregnancy episode can be replicated and the timing of SARS-CoV-2 infection (in weeks) can be detected. This design is anticipated to be effective for pregnant women with regular labor and delivery hospitalization, without complete hospitalization records, and those who have pre-term delivery, miscarriage, early-stage pregnancy and termination, and multiple births. This algorithm is designed for EHR that are normalized by Observational Medical Outcomes Partnership (OMOP) Common Data Model (CDM) [8, 25] and is implemented on the N3C enclave. EHR on N3C have 1) individual-level data linked among multiple health systems nationwide and 2) normalized procedures, laboratory tests/results, and annotated clinical notes, which enable the reasoning of GA and DOD for patients with missing and conflicting data. These unique characteristics of OMOP CDM-normalized EHR are critical for detecting the temporal information for pregnant women with COVID-19. The performance and clinical validity of TED-PC were tested systematically on the N3C platform. Presently, this algorithm is used as a critical clinical information extraction tool to identify comprehensive temporal relations of clinical events from multiple COVID-19 pregnant women cohorts on N3C [26–28].

## Materials and methods

### Data sources

We used the N3C database (level 3, containing dates of clinical events and zip codes), a multi-center clinical data repository that contains de-identified EHR data of individuals with COVID-19 blended with controls (i.e., non-COVID-19) [8]. N3C currently has EHR and medical claims data from more than 73 healthcare systems and institutes across 50 states. The EHR data are normalized using the OMOP CDM [8, 25]. In order to find the full clinical course of each pregnancy, the study cohort included women who met the following conditions: (1) have at least one childbirth between June 1^st^, 2018 and May 31^st^, 2021, (2) be aged between 15 to 49 years old at the DOD, and (3) have at least one GA-related record during the pregnancy.

Because the N3C database is normalized by OMOP CDM, we utilized the following resources for EHR phenotyping. The ATHENA vocabulary repository is used for retrieving OMOP CDM concept IDs and phenotyping patients with GA and childbirth-related records. The Algorithms section details the design and the procedures for using the ATHENA.

### Algorithms

**Fig 1:**
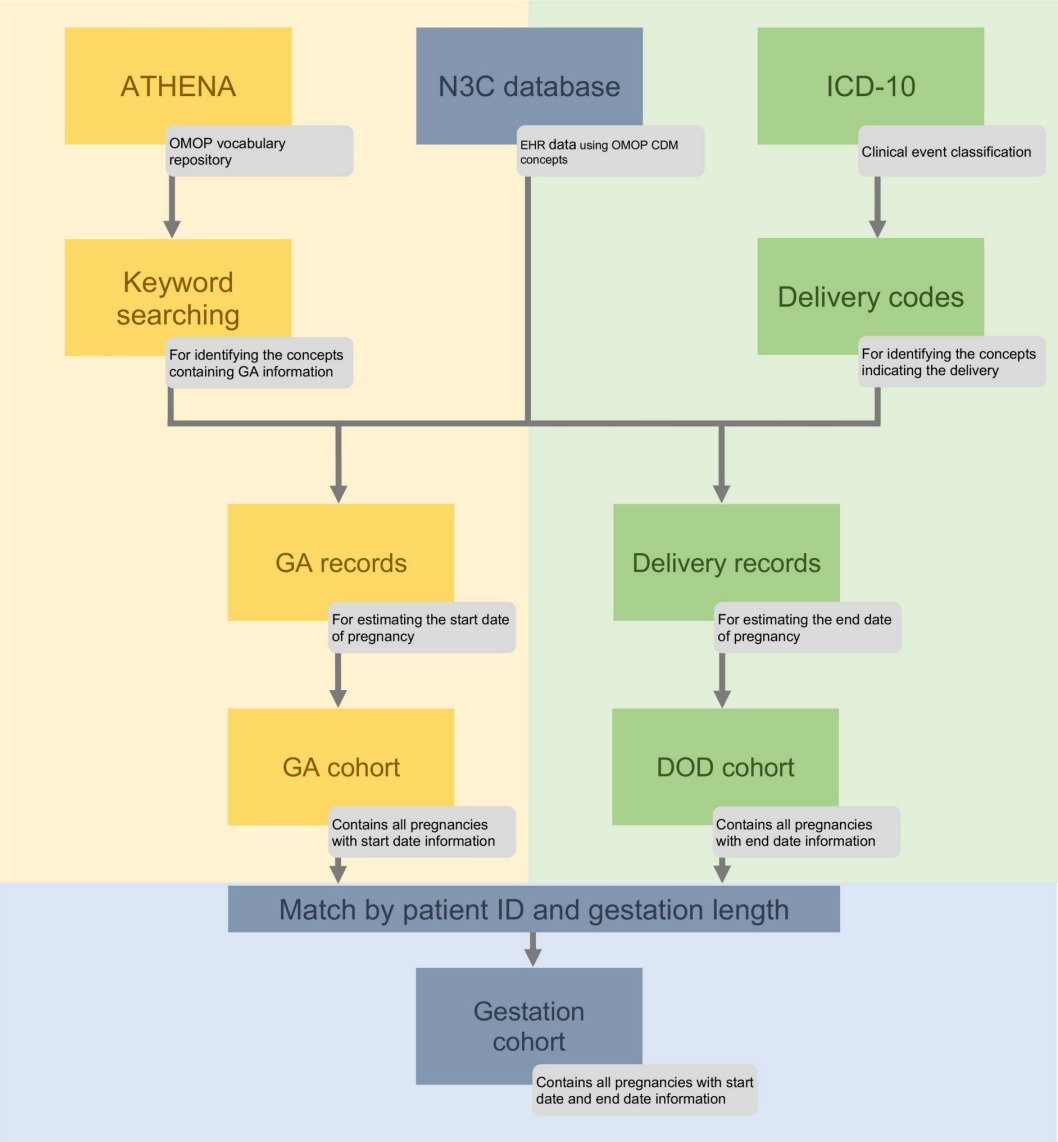
Pipeline of TED-PC for extracting information and building cohorts.

To retrieve the full spectrum of each pregnancy in the EHR, it is crucial to identify the start date (i.e., pregnancy start) and the end date (i.e., childbirth delivery) of the pregnancy. The start date can be estimated by the GA-related records that indicate the GA (e.g., in weeks, in a range of weeks, or a particular trimester) and the date of the record. The end date can be estimated by the identification of the DOD. Because some pregnant women’s EHR data only have either GA-related records or childbirth delivery records, we first estimated GA and DOD, respectively, which resulted in a cohort of pregnant women with estimated GA, denoted as the GA cohort, and a cohort of pregnant women with estimated DOD, denoted as the DOD cohort. Then we estimated the start date and end date of the pregnancy by consolidating the temporal information from both cohorts (Fig 1).

#### Gestational age cohort (GA cohort)

*Phenotyping*. The purpose of this step is to find OMOP CDM concepts that can be used to retrieve GA-related information from EHR data. For phenotyping the GA cohort, we used a keyword search strategy followed by a review of retrieved OMOP CDM concepts. Using ATHENA, a set of keywords were reviewed and determined: “trimester”, “gestation”, and “pregnan” (regular expression of “pregnancy” or “pregnant”). These keywords were used in conjunction with three filters in the ATHENA database: (1) “DOMAIN”, which included “Condition”, “Observation”, “Procedure”, “Measurement”, etc.; (2) “CONCEPT”, which included “Standard” and “Non-standard”; (3) “VALIDITY”, which included “Valid” and “Invalid”. The pseudo-query is:

(“trimester” OR “gestation” OR “pregnan”) AND (((DOMAIN = “Condition”) OR (DOMAIN = “Observation”) OR (DOMAIN = “Procedure”) OR (DOMAIN = “Measurement”)) AND (CONCEPT = “Standard”) AND (VALIDITY = “Valid”))

In the review of the returned OMOP CDM concepts, we applied the following three criteria to narrow down the scope step by step to our target concepts: (a) “Whether a record indicates a pregnant patient”; (b) “If yes, whether the record contains GA information of the patient”; (c) “If yes, what is the value of the GA?”. Finally, we identified 138 OMOP CDM concepts (See S1 Table). The researcher (TL) who performed the phenotyping was not involved in the phenotyping evaluation.

**Fig 2:**
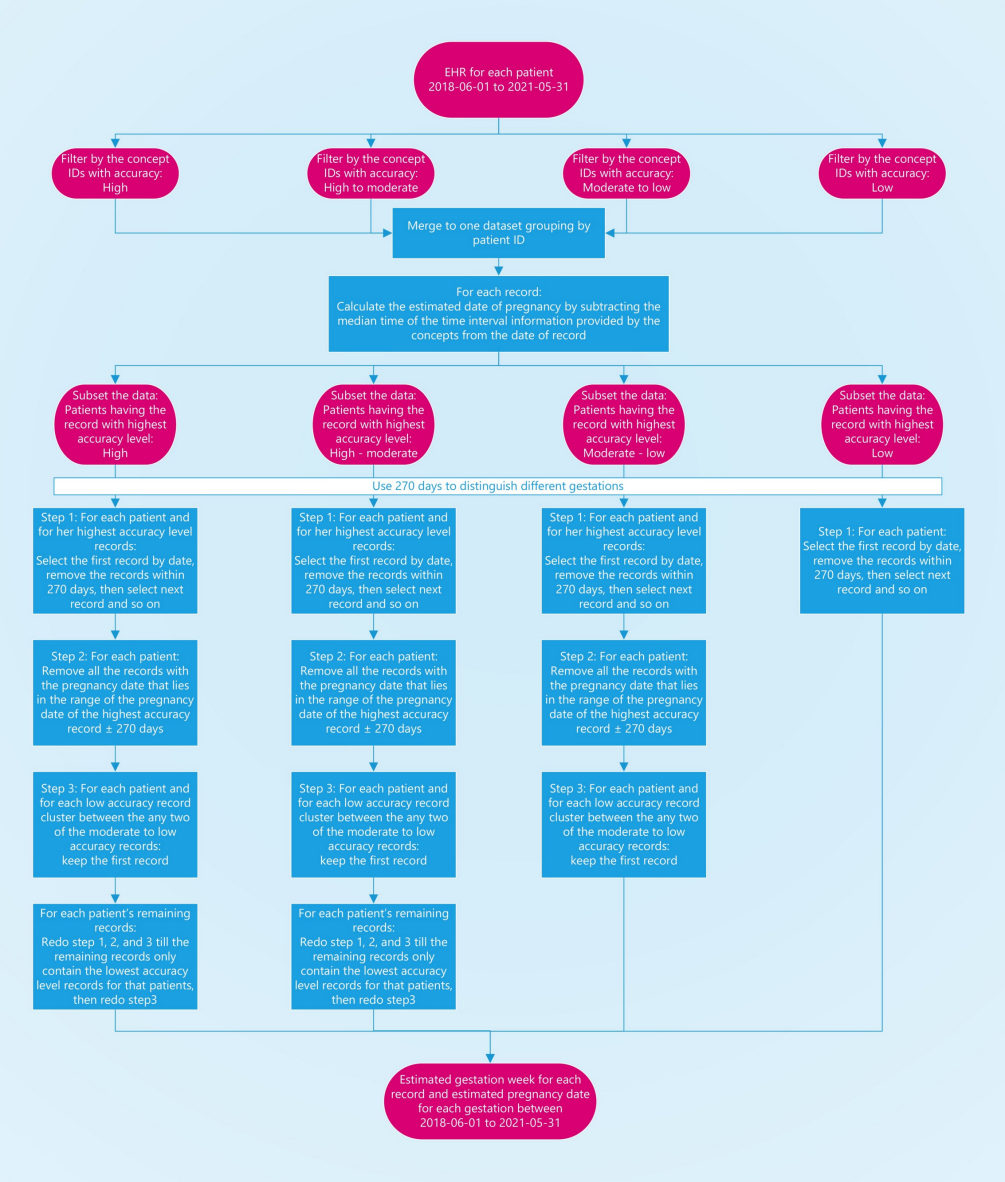
Flowchart for estimating gestational age and pregnancy dates.

*Rule-based algorithm*. We developed a rule-based algorithm to infer GA from EHR data (Fig 2). A critical feature of the algorithm is that we divided all extracted OMOP CDM concepts into four accuracy levels based on their clinical meanings and granularity of the date: high, moderate-high, moderate-low, and low (Table 1) and prioritized the retrieval of GA-related information based on accuracy levels. Table 2 shows the pseudocode for the algorithm.

**Table 1:** Concepts categorization by accuracy level.

Accuracy level	Time interval	Definition	Example concept IDs
High	1 week	The concept name specifies the value of GA in weeks (e.g., Gestation period, 15 weeks).	4337360: Gestation period, 1 week
			4097608: Gestation period, 18 weeks
			444098: Gestation period, 40 weeks
Moderate—high	2–5 weeks	The concept name does not specify the value of GA in weeks but specifies the range of GA in weeks which is larger than 1 week and smaller than 6 weeks (e.g., Gestation 9–13 weeks)	4181468: Gestation 9–13 weeks
			44791171: 9–13 weeks gestational age
			45757118: Spontaneous onset of labor between 37 and 39 weeks gestation with planned cesarean section
Moderate—low	6–10 weeks	The concept name does not specify the value of GA in weeks but specifies the range of GA in weeks which is larger than 5 weeks and smaller than 11 weeks (e.g., Gestation 14–20 weeks)	4180111: Third trimester pregnancy less than 36 weeks
			4178165: Gestation 14–20 weeks
			44791170: 14–20 weeks gestational age
Low	11–13 weeks	The concept name does not specify the value of GA in weeks but specifies the trimester (e.g., first trimester)	3657563: First trimester bleeding
			4239938: First trimester pregnancy
			4112238: Third trimester

GA: gestational age.

**Table 2:** The pseudocode for the algorithm: Estimating the gestational age.

Algorithm: GA estimation for each gestation	
1:	**procedure** GA ESTIMATION
2:	**Input:** GA-related clinical events, the date of GA-related clinical events
3:	**Output:** Estimated GA for each gestation
4:	pregnancy date <- calculated by the date of GA-related clinical events
5:	**if** the time range indicated by the GA-related clinical events = = 1 week **then**
6:	accuracy <- 1
7:	**else if** 2 weeks < = the time range indicated by the GA-related clinical events < = 5 weeks **then**
8:	accuracy <- 2
9:	**else if** 6 weeks < = the time range indicated by the GA-related clinical events < = 10 weeks **then**
10:	accuracy <- 3
11:	**else if** 11 weeks < = the time range indicated by the GA-related clinical events < = 13 weeks **then**
12:	accuracy <- 4
13:	**endif**
14:	**for** each patient
15:	Sort the GA-related clinical events by event date chronologically
16:	**repeat**
17:	Select the first pregnancy date with the highest accuracy*
18:	**for** the GA-related clinical events in (±270 days of the selected pregnancy date)
19:	Remove
20:	**endfor**
21:	Estimated GA <- the first GA-related clinical event with the highest accuracy*
22:	**until** the last GA-related clinical event
23:	**repeat**
24:	**if** remaining GA-related clinical events exist **then**
25:	**repeat**
26:	Select the first pregnancy date with the highest accuracy*
27:	**for** the GA-related clinical events in (±270 days of the selected pregnancy date)
28:	Remove
29:	**endfor**
30:	Estimated GA <- the first GA-related clinical event with the highest accuracy*
31:	**until** the GA-related clinical event
32:	**else** stop
33:	**endif**
34:	**until** the GA-related clinical event
35:	**endfor**
36:	**endprocedure**

GA: gestational age.

#### Childbirth delivery cohort (DOD cohort)

*Phenotyping*. For phenotyping the DOD cohort, we started with a list of CDC-recommended ICD, DRG, and CPT codes used for childbirth delivery and followed by exploring the relevant OMOP CDM concepts using the semantic relationships of concepts on ATHENA. First, we used a set of ICD-10, DRG, and CPT codes suggestive of childbirth delivery (see S2 Table) [29]. These codes were used to retrieve corresponding OMOP CDM concepts in ATHENA in which the resulting CDM concepts were then used to identify the childbirth delivery records in the EHR. Second, since these codes may not comprehensively capture all the OMOP CDM concepts indicating childbirth delivery, we explored the semantic relationships of the OMOP CDM concepts retrieved by these codes and supplemented them with the newly identified concepts [30]. The final concept set contained 105 OMOP CDM standard concepts (See S3 Table). Researchers (TL and YS) who performed the phenotyping and were not involved in the phenotyping evaluation.

**Fig 3:**
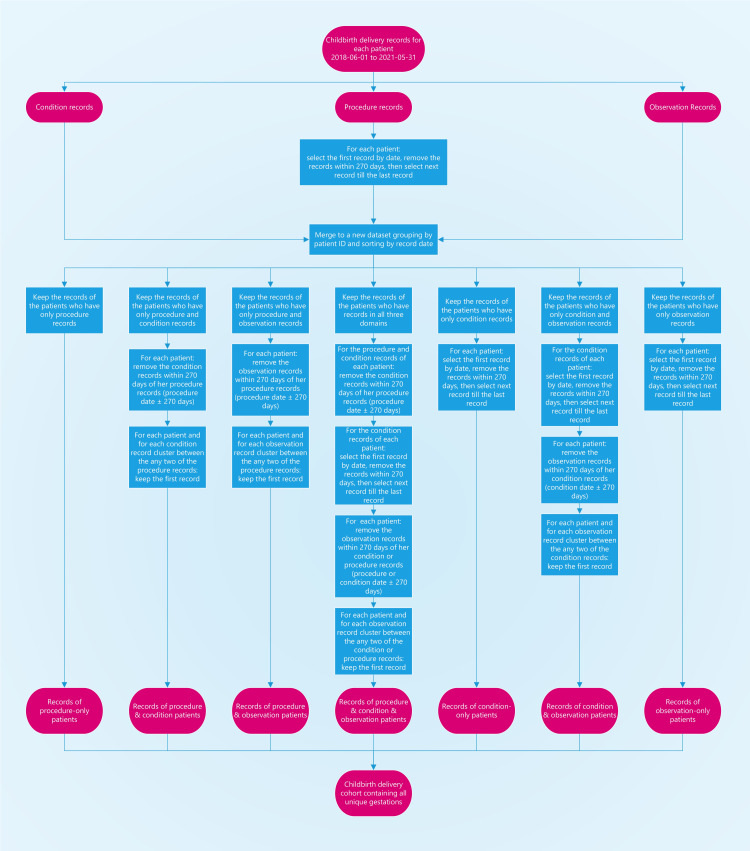
Flowchart for estimating dates of delivery.

*Rule-based algorithm*. Upon manual chart review of the EHR data, we found the OMOP CDM concepts with the domain of procedure have the highest accuracy with respect to determining the DOD, followed by domains of condition and then observation. Thus, we developed a rule-based algorithm to approximate the true DOD by prioritizing the OMOP CDM ‘procedure’ domain over the ‘condition’ domain over the ‘observation’ domain (Fig 3). Table 3 shows the pseudocode for the algorithm.

**Table 3:** The pseudocode for the algorithm: Estimating the date of delivery.

Algorithm: DOD estimation for each gestation	
1:	**procedure** DOD ESTIMATION
2:	**Input:** childbirth delivery clinical events, the domain and the date of DOD-related clinical events
3:	**Output:** Estimated DOD for each gestation
4:	Event DOD <- the date of childbirth delivery clinical events
5:	**if** domain = = “procedure” **then**
6:	accuracy <- 1
7:	**else if** domain = = “condition” **then**
8:	accuracy <- 2
9:	**else if** domain = = “observation” **then**
10:	accuracy <- 3
11:	**endif**
12:	**for** each patient
13:	Sort the childbirth delivery clinical events by event DOD in reversed chronological order
14:	**repeat**
15:	Select the first event DOD with the highest accuracy*
16:	**for** the childbirth delivery clinical event in (±270 days of the selected event DOD)
17:	Remove
18:	**endfor**
19:	Estimated DOD <- the first event DOD with the highest accuracy*
20:	**until** the last childbirth delivery clinical event
21:	**repeat**
22:	**if** remaining childbirth delivery clinical events exist **then**
23:	**repeat**
24:	Select the first event DOD with the highest accuracy*
25:	**for** the childbirth delivery clinical events in (±270 days of the selected event DOD)
26:	Remove
27:	**endfor**
28:	Estimated DOD <- the first event DOD with the highest accuracy*
29:	**until** the last childbirth delivery clinical event
30:	**else** stop
31:	**endif**
32:	**until** the last childbirth delivery clinical event
33:	**endfor**
34:	**endprocedure**

DOD: date of delivery.

All data manipulation, phenotyping, and algorithms were implemented using SQL, R, Python, and PySpark on the N3C platform. Source programming codes are available at N3C, project “[RP-2B9622] Assessing and predicting the clinical outcomes of pregnant women with COVID-19 using machine learning approach.”

### Evaluation

We performed a multi-level evaluation to test the validity of the algorithm as well as inter-rater reliability. To test the content validity of the OMOP CDM concepts resulting from the phenotyping, two researchers (CL and NG) independently reviewed the concept IDs and their semantic meanings and properties on the ATHENA and dichotomously rated the relevance of all concept IDs. Inter-rater reliability was measured by Cohen’s Kappa. Disagreements were discussed and resolved together with a senior OB/GYN physician (BC).

To test the clinical validity of the algorithm for inferring GA, we randomly selected 30 patients from the final cohort, which resulted in 40 distinct pregnancies, including multiple gestations. Their comprehensive medical records on GA, excluding laboratory data, were extracted from the EHR. We calculated the start date of pregnancy by subtracting the GA in weeks from the event date for each record. Two clinical experts (CL and NG) independently reviewed the retrieved records and rated them based on two metrics: accuracy (high/moderate/low) and granularity (high/moderate/low). Accuracy is concerned with the level that the selected OMOP CDM concepts can accurately indicate the GA. For example, GA-related records are typically documented during antenatal care visits. Multiple GA-related records, even when documented at different dates, can suggest a consistent GA. The algorithm-selected concept is most accurate if it is among these records. When there are other GA-related records suggesting a GA different from the algorithm-selected record, the accuracy would not be high. Granularity refers to the extent that the algorithm-selected concept can indicate a specific gestational week. For example, the “gestation period, 38 weeks” has a high granularity level whereas “third-trimester pregnancy” has a low granularity level.

To test the clinical validity of the algorithm for inferring DOD, we randomly selected 30 gestations from the final cohort. Their records consisting of procedures, conditions, observations, and measurements were extracted from the EHR within ± 14 days of estimated DOD. Two clinical experts (CL and NG) independently reviewed the charts and labeled whether the DOD was correctly inferred by the algorithm.

Despite the average gestation being around 280 days, this estimation varies among individuals. To represent rare cases such as preterm birth, post-term birth, and early-stage termination, we also performed extreme value analysis, in which two clinical experts (TL and CL) performed chart reviews for 30 randomly selected samples with <150 or >300 days of gestation.

### Characteristics of pregnant women with and without COVID-19

Using TED-PC, we performed descriptive analyses to explore maternal demographics and underlying conditions (See S4 Table for OMOP CDM concepts) for pregnant women with (cases) and without COVID-19 (controls) which are characterized by temporal information of the gestational weeks when SARS-CoV-2 infection was identified.

## Results

### Identified OMOP CDM concepts

We identified 2,773 OMOP CDM concepts from the ATHENA vocabulary repository, of which 2,370 indicated pregnancy. Among the concepts relating to pregnancy, 336 have GA-related information. We excluded 189 concepts that either indicated the inaccurate time range broader than one trimester (13 weeks) (e.g., concept ID 21493940: US for pregnancy in the second or third trimester) or did not have corresponding records in the N3C database (e.g., concept ID 3025286: Gestational age estimated from foot length on US by Mercer 1987 method). Totally, 138 concepts contained useful gestational week information with a time range from one week to one trimester. Within the selected 138 concepts, 42 (30.4%) were in high accuracy, 9 (6.5%) were in moderate-high accuracy, 5 (3.6%) were in moderate-low accuracy, and 82 (59.4%) were in low accuracy.

### Algorithm performance

To evaluate phenotyping results, the content validity of the selected concepts was assessed and rated blindfolded by two independent reviewers (CL and NG) who did not participate in the phenotyping. Both reviewers rated all concepts as “valid” (100% agreement).

We evaluated the performance of the GA algorithm in two dimensions: accuracy and granularity. Among the 30 randomly selected pregnant women, eight of them had two gestations and one of them had three gestations during the study time frame. The mean gestation length was 270.15 days with a maximum of 299 days and a minimum of 159 days. Among the 40 pregnancies, one reviewer rated 34 (85.0%) samples as high accuracy, 4 (10.0%) samples as moderate accuracy, and 2 (5.0%) sample as low accuracy. The other reviewer rated 35 (87.5%) samples as high accuracy, 3 (7.5%) samples as moderate accuracy, and 2 (5.0%) samples as low accuracy. The Cohen’s Kappa with linear weighting is 0.62, CI = [0.35, 0.90]. For granularity, both reviewers rated the 39 samples as high granularity and one sample as low granularity (100% agreement, unweighted Cohen’s Kappa = 1). See Table 4 for the confusion matrix.

**Table 4:** Confusion matrix of the accuracy rating for the performance of the GA algorithm.

		Reviewer2			
		High	Moderate	Low	Total
Reviewer1	High	33	1	0	34
	Moderate	2	1	1	4
	Low	0	1	1	2
	Total	35	3	2	40

For the DOD algorithm, a total of 30 patients’ EHR were reviewed independently. Both reviewers rated the 30 samples to be accurate (100% agreement, unweighted Cohen’s Kappa = 1)

### Extreme value analysis

We randomly selected 30 gestations with a gestation length either smaller than 150 days or greater than 300 days and extracted their EHR. After chart review, 28 of the 30 gestations were extracted with correct GA information, with an accuracy of 93.3%. For the two error cases, the first one was due to the contradiction between the GA records on different dates. For example, there was a record with the concept name “Gestation period, 38 weeks” on date 1, but other records with the same concept on date 2. The second error case was due to the contradiction between the GA records on the same date. For example, on the same date, one record was with the concept name “Gestation period, 36 weeks” and another record was with the concept name “Gestation period, 39 weeks”. Among the correctly inferred cases, a certain level of inaccuracy existed. Five gestations only had low accuracy level GA records. Among the extreme case, one of them had only one GA record. Inter-rater reliability is 100% (unweighted Cohen’s Kappa = 1).

### Characteristics of pregnant women with and without COVID-19

Between June 1^st^, 2018 and May 31^st^, 2021, a total of 296,194 gestations in 270,897 pregnant women were identified from the N3C database. The mean and median ages are 30.31 and 31, respectively. There were 245,892 women who had one pregnancy during the study time, 24,713 and 292 had two and three pregnancies, respectively. The mean gestational length was 274.14 days. The median was 278 days, with a minimum of 140 days and a maximum of 308 days. N3C data retrieval was completed on 02/12/2022.

**Fig 4:**
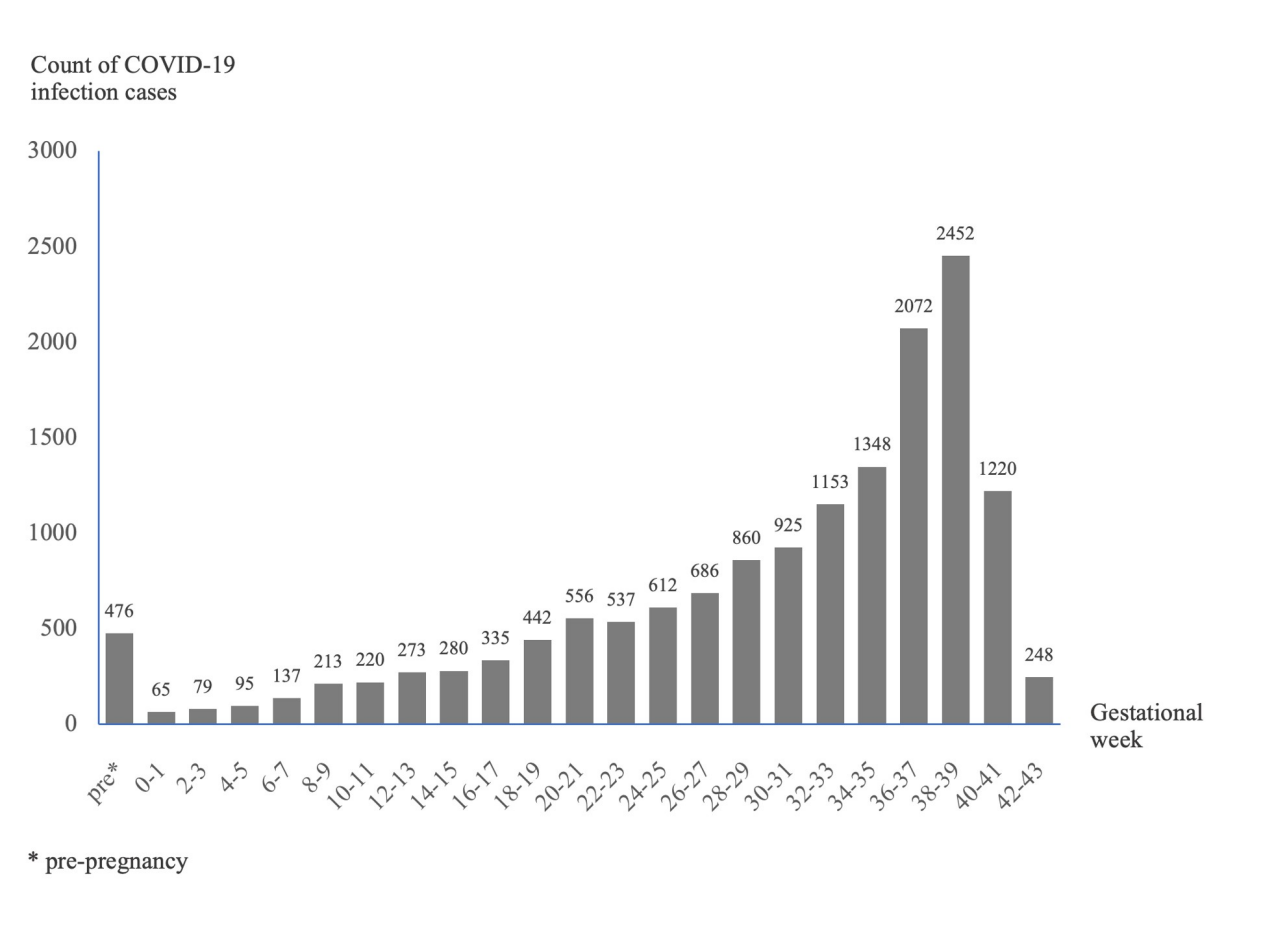
The distribution of the SARS-CoV-2 infection cases by gestational week.

Using TED-PC, we identified the timing of SARS-CoV-2 infections in gestational weeks. Fig 4 shows the frequency of infections across gestational weeks. More than half of the infections happened during late pregnancy (between 32 and 41 weeks), which might be related to the increased antenatal visits in late pregnancy. See S5 Table for the selected demographics and underlying conditions of the cohort captured by TED-PC, stratified by trimesters. There were 104,791 and 191,403 gestations before and during the COVID-19 pandemic, respectively, among which there are 16,659 gestations with COVID-19 and 174,744 without COVID-19 peri-pandemic. Age group 30–34 shared the largest proportion across the age groups, followed by the age groups 25–29, 40–44, and 20–24. White people made up the largest percentage of nearly 50% of the total population, followed by Black and Hispanic/Latino races. For mothers who had ever been infected by SARS-CoV-2 before the DOD (before or during the pregnancy), age groups 30–34, 20–24, and 25–29 had the largest percentage. Besides, the percentage of White was lower (39.7%) compared with that of pre-pandemic (57.1%). Hispanic/Latino made up the largest proportion across the races in those SARS-CoV-2 infected mothers (31.5%). Compared with the pregnant women without COVID-19, pregnant women with COVID-19 had a higher prevalence in obesity or overweight (35.1% vs. 29.5%), diabetes (17.8% vs. 17.0%), chronic obstructive pulmonary disease (COPD) (0.2% vs. 0.1%), respiratory distress syndrome (ARDS) or acute respiratory failure (ARF) (1.8% vs. 0.2%), myocardial infarction (0.2% vs. 0.1%), and HIV/AIDS (0.6% vs. 0.4%). The characteristics of the proportions shared similar trends when stratified by different trimesters.

## Discussion

Using N3C data, we created the first EHR-based cohort of SARS-CoV-2-infected pregnant women in the US with complete temporal information of clinical events spanning the gestation length, which supports urgently needed COVID-19 research for pregnant women. Our algorithm is among the first that detects exact gestational week of viral infection, early-state pregnancy, preterm birth, early termination, post-term birth, and other adverse clinical events. Because viral infection at different stages of pregnancy is associated with different risks of fetus development and maternal status, TED-PC is generalizable to viral infection aside from COVID-19 as well as adverse events that would affect pregnancy. This algorithm shows the promise to underpin EHR deep phenotyping of pregnancy care as well as machine learning methods, in which both require precise temporal information of clinical events. As a rapid development of the clinical information extraction tool for combating COVID-19, our algorithm is currently supporting several EHR-based cohort studies on the N3C to examine the impact of COVID-19 on pregnant women’s real-time clinical inflammatory progression and pregnancy complications.

The accuracy of TED-PC is warranted by a few logic layers. First, compared with previous studies that focused on claims data or required labor efforts, our study took advantage of the OMOP CDM normalized EHR data to categorize normalized concepts into different priority groups [17, 22–24, 31–34]. For example, the GA algorithm prioritized the concepts in the “Procedure” domain over “Condition” domain over “Observation” domain, which logically prevented the algorithm from selecting the records at a higher risk of semantic ambiguity and low granularity. Second, our algorithm categorized the GA-related concepts into different accuracy levels by indicating the time range. This step allows TED-PC to prioritize records with the most accurate information. Third, the use of the 270-day interval in our algorithm enabled us to distinguish different gestations of the same pregnant woman within the time frame. Fourth, the merging and matching process of the GA cohort and the DOD cohort excluded gestations with untrustworthy or missing values, which is common in EHR.

Detection of temporal information for pregnant women with COVID-19 is made available by two major features of OMOP CDM. First, OMOP CDM normalizes multi-system EHR data linked at an individual level. This unique feature enables our algorithm to impute a huge amount of missing temporal values and to resolve conflicts of temporal values among health records from different hospital systems. Second, OMOP CDM includes annotated clinical notes, procedures, and laboratory tests/results, which allows the algorithm to leverage multi-source contextual information for inferring temporal information at an adequate level of granularity.

A few limitations of this study warrant note. First, because several GA-related OMOP CDM concepts do not indicate specific gestational weeks (e.g., “Spontaneous onset of labor between 37 and 39 week gestation with planned cesarean section”), we inferred the gestational weeks using the median time point of the range for these concepts, which may impair the performance of the algorithm. This impact is mild on the concepts with high or moderate-high accuracy levels since the time range is small, but it could be severe in the concepts with the low accuracy level. Second, our EHR data may not be comprehensive. For example, some examinations or laboratory tests do not have time information, but they are often prescribed to pregnant women during a specific time frame of gestation. Our future direction will aim to improve the performance of TED-PC and test the external validity. From error analysis, data incompleteness and inconsistency remain the major sources of error. Well-designed EHR data imputation methods and a hybrid model of rule-based and machine learning algorithms hold promises for addressing these issues. Although our algorithm is implemented on N3C, it could be potentially repurposed for other OMOP CDM normalized EHR.

## Conclusion

We explored and compared the characteristics of pregnant women by different timing of SARS-CoV-2 infection with our newly developed technique: TED-PC, a rule-based algorithm to automatically infer comprehensive temporal information of clinical events from EHR during pregnancy care. The performance of TED-PC is satisfactory as collectively the accuracy and granularity of temporal information are beyond 90%. TED-PC has been implemented on N3C, supporting multiple national EHR cohorts for desperately needed research on the impact of COVID-19 on pregnancy. TED-PC is implemented on N3C data but remains generalizable for OMOP CDM normalized EHR.

## Supporting information

S1 Table OMOP CDM concepts for gestational age-related EHR records. (DOCX)

S2 Table ICD, CPT, and DRG codes suggestive of childbirth delivery dates. (DOCX)

S3 Table OMOP CDM concepts for delivery date-related EHR records. (DOCX)

S4 Table OMOP CDM concepts for underlying conditions. (DOCX)

S5 Table Selected demographics and underlying conditions for pregnant women with and without COVID-19 by gestations during pre- and peri-pandemic. (DOCX)
