# Supplementary material for: Temporal Events Detector for Pregnancy Care (TED-PC): A rule-based algorithm to infer gestational age and delivery date from electronic health records of pregnant women with and without COVID-19
Source: PLoS One. 2022 Oct 31;17(10):e0276923. doi: 10.1371/journal.pone.0276923 (PMC9621451; doi:10.1371/journal.pone.0276923)
Supplement: S1 Table — (DOCX) [file pone.0276923.s001.docx]

**Supporting information 1**

Table. OMOP CDM concepts for gestational age-related EHR.

| **Concept ID** | **Concept Name** | **Class** | **Domain** | **Vocabulary** |
| --- | --- | --- | --- | --- |
| 2793352 | Ultrasonography of Third Trimester, Multiple Gestation | ICD10PCS | Procedure | ICD10PCS |
| 2825091 | Imaging @ Fetus and Obstetrical @ Ultrasonography @ Third Trimester, Multiple Gestation | ICD10PCS Hierarchy | Procedure | ICD10PCS |
| 2871670 | Imaging @ Fetus and Obstetrical @ Ultrasonography @ Third Trimester, Multiple Gestation @ None | ICD10PCS Hierarchy | Procedure | ICD10PCS |
| 2898834 | Imaging @ Fetus and Obstetrical @ Ultrasonography @ Third Trimester, Multiple Gestation @ None @ None | ICD10PCS Hierarchy | Procedure | ICD10PCS |
| 4112238 | Third trimester | Qualifier Value | Observation | SNOMED |
| 4218813 | Third trimester pregnancy | Clinical Finding | Condition | SNOMED |
| 4142581 | Third trimester bleeding | Clinical Finding | Condition | SNOMED |
| 4167685 | Miscarriage in third trimester | Clinical Finding | Condition | SNOMED |
| 40487413 | Ultrasonography in third trimester | Procedure | Procedure | SNOMED |
| 4032055 | Threatened miscarriage in third trimester | Clinical Finding | Condition | SNOMED |
| 45757176 | Preterm labor in third trimester with preterm delivery in third trimester | Clinical Finding | Condition | SNOMED |
| 4029320 | Prenatal state of fetus, 3rd trimester | Clinical Finding | Condition | SNOMED |
| 35625971 | Vomiting during third trimester of pregnancy | Clinical Finding | Condition | SNOMED |
| 36713272 | Three dimensional obstetric ultrasonography in third trimester | Procedure | Procedure | SNOMED |
| 2793351 | Ultrasonography of Third Trimester, Single Fetus | ICD10PCS | Procedure | ICD10PCS |
| 3032525 | Fetal Narrative [Interpretation] Study observation.general 3rd trimester US | Clinical Observation | Measurement | LOINC |
| 3032291 | Fetal Narrative [Interpretation] Study observation.general 3rd trimester, multiple fetuses US | Clinical Observation | Measurement | LOINC |
| 2898832 | Imaging @ Fetus and Obstetrical @ Ultrasonography @ Third Trimester, Single Fetus | ICD10PCS Hierarchy | Procedure | ICD10PCS |
| 2898833 | Imaging @ Fetus and Obstetrical @ Ultrasonography @ Third Trimester, Single Fetus @ None | ICD10PCS Hierarchy | Procedure | ICD10PCS |
| 2807693 | Imaging @ Fetus and Obstetrical @ Ultrasonography @ Third Trimester, Single Fetus @ None @ None | ICD10PCS Hierarchy | Procedure | ICD10PCS |
| 2793350 | Ultrasonography of Second Trimester, Multiple Gestation | ICD10PCS | Procedure | ICD10PCS |
| 2832899 | Imaging @ Fetus and Obstetrical @ Ultrasonography @ Second Trimester, Multiple Gestation | ICD10PCS Hierarchy | Procedure | ICD10PCS |
| 2871669 | Imaging @ Fetus and Obstetrical @ Ultrasonography @ Second Trimester, Multiple Gestation @ None | ICD10PCS Hierarchy | Procedure | ICD10PCS |
| 2879607 | Imaging @ Fetus and Obstetrical @ Ultrasonography @ Second Trimester, Multiple Gestation @ None @ None | ICD10PCS Hierarchy | Procedure | ICD10PCS |
| 4113140 | Second trimester | Qualifier Value | Observation | SNOMED |
| 4327745 | Second trimester bleeding | Clinical Finding | Condition | SNOMED |
| 4244438 | Second trimester pregnancy | Clinical Finding | Condition | SNOMED |
| 40486918 | Ultrasonography in second trimester | Procedure | Procedure | SNOMED |
| 4224646 | Miscarriage in second trimester | Clinical Finding | Condition | SNOMED |
| 4294259 | Threatened miscarriage in second trimester | Clinical Finding | Condition | SNOMED |
| 45757175 | Preterm labor in second trimester with preterm delivery in second trimester | Clinical Finding | Condition | SNOMED |
| 4240362 | Prenatal state of fetus, 2nd trimester | Clinical Finding | Condition | SNOMED |
| 45763591 | Induced termination of pregnancy in second trimester | Clinical Finding | Condition | SNOMED |
| 40483600 | Measurement of alpha fetoprotein in second trimester | Procedure | Measurement | SNOMED |
| 4202200 | Surgical treatment of missed miscarriage of second trimester | Procedure | Procedure | SNOMED |
| 4290406 | Surgical treatment of missed miscarriage of third trimester | Procedure | Procedure | SNOMED |
| 2110327 | Treatment of missed abortion, completed surgically; second trimester | CPT4 | Procedure | CPT4 |
| 36305399 | Cigarettes smoked per day by Mother--2nd trimester | Survey | Observation | LOINC |
| 2793349 | Ultrasonography of Second Trimester, Single Fetus | ICD10PCS | Procedure | ICD10PCS |
| 3037974 | Fetal Narrative [Interpretation] Study observation.general 2nd trimester US | Clinical Observation | Measurement | LOINC |
| 3031374 | Fetal Narrative [Interpretation] Study observation.general 2nd trimester, multiple fetuses US | Clinical Observation | Measurement | LOINC |
| 2884808 | Imaging @ Fetus and Obstetrical @ Ultrasonography @ Second Trimester, Single Fetus | ICD10PCS Hierarchy | Procedure | ICD10PCS |
| 2840219 | Imaging @ Fetus and Obstetrical @ Ultrasonography @ Second Trimester, Single Fetus @ None | ICD10PCS Hierarchy | Procedure | ICD10PCS |
| 2852789 | Imaging @ Fetus and Obstetrical @ Ultrasonography @ Second Trimester, Single Fetus @ None @ None | ICD10PCS Hierarchy | Procedure | ICD10PCS |
| 2211747 | Ultrasound, pregnant uterus, real time with image documentation, fetal and maternal evaluation, first trimester (< 14 weeks 0 days), transabdominal approach; single or first gestation | CPT4 | Procedure | CPT4 |
| 2211748 | Ultrasound, pregnant uterus, real time with image documentation, fetal and maternal evaluation, first trimester (< 14 weeks 0 days), transabdominal approach; each additional gestation (List separately in addition to code for primary procedure) | CPT4 | Procedure | CPT4 |
| 2211753 | Ultrasound, pregnant uterus, real time with image documentation, first trimester fetal nuchal translucency measurement, transabdominal or transvaginal approach; single or first gestation | CPT4 | Procedure | CPT4 |
| 2211754 | Ultrasound, pregnant uterus, real time with image documentation, first trimester fetal nuchal translucency measurement, transabdominal or transvaginal approach; each additional gestation (List separately in addition to code for primary procedure) | CPT4 | Procedure | CPT4 |
| 2793348 | Ultrasonography of First Trimester, Multiple Gestation | ICD10PCS | Procedure | ICD10PCS |
| 21493908 | US for multiple gestation pregnancy in first trimester | Clinical Observation | Measurement | LOINC |
| 2879606 | Imaging @ Fetus and Obstetrical @ Ultrasonography @ First Trimester, Multiple Gestation | ICD10PCS Hierarchy | Procedure | ICD10PCS |
| 2898831 | Imaging @ Fetus and Obstetrical @ Ultrasonography @ First Trimester, Multiple Gestation @ None | ICD10PCS Hierarchy | Procedure | ICD10PCS |
| 21493909 | US transabdominal and transvaginal for multiple gestation pregnancy in first trimester | Clinical Observation | Measurement | LOINC |
| 2840218 | Imaging @ Fetus and Obstetrical @ Ultrasonography @ First Trimester, Multiple Gestation @ None @ None | ICD10PCS Hierarchy | Procedure | ICD10PCS |
| 21494044 | US for pregnancy in first trimester | Clinical Observation | Measurement | LOINC |
| 21494042 | US transabdominal and transvaginal for pregnancy in first trimester | Clinical Observation | Measurement | LOINC |
| 36203523 | Traveled outside the U.S. during first trimester of pregnancy of Mother | Clinical Observation | Measurement | LOINC |
| 3050129 | First trimester maternal screen panel - Serum or Plasma | Lab Test | Measurement | LOINC |
| 3030256 | First trimester maternal screen with nuchal translucency [Interpretation] | Clinical Observation | Observation | LOINC |
| 3031648 | First trimester maternal screen with nuchal translucency [Interpretation] Narrative | Clinical Observation | Observation | LOINC |
| 3050402 | First trimester maternal screen with nuchal translucency panel | Lab Test | Measurement | LOINC |
| 4113139 | First trimester | Qualifier Value | Observation | SNOMED |
| 3657563 | First trimester bleeding | Clinical Finding | Condition | SNOMED |
| 4239938 | First trimester pregnancy | Clinical Finding | Condition | SNOMED |
| 4078393 | Miscarriage in first trimester | Clinical Finding | Condition | SNOMED |
| 40488298 | Ultrasonography in first trimester | Procedure | Procedure | SNOMED |
| 4252252 | Threatened miscarriage in first trimester | Clinical Finding | Condition | SNOMED |
| 4034340 | Prenatal state of fetus, 1st trimester | Clinical Finding | Condition | SNOMED |
| 45763590 | Induced termination of pregnancy in first trimester | Clinical Finding | Condition | SNOMED |
| 42538969 | First trimester Down screening blood test abnormal | Clinical Finding | Condition | SNOMED |
| 40480885 | Education about folic acid in first trimester | Procedure | Procedure | SNOMED |
| 43020954 | Termination of pregnancy after first trimester | Procedure | Procedure | SNOMED |
| 4087135 | Surgical treatment of missed miscarriage of first trimester | Procedure | Procedure | SNOMED |
| 2110326 | Treatment of missed abortion, completed surgically; first trimester | CPT4 | Procedure | CPT4 |
| 36304648 | Cigarettes smoked per day by Mother--1st trimester | Survey | Observation | LOINC |
| 2793347 | Ultrasonography of First Trimester, Single Fetus | ICD10PCS | Procedure | ICD10PCS |
| 3034062 | Fetal Narrative [Interpretation] Study observation.general 1st trimester US | Clinical Observation | Measurement | LOINC |
| 3037993 | Fetal Narrative [Interpretation] Study observation.general transvaginal 1st trimester US | Clinical Observation | Measurement | LOINC |
| 3034647 | Fetal Narrative [Interpretation] Study observation.general 1st trimester, multiple fetuses US | Clinical Observation | Measurement | LOINC |
| 2892608 | Imaging @ Fetus and Obstetrical @ Ultrasonography @ First Trimester, Single Fetus | ICD10PCS Hierarchy | Procedure | ICD10PCS |
| 2892609 | Imaging @ Fetus and Obstetrical @ Ultrasonography @ First Trimester, Single Fetus @ None | ICD10PCS Hierarchy | Procedure | ICD10PCS |
| 2807692 | Imaging @ Fetus and Obstetrical @ Ultrasonography @ First Trimester, Single Fetus @ None @ None | ICD10PCS Hierarchy | Procedure | ICD10PCS |
| 4181468 | Gestation 9- 13 weeks | Clinical Finding | Condition | SNOMED |
| 44791171 | 9 - 13 weeks gestational age | Clinical Finding | Condition | SNOMED |
| 4245908 | Gestation period, 9 weeks | Clinical Finding | Condition | SNOMED |
| 4270513 | Gestation period, 7 weeks | Clinical Finding | Condition | SNOMED |
| 4313026 | Gestation period, 6 weeks | Clinical Finding | Condition | SNOMED |
| 4290009 | Gestation period, 5 weeks | Clinical Finding | Condition | SNOMED |
| 444067 | Gestation period, 42 weeks | Clinical Finding | Condition | SNOMED |
| 442769 | Gestation period, 41 weeks | Clinical Finding | Condition | SNOMED |
| 45773507 | Post-term pregnancy of 40 to 42 weeks | Clinical Finding | Condition | SNOMED |
| 444098 | Gestation period, 40 weeks | Clinical Finding | Condition | SNOMED |
| 435655 | Gestation period, 39 weeks | Clinical Finding | Condition | SNOMED |
| 443871 | Gestation period, 38 weeks | Clinical Finding | Condition | SNOMED |
| 45757118 | Spontaneous onset of labor between 37 and 39 weeks gestation with planned cesarean section | Clinical Finding | Condition | SNOMED |
| 442355 | Gestation period, 37 weeks | Clinical Finding | Condition | SNOMED |
| 40757033 | Group B Streptococcus (GBS) screening documented as performed during week 35-37 gestation (Pre-Cr) | CPT4 | Observation | CPT4 |
| 444267 | Gestation period, 35 weeks | Clinical Finding | Condition | SNOMED |
| 443874 | Gestation period, 34 weeks | Clinical Finding | Condition | SNOMED |
| 441678 | Gestation period, 33 weeks | Clinical Finding | Condition | SNOMED |
| 433864 | Gestation period, 31 weeks | Clinical Finding | Condition | SNOMED |
| 434484 | Gestation period, 30 weeks | Clinical Finding | Condition | SNOMED |
| 4326232 | Gestation period, 3 weeks | Clinical Finding | Condition | SNOMED |
| 444417 | Gestation period, 29 weeks | Clinical Finding | Condition | SNOMED |
| 44817054 | Mother's Non-treponemal or treponemal test was performed at 28-32 weeks gestation [CDC.CS] | Survey | Observation | LOINC |
| 4180111 | Third trimester pregnancy less than 36 weeks | Clinical Finding | Condition | SNOMED |
| 432430 | Gestation period, 27 weeks | Clinical Finding | Condition | SNOMED |
| 444023 | Gestation period, 26 weeks | Clinical Finding | Condition | SNOMED |
| 435640 | Gestation period, 25 weeks | Clinical Finding | Condition | SNOMED |
| 4336226 | Gestation period, 23 weeks | Clinical Finding | Condition | SNOMED |
| 4274955 | Gestation period, 22 weeks | Clinical Finding | Condition | SNOMED |
| 4185780 | Gestation period, 21 weeks | Clinical Finding | Condition | SNOMED |
| 4220085 | Gestation period, 2 weeks | Clinical Finding | Condition | SNOMED |
| 4181751 | Gestation period, 19 weeks | Clinical Finding | Condition | SNOMED |
| 44790206 | Mid trimester scan | Procedure | Procedure | SNOMED |
| 4097608 | Gestation period, 18 weeks | Clinical Finding | Condition | SNOMED |
| 4277749 | Gestation period, 17 weeks | Clinical Finding | Condition | SNOMED |
| 4283690 | Gestation period, 15 weeks | Clinical Finding | Condition | SNOMED |
| 4178165 | Gestation 14 - 20 weeks | Clinical Finding | Condition | SNOMED |
| 44791170 | 14 - 20 weeks gestational age | Clinical Finding | Condition | SNOMED |
| 4248725 | Gestation period, 14 weeks | Clinical Finding | Condition | SNOMED |
| 4266517 | Gestation period, 13 weeks | Clinical Finding | Condition | SNOMED |
| 4174506 | Gestation period, 11 weeks | Clinical Finding | Condition | SNOMED |
| 4242241 | Gestation period, 10 weeks | Clinical Finding | Condition | SNOMED |
| 4337360 | Gestation period, 1 week | Clinical Finding | Condition | SNOMED |
| 762907 | Gestation period greater than or equal to 37 weeks | Clinical Finding | Condition | SNOMED |
| 4062558 | False labor at or after 37 completed weeks of gestation | Clinical Finding | Condition | SNOMED |
| 4322726 | Gestation less than 9 weeks | Clinical Finding | Condition | SNOMED |
| 44791172 | Under 9 weeks gestational age | Clinical Finding | Condition | SNOMED |
| 438543 | Gestation period, 36 weeks | Clinical Finding | Condition | SNOMED |
| 442558 | Gestation period, 32 weeks | Clinical Finding | Condition | SNOMED |
| 444461 | Gestation period, 28 weeks | Clinical Finding | Condition | SNOMED |
| 439922 | Gestation period, 24 weeks | Clinical Finding | Condition | SNOMED |
| 4051642 | Gestation period, 20 weeks | Clinical Finding | Condition | SNOMED |
| 4049621 | Gestation period, 16 weeks | Clinical Finding | Condition | SNOMED |
| 4197245 | Gestation period, 12 weeks | Clinical Finding | Condition | SNOMED |
| 4132434 | Gestation period, 8 weeks | Clinical Finding | Condition | SNOMED |
| 4195157 | Gestation period, 4 weeks | Clinical Finding | Condition | SNOMED |
