# Supplementary material for: Temporal Events Detector for Pregnancy Care (TED-PC): A rule-based algorithm to infer gestational age and delivery date from electronic health records of pregnant women with and without COVID-19
Source: PLoS One. 2022 Oct 31;17(10):e0276923. doi: 10.1371/journal.pone.0276923 (PMC9621451; doi:10.1371/journal.pone.0276923)
Supplement: S2 Table — (DOCX) [file pone.0276923.s002.docx]

**Supporting information 2**

Table. ICD, CPT, and DRG codes suggestive of childbirth delivery dates.

| **Medical Language System** | **Code** | **Description** |
| --- | --- | --- |
| ICD-10_DX | Z37.0 | Single live birth |
| ICD-10_DX | Z37.1 | Single stillbirth |
| ICD-10_DX | Z37.2 | Twins, both liveborn |
| ICD-10_DX | Z37.3 | Twins, one liveborn and one stillborn |
| ICD-10_DX | Z37.4 | Twins, both stillborn |
| ICD-10_DX | Z37.5 | Other multiple births, all liveborn |
| ICD-10_DX | Z37.6 | Other multiple births, some liveborn |
| ICD-10_DX | Z37.7 | Other multiple births, all stillborn |
| ICD-10_DX | Z37.9 | Outcome of delivery, unspecified |
| ICD-10_DX | O80 | Encounter for full-term uncomplicated delivery |
| ICD-10_DX | O82 | Encounter for cesarean delivery without indication |
| ICD-10_DX | O7582 | Onset (spontaneous) of labor after 37 completed weeks of gestation but before 39 completed weeks gestation, with delivery by (planned) cesarean section |
| ICD_10_PCS | 10D07Z3 | Extraction of Products of Conception, Low Forceps, Via Natural or Artificial Opening |
| ICD_10_PCS | 10D07Z4 | Extraction of Products of Conception, Mid Forceps, Via Natural or Artificial Opening |
| ICD_10_PCS | 10D07Z5 | Extraction of Products of Conception, High Forceps, Via Natural or Artificial Opening |
| ICD_10_PCS | 10D07Z6 | Extraction of Products of Conception, Vacuum, Via Natural or Artificial Opening |
| ICD_10_PCS | 10D07Z7 | Extraction of Products of Conception, Internal Version, Via Natural or Artificial Opening |
| ICD_10_PCS | 10D07Z8 | Extraction of Products of Conception, Other, Via Natural or Artificial Opening |
| ICD_10_PCS | 10E0XZZ | Delivery of Products of Conception, External Approach |
| ICD_10_PCS | 10D00Z0 | Extraction of Products of Conception, High, Open Approach |
| ICD_10_PCS | 10D00Z1 | Extraction of Products of Conception, Low, Open Approach |
| ICD_10_PCS | 10D00Z2 | Extraction of Products of Conception, Extraperitoneal, Open Approach |
| DRG | 765 | CESAREAN SECTION WITH CC/MCC |
| DRG | 766 | CESAREAN SECTION WITHOUT CC/MCC |
| DRG | 767 | VAGINAL DELIVERY WITH STERILIZATION AND/OR D&C |
| DRG | 768 | VAGINAL DELIVERY WITH O.R. PROCEDURES EXCEPT STERILIZATION AND/OR D&C |
| DRG | 774 | VAGINAL DELIVERY WITH COMPLICATING DIAGNOSES |
| DRG | 775 | VAGINAL DELIVERY WITHOUT COMPLICATING DIAGNOSES |
| DRG | 783 | CESAREAN SECTION WITH STERILIZATION WITH MCC |
| DRG | 784 | CESAREAN SECTION WITH STERILIZATION WITH CC |
| DRG | 785 | CESAREAN SECTION WITH STERILIZATION WITHOUT CC/MCC |
| DRG | 786 | CESAREAN SECTION WITHOUT STERILIZATION WITH MCC |
| DRG | 787 | CESAREAN SECTION WITHOUT STERILIZATION WITH CC |
| DRG | 788 | CESAREAN SECTION WITHOUT STERILIZATION WITHOUT CC/MCC |
| DRG | 796 | VAGINAL DELIVERY WITH STERILIZATION/D&C WITH MCC |
| DRG | 797 | VAGINAL DELIVERY WITH STERILIZATION AND/OR D&C WITH CC |
| DRG | 798 | VAGINAL DELIVERY WITH STERILIZATION/D&C WITHOUT CC/MCC |
| DRG | 805 | VAGINAL DELIVERY WITHOUT STERILIZATION/D&C WITH MCC |
| DRG | 806 | VAGINAL DELIVERY WITHOUT STERILIZATION/D&C WITH CC |
| DRG | 807 | VAGINAL DELIVERY WITHOUT STERILIZATION OR D&C WITHOUT CC/MCC |
| CPT | 59400 | Vaginal Delivery, Antepartum and Postpartum Care Procedures |
| CPT | 59409 | Vaginal delivery only (with or without episiotomy and/or forceps) |
| CPT | 59410 | Vaginal delivery only (with or without episiotomy and/or forceps) |
| CPT | 59514 | Cesarean delivery only |
| CPT | 59610 | Delivery Procedures After Previous Cesarean Delivery |
| CPT | 59612 | Vaginal delivery only, after previous cesarean delivery (with or without episiotomy and/or forceps) |
| CPT | 59614 | Vaginal delivery only, after previous cesarean delivery (with or without episiotomy and/or forceps) |
| CPT | 59620 | Cesarean delivery only, following attempted vaginal delivery after previous cesarean delivery |
