# Supplementary material for: Temporal Events Detector for Pregnancy Care (TED-PC): A rule-based algorithm to infer gestational age and delivery date from electronic health records of pregnant women with and without COVID-19
Source: PLoS One. 2022 Oct 31;17(10):e0276923. doi: 10.1371/journal.pone.0276923 (PMC9621451; doi:10.1371/journal.pone.0276923)
Supplement: S3 Table — (DOCX) [file pone.0276923.s003.docx]

**Supporting information 3**

Table. OMOP CDM concepts for delivery date-related EHR.

| **Concept ID** | **Concept Name** | **Class** | **Domain** | **Vocabulary** |
| --- | --- | --- | --- | --- |
| 443445 | [Outcome of delivery - finding](https://athena.ohdsi.org/search-terms/terms/443445) | clinical finding | Condition | SNOMED |
| 4145318 | [Outcome of delivery](https://athena.ohdsi.org/search-terms/terms/4145318) | clinical finding | Observation | SNOMED |
| 4163851 | [Multiple birth](https://athena.ohdsi.org/search-terms/terms/4163851) | clinical finding | Condition | SNOMED |
| 4014456 | [Triplets - all live born](https://athena.ohdsi.org/search-terms/terms/4014456) | clinical finding | Condition | SNOMED |
| 45757166 | [Quadruplets, all live born](https://athena.ohdsi.org/search-terms/terms/45757166) | clinical finding | Condition | SNOMED |
| 45757167 | [Quintuplets, all live born](https://athena.ohdsi.org/search-terms/terms/45757167) | clinical finding | Condition | SNOMED |
| 45772082 | [Sextuplets, all live born](https://athena.ohdsi.org/search-terms/terms/45772082) | clinical finding | Condition | SNOMED |
| 4094046 | [Triplet birth](https://athena.ohdsi.org/search-terms/terms/4094046) | clinical finding | Condition | SNOMED |
| 45765500 | [Quadruplet birth](https://athena.ohdsi.org/search-terms/terms/45765500) | clinical finding | Condition | SNOMED |
| 45765501 | [Quintuplet birth](https://athena.ohdsi.org/search-terms/terms/45765501) | clinical finding | Condition | SNOMED |
| 45765502 | [Sextuplet birth](https://athena.ohdsi.org/search-terms/terms/45765502) | clinical finding | Condition | SNOMED |
| 4014295 | [Single live birth](https://athena.ohdsi.org/search-terms/terms/4014295) | clinical finding | Condition | SNOMED |
| 4014454 | [Single stillbirth](https://athena.ohdsi.org/search-terms/terms/4014454) | clinical finding | Condition | SNOMED |
| 4014296 | [Twins - both live born](https://athena.ohdsi.org/search-terms/terms/4014296) | clinical finding | Condition | SNOMED |
| 4014455 | [Twins - one still and one live born](https://athena.ohdsi.org/search-terms/terms/4014455) | clinical finding | Condition | SNOMED |
| 4015162 | [Twins - both stillborn](https://athena.ohdsi.org/search-terms/terms/4015162) | clinical finding | Condition | SNOMED |
| 441641 | [Delivery normal](https://athena.ohdsi.org/search-terms/terms/441641) | clinical finding | Condition | SNOMED |
| 4205240 | [Spontaneous vertex delivery](https://athena.ohdsi.org/search-terms/terms/4205240) | clinical finding | Condition | SNOMED |
| 4073422 | [Spontaneous breech delivery](https://athena.ohdsi.org/search-terms/terms/4073422) | procedure | Procedure | SNOMED |
| 193277 | [Deliveries by cesarean](https://athena.ohdsi.org/search-terms/terms/193277) | clinical finding | Condition | SNOMED |
| 4061457 | [Delivery by elective cesarean section](https://athena.ohdsi.org/search-terms/terms/4061457) | clinical finding | Condition | SNOMED |
| 4066112 | [Delivery by emergency cesarean section](https://athena.ohdsi.org/search-terms/terms/4066112) | clinical finding | Condition | SNOMED |
| 4061458 | [Delivery by cesarean hysterectomy](https://athena.ohdsi.org/search-terms/terms/4061458) | clinical finding | Condition | SNOMED |
| 45757118 | [Spontaneous onset of labor between 37 and 39 weeks gestation with planned cesarean section](https://athena.ohdsi.org/search-terms/terms/45757118) | clinical finding | Condition | SNOMED |
| 2784567 | [Extraction of Products of Conception, Low Forceps, Via Natural or Artificial Opening](https://athena.ohdsi.org/search-terms/terms/2784567) | ICD10PCS | Procedure | ICD10PCS |
| 2784568 | [Extraction of Products of Conception, Mid Forceps, Via Natural or Artificial Opening](https://athena.ohdsi.org/search-terms/terms/2784568) | ICD10PCS | Procedure | ICD10PCS |
| 2784569 | [Extraction of Products of Conception, High Forceps, Via Natural or Artificial Opening](https://athena.ohdsi.org/search-terms/terms/2784569) | ICD10PCS | Procedure | ICD10PCS |
| 2784570 | [Extraction of Products of Conception, Vacuum, Via Natural or Artificial Opening](https://athena.ohdsi.org/search-terms/terms/2784570) | ICD10PCS | Procedure | ICD10PCS |
| 2784571 | [Extraction of Products of Conception, Internal Version, Via Natural or Artificial Opening](https://athena.ohdsi.org/search-terms/terms/2784571) | ICD10PCS | Procedure | ICD10PCS |
| 2784572 | [Extraction of Products of Conception, Other, Via Natural or Artificial Opening](https://athena.ohdsi.org/search-terms/terms/2784572) | ICD10PCS | Procedure | ICD10PCS |
| 2784578 | [Delivery of Products of Conception, External Approach](https://athena.ohdsi.org/search-terms/terms/2784578) | ICD10PCS | Procedure | ICD10PCS |
| 2784564 | [Extraction of Products of Conception, High, Open Approach](https://athena.ohdsi.org/search-terms/terms/2784564) | ICD10PCS | Procedure | ICD10PCS |
| 2784565 | [Extraction of Products of Conception, Low, Open Approach](https://athena.ohdsi.org/search-terms/terms/2784565) | ICD10PCS | Procedure | ICD10PCS |
| 2784566 | [Extraction of Products of Conception, Extraperitoneal, Open Approach](https://athena.ohdsi.org/search-terms/terms/2784566) | ICD10PCS | Procedure | ICD10PCS |
| 38001485 | [Cesarean section w CC/MCC](https://athena.ohdsi.org/search-terms/terms/38001485) | MS-DRG (Domain Observation) | Observation | DRG |
| 38001486 | [Cesarean section w/o CC/MCC](https://athena.ohdsi.org/search-terms/terms/38001486) | MS-DRG (Domain Observation) | Observation | DRG |
| 38001487 | [Vaginal delivery w sterilization &/or D&C](https://athena.ohdsi.org/search-terms/terms/38001487) | MS-DRG (Domain Observation) | Observation | DRG |
| 38001488 | [Vaginal delivery w O.R. proc except steril &/or D&C](https://athena.ohdsi.org/search-terms/terms/38001488) | MS-DRG (Domain Observation) | Observation | DRG |
| 38001491 | [Vaginal delivery w complicating diagnoses](https://athena.ohdsi.org/search-terms/terms/38001491) | MS-DRG (Domain Observation) | Observation | DRG |
| 38001492 | [Vaginal delivery w/o complicating diagnoses](https://athena.ohdsi.org/search-terms/terms/38001492) | MS-DRG (Domain Observation) | Observation | DRG |
| 2110307 | [Routine obstetric care including antepartum care, vaginal delivery (with or without episiotomy, and/or forceps) and postpartum care](https://athena.ohdsi.org/search-terms/terms/2110307) | CPT4(Domain Procedure) | Procedure | CPT4 |
| 2110308 | [Vaginal delivery only (with or without episiotomy and/or forceps)](https://athena.ohdsi.org/search-terms/terms/2110308) | CPT4(Domain Procedure) | Procedure | CPT4 |
| 2110309 | [Vaginal delivery only (with or without episiotomy and/or forceps); including postpartum care](https://athena.ohdsi.org/search-terms/terms/2110309) | CPT4(Domain Procedure) | Procedure | CPT4 |
| 2110319 | [Routine obstetric care including antepartum care, vaginal delivery (with or without episiotomy, and/or forceps) and postpartum care, after previous cesarean delivery](https://athena.ohdsi.org/search-terms/terms/2110319) | CPT4(Domain Procedure) | Procedure | CPT4 |
| 2110320 | [Vaginal delivery only, after previous cesarean delivery (with or without episiotomy and/or forceps)](https://athena.ohdsi.org/search-terms/terms/2110320) | CPT4(Domain Procedure) | Procedure | CPT4 |
| 2110321 | [Vaginal delivery only, after previous cesarean delivery (with or without episiotomy and/or forceps); including postpartum care](https://athena.ohdsi.org/search-terms/terms/2110321) | CPT4(Domain Procedure) | Procedure | CPT4 |
| 2110316 | [Cesarean delivery only](https://athena.ohdsi.org/search-terms/terms/2110316) | CPT4(Domain Procedure) | Procedure | CPT4 |
| 2110323 | [Cesarean delivery only, following attempted vaginal delivery after previous cesarean delivery](https://athena.ohdsi.org/search-terms/terms/2110323) | CPT4(Domain Procedure) | Procedure | CPT4 |
| 4264823 | [Birth outcome (observable entity)](https://athena.ohdsi.org/search-terms/terms/4264823) | Observable Entity | Observation | SNOMED |
| 4015270 | [Birth of child (finding)](https://athena.ohdsi.org/search-terms/terms/4015270) | clinical finding | condition | SNOMED |
| 44793347 | [Total number of registerable births at delivery](https://athena.ohdsi.org/search-terms/terms/44793347) | Observable Entity | observation | SNOMED |
| 4063163 | [Multiple delivery, all by cesarean section](https://athena.ohdsi.org/search-terms/terms/4063163) | clinical finding | condition | SNOMED |
| 4063162 | [Multiple delivery, all by forceps and vacuum extractor](https://athena.ohdsi.org/search-terms/terms/4063162) | clinical finding | condition | SNOMED |
| 4059751 | [Multiple delivery, all spontaneous](https://athena.ohdsi.org/search-terms/terms/4059751) | clinical finding | condition | SNOMED |
| 4069200 | [Premature birth of multiple newborns](https://athena.ohdsi.org/search-terms/terms/4069200) | clinical finding | condition | SNOMED |
| 4066292 | [Term birth of multiple newborns](https://athena.ohdsi.org/search-terms/terms/4066292) | clinical finding | condition | SNOMED |
| 4101844 | [Twin birth](https://athena.ohdsi.org/search-terms/terms/4101844) | clinical finding | condition | SNOMED |
| 40482735 | [Liveborn born in hospital (situation)](https://athena.ohdsi.org/search-terms/terms/40482735) | context-dependent | observation | SNOMED |
| 40483126 | [Liveborn born in hospital by cesarean section (situation)](https://athena.ohdsi.org/search-terms/terms/40483126) | context-dependent | observation | SNOMED |
| 42539267 | [Multiple liveborn in hospital by vaginal delivery (situation)](https://athena.ohdsi.org/search-terms/terms/42539267) | context-dependent | observation | SNOMED |
| 36713468 | [Multiple liveborn other than twins born in hospital (situation)](https://athena.ohdsi.org/search-terms/terms/36713468) | context-dependent | observation | SNOMED |
| 40483521 | [Single liveborn born in hospital by cesarean section (situation)](https://athena.ohdsi.org/search-terms/terms/40483521) | context-dependent | observation | SNOMED |
| 36713074 | [Single liveborn born in hospital by vaginal delivery (situation)](https://athena.ohdsi.org/search-terms/terms/36713074) | context-dependent | observation | SNOMED |
| 36713465 | [Singleton liveborn born in hospital (situation)](https://athena.ohdsi.org/search-terms/terms/36713465) | context-dependent | observation | SNOMED |
| 42539210 | [Triplet liveborn in hospital by cesarean section (situation)](https://athena.ohdsi.org/search-terms/terms/42539210) | context-dependent | observation | SNOMED |
| 40483084 | [Twin liveborn born in hospital (situation)](https://athena.ohdsi.org/search-terms/terms/40483084) | context-dependent | observation | SNOMED |
| 40483101 | [Twin liveborn born in hospital by cesarean section (situation)](https://athena.ohdsi.org/search-terms/terms/40483101) | context-dependent | observation | SNOMED |
| 4014719 | [Labor details (finding)](https://athena.ohdsi.org/search-terms/terms/4014719) | drug product | Drug | VANDF |
| 4262313 | [Time of delivery (observable entity)](https://athena.ohdsi.org/search-terms/terms/4262313) | Observable Entity | observation | SNOMED |
| 4014291 | [Birth detail (observable entity)](https://athena.ohdsi.org/search-terms/terms/4014291) | drug product | Drug | VANDF |
| 4250009 | [Born by breech delivery](https://athena.ohdsi.org/search-terms/terms/4250009) | context-dependent | observation | SNOMED |
| 4192676 | [Born by cesarean section](https://athena.ohdsi.org/search-terms/terms/4192676) | context-dependent | observation | SNOMED |
| 4212794 | [Born by elective cesarean section](https://athena.ohdsi.org/search-terms/terms/4212794) | context-dependent | observation | SNOMED |
| 4250010 | [Born by emergency cesarean section](https://athena.ohdsi.org/search-terms/terms/4250010) | context-dependent | observation | SNOMED |
| 4217586 | [Born by forceps delivery](https://athena.ohdsi.org/search-terms/terms/4217586) | context-dependent | observation | SNOMED |
| 37310369 | [Born by high forceps delivery](https://athena.ohdsi.org/search-terms/terms/37310369) | context-dependent | observation | SNOMED |
| 37310393 | [Born by low forceps delivery](https://athena.ohdsi.org/search-terms/terms/37310393) | context-dependent | observation | SNOMED |
| 37310404 | [Born by mid-cavity forceps delivery](https://athena.ohdsi.org/search-terms/terms/37310404) | context-dependent | observation | SNOMED |
| 4216797 | [Born by normal vaginal delivery](https://athena.ohdsi.org/search-terms/terms/4216797) | context-dependent | observation | SNOMED |
| 4236293 | [Born by ventouse delivery](https://athena.ohdsi.org/search-terms/terms/4236293) | context-dependent | observation | SNOMED |
| 44813089 | [Midwife in attendance at birth](https://athena.ohdsi.org/search-terms/terms/44813089) | context-dependent | observation | SNOMED |
| 44802498 | [Midwife not in attendance at birth](https://athena.ohdsi.org/search-terms/terms/44802498) | context-dependent | observation | SNOMED |
| 4088584 | [Caul membrane over baby's head at delivery](https://athena.ohdsi.org/search-terms/terms/4088584) | context-dependent | observation | SNOMED |
| 4128845 | [Delivery problem](https://athena.ohdsi.org/search-terms/terms/4128845) | clinical finding | Condition | SNOMED |
| 4200201 | [Finding of birth outcome](https://athena.ohdsi.org/search-terms/terms/4200201) | clinical finding | condition | SNOMED |
| 4126390 | [Finding of pattern of delivery](https://athena.ohdsi.org/search-terms/terms/4126390) | clinical finding | condition | SNOMED |
| 4122720 | [Finding of second stage of labor](https://athena.ohdsi.org/search-terms/terms/4122720) | clinical finding | condition | SNOMED |
| 4096383 | [Finding of speed of delivery](https://athena.ohdsi.org/search-terms/terms/4096383) | clinical finding | condition | SNOMED |
| 4125602 | [Finding of third stage of labor](https://athena.ohdsi.org/search-terms/terms/4125602) | clinical finding | condition | SNOMED |
| 4009879 | [Good neonatal condition at birth](https://athena.ohdsi.org/search-terms/terms/4009879) | clinical finding | condition | SNOMED |
| 433260 | [Mother delivered](https://athena.ohdsi.org/search-terms/terms/433260) | clinical finding | condition | SNOMED |
| 3174660 | [Traumatic birth](https://athena.ohdsi.org/search-terms/terms/3174660) | clinical finding | condition | Nebraska Lexicon |
| 4014720 | [Normal birth](https://athena.ohdsi.org/search-terms/terms/4014720) | clinical finding | condition | SNOMED |
| 4118903 | [Normal delivery - occipitoanterior](https://athena.ohdsi.org/search-terms/terms/4118903) | clinical finding | condition | SNOMED |
| 4063160 | [Normal delivery but ante- or post- natal conditions present](https://athena.ohdsi.org/search-terms/terms/4063160) | clinical finding | condition | SNOMED |
| 442069 | [Vacuum extractor delivery - delivered](https://athena.ohdsi.org/search-terms/terms/442069) | clinical finding | condition | SNOMED |
| 4065737 | [Delivery by combination of forceps and vacuum extractor](https://athena.ohdsi.org/search-terms/terms/4065737) | clinical finding | condition | SNOMED |
| 45757174 | [Vacuum assisted vaginal delivery](https://athena.ohdsi.org/search-terms/terms/45757174) | clinical finding | condition | SNOMED |
| 4216316 | [Birth](https://athena.ohdsi.org/search-terms/terms/4216316) | clinical finding | condition | SNOMED |
| 4009589 | [Post-term delivery](https://athena.ohdsi.org/search-terms/terms/4009589) | clinical finding | condition | SNOMED |
| 4086393 | [Premature delivery](https://athena.ohdsi.org/search-terms/terms/4086393) | clinical finding | condition | SNOMED |
| 4092289 | [Livebirth](https://athena.ohdsi.org/search-terms/terms/4092289) | clinical finding | condition | SNOMED |
| 4272248 | [Premature birth of newborn](https://athena.ohdsi.org/search-terms/terms/4272248) | clinical finding | condition | SNOMED |
| 443213 | [Stillbirth](https://athena.ohdsi.org/search-terms/terms/443213) | clinical finding | condition | SNOMED |
| 4054968 | [Term birth of newborn](https://athena.ohdsi.org/search-terms/terms/4054968) | clinical finding | condition | SNOMED |
| 443445 | [Outcome of delivery - finding](https://athena.ohdsi.org/search-terms/terms/443445) | clinical finding | Condition | SNOMED |
| 4145318 | [Outcome of delivery](https://athena.ohdsi.org/search-terms/terms/4145318) | clinical finding | Observation | SNOMED |
| 4163851 | [Multiple birth](https://athena.ohdsi.org/search-terms/terms/4163851) | clinical finding | Condition | SNOMED |
| 4014456 | [Triplets - all live born](https://athena.ohdsi.org/search-terms/terms/4014456) | clinical finding | Condition | SNOMED |
| 45757166 | [Quadruplets, all live born](https://athena.ohdsi.org/search-terms/terms/45757166) | clinical finding | Condition | SNOMED |
| 45757167 | [Quintuplets, all live born](https://athena.ohdsi.org/search-terms/terms/45757167) | clinical finding | Condition | SNOMED |
| 45772082 | [Sextuplets, all live born](https://athena.ohdsi.org/search-terms/terms/45772082) | clinical finding | Condition | SNOMED |
| 4094046 | [Triplet birth](https://athena.ohdsi.org/search-terms/terms/4094046) | clinical finding | Condition | SNOMED |
| 45765500 | [Quadruplet birth](https://athena.ohdsi.org/search-terms/terms/45765500) | clinical finding | Condition | SNOMED |
| 45765501 | [Quintuplet birth](https://athena.ohdsi.org/search-terms/terms/45765501) | clinical finding | Condition | SNOMED |
| 45765502 | [Sextuplet birth](https://athena.ohdsi.org/search-terms/terms/45765502) | clinical finding | Condition | SNOMED |
| 4014295 | [Single live birth](https://athena.ohdsi.org/search-terms/terms/4014295) | clinical finding | Condition | SNOMED |
| 4014454 | [Single stillbirth](https://athena.ohdsi.org/search-terms/terms/4014454) | clinical finding | Condition | SNOMED |
| 4014296 | [Twins - both live born](https://athena.ohdsi.org/search-terms/terms/4014296) | clinical finding | Condition | SNOMED |
| 4014455 | [Twins - one still and one live born](https://athena.ohdsi.org/search-terms/terms/4014455) | clinical finding | Condition | SNOMED |
| 4015162 | [Twins - both stillborn](https://athena.ohdsi.org/search-terms/terms/4015162) | clinical finding | Condition | SNOMED |
| 441641 | [Delivery normal](https://athena.ohdsi.org/search-terms/terms/441641) | clinical finding | Condition | SNOMED |
| 4205240 | [Spontaneous vertex delivery](https://athena.ohdsi.org/search-terms/terms/4205240) | clinical finding | Condition | SNOMED |
| 4073422 | [Spontaneous breech delivery](https://athena.ohdsi.org/search-terms/terms/4073422) | procedure | Procedure | SNOMED |
| 193277 | [Deliveries by cesarean](https://athena.ohdsi.org/search-terms/terms/193277) | clinical finding | Condition | SNOMED |
| 4061457 | [Delivery by elective cesarean section](https://athena.ohdsi.org/search-terms/terms/4061457) | clinical finding | Condition | SNOMED |
| 4066112 | [Delivery by emergency cesarean section](https://athena.ohdsi.org/search-terms/terms/4066112) | clinical finding | Condition | SNOMED |
| 4061458 | [Delivery by cesarean hysterectomy](https://athena.ohdsi.org/search-terms/terms/4061458) | clinical finding | Condition | SNOMED |
| 45757118 | [Spontaneous onset of labor between 37 and 39 weeks gestation with planned cesarean section](https://athena.ohdsi.org/search-terms/terms/45757118) | clinical finding | Condition | SNOMED |
| 2784567 | [Extraction of Products of Conception, Low Forceps, Via Natural or Artificial Opening](https://athena.ohdsi.org/search-terms/terms/2784567) | ICD10PCS | Procedure | ICD10PCS |
| 2784568 | [Extraction of Products of Conception, Mid Forceps, Via Natural or Artificial Opening](https://athena.ohdsi.org/search-terms/terms/2784568) | ICD10PCS | Procedure | ICD10PCS |
| 2784569 | [Extraction of Products of Conception, High Forceps, Via Natural or Artificial Opening](https://athena.ohdsi.org/search-terms/terms/2784569) | ICD10PCS | Procedure | ICD10PCS |
| 2784570 | [Extraction of Products of Conception, Vacuum, Via Natural or Artificial Opening](https://athena.ohdsi.org/search-terms/terms/2784570) | ICD10PCS | Procedure | ICD10PCS |
| 2784571 | [Extraction of Products of Conception, Internal Version, Via Natural or Artificial Opening](https://athena.ohdsi.org/search-terms/terms/2784571) | ICD10PCS | Procedure | ICD10PCS |
| 2784572 | [Extraction of Products of Conception, Other, Via Natural or Artificial Opening](https://athena.ohdsi.org/search-terms/terms/2784572) | ICD10PCS | Procedure | ICD10PCS |
| 2784578 | [Delivery of Products of Conception, External Approach](https://athena.ohdsi.org/search-terms/terms/2784578) | ICD10PCS | Procedure | ICD10PCS |
| 2784564 | [Extraction of Products of Conception, High, Open Approach](https://athena.ohdsi.org/search-terms/terms/2784564) | ICD10PCS | Procedure | ICD10PCS |
| 2784565 | [Extraction of Products of Conception, Low, Open Approach](https://athena.ohdsi.org/search-terms/terms/2784565) | ICD10PCS | Procedure | ICD10PCS |
| 2784566 | [Extraction of Products of Conception, Extraperitoneal, Open Approach](https://athena.ohdsi.org/search-terms/terms/2784566) | ICD10PCS | Procedure | ICD10PCS |
| 38001485 | [Cesarean section w CC/MCC](https://athena.ohdsi.org/search-terms/terms/38001485) | MS-DRG (Domain Observation) | Observation | DRG |
| 38001486 | [Cesarean section w/o CC/MCC](https://athena.ohdsi.org/search-terms/terms/38001486) | MS-DRG (Domain Observation) | Observation | DRG |
| 38001487 | [Vaginal delivery w sterilization &/or D&C](https://athena.ohdsi.org/search-terms/terms/38001487) | MS-DRG (Domain Observation) | Observation | DRG |
| 38001488 | [Vaginal delivery w O.R. proc except steril &/or D&C](https://athena.ohdsi.org/search-terms/terms/38001488) | MS-DRG (Domain Observation) | Observation | DRG |
| 38001491 | [Vaginal delivery w complicating diagnoses](https://athena.ohdsi.org/search-terms/terms/38001491) | MS-DRG (Domain Observation) | Observation | DRG |
| 38001492 | [Vaginal delivery w/o complicating diagnoses](https://athena.ohdsi.org/search-terms/terms/38001492) | MS-DRG (Domain Observation) | Observation | DRG |
| 2110307 | [Routine obstetric care including antepartum care, vaginal delivery (with or without episiotomy, and/or forceps) and postpartum care](https://athena.ohdsi.org/search-terms/terms/2110307) | CPT4(Domain Procedure) | Procedure | CPT4 |
| 2110308 | [Vaginal delivery only (with or without episiotomy and/or forceps)](https://athena.ohdsi.org/search-terms/terms/2110308) | CPT4(Domain Procedure) | Procedure | CPT4 |
| 2110309 | [Vaginal delivery only (with or without episiotomy and/or forceps); including postpartum care](https://athena.ohdsi.org/search-terms/terms/2110309) | CPT4(Domain Procedure) | Procedure | CPT4 |
| 2110319 | [Routine obstetric care including antepartum care, vaginal delivery (with or without episiotomy, and/or forceps) and postpartum care, after previous cesarean delivery](https://athena.ohdsi.org/search-terms/terms/2110319) | CPT4(Domain Procedure) | Procedure | CPT4 |
| 2110320 | [Vaginal delivery only, after previous cesarean delivery (with or without episiotomy and/or forceps)](https://athena.ohdsi.org/search-terms/terms/2110320) | CPT4(Domain Procedure) | Procedure | CPT4 |
| 2110321 | [Vaginal delivery only, after previous cesarean delivery (with or without episiotomy and/or forceps); including postpartum care](https://athena.ohdsi.org/search-terms/terms/2110321) | CPT4(Domain Procedure) | Procedure | CPT4 |
| 2110316 | [Cesarean delivery only](https://athena.ohdsi.org/search-terms/terms/2110316) | CPT4(Domain Procedure) | Procedure | CPT4 |
| 2110323 | [Cesarean delivery only, following attempted vaginal delivery after previous cesarean delivery](https://athena.ohdsi.org/search-terms/terms/2110323) | CPT4(Domain Procedure) | Procedure | CPT4 |
| 4264823 | [Birth outcome (observable entity)](https://athena.ohdsi.org/search-terms/terms/4264823) | Observable Entity | Observation | SNOMED |
| 4015270 | [Birth of child (finding)](https://athena.ohdsi.org/search-terms/terms/4015270) | clinical finding | condition | SNOMED |
| 44793347 | [Total number of registerable births at delivery](https://athena.ohdsi.org/search-terms/terms/44793347) | Observable Entity | observation | SNOMED |
| 4063163 | [Multiple delivery, all by cesarean section](https://athena.ohdsi.org/search-terms/terms/4063163) | clinical finding | condition | SNOMED |
| 4063162 | [Multiple delivery, all by forceps and vacuum extractor](https://athena.ohdsi.org/search-terms/terms/4063162) | clinical finding | condition | SNOMED |
| 4059751 | [Multiple delivery, all spontaneous](https://athena.ohdsi.org/search-terms/terms/4059751) | clinical finding | condition | SNOMED |
| 4069200 | [Premature birth of multiple newborns](https://athena.ohdsi.org/search-terms/terms/4069200) | clinical finding | condition | SNOMED |
| 4066292 | [Term birth of multiple newborns](https://athena.ohdsi.org/search-terms/terms/4066292) | clinical finding | condition | SNOMED |
| 4101844 | [Twin birth](https://athena.ohdsi.org/search-terms/terms/4101844) | clinical finding | condition | SNOMED |
| 40482735 | [Liveborn born in hospital (situation)](https://athena.ohdsi.org/search-terms/terms/40482735) | context-dependent | observation | SNOMED |
| 40483126 | [Liveborn born in hospital by cesarean section (situation)](https://athena.ohdsi.org/search-terms/terms/40483126) | context-dependent | observation | SNOMED |
| 42539267 | [Multiple liveborn in hospital by vaginal delivery (situation)](https://athena.ohdsi.org/search-terms/terms/42539267) | context-dependent | observation | SNOMED |
| 36713468 | [Multiple liveborn other than twins born in hospital (situation)](https://athena.ohdsi.org/search-terms/terms/36713468) | context-dependent | observation | SNOMED |
| 40483521 | [Single liveborn born in hospital by cesarean section (situation)](https://athena.ohdsi.org/search-terms/terms/40483521) | context-dependent | observation | SNOMED |
| 36713074 | [Single liveborn born in hospital by vaginal delivery (situation)](https://athena.ohdsi.org/search-terms/terms/36713074) | context-dependent | observation | SNOMED |
| 36713465 | [Singleton liveborn born in hospital (situation)](https://athena.ohdsi.org/search-terms/terms/36713465) | context-dependent | observation | SNOMED |
| 42539210 | [Triplet liveborn in hospital by cesarean section (situation)](https://athena.ohdsi.org/search-terms/terms/42539210) | context-dependent | observation | SNOMED |
| 40483084 | [Twin liveborn born in hospital (situation)](https://athena.ohdsi.org/search-terms/terms/40483084) | context-dependent | observation | SNOMED |
| 40483101 | [Twin liveborn born in hospital by cesarean section (situation)](https://athena.ohdsi.org/search-terms/terms/40483101) | context-dependent | observation | SNOMED |
| 4014719 | [Labor details (finding)](https://athena.ohdsi.org/search-terms/terms/4014719) | drug product | Drug | VANDF |
| 4262313 | [Time of delivery (observable entity)](https://athena.ohdsi.org/search-terms/terms/4262313) | Observable Entity | observation | SNOMED |
| 4014291 | [Birth detail (observable entity)](https://athena.ohdsi.org/search-terms/terms/4014291) | drug product | Drug | VANDF |
| 4250009 | [Born by breech delivery](https://athena.ohdsi.org/search-terms/terms/4250009) | context-dependent | observation | SNOMED |
| 4192676 | [Born by cesarean section](https://athena.ohdsi.org/search-terms/terms/4192676) | context-dependent | observation | SNOMED |
| 4212794 | [Born by elective cesarean section](https://athena.ohdsi.org/search-terms/terms/4212794) | context-dependent | observation | SNOMED |
| 4250010 | [Born by emergency cesarean section](https://athena.ohdsi.org/search-terms/terms/4250010) | context-dependent | observation | SNOMED |
| 4217586 | [Born by forceps delivery](https://athena.ohdsi.org/search-terms/terms/4217586) | context-dependent | observation | SNOMED |
| 37310369 | [Born by high forceps delivery](https://athena.ohdsi.org/search-terms/terms/37310369) | context-dependent | observation | SNOMED |
| 37310393 | [Born by low forceps delivery](https://athena.ohdsi.org/search-terms/terms/37310393) | context-dependent | observation | SNOMED |
| 37310404 | [Born by mid-cavity forceps delivery](https://athena.ohdsi.org/search-terms/terms/37310404) | context-dependent | observation | SNOMED |
| 4216797 | [Born by normal vaginal delivery](https://athena.ohdsi.org/search-terms/terms/4216797) | context-dependent | observation | SNOMED |
| 4236293 | [Born by ventouse delivery](https://athena.ohdsi.org/search-terms/terms/4236293) | context-dependent | observation | SNOMED |
| 44813089 | [Midwife in attendance at birth](https://athena.ohdsi.org/search-terms/terms/44813089) | context-dependent | observation | SNOMED |
| 44802498 | [Midwife not in attendance at birth](https://athena.ohdsi.org/search-terms/terms/44802498) | context-dependent | observation | SNOMED |
| 4088584 | [Caul membrane over baby's head at delivery](https://athena.ohdsi.org/search-terms/terms/4088584) | context-dependent | observation | SNOMED |
| 4128845 | [Delivery problem](https://athena.ohdsi.org/search-terms/terms/4128845) | clinical finding | Condition | SNOMED |
| 4200201 | [Finding of birth outcome](https://athena.ohdsi.org/search-terms/terms/4200201) | clinical finding | condition | SNOMED |
| 4126390 | [Finding of pattern of delivery](https://athena.ohdsi.org/search-terms/terms/4126390) | clinical finding | condition | SNOMED |
| 4122720 | [Finding of second stage of labor](https://athena.ohdsi.org/search-terms/terms/4122720) | clinical finding | condition | SNOMED |
| 4096383 | [Finding of speed of delivery](https://athena.ohdsi.org/search-terms/terms/4096383) | clinical finding | condition | SNOMED |
| 4125602 | [Finding of third stage of labor](https://athena.ohdsi.org/search-terms/terms/4125602) | clinical finding | condition | SNOMED |
| 4009879 | [Good neonatal condition at birth](https://athena.ohdsi.org/search-terms/terms/4009879) | clinical finding | condition | SNOMED |
| 433260 | [Mother delivered](https://athena.ohdsi.org/search-terms/terms/433260) | clinical finding | condition | SNOMED |
| 3174660 | [Traumatic birth](https://athena.ohdsi.org/search-terms/terms/3174660) | clinical finding | condition | Nebraska Lexicon |
| 4014720 | [Normal birth](https://athena.ohdsi.org/search-terms/terms/4014720) | clinical finding | condition | SNOMED |
| 4118903 | [Normal delivery - occipitoanterior](https://athena.ohdsi.org/search-terms/terms/4118903) | clinical finding | condition | SNOMED |
| 4063160 | [Normal delivery but ante- or post- natal conditions present](https://athena.ohdsi.org/search-terms/terms/4063160) | clinical finding | condition | SNOMED |
| 442069 | [Vacuum extractor delivery - delivered](https://athena.ohdsi.org/search-terms/terms/442069) | clinical finding | condition | SNOMED |
| 4065737 | [Delivery by combination of forceps and vacuum extractor](https://athena.ohdsi.org/search-terms/terms/4065737) | clinical finding | condition | SNOMED |
| 45757174 | [Vacuum assisted vaginal delivery](https://athena.ohdsi.org/search-terms/terms/45757174) | clinical finding | condition | SNOMED |
| 4216316 | [Birth](https://athena.ohdsi.org/search-terms/terms/4216316) | clinical finding | condition | SNOMED |
| 4009589 | [Post-term delivery](https://athena.ohdsi.org/search-terms/terms/4009589) | clinical finding | condition | SNOMED |
| 4086393 | [Premature delivery](https://athena.ohdsi.org/search-terms/terms/4086393) | clinical finding | condition | SNOMED |
| 4092289 | [Livebirth](https://athena.ohdsi.org/search-terms/terms/4092289) | clinical finding | condition | SNOMED |
| 4272248 | [Premature birth of newborn](https://athena.ohdsi.org/search-terms/terms/4272248) | clinical finding | condition | SNOMED |
| 443213 | [Stillbirth](https://athena.ohdsi.org/search-terms/terms/443213) | clinical finding | condition | SNOMED |
| 4054968 | [Term birth of newborn](https://athena.ohdsi.org/search-terms/terms/4054968) | clinical finding | condition | SNOMED |
