# Supplementary material for: Temporal Events Detector for Pregnancy Care (TED-PC): A rule-based algorithm to infer gestational age and delivery date from electronic health records of pregnant women with and without COVID-19
Source: PLoS One. 2022 Oct 31;17(10):e0276923. doi: 10.1371/journal.pone.0276923 (PMC9621451; doi:10.1371/journal.pone.0276923)
Supplement: S4 Table — (DOCX) [file pone.0276923.s004.docx]

**Supporting information 4**

OMOP CDM concepts for underlying conditions.

Table 1. Obesity and overweight.

| **Concept ID** | **Concept Name** | **Class** | **Domain** | **Vocabulary** |
| --- | --- | --- | --- | --- |
| 44837875 | Body Mass Index 25.0-25.9, adult | 5-dig billing V code | Observation | ICD9CM |
| 4171317 | Lifelong obesity | Clinical Finding | Condition | SNOMED |
| 1571929 | Obesity complicating pregnancy, childbirth, and the puerperium | 5-char nonbill code | Condition | ICD10CM |
| 45949361 | obesity due to excess calories | Diagnosis | Condition | CIEL |
| 40298253 | Body mass index index 25-29 - overweight | Clinical Finding | Observation | SNOMED |
| 42872398 | Maternal obesity complicating pregnancy, childbirth and the puerperium, antepartum | Clinical Finding | Condition | SNOMED |
| 45908113 | Steatopygia | Diagnosis | Condition | CIEL |
| 4256640 | Body mass index 40+ - severely obese | Clinical Finding | Observation | SNOMED |
| 45606672 | Obesity complicating the puerperium | 6-char billing code | Condition | ICD10CM |
| 44829684 | Body Mass Index between 25-29, adult | 4-dig nonbill V code | Observation | ICD9CM |
| 45945889 | Hypogonadal Obesity | Diagnosis | Condition | CIEL |
| 44826272 | Body Mass Index, pediatric, greater than or equal to 95th percentile for age | 5-dig billing V code | Condition | ICD9CM |
| 4029276 | Generalized obesity | Clinical Finding | Condition | SNOMED |
| 763589 | Obese class II | Clinical Finding | Observation | SNOMED |
| 3109986 | O/E weight: [>20% over ideal] or [obese] | Clinical Finding | Condition | Nebraska Lexicon |
| 44826271 | Body Mass Index 45.0-49.9, adult | 5-dig billing V code | Observation | ICD9CM |
| 45948037 | Simple Obesity | Diagnosis | Condition | CIEL |
| 4177337 | Hypogonadal obesity | Clinical Finding | Condition | SNOMED |
| 44822953 | Obesity hypoventilation syndrome | 5-dig billing code | Condition | ICD9CM |
| 45609961 | Body mass index [BMI] 38.0-38.9, adult | 5-char billing code | Observation | ICD10CM |
| 45576460 | Obesity, unspecified | ICD10 code | Condition | ICD10 |
| 44833167 | Body Mass Index 40.0-44.9, adult | 5-dig billing V code | Observation | ICD9CM |
| 3479291 | Extreme obesity with alveolar hypoventilation | Clinical Finding | Condition | Nebraska Lexicon |
| 4087487 | Central obesity | Clinical Finding | Condition | SNOMED |
| 3477540 | Childhood obesity | Clinical Finding | Condition | Nebraska Lexicon |
| 45576459 | Localized adiposity | ICD10 Hierarchy | Condition | ICD10 |
| 44834364 | Body Mass Index 31.0-31.9, adult | 5-dig billing V code | Observation | ICD9CM |
| 3477838 | Hydrocephalus with obesity and hypogonadism syndrome | Clinical Finding | Condition | Nebraska Lexicon |
| 3085651 | Hyperplasmic obesity | Clinical Finding | Condition | Nebraska Lexicon |
| 3477492 | Buffalo obesity | Clinical Finding | Condition | Nebraska Lexicon |
| 45566454 | Body mass index [BMI] 60.0-69.9, adult | 5-char billing code | Observation | ICD10CM |
| 44821625 | Body Mass Index 29.0-29.9, adult | 5-dig billing V code | Observation | ICD9CM |
| 44836723 | Body Mass Index 33.0-33.9, adult | 5-dig billing V code | Observation | ICD9CM |
| 3478662 | Hyperinsulinar obesity | Clinical Finding | Condition | Nebraska Lexicon |
| 40598145 | Adiposity | Clinical Finding | Condition | SNOMED |
| 45933471 | Alstrom Syndrome | Diagnosis | Condition | CIEL |
| 3479515 | Android obesity | Clinical Finding | Condition | Nebraska Lexicon |
| 4215968 | Obese | Clinical Finding | Observation | SNOMED |
| 40321248 | Fat pad syndrome | Clinical Finding | Condition | SNOMED |
| 3478847 | Hypogonadal obesity | Clinical Finding | Condition | Nebraska Lexicon |
| 4062918 | Edema or excessive weight gain in pregnancy without mention of hypertension, not delivered during current episode of care | Clinical Finding | Condition | SNOMED |
| 3480057 | Alstrom syndrome | Clinical Finding | Condition | Nebraska Lexicon |
| 3477680 | Obesity of endocrine origin | Clinical Finding | Condition | Nebraska Lexicon |
| 44829108 | Edema or excessive weight gain in pregnancy, without mention of hypertension, antepartum condition or complication | 5-dig billing code | Condition | ICD9CM |
| 45925866 | Endogenous Obesity | Diagnosis | Condition | CIEL |
| 45576172 | Body mass index [BMI] 50.0-59.9, adult | 5-char billing code | Observation | ICD10CM |
| 4150937 | O/E - weight 10-20% over ideal | Clinical Finding | Observation | SNOMED |
| 35207022 | Morbid (severe) obesity with alveolar hypoventilation | 4-char billing code | Condition | ICD10CM |
| 3137946 | Excessive body weight gain | Clinical Finding | Condition | Nebraska Lexicon |
| 36674893 | Intellectual disability, seizures, macrocephaly, obesity syndrome | Clinical Finding | Condition | SNOMED |
| 37395980 | MORM syndrome | Clinical Finding | Condition | SNOMED |
| 3477651 | Obesity in mother complicating childbirth | Clinical Finding | Condition | Nebraska Lexicon |
| 4079899 | Android obesity | Clinical Finding | Condition | SNOMED |
| 45591051 | Other obesity due to excess calories | 5-char billing code | Condition | ICD10CM |
| 3068160 | Adiposity | Clinical Finding | Condition | Nebraska Lexicon |
| 36676689 | Rapid-onset childhood obesity, hypothalamic dysfunction, hypoventilation, autonomic dysregulation syndrome | Clinical Finding | Condition | SNOMED |
| 3092196 | Body mass index index 25-29 - overweight | Clinical Finding | Condition | Nebraska Lexicon |
| 3478574 | Constitutional obesity | Clinical Finding | Condition | Nebraska Lexicon |
| 3479887 | X-linked intellectual disability, epileptic seizures, hypogonadism and hypogenitalism, microcephaly, obesity syndrome | Clinical Finding | Condition | Nebraska Lexicon |
| 44824092 | Overweight, obesity and other hyperalimentation | 3-dig nonbill code | Observation | ICD9CM |
| 3445188 | On examination - weight greater than 20% over ideal | Clinical Finding | Observation | Nebraska Lexicon |
| 4270189 | Alstrom syndrome | Clinical Finding | Condition | SNOMED |
| 3122233 | Obesity | Clinical Finding | Condition | Nebraska Lexicon |
| 44836089 | Obesity complicating pregnancy, childbirth, or the puerperium, delivered, with or without mention of antepartum condition | 5-dig billing code | Condition | ICD9CM |
| 45934001 | Drug-Induced Obesity | Diagnosis | Condition | CIEL |
| 45910033 | Hypothalamic Obesity | Diagnosis | Condition | CIEL |
| 35209009 | Hypertrophy of (infrapatellar) fat pad | 4-char billing code | Condition | ICD10CM |
| 4217557 | Simple obesity | Clinical Finding | Condition | SNOMED |
| 45566453 | Body mass index [BMI] 36.0-36.9, adult | 5-char billing code | Observation | ICD10CM |
| 3145697 | Alimentary obesity | Clinical Finding | Condition | Nebraska Lexicon |
| 44822107 | Obesity complicating pregnancy, childbirth, or the puerperium, antepartum condition or complication | 5-dig billing code | Condition | ICD9CM |
| 37208175 | Lower body obesity | Clinical Finding | Condition | SNOMED |
| 44831059 | Morbid obesity | 5-dig billing code | Condition | ICD9CM |
| 40282757 | Localized adiposity | Clinical Finding | Condition | SNOMED |
| 45548719 | Excessive weight gain in pregnancy, third trimester | 5-char billing code | Condition | ICD10CM |
| 3122242 | Simple obesity NOS | Clinical Finding | Condition | Nebraska Lexicon |
| 4060985 | Body mass index 30+ - obesity | Clinical Finding | Observation | SNOMED |
| 3479281 | Syndromic X-linked intellectual disability type 7 | Clinical Finding | Condition | Nebraska Lexicon |
| 3085712 | Buffalo obesity (disorder) [Ambiguous] | Clinical Finding | Condition | Nebraska Lexicon |
| 40433887 | Excessive body weight gain | Clinical Finding | Observation | SNOMED |
| 4203289 | Hypothyroid obesity | Clinical Finding | Condition | SNOMED |
| 45590750 | Body mass index [BMI] 28.0-28.9, adult | 5-char billing code | Observation | ICD10CM |
| 3478362 | Hypothalamic infantilism with obesity syndrome | Clinical Finding | Condition | Nebraska Lexicon |
| 4093860 | Peripheral obesity | Clinical Finding | Condition | SNOMED |
| 45916795 | Adiposogenital Dystrophy | Diagnosis | Condition | CIEL |
| 45576171 | Body mass index [BMI] 25.0-25.9, adult | 5-char billing code | Observation | ICD10CM |
| 3126798 | Oedema or excessive weight gain in pregnancy without mention of hypertension, unspecified as to episode of care | Clinical Finding | Condition | Nebraska Lexicon |
| 3150599 | Obesity associated adipose tissue distribution pattern | Clinical Finding | Condition | Nebraska Lexicon |
| 45547648 | Obesity | ICD10 Hierarchy | Condition | ICD10 |
| 3477915 | Dwarfism-hepatomegaly-obesity-juvenile diabetes syndrome | Clinical Finding | Condition | Nebraska Lexicon |
| 3126799 | Oedema or excessive weight gain in pregnancy without mention of hypertension, delivered, with or without mention of antepartum condition | Clinical Finding | Condition | Nebraska Lexicon |
| 40481140 | Childhood obesity | Clinical Finding | Condition | SNOMED |
| 3479727 | Maternal obesity without hypertension | Clinical Finding | Condition | Nebraska Lexicon |
| 44821626 | Body Mass Index 38.0-38.9, adult | 5-dig billing V code | Observation | ICD9CM |
| 36717154 | Hydrocephalus with obesity and hypogonadism syndrome | Clinical Finding | Condition | SNOMED |
| 3432505 | Body mass index 30.00 to 34.99 | Clinical Finding | Observation | Nebraska Lexicon |
| 3477497 | Obesity by contributing factors | Clinical Finding | Condition | Nebraska Lexicon |
| 36674827 | Prader-Willi-like syndrome | Clinical Finding | Condition | SNOMED |
| 44833387 | Obesity, unspecified | 5-dig billing code | Condition | ICD9CM |
| 40389152 | Morbid obesity | Clinical Finding | Condition | SNOMED |
| 45566451 | Body mass index [BMI] 30.0-30.9, adult | 5-char billing code | Observation | ICD10CM |
| 4029277 | Fat pad syndrome | Clinical Finding | Condition | SNOMED |
| 3054262 | Obese | Clinical Finding | Observation | Nebraska Lexicon |
| 45590751 | Body mass index [BMI] 40.0-44.9, adult | 5-char billing code | Observation | ICD10CM |
| 40538359 | Obesity associated adipose tissue distribution pattern | Clinical Finding | Condition | SNOMED |
| 1571700 | Excessive weight gain in pregnancy | 4-char nonbill code | Condition | ICD10CM |
| 40298155 | O/E weight: [>20% over ideal] or [obese] | Clinical Finding | Observation | SNOMED |
| 45921030 | Central Obesity | Diagnosis | Condition | CIEL |
| 45581373 | Extreme obesity with alveolar hypoventilation | ICD10 code | Condition | ICD10 |
| 3476569 | Body mass index 25-29 - overweight | Clinical Finding | Observation | Nebraska Lexicon |
| 40550389 | Obesity of endocrine origin | Clinical Finding | Condition | SNOMED |
| 3153185 | Obesity of endocrine origin | Clinical Finding | Condition | Nebraska Lexicon |
| 3452265 | Body mass index 40+ - morbidly obese | Clinical Finding | Observation | Nebraska Lexicon |
| 45920062 | Maternal Obesity Syndrome | Diagnosis | Condition | CIEL |
| 44826786 | Obesity complicating pregnancy, childbirth, or the puerperium, unspecified as to episode of care or not applicable | 5-dig billing code | Condition | ICD9CM |
| 3479238 | Fat limbs with thin body | Clinical Finding | Condition | Nebraska Lexicon |
| 44828615 | Body Mass Index 32.0-32.9, adult | 5-dig billing V code | Observation | ICD9CM |
| 3164699 | Lifelong obesity | Clinical Finding | Condition | Nebraska Lexicon |
| 44833168 | Body Mass Index 60.0-69.9, adult | 5-dig billing V code | Observation | ICD9CM |
| 45938594 | Fatness | Diagnosis | Condition | CIEL |
| 4216214 | Mauriac's syndrome | Clinical Finding | Condition | SNOMED |
| 3105166 | Hyperalimentation incl.obesity | Clinical Finding | Condition | Nebraska Lexicon |
| 3478290 | Gynaecoid obesity | Clinical Finding | Condition | Nebraska Lexicon |
| 45592201 | Excessive weight gain in pregnancy, second trimester | 5-char billing code | Condition | ICD10CM |
| 45595538 | Body mass index [BMI] 32.0-32.9, adult | 5-char billing code | Observation | ICD10CM |
| 3477577 | Obesity due to melanocortin 4 receptor deficiency | Clinical Finding | Condition | Nebraska Lexicon |
| 4146858 | O/E - weight greater than 20% over ideal | Clinical Finding | Observation | SNOMED |
| 4060288 | Edema or excessive weight gain in pregnancy without mention of hypertension, unspecified as to episode of care | Clinical Finding | Condition | SNOMED |
| 44836724 | Body Mass Index 70 and over, adult | 5-dig billing V code | Observation | ICD9CM |
| 3478378 | Morbid obesity | Clinical Finding | Condition | Nebraska Lexicon |
| 4160821 | Familial obesity | Clinical Finding | Condition | SNOMED |
| 40517172 | Adiposity | Clinical Finding | Condition | SNOMED |
| 3478419 | Obesity due to prohormone convertase I deficiency | Clinical Finding | Condition | Nebraska Lexicon |
| 45950639 | Hyperplastic-Hypertrophic Obesity | Diagnosis | Condition | CIEL |
| 3122234 | Obesity due to excess calories | Clinical Finding | Condition | Nebraska Lexicon |
| 3477005 | On examination - overweight | Clinical Finding | Observation | Nebraska Lexicon |
| 3479818 | Generalised obesity | Clinical Finding | Condition | Nebraska Lexicon |
| 3478160 | Maternal obesity complicating pregnancy, childbirth and the puerperium, antepartum | Clinical Finding | Condition | Nebraska Lexicon |
| 45947001 | Hypertrophy of Fat Pad, Knee | Diagnosis | Condition | CIEL |
| 37204685 | Obesity due to CEP19 deficiency | Clinical Finding | Condition | SNOMED |
| 45948976 | adult body mass index greater than 30 | Diagnosis | Condition | CIEL |
| 45568006 | Obesity complicating pregnancy, third trimester | 6-char billing code | Condition | ICD10CM |
| 40298254 | Body mass index 30+ - obesity | Clinical Finding | Observation | SNOMED |
| 40616701 | Adult-onset obesity | Clinical Finding | Condition | SNOMED |
| 443321 | Edema or excessive weight gain in pregnancy without mention of hypertension, delivered with mention of postpartum complication | Clinical Finding | Condition | SNOMED |
| 44836722 | Body Mass Index 27.0-27.9, adult | 5-dig billing V code | Observation | ICD9CM |
| 4097929 | Constitutional obesity | Clinical Finding | Condition | SNOMED |
| 3092036 | O/E - obese | Clinical Finding | Condition | Nebraska Lexicon |
| 3122239 | Other hyperalimentation | Clinical Finding | Condition | Nebraska Lexicon |
| 40389161 | Simple obesity NOS | Clinical Finding | Condition | SNOMED |
| 40362694 | Excessive weight gain in pregnancy | Clinical Finding | Condition | SNOMED |
| 40389159 | Pickwickian syndrome | Clinical Finding | Condition | SNOMED |
| 44824093 | Localized adiposity | 4-dig billing code | Condition | ICD9CM |
| 1568023 | Obesity due to excess calories | 4-char nonbill code | Condition | ICD10CM |
| 3479009 | Drug-induced obesity | Clinical Finding | Condition | Nebraska Lexicon |
| 40321247 | (Hyperalimentation including obesity) or (adiposity) | Clinical Finding | Observation | SNOMED |
| 40505526 | Constitutional obesity | Clinical Finding | Condition | SNOMED |
| 45915124 | Morbid Obesity | Diagnosis | Condition | CIEL |
| 439893 | Maternal obesity syndrome | Clinical Finding | Condition | SNOMED |
| 3480034 | Adult-onset obesity | Clinical Finding | Condition | Nebraska Lexicon |
| 45945330 | Adult-Onset Obesity | Diagnosis | Condition | CIEL |
| 45577673 | Obesity complicating pregnancy, first trimester | 6-char billing code | Condition | ICD10CM |
| 4211019 | Steatopygia | Clinical Finding | Condition | SNOMED |
| 3063176 | Constitutional obesity | Clinical Finding | Condition | Nebraska Lexicon |
| 4097996 | Drug-induced obesity | Clinical Finding | Condition | SNOMED |
| 40631574 | Pickwickian syndrome | Clinical Finding | Condition | SNOMED |
| 44811757 | Obese class III (body mass index equal to or greater than 40.0) | Clinical Finding | Observation | SNOMED |
| 36678790 | Colobomatous microphthalmia, obesity, hypogenitalism, intellectual disability syndrome | Clinical Finding | Condition | SNOMED |
| 4220527 | Hypothalamic obesity | Clinical Finding | Condition | SNOMED |
| 4163032 | Hyperplastic-hypertrophic obesity | Clinical Finding | Condition | SNOMED |
| 3122240 | Pickwickian syndrome | Clinical Finding | Condition | Nebraska Lexicon |
| 3144683 | Excessive weight gain in pregnancy | Clinical Finding | Condition | Nebraska Lexicon |
| 3477571 | Obesity, early-onset, adrenal insufficiency, and red hair | Clinical Finding | Condition | Nebraska Lexicon |
| 4058105 | Edema or excessive weight gain in pregnancy NOS | Clinical Finding | Condition | SNOMED |
| 36716151 | Obesity due to prohormone convertase I deficiency | Clinical Finding | Condition | SNOMED |
| 45571681 | Drug-induced obesity | ICD10 code | Condition | ICD10 |
| 3126803 | Oedema or excessive weight gain in pregnancy without mention of hypertension with postnatal complication | Clinical Finding | Condition | Nebraska Lexicon |
| 35207021 | Drug-induced obesity | 4-char billing code | Condition | ICD10CM |
| 3479629 | Obesity of face and trunk, sparing limbs | Clinical Finding | Condition | Nebraska Lexicon |
| 45534322 | Obesity complicating childbirth | 6-char billing code | Condition | ICD10CM |
| 3478633 | Intellectual disability, obesity, brain malformation, facial dysmorphism syndrome | Clinical Finding | Condition | Nebraska Lexicon |
| 45936511 | Obesity of Endocrine Origin | Diagnosis | Condition | CIEL |
| 37018860 | Severe obesity | Clinical Finding | Condition | SNOMED |
| 45596930 | Excessive weight gain in pregnancy, unspecified trimester | 5-char billing code | Condition | ICD10CM |
| 4171972 | Adult-onset obesity | Clinical Finding | Condition | SNOMED |
| 44823910 | Body Mass Index 37.0-37.9, adult | 5-dig billing V code | Observation | ICD9CM |
| 3142059 | Oedema or excessive weight gain in pregnancy without mention of hypertension | Clinical Finding | Condition | Nebraska Lexicon |
| 3122235 | Morbid obesity | Clinical Finding | Condition | Nebraska Lexicon |
| 37397209 | Obesity due to melanocortin 4 receptor deficiency | Clinical Finding | Condition | SNOMED |
| 4189664 | Obesity by age of onset | Clinical Finding | Condition | SNOMED |
| 433736 | Obesity | Clinical Finding | Condition | SNOMED |
| 44813376 | [X]Other obesity | Clinical Finding | Observation | SNOMED |
| 1568022 | Overweight and obesity | 3-char nonbill code | Condition | ICD10CM |
| 45543688 | Hypertrophy of (infrapatellar) fat pad | ICD10 code | Condition | ICD10 |
| 37310480 | Hypertrophy of fat pad of right knee | Clinical Finding | Condition | SNOMED |
| 3477878 | Macrocephaly, obesity, mental disability, ocular abnormality syndrome | Clinical Finding | Condition | Nebraska Lexicon |
| 3160813 | Calorie overload | Clinical Finding | Condition | Nebraska Lexicon |
| 37311904 | Obesity in adolescence | Clinical Finding | Condition | SNOMED |
| 45600347 | Body mass index [BMI] 27.0-27.9, adult | 5-char billing code | Observation | ICD10CM |
| 3109985 | O/E - overweight (& [weight 10-20% over ideal]) | Clinical Finding | Condition | Nebraska Lexicon |
| 443322 | Edema or excessive weight gain in pregnancy without mention of hypertension, delivered, with or without mention of antepartum condition | Clinical Finding | Condition | SNOMED |
| 3146729 | Simple obesity NOS | Clinical Finding | Condition | Nebraska Lexicon |
| 35207023 | Other obesity | 4-char billing code | Condition | ICD10CM |
| 45927837 | Mauriac's Syndrome | Diagnosis | Condition | CIEL |
| 4029900 | Gynecoid obesity | Clinical Finding | Condition | SNOMED |
| 37206117 | Obesity due to leptin receptor gene deficiency | Clinical Finding | Condition | SNOMED |
| 4212443 | Obesity by adipocyte growth pattern | Clinical Finding | Condition | SNOMED |
| 36716554 | Overweight in adulthood with body mass index of 25 or more but less than 30 | Clinical Finding | Observation | SNOMED |
| 45910032 | Hypothyroid Obesity | Diagnosis | Condition | CIEL |
| 45927945 | Lifelong Obesity | Diagnosis | Condition | CIEL |
| 3432756 | On examination - weight 10-20% over ideal | Clinical Finding | Observation | Nebraska Lexicon |
| 44821032 | Obesity complicating pregnancy, childbirth, or the puerperium, delivered, with mention of postpartum complication | 5-dig billing code | Condition | ICD9CM |
| 45940998 | Localized Adiposity | Diagnosis | Condition | CIEL |
| 45910056 | Hypertrophic Obesity | Diagnosis | Condition | CIEL |
| 3479302 | Hypothyroid obesity | Clinical Finding | Condition | Nebraska Lexicon |
| 4183240 | Obesity of endocrine origin | Clinical Finding | Condition | SNOMED |
| 45586164 | Overweight | 4-char billing code | Observation | ICD10CM |
| 45927251 | Obesity | Diagnosis | Condition | CIEL |
| 40493831 | Exogenous obesity | Clinical Finding | Condition | SNOMED |
| 438731 | Localized adiposity | Clinical Finding | Condition | SNOMED |
| 3105168 | Obesity | Clinical Finding | Condition | Nebraska Lexicon |
| 3481804 | Adiposity | Clinical Finding | Condition | Nebraska Lexicon |
| 763588 | Obese class I | Clinical Finding | Observation | SNOMED |
| 3142260 | Hyperalimentation incl.obesity | Clinical Finding | Condition | Nebraska Lexicon |
| 44834896 | Obesity complicating pregnancy, childbirth, or the puerperium | 4-dig nonbill code | Condition | ICD9CM |
| 3198963 | Body mass index 35.00 to 39.99 | Clinical Finding | Observation | Nebraska Lexicon |
| 37110069 | MOMO syndrome | Clinical Finding | Condition | SNOMED |
| 40321250 | Hyperalimentation: [Pickwickian syndrome] or [other] | Clinical Finding | Condition | SNOMED |
| 3154249 | Morbid obesity | Clinical Finding | Condition | Nebraska Lexicon |
| 40272781 | Hyperplasmic obesity | Clinical Finding | Condition | SNOMED |
| 4235799 | Buffalo obesity | Clinical Finding | Condition | SNOMED |
| 45609962 | Body mass index [BMI] 39.0-39.9, adult | 5-char billing code | Observation | ICD10CM |
| 4185912 | Hyperplastic obesity | Clinical Finding | Condition | SNOMED |
| 36674490 | Intellectual disability, obesity, prognathism, eye and skin anomalies syndrome | Clinical Finding | Condition | SNOMED |
| 3480182 | Steatopygia | Clinical Finding | Condition | Nebraska Lexicon |
| 45605144 | Body mass index [BMI] 29.0-29.9, adult | 5-char billing code | Observation | ICD10CM |
| 4271317 | Adiposogenital dystrophy | Clinical Finding | Condition | SNOMED |
| 3126804 | Oedema or excessive weight gain in pregnancy NOS | Clinical Finding | Condition | Nebraska Lexicon |
| 3163578 | Hypoventilation associated with obesity syndrome | Clinical Finding | Condition | Nebraska Lexicon |
| 37395839 | Choroideremia hypopituitarism | Clinical Finding | Condition | SNOMED |
| 3479740 | Pre-existing severe obesity in mother complicating pregnancy | Clinical Finding | Condition | Nebraska Lexicon |
| 40389153 | (Localized adiposity disorder) or (fat pad) | Clinical Finding | Condition | SNOMED |
| 37311728 | Obese class III | Clinical Finding | Observation | SNOMED |
| 44819858 | Edema or excessive weight gain in pregnancy, without mention of hypertension, delivered, with or without mention of antepartum complication | 5-dig billing code | Condition | ICD9CM |
| 3478148 | Hyperplastic obesity | Clinical Finding | Condition | Nebraska Lexicon |
| 35225256 | Body mass index [BMI] 40 or greater, adult | 4-char nonbill code | Observation | ICD10CM |
| 3478062 | Familial obesity | Clinical Finding | Condition | Nebraska Lexicon |
| 3479357 | Fat pad syndrome | Clinical Finding | Condition | Nebraska Lexicon |
| 40288245 | Buffalo obesity | Clinical Finding | Condition | SNOMED |
| 4182506 | Endogenous obesity | Clinical Finding | Condition | SNOMED |
| 3478803 | Obesity, colitis, hypothyroidism, cardiac hypertrophy, developmental delay syndrome | Clinical Finding | Condition | Nebraska Lexicon |
| 44829685 | Body Mass Index 34.0-34.9, adult | 5-dig billing V code | Observation | ICD9CM |
| 44822764 | Body Mass Index 40 and over, adult | 4-dig nonbill V code | Observation | ICD9CM |
| 44836721 | Body Mass Index 26.0-26.9, adult | 5-dig billing V code | Observation | ICD9CM |
| 40298153 | O/E - overweight (& [weight 10-20% over ideal]) | Clinical Finding | Observation | SNOMED |
| 44835553 | Body Mass Index 30.0-30.9, adult | 5-dig billing V code | Observation | ICD9CM |
| 45940129 | Familial Obesity | Diagnosis | Condition | CIEL |
| 3145848 | Exogenous obesity | Clinical Finding | Condition | Nebraska Lexicon |
| 37204815 | Obesity due to SIM1 deficiency | Clinical Finding | Condition | SNOMED |
| 45936095 | Buffalo Obesity | Diagnosis | Condition | CIEL |
| 3478297 | Choroideraemia with deafness and obesity syndrome | Clinical Finding | Condition | Nebraska Lexicon |
| 40389149 | Obesity due to excess calories | Clinical Finding | Condition | SNOMED |
| 40306878 | O/E - obese | Clinical Finding | Observation | SNOMED |
| 3477670 | Obesity by age of onset | Clinical Finding | Condition | Nebraska Lexicon |
| 44836090 | Obesity complicating pregnancy, childbirth, or the puerperium, postpartum condition or complication | 5-dig billing code | Condition | ICD9CM |
| 40328689 | O/E weight: [>20% over ideal] or [obese] | Clinical Finding | Observation | SNOMED |
| 45927221 | Overweight | Diagnosis | Condition | CIEL |
| 3126801 | Oedema or excessive weight gain in pregnancy without mention of hypertension, not delivered during current episode of care | Clinical Finding | Condition | Nebraska Lexicon |
| 438204 | Edema or excessive weight gain in pregnancy without mention of hypertension | Clinical Finding | Condition | SNOMED |
| 45553618 | Obesity complicating pregnancy, unspecified trimester | 6-char billing code | Condition | ICD10CM |
| 42539192 | Severe obesity complicating pregnancy | Clinical Finding | Condition | SNOMED |
| 45547336 | Body mass index [BMI] 33.0-33.9, adult | 5-char billing code | Observation | ICD10CM |
| 3477564 | Hypothalamic obesity | Clinical Finding | Condition | Nebraska Lexicon |
| 3479784 | Endogenous obesity | Clinical Finding | Condition | Nebraska Lexicon |
| 40389162 | Adiposity | Clinical Finding | Condition | SNOMED |
| 3086974 | Buffalo obesity | Clinical Finding | Condition | Nebraska Lexicon |
| 3092197 | Body mass index 30+ - obesity | Clinical Finding | Condition | Nebraska Lexicon |
| 3086593 | Localised adiposity | Clinical Finding | Condition | Nebraska Lexicon |
| 3057731 | Adiposity | Clinical Finding | Condition | Nebraska Lexicon |
| 45592200 | Excessive weight gain in pregnancy, first trimester | 5-char billing code | Condition | ICD10CM |
| 3455795 | Overweight in childhood | Clinical Finding | Observation | Nebraska Lexicon |
| 45757112 | Obesity in mother complicating childbirth | Clinical Finding | Condition | SNOMED |
| 44828616 | Body Mass Index 39.0-39.9, adult | 5-dig billing V code | Observation | ICD9CM |
| 40328688 | O/E - overweight (& [weight 10-20% over ideal]) | Clinical Finding | Observation | SNOMED |
| 45606671 | Obesity complicating pregnancy, second trimester | 6-char billing code | Condition | ICD10CM |
| 3122236 | Localized adiposity - fat pad | Clinical Finding | Condition | Nebraska Lexicon |
| 45609963 | Body mass index [BMI] 45.0-49.9, adult | 5-char billing code | Observation | ICD10CM |
| 45582462 | Excessive weight gain in pregnancy | ICD10 code | Condition | ICD10 |
| 44825074 | Body Mass Index 50.0-59.9, adult | 5-dig billing V code | Observation | ICD9CM |
| 44811711 | Obese class I (body mass index 30.0 - 34.9) | Clinical Finding | Observation | SNOMED |
| 3478593 | Fatty tissue hyperplasia | Clinical Finding | Condition | Nebraska Lexicon |
| 3434475 | On examination - obese | Clinical Finding | Observation | Nebraska Lexicon |
| 35622038 | Intellectual disability, obesity, brain malformation, facial dysmorphism syndrome | Clinical Finding | Condition | SNOMED |
| 3478613 | Lifelong obesity | Clinical Finding | Condition | Nebraska Lexicon |
| 40565487 | Morbid obesity | Clinical Finding | Condition | SNOMED |
| 36714548 | Wilson Turner syndrome | Clinical Finding | Condition | SNOMED |
| 4005991 | Hyperinsulinar obesity | Clinical Finding | Condition | SNOMED |
| 3078257 | Hypoplasmic obesity | Clinical Finding | Condition | Nebraska Lexicon |
| 4060705 | Body mass index 25-29 - overweight | Clinical Finding | Observation | SNOMED |
| 3439443 | Overweight in adulthood with body mass index of 25 or more but less than 30 | Clinical Finding | Observation | Nebraska Lexicon |
| 36716144 | MEHMO syndrome | Clinical Finding | Condition | SNOMED |
| 45600348 | Body mass index [BMI] 31.0-31.9, adult | 5-char billing code | Observation | ICD10CM |
| 4143290 | Simple obesity NOS | Clinical Finding | Condition | SNOMED |
| 3479268 | Wilson Turner syndrome | Clinical Finding | Condition | Nebraska Lexicon |
| 36716555 | Obesity caused by energy imbalance | Clinical Finding | Condition | SNOMED |
| 44811712 | Obese class II (body mass index 35.0 - 39.9) | Clinical Finding | Observation | SNOMED |
| 40274337 | Buffalo obesity | Clinical Finding | Condition | SNOMED |
| 44830873 | Body Mass Index, pediatric, 85th percentile to less than 95th percentile for age | 5-dig billing V code | Observation | ICD9CM |
| 3479060 | Hyperplastic-hypertrophic obesity | Clinical Finding | Condition | Nebraska Lexicon |
| 3478080 | Severe obesity | Clinical Finding | Condition | Nebraska Lexicon |
| 3068677 | Other hyperalimentation | Clinical Finding | Condition | Nebraska Lexicon |
| 45581058 | Body mass index [BMI] 37.0-37.9, adult | 5-char billing code | Observation | ICD10CM |
| 37204691 | Severe early-onset obesity insulin resistance syndrome due to SH2B1 deficiency | Clinical Finding | Condition | SNOMED |
| 45771307 | Proopiomelanocortin deficiency syndrome | Clinical Finding | Condition | SNOMED |
| 44830215 | Edema or excessive weight gain in pregnancy, without mention of hypertension, postpartum condition or complication | 5-dig billing code | Condition | ICD9CM |
| 4100857 | Extreme obesity with alveolar hypoventilation | Clinical Finding | Condition | SNOMED |
| 44834365 | Body Mass Index 35.0-35.9, adult | 5-dig billing V code | Observation | ICD9CM |
| 36713437 | Choroideremia with deafness and obesity syndrome | Clinical Finding | Condition | SNOMED |
| 36717199 | Obesity, colitis, hypothyroidism, cardiac hypertrophy, developmental delay syndrome | Clinical Finding | Condition | SNOMED |
| 44827941 | Edema or excessive weight gain in pregnancy, without mention of hypertension, delivered, with mention of postpartum complication | 5-dig billing code | Condition | ICD9CM |
| 36714072 | Syndromic X-linked intellectual disability type 7 | Clinical Finding | Condition | SNOMED |
| 4037679 | O/E - obese | Clinical Finding | Observation | SNOMED |
| 3479628 | Obesity due to excess calories | Clinical Finding | Condition | Nebraska Lexicon |
| 4060289 | Edema or excessive weight gain in pregnancy without mention of hypertension with postnatal complication | Clinical Finding | Condition | SNOMED |
| 44830872 | Body Mass Index between 30-39, adult | 4-dig nonbill V code | Observation | ICD9CM |
| 3478016 | Hypertrophic obesity | Clinical Finding | Condition | Nebraska Lexicon |
| 3479731 | Intellectual disability, truncal obesity, retinal dystrophy and micropenis syndrome | Clinical Finding | Condition | Nebraska Lexicon |
| 35207024 | Obesity, unspecified | 4-char billing code | Condition | ICD10CM |
| 45907838 | Hump Behind the Shoulders | Diagnosis | Condition | CIEL |
| 40376430 | Alimentary obesity | Clinical Finding | Condition | SNOMED |
| 3092107 | O/E - overweight (& [weight 10-20% over ideal]) | Clinical Finding | Condition | Nebraska Lexicon |
| 45571383 | Body mass index [BMI] 70 or greater, adult | 5-char billing code | Observation | ICD10CM |
| 40389158 | Hyperalimentation: [Pickwickian syndrome] or [other] | Clinical Finding | Condition | SNOMED |
| 380500 | Hypertrophy of fat pad of knee | Clinical Finding | Condition | SNOMED |
| 3478590 | Hypertrophy of infrapatellar fat pad | Clinical Finding | Condition | Nebraska Lexicon |
| 44821800 | Overweight | 5-dig billing code | Observation | ICD9CM |
| 44825073 | Body Mass Index 28.0-28.9, adult | 5-dig billing V code | Observation | ICD9CM |
| 40272782 | Hypoplasmic obesity | Clinical Finding | Observation | SNOMED |
| 44834569 | Overweight and obesity | 4-dig nonbill code | Observation | ICD9CM |
| 3105167 | Fat pad syndrome | Clinical Finding | Condition | Nebraska Lexicon |
| 40645209 | Lifelong obesity | Clinical Finding | Condition | SNOMED |
| 45600659 | Morbid (severe) obesity due to excess calories | 5-char billing code | Condition | ICD10CM |
| 45533036 | Other obesity | ICD10 code | Condition | ICD10 |
| 44829223 | Hypertrophy of fat pad, knee | 5-dig billing code | Condition | ICD9CM |
| 45910789 | Constitutional Obesity | Diagnosis | Condition | CIEL |
| 45581057 | Body mass index [BMI] 26.0-26.9, adult | 5-char billing code | Observation | ICD10CM |
| 45940321 | Extreme Obesity with Alveolar Hypoventilation | Diagnosis | Condition | CIEL |
| 35207020 | Localized adiposity | 3-char billing code | Condition | ICD10CM |
| 3126800 | Oedema or excessive weight gain in pregnancy without mention of hypertension, delivered with mention of postpartum complication | Clinical Finding | Condition | Nebraska Lexicon |
| 3092108 | O/E weight: [>20% over ideal] or [obese] | Clinical Finding | Condition | Nebraska Lexicon |
| 40321249 | Obesity | Clinical Finding | Observation | SNOMED |
| 3427522 | Patient overweight | Clinical Finding | Observation | Nebraska Lexicon |
| 45581372 | Obesity due to excess calories | ICD10 code | Condition | ICD10 |
| 3476833 | Body mass index 30+ - obesity | Clinical Finding | Observation | Nebraska Lexicon |
| 4189665 | Obesity by fat distribution pattern | Clinical Finding | Condition | SNOMED |
| 3272490 | Choroideraemia co-occurrent with hypopituitarism | Clinical Finding | Condition | Nebraska Lexicon |
| 434005 | Morbid obesity | Clinical Finding | Condition | SNOMED |
| 3478101 | Obesity by adipocyte growth pattern | Clinical Finding | Condition | Nebraska Lexicon |
| 3162741 | Adult-onset obesity | Clinical Finding | Condition | Nebraska Lexicon |
| 45585849 | Body mass index [BMI] 34.0-34.9, adult | 5-char billing code | Observation | ICD10CM |
| 4215969 | Obesity by contributing factors | Clinical Finding | Condition | SNOMED |
| 3199162 | Pregnancy complicated by obesity | Clinical Finding | Condition | Nebraska Lexicon |
| 40389148 | Obesity | Clinical Finding | Condition | SNOMED |
| 44834366 | Body Mass Index 36.0-36.9, adult | 5-dig billing V code | Observation | ICD9CM |
| 4171147 | Hypertrophic obesity | Clinical Finding | Condition | SNOMED |
| 40351293 | (Hyperalimentation including obesity) or (adiposity) | Clinical Finding | Observation | SNOMED |
| 45566452 | Body mass index [BMI] 35.0-35.9, adult | 5-char billing code | Observation | ICD10CM |
| 1576153 | Body mass index [BMI] 30-39, adult | 4-char nonbill code | Observation | ICD10CM |
| 437525 | Overweight | Clinical Finding | Observation | SNOMED |
| 4175214 | O/E - overweight | Clinical Finding | Observation | SNOMED |
| 3480139 | Obesity by fat distribution pattern | Clinical Finding | Condition | Nebraska Lexicon |
| 3479839 | Obesity caused by energy imbalance | Clinical Finding | Condition | Nebraska Lexicon |
| 45917492 | overweight (BMI 25.0-29.9) | Diagnosis | Condition | CIEL |
| 37310479 | Hypertrophy of fat pad of left knee | Clinical Finding | Condition | SNOMED |
| 42872460 | Overweight in childhood | Clinical Finding | Observation | SNOMED |

Table 2. Hypertension disorders (any).

| **Concept ID** | **Concept Name** | **Class** | **Domain** | **Vocabulary** |
| --- | --- | --- | --- | --- |
| 1571639 | Pre-existing hypertensive chronic kidney disease complicating pregnancy, childbirth and the puerperium | 4-char nonbill code | Condition | ICD10CM |
| 44821020 | Mild or unspecified pre-eclampsia | 4-dig nonbill code | Condition | ICD9CM |
| 4311246 | Pre-existing hypertension in obstetric context | Clinical Finding | Condition | SNOMED |
| 3537162 | Unspecified hypertension complicating pregnancy, childbirth and the puerperium - delivered | Clinical Finding | Condition | SNOMED |
| 3126729 | Transient hypertension of pregnancy | Clinical Finding | Condition | Nebraska Lexicon |
| 3255263 | Hypertension complicating pregnancy | Clinical Finding | Condition | Nebraska Lexicon |
| 3126740 | Severe proteinuric hypertension of pregnancy | Clinical Finding | Condition | Nebraska Lexicon |
| 3181572 | Pre-eclampsia or eclampsia with pre-existing hypertension | Clinical Finding | Condition | Nebraska Lexicon |
| 3126727 | Transient hypertension of pregnancy unspecified | Clinical Finding | Condition | Nebraska Lexicon |
| 45940897 | Renal Arterial Hypertension | Diagnosis | Condition | CIEL |
| 4062555 | Unspecified hypertension complicating pregnancy, childbirth and the puerperium NOS | Clinical Finding | Condition | SNOMED |
| 45944917 | Malignant Hypertension Complicating or Reason for Care During Pregnancy | Diagnosis | Condition | CIEL |
| 45572765 | Unspecified maternal hypertension, first trimester | 4-char billing code | Condition | ICD10CM |
| 45757787 | Postpartum pre-existing essential hypertension | Clinical Finding | Condition | SNOMED |
| 45925220 | Severe Preeclampsia | Diagnosis | Condition | CIEL |
| 3531688 | Toxaemia NOS | Clinical Finding | Condition | SNOMED |
| 4062907 | Mild or unspecified pre-eclampsia - delivered | Clinical Finding | Condition | SNOMED |
| 44827931 | Other pre-existing hypertension complicating pregnancy, childbirth, and the puerperium | 4-dig nonbill code | Condition | ICD9CM |
| 45592189 | Pre-eclampsia | ICD10 Hierarchy | Condition | ICD10 |
| 44824391 | Hypertension secondary to renal disease, complicating pregnancy, childbirth, and the puerperium | 4-dig nonbill code | Condition | ICD9CM |
| 4028741 | Benign hypertension | Clinical Finding | Condition | SNOMED |
| 3388867 | Pre-eclampsia or eclampsia with pre-existing hypertension - delivered with postnatal complication | Clinical Finding | Condition | Nebraska Lexicon |
| 4083723 | Essential hypertension complicating AND/OR reason for care during childbirth | Clinical Finding | Condition | SNOMED |
| 3126723 | Other pre-existing hypertension complicating pregnancy, childbirth and the puerperium - not delivered | Clinical Finding | Condition | Nebraska Lexicon |
| 45956666 | Benign Essential Hypertension Complicating Pregnancy, Childbirth and the Puerperium - Not Delivered | Diagnosis | Condition | CIEL |
| 4062425 | Renal hypertension complicating pregnancy, childbirth and the puerperium NOS | Clinical Finding | Condition | SNOMED |
| 45548699 | Pre-existing secondary hypertension complicating pregnancy, unspecified trimester | 6-char billing code | Condition | ICD10CM |
| 45534175 | Mild to moderate pre-eclampsia, second trimester | 5-char billing code | Condition | ICD10CM |
| 36713024 | Labile systemic arterial hypertension | Clinical Finding | Condition | SNOMED |
| 3313565 | Low-renin essential hypertension | Clinical Finding | Condition | Nebraska Lexicon |
| 4062902 | Other pre-existing hypertension complicating pregnancy, childbirth and the puerperium - not delivered | Clinical Finding | Condition | SNOMED |
| 45952005 | HYPERTENSION | Diagnosis | Condition | CIEL |
| 1571656 | Unspecified pre-eclampsia | 4-char nonbill code | Condition | ICD10CM |
| 3295995 | Hypertension in chronic kidney disease stage 4 due to type 2 diabetes mellitus | Clinical Finding | Condition | Nebraska Lexicon |
| 3234093 | Malignant hypertension complicating AND/OR reason for care during puerperium | Clinical Finding | Condition | Nebraska Lexicon |
| 3124278 | Hypertensive disease | Clinical Finding | Condition | Nebraska Lexicon |
| 3191244 | Hypertension with hyperadrenergic findings | Clinical Finding | Condition | Nebraska Lexicon |
| 3656115 | Hypertension due to aortic arch obstruction | Clinical Finding | Condition | SNOMED |
| 45606537 | Unspecified pre-existing hypertension complicating pregnancy, second trimester | 6-char billing code | Condition | ICD10CM |
| 3526658 | Diastolic hypertension | Clinical Finding | Condition | SNOMED |
| 3126752 | Syndrome of haemolysis, elevated liver enzymes and low platelet | Clinical Finding | Condition | Nebraska Lexicon |
| 319826 | Secondary hypertension | Clinical Finding | Condition | SNOMED |
| 3060150 | Accelerated secondary hypertension | Clinical Finding | Condition | Nebraska Lexicon |
| 45954735 | Low-Renin Essential Hypertension | Diagnosis | Condition | CIEL |
| 44827781 | Other benign secondary hypertension | 5-dig billing code | Condition | ICD9CM |
| 45944007 | Renal Hypertension Complicating Pregnancy, Childbirth and the Puerperium - Delivered | Diagnosis | Condition | CIEL |
| 3124307 | Hypertensive disease NOS | Clinical Finding | Condition | Nebraska Lexicon |
| 3126736 | Mild or unspecified pre-eclampsia with postnatal complication | Clinical Finding | Condition | Nebraska Lexicon |
| 45534172 | Pre-existing hypertensive chronic kidney disease complicating childbirth | 5-char billing code | Condition | ICD10CM |
| 3335141 | Diastolic hypertension | Clinical Finding | Condition | Nebraska Lexicon |
| 4034094 | Moderate proteinuric hypertension of pregnancy | Clinical Finding | Condition | SNOMED |
| 40357906 | Non-proteinuric hypertension of pregnancy | Clinical Finding | Condition | SNOMED |
| 44819850 | Hypertension secondary to renal disease, complicating pregnancy, childbirth, and the puerperium, antepartum condition or complication | 5-dig billing code | Condition | ICD9CM |
| 45951903 | Pregnancy, hypertension associated | Diagnosis | Condition | CIEL |
| 4062427 | Mild or unspecified pre-eclampsia unspecified | Clinical Finding | Condition | SNOMED |
| 37200495 | Hypertensive crisis, unspecified | 4-char billing code | Condition | ICD10CM |
| 44837233 | Other pre-existing hypertension complicating pregnancy, childbirth, and the puerperium, unspecified as to episode of care or not applicable | 5-dig billing code | Condition | ICD9CM |
| 40323857 | Secondary hypertension | Clinical Finding | Condition | SNOMED |
| 3312399 | Hypertensive urgency | Clinical Finding | Condition | Nebraska Lexicon |
| 3221809 | Systolic hypertension | Clinical Finding | Condition | Nebraska Lexicon |
| 3267063 | Pre-eclampsia or eclampsia with pre-existing hypertension with postnatal complication | Clinical Finding | Condition | Nebraska Lexicon |
| 44823229 | Benign essential hypertension complicating pregnancy, childbirth, and the puerperium, delivered, with or without mention of antepartum condition | 5-dig billing code | Condition | ICD9CM |
| 3126715 | Benign essential hypertension complicating pregnancy, childbirth and the puerperium unspecified | Clinical Finding | Condition | Nebraska Lexicon |
| 3375648 | Hypertension secondary to renal disease complicating AND/OR reason for care during pregnancy | Clinical Finding | Condition | Nebraska Lexicon |
| 3220548 | Hypertension due to renovascular disease | Clinical Finding | Condition | Nebraska Lexicon |
| 3424780 | Benign essential hypertension | Clinical Finding | Condition | Nebraska Lexicon |
| 3414459 | Benign essential hypertension complicating pregnancy, childbirth and the puerperium - delivered with postnatal complication | Clinical Finding | Condition | Nebraska Lexicon |
| 3126755 | Unspecified hypertension complicating pregnancy, childbirth and the puerperium unspecified | Clinical Finding | Condition | Nebraska Lexicon |
| 4057975 | Mild or unspecified pre-eclampsia - not delivered | Clinical Finding | Condition | SNOMED |
| 40484842 | Benign secondary hypertension | Clinical Finding | Condition | SNOMED |
| 45925579 | Chronic Hypertension Complicating and/or Reason for Care During Puerperium | Diagnosis | Condition | CIEL |
| 45587283 | Severe pre-eclampsia | ICD10 code | Condition | ICD10 |
| 44834877 | Mild or unspecified pre-eclampsia, antepartum condition or complication | 5-dig billing code | Condition | ICD9CM |
| 3126732 | Mild or unspecified pre-eclampsia unspecified | Clinical Finding | Condition | Nebraska Lexicon |
| 45953779 | Pre-existing hypertension in obstetric context | Diagnosis | Condition | CIEL |
| 45572764 | Pre-eclampsia, unspecified | ICD10 code | Condition | ICD10 |
| 3106386 | Pre-eclampsia NOS | Clinical Finding | Condition | Nebraska Lexicon |
| 45908029 | Transient Hypertension of Pregnancy with Postnatal Complication | Diagnosis | Condition | CIEL |
| 3124302 | Secondary benign hypertension NOS | Clinical Finding | Condition | Nebraska Lexicon |
| 3107237 | Maternal hypertension | Clinical Finding | Condition | Nebraska Lexicon |
| 3333074 | Hypertension with albuminuria | Clinical Finding | Condition | Nebraska Lexicon |
| 45539086 | Pre-existing hypertensive chronic kidney disease complicating the puerperium | 5-char billing code | Condition | ICD10CM |
| 3352438 | Hypertensive emergency | Clinical Finding | Condition | Nebraska Lexicon |
| 3246076 | Transient hypertension of pregnancy - not delivered | Clinical Finding | Condition | Nebraska Lexicon |
| 45908382 | Renal Hypertension | Diagnosis | Condition | CIEL |
| 3404956 | Parenchymal renal hypertension | Clinical Finding | Condition | Nebraska Lexicon |
| 45915529 | White Coat Hypertension | Diagnosis | Condition | CIEL |
| 45944847 | Benign Essential Hypertension Complicating or Reason for Care During Childbirth | Diagnosis | Condition | CIEL |
| 200157 | Renal hypertension complicating pregnancy, childbirth and the puerperium - delivered | Clinical Finding | Condition | SNOMED |
| 3535482 | Other pre-existing hypertension complicating pregnancy, childbirth and the puerperium - not delivered | Clinical Finding | Condition | SNOMED |
| 45932564 | Essential hypertension | Diagnosis | Condition | CIEL |
| 3126720 | Other pre-existing hypertension complicating pregnancy, childbirth and the puerperium unspecified | Clinical Finding | Condition | Nebraska Lexicon |
| 3070805 | Accelerated essential hypertension | Clinical Finding | Condition | Nebraska Lexicon |
| 37200973 | Unspecified pre-eclampsia, complicating childbirth | 5-char billing code | Condition | ICD10CM |
| 40530211 | Hypertension in the obstetric context | Clinical Finding | Condition | SNOMED |
| 4243055 | Endocrine hypertension | Clinical Finding | Condition | SNOMED |
| 3126721 | Other pre-existing hypertension complicating pregnancy, childbirth and the puerperium - delivered | Clinical Finding | Condition | Nebraska Lexicon |
| 45939852 | Goldblatt Hypertension | Diagnosis | Condition | CIEL |
| 3383916 | Secondary hypertension | Clinical Finding | Condition | Nebraska Lexicon |
| 3263327 | Pre-existing hypertensive chronic kidney disease in mother complicating pregnancy | Clinical Finding | Condition | Nebraska Lexicon |
| 45911606 | unspecified maternal hypertension, postpartum condition or complication | Diagnosis | Condition | CIEL |
| 3229356 | Pre-existing hypertension, NOS, complicating or reason for care during puerperium | Clinical Finding | Condition | Nebraska Lexicon |
| 1571636 | Pre-existing essential hypertension complicating pregnancy, | 5-char nonbill code | Condition | ICD10CM |
| 3126758 | Unspecified hypertension complicating pregnancy, childbirth and the puerperium - not delivered | Clinical Finding | Condition | Nebraska Lexicon |
| 45936108 | Severe Pre-Eclampsia - Not Delivered | Diagnosis | Condition | CIEL |
| 40557800 | White coat hypertension | Clinical Finding | Condition | SNOMED |
| 44819852 | Severe pre-eclampsia, antepartum condition or complication | 5-dig billing code | Condition | ICD9CM |
| 45925432 | Pregnancy-induced hypertension | Diagnosis | Condition | CIEL |
| 316866 | Hypertensive disorder | Clinical Finding | Condition | SNOMED |
| 3369067 | Transient hypertension of pregnancy - delivered with postnatal complication | Clinical Finding | Condition | Nebraska Lexicon |
| 4057978 | Pre-existing hypertensive heart and renal disease complicating pregnancy, childbirth and the puerperium | Clinical Finding | Condition | SNOMED |
| 44827933 | Severe pre-eclampsia, unspecified as to episode of care or not applicable | 5-dig billing code | Condition | ICD9CM |
| 40394355 | Moderate proteinuric hypertension of pregnancy | Clinical Finding | Condition | SNOMED |
| 37200976 | Unspecified maternal hypertension, complicating the puerperium | 4-char billing code | Condition | ICD10CM |
| 45926873 | Secondary hypertension | Diagnosis | Condition | CIEL |
| 314423 | Benign essential hypertension complicating pregnancy, childbirth and the puerperium - not delivered | Clinical Finding | Condition | SNOMED |
| 44819851 | Mild or unspecified pre-eclampsia, unspecified as to episode of care or not applicable | 5-dig billing code | Condition | ICD9CM |
| 4151903 | Pre-existing hypertension complicating AND/OR reason for care during puerperium | Clinical Finding | Condition | SNOMED |
| 40562800 | White coat hypertension | Clinical Finding | Condition | SNOMED |
| 3285129 | Postoperative hypertension | Clinical Finding | Condition | Nebraska Lexicon |
| 37200967 | Mild to moderate pre-eclampsia, complicating childbirth | 5-char billing code | Condition | ICD10CM |
| 3124280 | Benign essential hypertension | Clinical Finding | Condition | Nebraska Lexicon |
| 44837234 | Mild or unspecified pre-eclampsia, delivered, with mention of postpartum complication | 5-dig billing code | Condition | ICD9CM |
| 439077 | Severe pre-eclampsia - delivered with postnatal complication | Clinical Finding | Condition | SNOMED |
| 3162040 | Proteinuric hypertension of pregnancy | Clinical Finding | Condition | Nebraska Lexicon |
| 45601778 | Pre-existing essential hypertension complicating pregnancy, second trimester | 6-char billing code | Condition | ICD10CM |
| 135601 | Pre-eclampsia or eclampsia with pre-existing hypertension - delivered | Clinical Finding | Condition | SNOMED |
| 45954468 | Non-Proteinuric Hypertension of Pregnancy | Diagnosis | Condition | CIEL |
| 3105645 | Hypertensive disease | Clinical Finding | Condition | Nebraska Lexicon |
| 1571647 | Pre-existing hypertension with pre-eclampsia | 3-char nonbill code | Condition | ICD10CM |
| 3303273 | High-renin essential hypertension | Clinical Finding | Condition | Nebraska Lexicon |
| 40603936 | Accelerated secondary hypertension | Clinical Finding | Condition | SNOMED |
| 45768449 | Hypertensive crisis | Clinical Finding | Condition | SNOMED |
| 4108666 | Secondary benign hypertension NOS | Clinical Finding | Condition | SNOMED |
| 45937104 | Pre-Existing Hypertension Complicating Pregnancy, Childbirth and Puerperium | Diagnosis | Condition | CIEL |
| 4253928 | Secondary diastolic hypertension | Clinical Finding | Condition | SNOMED |
| 45920724 | Chronic Hypertension in Obstetric Context | Diagnosis | Condition | CIEL |
| 3227775 | Chronic hypertension complicating AND/OR reason for care during puerperium | Clinical Finding | Condition | Nebraska Lexicon |
| 321080 | Hypertension complicating pregnancy, childbirth and the puerperium | Clinical Finding | Condition | SNOMED |
| 4215640 | Benign essential hypertension complicating AND/OR reason for care during childbirth | Clinical Finding | Condition | SNOMED |
| 45543913 | Pre-existing essential hypertension complicating pregnancy, third trimester | 6-char billing code | Condition | ICD10CM |
| 3335232 | Severe pre-eclampsia - delivered | Clinical Finding | Condition | Nebraska Lexicon |
| 4179379 | Exertional hypertension | Clinical Finding | Condition | SNOMED |
| 45539091 | Gestational [pregnancy-induced] hypertension | ICD10 Hierarchy | Condition | ICD10 |
| 35624277 | Hypertension due to gain-of-function mutation in mineralocorticoid receptor | Clinical Finding | Condition | SNOMED |
| 320456 | Benign essential hypertension complicating pregnancy, childbirth and the puerperium - delivered with postnatal complication | Clinical Finding | Condition | SNOMED |
| 45548707 | Unspecified maternal hypertension, third trimester | 4-char billing code | Condition | ICD10CM |
| 45596189 | Hypertension secondary to endocrine disorders | ICD10 code | Condition | ICD10 |
| 3106388 | Hypertension of pregnancy NOS | Clinical Finding | Condition | Nebraska Lexicon |
| 44832371 | Benign renovascular hypertension | 5-dig billing code | Condition | ICD9CM |
| 4058540 | Unspecified hypertension complicating pregnancy, childbirth and the puerperium unspecified | Clinical Finding | Condition | SNOMED |
| 4034031 | Benign essential hypertension complicating AND/OR reason for care during pregnancy | Clinical Finding | Condition | SNOMED |
| 3536734 | Unspecified hypertension complicating pregnancy, childbirth and the puerperium - not delivered | Clinical Finding | Condition | SNOMED |
| 3126717 | Renal hypertension complicating pregnancy, childbirth and the puerperium unspecified | Clinical Finding | Condition | Nebraska Lexicon |
| 37200975 | Unspecified maternal hypertension, complicating childbirth | 4-char billing code | Condition | ICD10CM |
| 45936107 | Severe Pre-Eclampsia with Postnatal Complication | Diagnosis | Condition | CIEL |
| 35207676 | Hypertension secondary to other renal disorders | 4-char billing code | Condition | ICD10CM |
| 45567163 | Secondary hypertension, unspecified | ICD10 code | Condition | ICD10 |
| 4111388 | Hypertensive disease NOS | Clinical Finding | Condition | SNOMED |
| 3126750 | Pre-eclampsia or eclampsia with pre-existing hypertension NOS | Clinical Finding | Condition | Nebraska Lexicon |
| 3126718 | Renal hypertension complicating pregnancy, childbirth and the puerperium NOS | Clinical Finding | Condition | Nebraska Lexicon |
| 443771 | Renal hypertension | Clinical Finding | Condition | SNOMED |
| 4023318 | Malignant hypertension complicating AND/OR reason for care during childbirth | Clinical Finding | Condition | SNOMED |
| 3533788 | Unspecified hypertension complicating pregnancy, childbirth and the puerperium NOS | Clinical Finding | Condition | SNOMED |
| 45591453 | Essential (primary) hypertension | ICD10 Hierarchy | Condition | ICD10 |
| 3148950 | Hypertension in the obstetric context | Clinical Finding | Condition | Nebraska Lexicon |
| 4062429 | Mild or unspecified pre-eclampsia NOS | Clinical Finding | Condition | SNOMED |
| 45928814 | Essential Hypertension Complicating or Reason for Care During Childbirth | Diagnosis | Condition | CIEL |
| 45939243 | Pre-Existing Hypertension, Complicating or Reason for Care During Puerperium | Diagnosis | Condition | CIEL |
| 4316372 | HELLP syndrome | Clinical Finding | Condition | SNOMED |
| 4032952 | Renal sclerosis with hypertension | Clinical Finding | Condition | SNOMED |
| 3106383 | Hypertension of pregnancy | Clinical Finding | Condition | Nebraska Lexicon |
| 45925431 | Hypertension Complicating Pregnancy, Childbirth, or the Puerperium | Diagnosis | Condition | CIEL |
| 136760 | Transient hypertension of pregnancy - not delivered | Clinical Finding | Condition | SNOMED |
| 40398393 | Benign essential hypertension | Clinical Finding | Condition | SNOMED |
| 3142057 | Mild or unspecified pre-eclampsia | Clinical Finding | Condition | Nebraska Lexicon |
| 3271356 | Labile systemic arterial hypertension | Clinical Finding | Condition | Nebraska Lexicon |
| 3344507 | Toxaemia of pregnancy | Clinical Finding | Condition | Nebraska Lexicon |
| 3126735 | Mild or unspecified pre-eclampsia - not delivered | Clinical Finding | Condition | Nebraska Lexicon |
| 45943813 | Severe Pre-Eclampsia - Delivered with Postnatal Complication | Diagnosis | Condition | CIEL |
| 3337784 | Chronic hypertension complicating AND/OR reason for care during childbirth | Clinical Finding | Condition | Nebraska Lexicon |
| 4048212 | Goldblatt hypertension | Clinical Finding | Condition | SNOMED |
| 4058530 | Other pre-existing hypertension in preg/childbirth/puerp | Clinical Finding | Condition | SNOMED |
| 4062909 | Unspecified hypertension complicating pregnancy, childbirth and the puerperium - delivered with postnatal complication | Clinical Finding | Condition | SNOMED |
| 3340018 | Pre-existing hypertensive heart and renal disease complicating pregnancy, childbirth and the puerperium | Clinical Finding | Condition | Nebraska Lexicon |
| 45757119 | Eclampsia with pre-existing hypertension in childbirth | Clinical Finding | Condition | SNOMED |
| 3181911 | Pre-eclampsia added to pre-existing hypertension | Clinical Finding | Condition | Nebraska Lexicon |
| 44811933 | Stage 1 hypertension (NICE 2011) with evidence of end organ damage | Clinical Finding | Condition | SNOMED |
| 37200968 | Mild to moderate pre-eclampsia, complicating the puerperium | 5-char billing code | Condition | ICD10CM |
| 4035655 | Toxemia of pregnancy | Clinical Finding | Condition | SNOMED |
| 45939246 | Pre-Eclampsia or Eclampsia with Pre-Existing Hypertension with Postnatal Complication | Diagnosis | Condition | CIEL |
| 45937233 | Malignant Secondary Renovascular Hypertension | Diagnosis | Condition | CIEL |
| 3179887 | Severe pre-eclampsia - not delivered | Clinical Finding | Condition | Nebraska Lexicon |
| 45950724 | Diastolic Hypertension | Diagnosis | Condition | CIEL |
| 44827930 | Hypertension secondary to renal disease, complicating pregnancy, childbirth, and the puerperium, delivered, with or without mention of antepartum condition | 5-dig billing code | Condition | ICD9CM |
| 3124305 | Secondary hypertension NOS | Clinical Finding | Condition | Nebraska Lexicon |
| 4219323 | Hypertension secondary to renal disease complicating AND/OR reason for care during puerperium | Clinical Finding | Condition | SNOMED |
| 3328019 | Eclampsia with pre-existing hypertension in labour and delivery | Clinical Finding | Condition | Nebraska Lexicon |
| 3271004 | Benign essential hypertension complicating AND/OR reason for care during puerperium | Clinical Finding | Condition | Nebraska Lexicon |
| 45916604 | Benign Secondary Hypertension | Diagnosis | Condition | CIEL |
| 3302215 | Impending eclampsia | Clinical Finding | Condition | Nebraska Lexicon |
| 3332857 | Renal arterial hypertension | Clinical Finding | Condition | Nebraska Lexicon |
| 45921944 | pre-existing hypertensive heart and renal disease complicating pregnancy, childbirth and the puerperium, unspecified as to episode of care, or not applicable | Diagnosis | Condition | CIEL |
| 44811932 | Stage 1 hypertension (NICE 2011) without evidence of end organ damage | Clinical Finding | Condition | SNOMED |
| 45534171 | Pre-existing hypertensive chronic kidney disease complicating pregnancy, unspecified trimester | 6-char billing code | Condition | ICD10CM |
| 1571643 | Pre-existing secondary hypertension complicating pregnancy, childbirth and the puerperium | 4-char nonbill code | Condition | ICD10CM |
| 44827929 | Benign essential hypertension, complicating pregnancy, childbirth, and the puerperium, delivered, with mention of postpartum complication | 5-dig billing code | Condition | ICD9CM |
| 35207677 | Hypertension secondary to endocrine disorders | 4-char billing code | Condition | ICD10CM |
| 3126734 | Mild or unspecified pre-eclampsia - delivered with postnatal complication | Clinical Finding | Condition | Nebraska Lexicon |
| 45577559 | Pre-existing hypertension with pre-eclampsia, third trimester | 4-char billing code | Condition | ICD10CM |
| 3124304 | Secondary renovascular hypertension NOS | Clinical Finding | Condition | Nebraska Lexicon |
| 3214529 | Hypertension secondary to drug | Clinical Finding | Condition | Nebraska Lexicon |
| 3317061 | Pre-eclampsia or eclampsia with pre-existing hypertension - delivered | Clinical Finding | Condition | Nebraska Lexicon |
| 4062905 | Other pre-existing hypertension complicating pregnancy, childbirth and the puerperium NOS | Clinical Finding | Condition | SNOMED |
| 4034095 | Impending eclampsia | Clinical Finding | Condition | SNOMED |
| 4062428 | Mild or unspecified pre-eclampsia with postnatal complication | Clinical Finding | Condition | SNOMED |
| 321638 | Benign essential hypertension complicating pregnancy, childbirth and the puerperium | Clinical Finding | Condition | SNOMED |
| 44823230 | Pre-eclampsia or eclampsia superimposed on pre-existing hypertension, delivered, with mention of postpartum complication | 5-dig billing code | Condition | ICD9CM |
| 3126726 | Transient hypertension of pregnancy | Clinical Finding | Condition | Nebraska Lexicon |
| 3315379 | Antepartum hypertensive vascular disease | Clinical Finding | Condition | Nebraska Lexicon |
| 45601777 | Pre-existing essential hypertension complicating pregnancy, childbirth and the puerperium | ICD10 code | Condition | ICD10 |
| 1571646 | Unspecified pre-existing hypertension complicating pregnancy | 5-char nonbill code | Condition | ICD10CM |
| 45757356 | Pre-existing hypertensive chronic kidney disease in mother complicating pregnancy | Clinical Finding | Condition | SNOMED |
| 45948451 | Benign Essential Hypertension | Diagnosis | Condition | CIEL |
| 198215 | Renal hypertension complicating pregnancy, childbirth and the puerperium with postnatal complication | Clinical Finding | Condition | SNOMED |
| 45771064 | Hypertension in chronic kidney disease due to type 2 diabetes mellitus | Clinical Finding | Condition | SNOMED |
| 44824392 | Hypertension secondary to renal disease, complicating pregnancy, childbirth, and the puerperium, delivered, with mention of postpartum complication | 5-dig billing code | Condition | ICD9CM |
| 40282397 | Accelerated essential hypertension | Clinical Finding | Condition | SNOMED |
| 44823231 | Unspecified hypertension complicating pregnancy, childbirth, or the puerperium | 4-dig nonbill code | Condition | ICD9CM |
| 45913987 | Transient Hypertension of Pregnancy | Diagnosis | Condition | CIEL |
| 45931155 | Hypertensive Disorder | Diagnosis | Condition | CIEL |
| 3542241 | Pre-eclampsia NOS | Clinical Finding | Condition | SNOMED |
| 4178312 | Hypertension secondary to kidney transplant | Clinical Finding | Condition | SNOMED |
| 44800519 | [X]Hypertensive diseases | Clinical Finding | Condition | SNOMED |
| 37200974 | Unspecified pre-eclampsia, complicating the puerperium | 5-char billing code | Condition | ICD10CM |
| 3267115 | Perioperative hypertension | Clinical Finding | Condition | Nebraska Lexicon |
| 4058532 | Other pre-existing hypertension complicating pregnancy, childbirth and the puerperium - delivered with postnatal complication | Clinical Finding | Condition | SNOMED |
| 3187664 | Hypertension complicating pregnancy, childbirth and the puerperium, antepartum | Clinical Finding | Condition | Nebraska Lexicon |
| 45606538 | Mild to moderate pre-eclampsia, unspecified trimester | 5-char billing code | Condition | ICD10CM |
| 45931624 | Pre-Eclampsia or Eclampsia with Pre-Existing Hypertension - not Delivered | Diagnosis | Condition | CIEL |
| 3126737 | Mild pre-eclampsia | Clinical Finding | Condition | Nebraska Lexicon |
| 3254994 | Endocrine hypertension | Clinical Finding | Condition | Nebraska Lexicon |
| 45908030 | Transient Hypertension of Pregnancy - Delivered | Diagnosis | Condition | CIEL |
| 45924334 | Renal Hypertension Complicating Pregnancy, Childbirth and the Puerperium - Delivered with Postnatal Complication | Diagnosis | Condition | CIEL |
| 45757447 | Hypertension in chronic kidney disease stage 2 due to type 2 diabetes mellitus | Clinical Finding | Condition | SNOMED |
| 35622939 | Postpartum pre-eclampsia | Clinical Finding | Condition | SNOMED |
| 40505836 | Transient hypertension of pregnancy | Clinical Finding | Condition | SNOMED |
| 3520701 | Mild or unspecified pre-eclampsia - delivered with postnatal complication | Clinical Finding | Condition | SNOMED |
| 1569124 | Secondary hypertension | 3-char nonbill code | Condition | ICD10CM |
| 45596923 | Unspecified maternal hypertension, unspecified trimester | 4-char billing code | Condition | ICD10CM |
| 35207679 | Secondary hypertension, unspecified | 4-char billing code | Condition | ICD10CM |
| 45567887 | Unspecified pre-existing hypertension complicating the puerperium | 5-char billing code | Condition | ICD10CM |
| 4110949 | Secondary hypertension NOS | Clinical Finding | Condition | SNOMED |
| 3345956 | Intermittent hypertension | Clinical Finding | Condition | Nebraska Lexicon |
| 4167358 | Diastolic hypertension | Clinical Finding | Condition | SNOMED |
| 44822083 | Unspecified hypertension complicating pregnancy, childbirth, or the puerperium, delivered, with mention of postpartum complication | 5-dig billing code | Condition | ICD9CM |
| 44784484 | Hypertension in the puerperium with pulmonary edema | Clinical Finding | Condition | SNOMED |
| 40323861 | Hypertensive disease NOS | Clinical Finding | Condition | SNOMED |
| 45915665 | Hypertension Secondary to Renal Disease Complicating or Reason for Care During Puerperium | Diagnosis | Condition | CIEL |
| 45539088 | Pre-existing secondary hypertension complicating pregnancy, childbirth and the puerperium | ICD10 code | Condition | ICD10 |
| 3126731 | Mild or unspecified pre-eclampsia | Clinical Finding | Condition | Nebraska Lexicon |
| 3423686 | Pregnancy induced hypertension with pulmonary oedema | Clinical Finding | Condition | Nebraska Lexicon |
| 4110948 | Hypertension secondary to endocrine disorder | Clinical Finding | Condition | SNOMED |
| 4057973 | Transient hypertension of pregnancy unspecified | Clinical Finding | Condition | SNOMED |
| 45950160 | Chronic Hypertension Complicating and/or Reason for Care During Pregnancy | Diagnosis | Condition | CIEL |
| 3415347 | Hypertension in chronic kidney disease due to type 1 diabetes mellitus | Clinical Finding | Condition | Nebraska Lexicon |
| 314103 | Benign essential hypertension complicating pregnancy, childbirth and the puerperium - delivered | Clinical Finding | Condition | SNOMED |
| 45539093 | Severe pre-eclampsia, unspecified trimester | 5-char billing code | Condition | ICD10CM |
| 4199306 | Transient hypertension | Clinical Finding | Condition | SNOMED |
| 3067182 | Severe proteinuric hypertension of pregnancy | Clinical Finding | Condition | Nebraska Lexicon |
| 45911929 | pre-existing secondary hypertension complicating pregnancy, childbirth and the puerperium | Diagnosis | Condition | CIEL |
| 3126724 | Other pre-existing hypertension complicating pregnancy, childbirth and the puerperium with postnatal complication | Clinical Finding | Condition | Nebraska Lexicon |
| 35210518 | Neonatal hypertension | 4-char billing code | Condition | ICD10CM |
| 3377964 | Transient hypertension of pregnancy | Clinical Finding | Condition | Nebraska Lexicon |
| 44831233 | Malignant secondary hypertension | 4-dig nonbill code | Condition | ICD9CM |
| 4058987 | High-renin essential hypertension | Clinical Finding | Condition | SNOMED |
| 44836077 | Transient hypertension of pregnancy, delivered , with or without mention of antepartum condition | 5-dig billing code | Condition | ICD9CM |
| 45582442 | Pre-existing essential hypertension complicating pregnancy, first trimester | 6-char billing code | Condition | ICD10CM |
| 312648 | Benign essential hypertension | Clinical Finding | Condition | SNOMED |
| 441922 | Transient hypertension of pregnancy | Clinical Finding | Condition | SNOMED |
| 4058088 | Unspecified hypertension complicating pregnancy, childbirth and the puerperium | Clinical Finding | Condition | SNOMED |
| 3126739 | Mild or unspecified pre-eclampsia NOS | Clinical Finding | Condition | Nebraska Lexicon |
| 40393963 | Hypertension of pregnancy (& [gestational] or [transient]) | Clinical Finding | Condition | SNOMED |
| 3362089 | Systolic essential hypertension | Clinical Finding | Condition | Nebraska Lexicon |
| 45563052 | Gestational [pregnancy-induced] hypertension without significant proteinuria, third trimester | 4-char billing code | Condition | ICD10CM |
| 3396586 | Goldblatt hypertension | Clinical Finding | Condition | Nebraska Lexicon |
| 4277110 | Pre-existing hypertension complicating AND/OR reason for care during pregnancy | Clinical Finding | Condition | SNOMED |
| 44834715 | Benign essential hypertension | 4-dig billing code | Condition | ICD9CM |
| 42619326 | HELLP syndrome | ICD10 code | Condition | ICD10 |
| 1571652 | Gestational [pregnancy-induced] hypertension without significant proteinuria | 3-char nonbill code | Condition | ICD10CM |
| 44825592 | Unspecified hypertension complicating pregnancy, childbirth, or the puerperium, postpartum condition or complication | 5-dig billing code | Condition | ICD9CM |
| 45939247 | Pre-Eclampsia Added to Pre-Existing Hypertension | Diagnosis | Condition | CIEL |
| 35207668 | Essential (primary) hypertension | 3-char billing code | Condition | ICD10CM |
| 3327142 | Pre-existing hypertensive heart and chronic kidney disease in mother complicating childbirth | Clinical Finding | Condition | Nebraska Lexicon |
| 45548705 | Severe pre-eclampsia, second trimester | 5-char billing code | Condition | ICD10CM |
| 45944169 | Pre-Eclampsia or Eclampsia with Pre-Existing Hypertension | Diagnosis | Condition | CIEL |
| 1571659 | Unspecified maternal hypertension | 3-char nonbill code | Condition | ICD10CM |
| 45548008 | Other secondary hypertension | ICD10 code | Condition | ICD10 |
| 4148205 | Benign essential hypertension complicating AND/OR reason for care during puerperium | Clinical Finding | Condition | SNOMED |
| 4058533 | Transient hypertension of pregnancy NOS | Clinical Finding | Condition | SNOMED |
| 44831235 | Benign secondary hypertension | 4-dig nonbill code | Condition | ICD9CM |
| 40358544 | Hypertension of pregnancy (& [pre-eclampsia]) | Clinical Finding | Condition | SNOMED |
| 43021830 | Postoperative hypertension | Clinical Finding | Condition | SNOMED |
| 3546634 | Stage 1 hypertension (NICE - National Institute for Health and Clinical Excellence 2011) without evidence of end organ damage | Clinical Finding | Condition | SNOMED |
| 4159755 | Labile essential hypertension | Clinical Finding | Condition | SNOMED |
| 3226189 | Essential hypertension complicating AND/OR reason for care during childbirth | Clinical Finding | Condition | Nebraska Lexicon |
| 45933831 | pre-existing essential hypertension complicating pregnancy, childbirth and the puerperium | Diagnosis | Condition | CIEL |
| 4263067 | Low-renin essential hypertension | Clinical Finding | Condition | SNOMED |
| 4283352 | Pre-eclampsia added to pre-existing hypertension | Clinical Finding | Condition | SNOMED |
| 3373357 | Renal hypertension | Clinical Finding | Condition | Nebraska Lexicon |
| 44832523 | Pre-eclampsia or eclampsia superimposed on pre-existing hypertension, delivered, with or without mention of antepartum condition | 5-dig billing code | Condition | ICD9CM |
| 4205410 | Gestational hypertension | Clinical Finding | Condition | SNOMED |
| 44809027 | Severe hypertension (NICE - National Institute for Health and Clinical Excellence 2011) | Clinical Finding | Condition | SNOMED |
| 4217486 | Essential hypertension in obstetric context | Clinical Finding | Condition | SNOMED |
| 3545642 | Stage 1 hypertension | Clinical Finding | Condition | SNOMED |
| 45910319 | Chronic Hypertension Complicating and/or Reason for Care During Childbirth | Diagnosis | Condition | CIEL |
| 4174979 | Hypertension secondary to renal disease complicating AND/OR reason for care during pregnancy | Clinical Finding | Condition | SNOMED |
| 4162306 | Malignant hypertension complicating AND/OR reason for care during pregnancy | Clinical Finding | Condition | SNOMED |
| 3189936 | Hypertension in the obstetric context | Clinical Finding | Condition | Nebraska Lexicon |
| 3314897 | Pre-existing secondary hypertension complicating pregnancy, childbirth and puerperium | Clinical Finding | Condition | Nebraska Lexicon |
| 44824390 | Benign essential hypertension complicating pregnancy, childbirth, and the puerperium, antepartum condition or complication | 5-dig billing code | Condition | ICD9CM |
| 40429408 | Malignant secondary renovascular hypertension | Clinical Finding | Condition | SNOMED |
| 44833715 | Transient hypertension of pregnancy, antepartum condition or complication | 5-dig billing code | Condition | ICD9CM |
| 44833714 | Hypertension secondary to renal disease, complicating pregnancy, childbirth, and the puerperium, postpartum condition or complication | 5-dig billing code | Condition | ICD9CM |
| 44809026 | Stage 1 hypertension (NICE - National Institute for Health and Clinical Excellence 2011) | Clinical Finding | Condition | SNOMED |
| 317895 | Renovascular hypertension | Clinical Finding | Condition | SNOMED |
| 40318658 | Pre-eclampsia NOS | Clinical Finding | Condition | SNOMED |
| 45557535 | Hypertension secondary to other renal disorders | ICD10 code | Condition | ICD10 |
| 45936145 | Secondary Diastolic Hypertension | Diagnosis | Condition | CIEL |
| 44822084 | Unspecified hypertension complicating pregnancy, childbirth, or the puerperium, antepartum condition or complication | 5-dig billing code | Condition | ICD9CM |
| 4209293 | Systolic hypertension | Clinical Finding | Condition | SNOMED |
| 4143966 | Essential hypertension NOS | Clinical Finding | Condition | SNOMED |
| 45935957 | moderate pre-eclampsia | Diagnosis | Condition | CIEL |
| 3342296 | Moderate proteinuric hypertension of pregnancy | Clinical Finding | Condition | Nebraska Lexicon |
| 3106382 | Pregnancy with hypertension | Clinical Finding | Condition | Nebraska Lexicon |
| 4062908 | Unspecified hypertension complicating pregnancy, childbirth and the puerperium - delivered | Clinical Finding | Condition | SNOMED |
| 4262182 | Hypertension with albuminuria | Clinical Finding | Condition | SNOMED |
| 40394319 | Mild pre-eclampsia | Clinical Finding | Condition | SNOMED |
| 3381404 | Hypertension in chronic kidney disease stage 3 due to type 2 diabetes mellitus | Clinical Finding | Condition | Nebraska Lexicon |
| 192684 | Renal hypertension complicating pregnancy, childbirth and the puerperium - not delivered | Clinical Finding | Condition | SNOMED |
| 45592185 | Pre-existing secondary hypertension complicating pregnancy, third trimester | 6-char billing code | Condition | ICD10CM |
| 37016726 | Supine hypertension | Clinical Finding | Condition | SNOMED |
| 321074 | Pre-existing hypertension complicating pregnancy, childbirth and puerperium | Clinical Finding | Condition | SNOMED |
| 45534176 | Unspecified pre-eclampsia, second trimester | 5-char billing code | Condition | ICD10CM |
| 45953181 | Pre-Eclampsia or Eclampsia with Pre-Existing Hypertension - Delivered | Diagnosis | Condition | CIEL |
| 45582444 | Pre-existing hypertensive heart and chronic kidney disease complicating the puerperium | 5-char billing code | Condition | ICD10CM |
| 45587284 | Severe pre-eclampsia, third trimester | 5-char billing code | Condition | ICD10CM |
| 44821019 | Transient hypertension of pregnancy, postpartum condition or complication | 5-dig billing code | Condition | ICD9CM |
| 45572762 | Pre-eclampsia superimposed on chronic hypertension | ICD10 Hierarchy | Condition | ICD10 |
| 40481896 | Hypertensive urgency | Clinical Finding | Condition | SNOMED |
| 3314185 | Labile hypertension due to being in a clinical environment | Clinical Finding | Condition | Nebraska Lexicon |
| 3230775 | Brachydactyly and arterial hypertension syndrome | Clinical Finding | Condition | Nebraska Lexicon |
| 4305599 | Renal arterial hypertension | Clinical Finding | Condition | SNOMED |
| 45539092 | Mild to moderate pre-eclampsia, third trimester | 5-char billing code | Condition | ICD10CM |
| 3536931 | Other pre-existing hypertension complicating pregnancy, childbirth and the puerperium NOS | Clinical Finding | Condition | SNOMED |
| 3142114 | Hypertension of pregnancy NOS | Clinical Finding | Condition | Nebraska Lexicon |
| 36715087 | Brachydactyly and arterial hypertension syndrome | Clinical Finding | Condition | SNOMED |
| 45946108 | High-Renin Essential Hypertension | Diagnosis | Condition | CIEL |
| 4321603 | Essential hypertension complicating AND/OR reason for care during puerperium | Clinical Finding | Condition | SNOMED |
| 3520700 | Mild or unspecified pre-eclampsia unspecified | Clinical Finding | Condition | SNOMED |
| 3367157 | Pre-eclampsia or eclampsia with pre-existing hypertension - not delivered | Clinical Finding | Condition | Nebraska Lexicon |
| 3390946 | Non-proteinuric hypertension of pregnancy | Clinical Finding | Condition | Nebraska Lexicon |
| 44825591 | Pre-eclampsia or eclampsia superimposed on pre-existing hypertension, antepartum condition or complication | 5-dig billing code | Condition | ICD9CM |
| 3106385 | Mild pre-eclampsia | Clinical Finding | Condition | Nebraska Lexicon |
| 3105651 | Malignant hypertension | Clinical Finding | Condition | Nebraska Lexicon |
| 44824393 | Severe pre-eclampsia, postpartum condition or complication | 5-dig billing code | Condition | ICD9CM |
| 3356866 | Transient hypertension of pregnancy with postnatal complication | Clinical Finding | Condition | Nebraska Lexicon |
| 4322735 | Hypertension associated with transplantation | Clinical Finding | Condition | SNOMED |
| 45567886 | Pre-existing hypertensive chronic kidney disease complicating pregnancy, first trimester | 6-char billing code | Condition | ICD10CM |
| 3141573 | Hypertensive disease | Clinical Finding | Condition | Nebraska Lexicon |
| 3563470 | Secondary hypertension NOS | Clinical Finding | Condition | SNOMED |
| 44826775 | Mild or unspecified pre-eclampsia, postpartum condition or complication | 5-dig billing code | Condition | ICD9CM |
| 44832370 | Secondary hypertension | 3-dig nonbill code | Condition | ICD9CM |
| 45930977 | Renal Hypertension Complicating Pregnancy, Childbirth and the Puerperium - Not Delivered | Diagnosis | Condition | CIEL |
| 45548700 | Unspecified pre-existing hypertension complicating pregnancy, childbirth and the puerperium | ICD10 code | Condition | ICD10 |
| 3124279 | Essential hypertension | Clinical Finding | Condition | Nebraska Lexicon |
| 45947705 | Toxemia of Pregnancy | Diagnosis | Condition | CIEL |
| 4218088 | Malignant hypertension in obstetric context | Clinical Finding | Condition | SNOMED |
| 40323860 | Malignant hypertension | Clinical Finding | Condition | SNOMED |
| 3386875 | Hypertension complicating pregnancy, childbirth and the puerperium | Clinical Finding | Condition | Nebraska Lexicon |
| 1571654 | Mild to moderate pre-eclampsia | 4-char nonbill code | Condition | ICD10CM |
| 44834876 | Transient hypertension of pregnancy, delivered, with mention of postpartum complication | 5-dig billing code | Condition | ICD9CM |
| 3380958 | Renal hypertension complicating pregnancy, childbirth and the puerperium | Clinical Finding | Condition | Nebraska Lexicon |
| 44832524 | Pre-eclampsia or eclampsia superimposed on pre-existing hypertension, postpartum condition or complication | 5-dig billing code | Condition | ICD9CM |
| 44783643 | Intermittent hypertension | Clinical Finding | Condition | SNOMED |
| 45917380 | secondary hypertension due to endocrine disorder | Diagnosis | Condition | CIEL |
| 4118910 | Maternal hypertension | Clinical Finding | Condition | SNOMED |
| 4146627 | Non-proteinuric hypertension of pregnancy | Clinical Finding | Condition | SNOMED |
| 3256113 | Secondary benign renovascular hypertension | Clinical Finding | Condition | Nebraska Lexicon |
| 45909529 | Malignant Secondary Hypertension | Diagnosis | Condition | CIEL |
| 3366242 | Accelerated secondary hypertension | Clinical Finding | Condition | Nebraska Lexicon |
| 4129829 | Toxemia NOS | Clinical Finding | Condition | SNOMED |
| 44833556 | Essential hypertension | 3-dig nonbill code | Condition | ICD9CM |
| 45939940 | Systolic Hypertension | Diagnosis | Condition | CIEL |
| 44796214 | [X]Hypertension secondary to other renal disorders | Clinical Finding | Condition | SNOMED |
| 40345175 | (Hypertensive disease) or (hypertension) | Clinical Finding | Condition | SNOMED |
| 40394313 | Mild &/or unspecified pre-eclampsia (& [toxemia NOS]) | Clinical Finding | Condition | SNOMED |
| 3405891 | Maternal hypertension | Clinical Finding | Condition | Nebraska Lexicon |
| 45539089 | Unspecified pre-existing hypertension complicating pregnancy, third trimester | 6-char billing code | Condition | ICD10CM |
| 45940918 | Rebound Hypertension | Diagnosis | Condition | CIEL |
| 45944168 | Pre-Eclampsia or Eclampsia with Pre-Existing Hypertension - Delivered with Postnatal Complication | Diagnosis | Condition | CIEL |
| 45548706 | Unspecified pre-eclampsia, unspecified trimester | 5-char billing code | Condition | ICD10CM |
| 45945902 | Hypertension Secondary to Renal Disease Complicating or Reason for Care During Pregnancy | Diagnosis | Condition | CIEL |
| 45534173 | Pre-existing secondary hypertension complicating childbirth | 5-char billing code | Condition | ICD10CM |
| 44824395 | Unspecified hypertension complicating pregnancy, childbirth, or the puerperium, delivered, with or without mention of antepartum condition | 5-dig billing code | Condition | ICD9CM |
| 3178834 | Secondary benign hypertension | Clinical Finding | Condition | Nebraska Lexicon |
| 3535048 | Secondary malignant hypertension NOS | Clinical Finding | Condition | SNOMED |
| 4289933 | Malignant hypertension | Clinical Finding | Condition | SNOMED |
| 45562338 | Secondary hypertension | ICD10 Hierarchy | Condition | ICD10 |
| 40318656 | Transient hypertension of pregnancy | Clinical Finding | Condition | SNOMED |
| 45923859 | Severe Pre-Eclampsia - Delivered | Diagnosis | Condition | CIEL |
| 3154349 | White coat hypertension | Clinical Finding | Condition | Nebraska Lexicon |
| 45596915 | Pre-existing essential hypertension complicating pregnancy, unspecified trimester | 6-char billing code | Condition | ICD10CM |
| 45567885 | Pre-existing essential hypertension complicating childbirth | 5-char billing code | Condition | ICD10CM |
| 3315191 | Severe pre-eclampsia with postnatal complication | Clinical Finding | Condition | Nebraska Lexicon |
| 1571635 | Pre-existing essential hypertension complicating pregnancy, childbirth and the puerperium | 4-char nonbill code | Condition | ICD10CM |
| 3141531 | Essential hypertension NOS | Clinical Finding | Condition | Nebraska Lexicon |
| 3377152 | Accelerated essential hypertension | Clinical Finding | Condition | Nebraska Lexicon |
| 35207678 | Other secondary hypertension | 4-char billing code | Condition | ICD10CM |
| 3126756 | Unspecified hypertension complicating pregnancy, childbirth and the puerperium - delivered | Clinical Finding | Condition | Nebraska Lexicon |
| 45934016 | Neonatal Hypertension | Diagnosis | Condition | CIEL |
| 3249390 | Mild proteinuric hypertension of pregnancy | Clinical Finding | Condition | Nebraska Lexicon |
| 3294712 | Systemic primary arterial hypertension | Clinical Finding | Condition | Nebraska Lexicon |
| 4269358 | Benign essential hypertension in obstetric context | Clinical Finding | Condition | SNOMED |
| 3290089 | Essential hypertension complicating AND/OR reason for care during pregnancy | Clinical Finding | Condition | Nebraska Lexicon |
| 3346249 | Renal sclerosis with hypertension | Clinical Finding | Condition | Nebraska Lexicon |
| 44835925 | Other unspecified secondary hypertension | 5-dig billing code | Condition | ICD9CM |
| 3262140 | Hypertension secondary to renal disease complicating AND/OR reason for care during puerperium | Clinical Finding | Condition | Nebraska Lexicon |
| 4049389 | Paroxysmal hypertension | Clinical Finding | Condition | SNOMED |
| 141084 | Pre-eclampsia or eclampsia with pre-existing hypertension | Clinical Finding | Condition | SNOMED |
| 4242878 | Sustained diastolic hypertension | Clinical Finding | Condition | SNOMED |
| 37200492 | Hypertensive crisis | 3-char nonbill code | Condition | ICD10CM |
| 42873163 | Hypertension complicating pregnancy, childbirth and the puerperium, antepartum | Clinical Finding | Condition | SNOMED |
| 45920865 | Hypertension Secondary to Renal Disease Complicating or Reason for Care During Childbirth | Diagnosis | Condition | CIEL |
| 141639 | Transient hypertension of pregnancy - delivered with postnatal complication | Clinical Finding | Condition | SNOMED |
| 44824236 | Other malignant secondary hypertension | 5-dig billing code | Condition | ICD9CM |
| 45771067 | Hypertension in chronic kidney disease due to type 1 diabetes mellitus | Clinical Finding | Condition | SNOMED |
| 45948045 | High Blood Pressure due to Other Cause | Diagnosis | Condition | CIEL |
| 3292357 | Eclampsia added to pre-existing hypertension | Clinical Finding | Condition | Nebraska Lexicon |
| 3222717 | Pre-eclampsia in puerperium | Clinical Finding | Condition | Nebraska Lexicon |
| 45915530 | Labile Diastolic Hypertension | Diagnosis | Condition | CIEL |
| 45928890 | Benign Essential Hypertension Complicating Pregnancy, Childbirth and the Puerperium with Postnatal Complication | Diagnosis | Condition | CIEL |
| 45956667 | Benign Essential Hypertension Complicating Pregnancy, Childbirth and the Puerperium - Delivered with Postnatal Complication | Diagnosis | Condition | CIEL |
| 3126754 | Unspecified hypertension complicating pregnancy, childbirth and the puerperium | Clinical Finding | Condition | Nebraska Lexicon |
| 45563054 | Unspecified maternal hypertension | ICD10 Hierarchy | Condition | ICD10 |
| 40323437 | Essential hypertension | Clinical Finding | Condition | SNOMED |
| 3182800 | Essential hypertension in obstetric context | Clinical Finding | Condition | Nebraska Lexicon |
| 1571634 | Pre-existing hypertension complicating pregnancy, childbirth and the puerperium | 3-char nonbill code | Condition | ICD10CM |
| 44809548 | Hypertension resistant to drug therapy | Clinical Finding | Condition | SNOMED |
| 4058542 | Unspecified hypertension complicating pregnancy, childbirth and the puerperium with postnatal complication | Clinical Finding | Condition | SNOMED |
| 3399815 | Renal hypertension complicating pregnancy, childbirth and the puerperium - not delivered | Clinical Finding | Condition | Nebraska Lexicon |
| 4304837 | Chronic hypertension complicating AND/OR reason for care during childbirth | Clinical Finding | Condition | SNOMED |
| 3126759 | Unspecified hypertension complicating pregnancy, childbirth and the puerperium with postnatal complication | Clinical Finding | Condition | Nebraska Lexicon |
| 3268727 | Benign essential hypertension in obstetric context | Clinical Finding | Condition | Nebraska Lexicon |
| 3524798 | Diastolic hypertension | Clinical Finding | Condition | SNOMED |
| 40394358 | Proteinuric hypertension of pregnancy | Clinical Finding | Condition | SNOMED |
| 44836074 | Other pre-existing hypertension, complicating pregnancy, childbirth, and the puerperium, delivered, with or without mention of antepartum condition | 5-dig billing code | Condition | ICD9CM |
| 3546635 | Stage 1 hypertension (NICE - National Institute for Health and Clinical Excellence 2011) with evidence of end organ damage | Clinical Finding | Condition | SNOMED |
| 134414 | Pre-eclampsia or eclampsia with pre-existing hypertension - delivered with postnatal complication | Clinical Finding | Condition | SNOMED |
| 44823109 | Malignant essential hypertension | 4-dig billing code | Condition | ICD9CM |
| 45563051 | Gestational [pregnancy-induced] hypertension without significant proteinuria, first trimester | 4-char billing code | Condition | ICD10CM |
| 44821949 | Unspecified essential hypertension | 4-dig billing code | Condition | ICD9CM |
| 4121926 | Pre-eclampsia NOS | Clinical Finding | Condition | SNOMED |
| 3545660 | Hypertension resistant to drug therapy | Clinical Finding | Condition | SNOMED |
| 3359351 | Gestational hypertension after childbirth | Clinical Finding | Condition | Nebraska Lexicon |
| 4058531 | Other pre-existing hypertension complicating pregnancy, childbirth and the puerperium unspecified | Clinical Finding | Condition | SNOMED |
| 4062550 | Pre-eclampsia or eclampsia with pre-existing hypertension with postnatal complication | Clinical Finding | Condition | SNOMED |
| 44819849 | Hypertension secondary to renal disease, complicating pregnancy, childbirth, and the puerperium, unspecified as to episode of care or not applicable | 5-dig billing code | Condition | ICD9CM |
| 3535049 | Secondary renovascular hypertension NOS | Clinical Finding | Condition | SNOMED |
| 45937830 | pre-existing hypertensive heart and renal disease complicating pregnancy, childbirth and the puerperium | Diagnosis | Condition | CIEL |
| 40398391 | Hypertensive disease | Clinical Finding | Condition | SNOMED |
| 762994 | Perioperative hypertension | Clinical Finding | Condition | SNOMED |
| 3335936 | Severe pre-eclampsia - delivered with postnatal complication | Clinical Finding | Condition | Nebraska Lexicon |
| 40318654 | Pregnancy with hypertension (& [pre-existing]) | Clinical Finding | Condition | SNOMED |
| 4279525 | Hypertension in the obstetric context | Clinical Finding | Condition | SNOMED |
| 40394309 | Hypertension induced by pregnancy | Clinical Finding | Condition | SNOMED |
| 45553477 | Mild to moderate pre-eclampsia | ICD10 code | Condition | ICD10 |
| 45548697 | Pre-existing hypertensive chronic kidney disease complicating pregnancy, second trimester | 6-char billing code | Condition | ICD10CM |
| 3221967 | Benign hypertension | Clinical Finding | Condition | Nebraska Lexicon |
| 4227607 | Chronic hypertension in obstetric context | Clinical Finding | Condition | SNOMED |
| 3266749 | Pre-existing hypertension, NOS, complicating or reason for care during pregnancy | Clinical Finding | Condition | Nebraska Lexicon |
| 45915433 | Malignant Essential Hypertension | Diagnosis | Condition | CIEL |
| 45939245 | Pre-Existing Hypertension, Complicating or Reason for Care During Childbirth | Diagnosis | Condition | CIEL |
| 4289142 | Eclampsia added to pre-existing hypertension | Clinical Finding | Condition | SNOMED |
| 3124306 | Other specified hypertensive disease | Clinical Finding | Condition | Nebraska Lexicon |
| 4302591 | Essential hypertension complicating AND/OR reason for care during pregnancy | Clinical Finding | Condition | SNOMED |
| 3347715 | Benign essential hypertension complicating pregnancy, childbirth and the puerperium | Clinical Finding | Condition | Nebraska Lexicon |
| 45548704 | Gestational [pregnancy-induced] hypertension without significant proteinuria, unspecified trimester | 4-char billing code | Condition | ICD10CM |
| 37200493 | Hypertensive urgency | 4-char billing code | Condition | ICD10CM |
| 4291933 | Chronic hypertension complicating AND/OR reason for care during pregnancy | Clinical Finding | Condition | SNOMED |
| 45910475 | Benign Hypertension | Diagnosis | Condition | CIEL |
| 45912740 | HYPERTENSION | Diagnosis | Condition | CIEL |
| 45924033 | Hypertension, Surgical Complication | Diagnosis | Condition | CIEL |
| 4062549 | Pre-eclampsia or eclampsia with pre-existing hypertension unspecified | Clinical Finding | Condition | SNOMED |
| 318437 | Malignant secondary hypertension | Clinical Finding | Condition | SNOMED |
| 3353736 | Labile diastolic hypertension | Clinical Finding | Condition | Nebraska Lexicon |
| 40318657 | Mild pre-eclampsia | Clinical Finding | Condition | SNOMED |
| 3229828 | Hypertension in the puerperium with pulmonary oedema | Clinical Finding | Condition | Nebraska Lexicon |
| 45949415 | pre-existing hypertensive renal disease complicating pregnancy, childbirth and the puerperium | Diagnosis | Condition | CIEL |
| 3142112 | Pregnancy with pre-existing hypertension | Clinical Finding | Condition | Nebraska Lexicon |
| 44811110 | Nocturnal hypertension | Clinical Finding | Condition | SNOMED |
| 4028951 | Malignant hypertension complicating AND/OR reason for care during puerperium | Clinical Finding | Condition | SNOMED |
| 45954712 | Malignant Hypertension Complicating or Reason for Care During Puerperium | Diagnosis | Condition | CIEL |
| 314090 | Mild pre-eclampsia | Clinical Finding | Condition | SNOMED |
| 45757137 | Pre-existing hypertensive heart and chronic kidney disease in mother complicating childbirth | Clinical Finding | Condition | SNOMED |
| 44826776 | Pre-eclampsia or eclampsia superimposed on pre-existing hypertension, unspecified as to episode of care or not applicable | 5-dig billing code | Condition | ICD9CM |
| 3187117 | Malignant hypertension complicating AND/OR reason for care during childbirth | Clinical Finding | Condition | Nebraska Lexicon |
| 45943545 | Transient Hypertension of Pregnancy - Not Delivered | Diagnosis | Condition | CIEL |
| 3545643 | Severe hypertension | Clinical Finding | Condition | SNOMED |
| 3126716 | Benign essential hypertension complicating pregnancy, childbirth and the puerperium NOS | Clinical Finding | Condition | Nebraska Lexicon |
| 45944916 | Malignant Hypertension in Obstetric Context | Diagnosis | Condition | CIEL |
| 44833713 | Hypertension complicating pregnancy, childbirth, and the puerperium | 3-dig nonbill code | Condition | ICD9CM |
| 40394357 | Syndrome of hemolysis, elevated liver enzymes and low platelet | Clinical Finding | Condition | SNOMED |
| 3145604 | Pre-eclampsia, unspecified | Clinical Finding | Condition | Nebraska Lexicon |
| 37200971 | HELLP syndrome, complicating childbirth | 5-char billing code | Condition | ICD10CM |
| 45534174 | Pre-existing hypertension with pre-eclampsia, second trimester | 4-char billing code | Condition | ICD10CM |
| 3338521 | Proteinuric hypertension of pregnancy | Clinical Finding | Condition | Nebraska Lexicon |
| 3126722 | Other pre-existing hypertension complicating pregnancy, childbirth and the puerperium - delivered with postnatal complication | Clinical Finding | Condition | Nebraska Lexicon |
| 37200957 | Pre-existing hypertension with pre-eclampsia, complicating childbirth | 4-char billing code | Condition | ICD10CM |
| 3274097 | Labile essential hypertension | Clinical Finding | Condition | Nebraska Lexicon |
| 44825589 | Severe pre-eclampsia, delivered, with or without mention of antepartum condition | 5-dig billing code | Condition | ICD9CM |
| 3535645 | Other pre-existing hypertension complicating pregnancy, childbirth and the puerperium - delivered | Clinical Finding | Condition | SNOMED |
| 44821021 | Severe pre-eclampsia | 4-dig nonbill code | Condition | ICD9CM |
| 3311037 | Hypertension secondary to renal disease complicating AND/OR reason for care during childbirth | Clinical Finding | Condition | Nebraska Lexicon |
| 44827932 | Other pre-existing hypertension, complicating pregnancy, childbirth, and the puerperium, delivered, with mention of postpartum complication | 5-dig billing code | Condition | ICD9CM |
| 314958 | Benign secondary hypertension | Clinical Finding | Condition | SNOMED |
| 44830078 | Unspecified renovascular hypertension | 5-dig billing code | Condition | ICD9CM |
| 3126728 | Hypertension induced by pregnancy | Clinical Finding | Condition | Nebraska Lexicon |
| 3245939 | Hypertension in antepartum pregnancy | Clinical Finding | Condition | Nebraska Lexicon |
| 40398394 | Systolic hypertension | Clinical Finding | Condition | SNOMED |
| 45907231 | Renovascular Hypertension | Diagnosis | Condition | CIEL |
| 45931743 | Eclampsia (Toxemia of Pregnancy) | Diagnosis | Condition | CIEL |
| 3124301 | Secondary benign renovascular hypertension | Clinical Finding | Condition | Nebraska Lexicon |
| 3279755 | Hypertension in chronic kidney disease stage 2 due to type 2 diabetes mellitus | Clinical Finding | Condition | Nebraska Lexicon |
| 4062547 | Severe pre-eclampsia NOS | Clinical Finding | Condition | SNOMED |
| 197930 | Renal hypertension complicating pregnancy, childbirth and the puerperium | Clinical Finding | Condition | SNOMED |
| 4249016 | Benign secondary renovascular hypertension | Clinical Finding | Condition | SNOMED |
| 45933333 | Benign Essential Hypertension Complicating or Reason for Care During Pregnancy | Diagnosis | Condition | CIEL |
| 3230119 | Hypertension complicating pregnancy | Clinical Finding | Condition | Nebraska Lexicon |
| 45951147 | Benign Secondary Renovascular Hypertension | Diagnosis | Condition | CIEL |
| 3436673 | Haemolysis-elevated liver enzymes-low platelet count syndrome | Clinical Finding | Condition | Nebraska Lexicon |
| 137940 | Transient hypertension of pregnancy - delivered | Clinical Finding | Condition | SNOMED |
| 45582445 | Pre-existing secondary hypertension complicating pregnancy, second trimester | 6-char billing code | Condition | ICD10CM |
| 4174760 | Labile hypertension | Clinical Finding | Condition | SNOMED |
| 45563048 | Pre-existing secondary hypertension complicating the puerperium | 5-char billing code | Condition | ICD10CM |
| 4110947 | Malignant secondary renovascular hypertension | Clinical Finding | Condition | SNOMED |
| 45572077 | Renovascular hypertension | ICD10 code | Condition | ICD10 |
| 4110950 | Other specified hypertensive disease | Clinical Finding | Condition | SNOMED |
| 4221991 | Rebound hypertension | Clinical Finding | Condition | SNOMED |
| 3126741 | Severe pre-eclampsia unspecified | Clinical Finding | Condition | Nebraska Lexicon |
| 45920281 | Essential Hypertension Complicating and/or Reason for Care During Pregnancy | Diagnosis | Condition | CIEL |
| 45582446 | Unspecified pre-existing hypertension complicating pregnancy, first trimester | 6-char billing code | Condition | ICD10CM |
| 4062906 | Transient hypertension of pregnancy with postnatal complication | Clinical Finding | Condition | SNOMED |
| 45592183 | Pre-existing essential hypertension complicating the puerperium | 5-char billing code | Condition | ICD10CM |
| 40398431 | Secondary hypertension NOS | Clinical Finding | Condition | SNOMED |
| 3250794 | Paroxysmal hypertension | Clinical Finding | Condition | Nebraska Lexicon |
| 4212496 | Parenchymal renal hypertension | Clinical Finding | Condition | SNOMED |
| 45937201 | Maternal Hypertension | Diagnosis | Condition | CIEL |
| 3287028 | Pre-existing hypertension complicating pregnancy, childbirth and puerperium | Clinical Finding | Condition | Nebraska Lexicon |
| 44824389 | Benign essential hypertension complicating pregnancy, childbirth, and the puerperium, unspecified as to episode of care or not applicable | 5-dig billing code | Condition | ICD9CM |
| 1571644 | Pre-existing secondary hypertension complicating pregnancy | 5-char nonbill code | Condition | ICD10CM |
| 40358543 | Pregnancy with hypertension (& [pre-existing]) | Clinical Finding | Condition | SNOMED |
| 45757445 | Hypertension in chronic kidney disease stage 4 due to type 2 diabetes mellitus | Clinical Finding | Condition | SNOMED |
| 45755599 | Pre-existing hypertension complicating pregnancy, childbirth and the puerperium | ICD10 Hierarchy | Condition | ICD10 |
| 4167493 | Pregnancy-induced hypertension | Clinical Finding | Condition | SNOMED |
| 1571641 | Pre-existing hypertensive heart and chronic kidney disease complicating pregnancy, childbirth and the puerperium | 4-char nonbill code | Condition | ICD10CM |
| 3159636 | Benign secondary hypertension | Clinical Finding | Condition | Nebraska Lexicon |
| 40563470 | White coat hypertension | Clinical Finding | Condition | SNOMED |
| 45937510 | Hypertensive emergency | Diagnosis | Condition | CIEL |
| 45757446 | Hypertension in chronic kidney disease stage 3 due to type 2 diabetes mellitus | Clinical Finding | Condition | SNOMED |
| 3155031 | White coat hypertension | Clinical Finding | Condition | Nebraska Lexicon |
| 45539090 | Unspecified pre-existing hypertension complicating childbirth | 5-char billing code | Condition | ICD10CM |
| 3372614 | Diastolic hypertension co-occurrent with systolic hypertension | Clinical Finding | Condition | Nebraska Lexicon |
| 3363749 | Hypertensive disorder, systemic arterial | Clinical Finding | Condition | Nebraska Lexicon |
| 45924277 | Essential Hypertension in Obstetric Context | Diagnosis | Condition | CIEL |
| 40394310 | Transient hypertension of pregnancy | Clinical Finding | Condition | SNOMED |
| 3424342 | Supine hypertension | Clinical Finding | Condition | Nebraska Lexicon |
| 3105649 | Secondary hypertension | Clinical Finding | Condition | Nebraska Lexicon |
| 45548698 | Pre-existing hypertensive heart and renal disease complicating pregnancy, childbirth and the puerperium | ICD10 code | Condition | ICD10 |
| 45572763 | HELLP syndrome (HELLP), second trimester | 5-char billing code | Condition | ICD10CM |
| 3126738 | Pre-eclampsia NOS | Clinical Finding | Condition | Nebraska Lexicon |
| 45954065 | Severe Uncontrolled Hypertension | Diagnosis | Condition | CIEL |
| 3535050 | Other specified hypertensive disease | Clinical Finding | Condition | SNOMED |
| 4071202 | Neonatal hypertension | Clinical Finding | Condition | SNOMED |
| 3378857 | Hypertension secondary to kidney transplant | Clinical Finding | Condition | Nebraska Lexicon |
| 3395794 | Secondary malignant renovascular hypertension | Clinical Finding | Condition | Nebraska Lexicon |
| 4057974 | Mild or unspecified pre-eclampsia - delivered with postnatal complication | Clinical Finding | Condition | SNOMED |
| 3407752 | Renal hypertension complicating pregnancy, childbirth and the puerperium - delivered | Clinical Finding | Condition | Nebraska Lexicon |
| 45558203 | Pre-existing secondary hypertension complicating pregnancy, first trimester | 6-char billing code | Condition | ICD10CM |
| 45926665 | HELLP (hemolytic anemia/elev liver enzymes/low platelets in pregnancy) | Diagnosis | Condition | CIEL |
| 3126742 | Severe pre-eclampsia NOS | Clinical Finding | Condition | Nebraska Lexicon |
| 4058539 | Pre-eclampsia or eclampsia with pre-existing hypertension NOS | Clinical Finding | Condition | SNOMED |
| 3534305 | Pre-eclampsia or eclampsia with pre-existing hypertension unspecified | Clinical Finding | Condition | SNOMED |
| 45908656 | Parenchymal Renal Hypertension | Diagnosis | Condition | CIEL |
| 3126760 | Unspecified hypertension complicating pregnancy, childbirth and the puerperium NOS | Clinical Finding | Condition | Nebraska Lexicon |
| 45582448 | HELLP syndrome | 4-char nonbill code | Condition | ICD10CM |
| 4062904 | Other pre-existing hypertension complicating pregnancy, childbirth and the puerperium with postnatal complication | Clinical Finding | Condition | SNOMED |
| 45577558 | Unspecified pre-existing hypertension complicating pregnancy, unspecified trimester | 6-char billing code | Condition | ICD10CM |
| 45915431 | Malignant Hypertension Complicating or Reason for Care During Childbirth | Diagnosis | Condition | CIEL |
| 45931863 | Sustained Diastolic Hypertension | Diagnosis | Condition | CIEL |
| 320128 | Essential hypertension | Clinical Finding | Condition | SNOMED |
| 45930633 | unspecified maternal hypertension, unspecified as to episode of care, or not applicable | Diagnosis | Condition | CIEL |
| 45939244 | Pre-Existing Hypertension, Complicating or Reason for Care During Pregnancy | Diagnosis | Condition | CIEL |
| 44836075 | Other pre-existing hypertension,complicating pregnancy, childbirth, and the puerperium, , postpartum condition or complication | 5-dig billing code | Condition | ICD9CM |
| 3254516 | Hypertension in chronic kidney disease due to type 2 diabetes mellitus | Clinical Finding | Condition | Nebraska Lexicon |
| 45920864 | Hypertension Secondary to Renal Disease in Obstetric Context | Diagnosis | Condition | CIEL |
| 45587280 | Pre-existing hypertensive chronic kidney disease complicating pregnancy, third trimester | 6-char billing code | Condition | ICD10CM |
| 4061667 | Hypertension induced by oral contraceptive pill | Clinical Finding | Condition | SNOMED |
| 37200965 | Gestational [pregnancy-induced] hypertension without significant proteinuria, complicating childbirth | 4-char billing code | Condition | ICD10CM |
| 44837098 | Unspecified secondary hypertension | 4-dig nonbill code | Condition | ICD9CM |
| 3216451 | Pre-existing hypertension, NOS, complicating or reason for care during childbirth | Clinical Finding | Condition | Nebraska Lexicon |
| 37208293 | Multiple drug intolerant hypertension | Clinical Finding | Observation | SNOMED |
| 4111387 | Secondary malignant hypertension NOS | Clinical Finding | Condition | SNOMED |
| 438490 | Severe pre-eclampsia - delivered | Clinical Finding | Condition | SNOMED |
| 45928891 | Benign Essential Hypertension Complicating Pregnancy, Childbirth and the Puerperium | Diagnosis | Condition | CIEL |
| 3124300 | Secondary malignant hypertension NOS | Clinical Finding | Condition | Nebraska Lexicon |
| 3144554 | White coat hypertension | Clinical Finding | Condition | Nebraska Lexicon |
| 4080325 | Chronic hypertension complicating AND/OR reason for care during puerperium | Clinical Finding | Condition | SNOMED |
| 3421186 | Hypertension secondary to endocrine disorder | Clinical Finding | Condition | Nebraska Lexicon |
| 4143075 | Mild or unspecified pre-eclampsia | Clinical Finding | Condition | SNOMED |
| 3352019 | Benign essential hypertension complicating AND/OR reason for care during childbirth | Clinical Finding | Condition | Nebraska Lexicon |
| 45757444 | Hypertension in chronic kidney disease stage 5 due to type 2 diabetes mellitus | Clinical Finding | Condition | SNOMED |
| 45938645 | Malignant Hypertension | Diagnosis | Condition | CIEL |
| 3339592 | Neonatal hypertension | Clinical Finding | Condition | Nebraska Lexicon |
| 1571640 | Pre-existing hypertensive chronic kidney disease complicating pregnancy | 5-char nonbill code | Condition | ICD10CM |
| 3335984 | Pre-existing essential hypertension in postpartum | Clinical Finding | Condition | Nebraska Lexicon |
| 3264606 | Pre-existing hypertension complicating pregnancy | Clinical Finding | Condition | Nebraska Lexicon |
| 44809569 | Stage 2 hypertension (NICE - National Institute for Health and Clinical Excellence 2011) | Clinical Finding | Condition | SNOMED |
| 45577561 | HELLP syndrome (HELLP), unspecified trimester | 5-char billing code | Condition | ICD10CM |
| 1571645 | Unspecified pre-existing hypertension complicating pregnancy, childbirth and the puerperium | 4-char nonbill code | Condition | ICD10CM |
| 4057979 | Pre-existing secondary hypertension complicating pregnancy, childbirth and puerperium | Clinical Finding | Condition | SNOMED |
| 40398396 | Hypertension NOS (& [essential]) | Clinical Finding | Condition | SNOMED |
| 3126751 | Moderate proteinuric hypertension of pregnancy | Clinical Finding | Condition | Nebraska Lexicon |
| 42538946 | Hypertension complicating pregnancy | Clinical Finding | Condition | SNOMED |
| 3124303 | Secondary hypertension NOS | Clinical Finding | Condition | Nebraska Lexicon |
| 35207675 | Renovascular hypertension | 4-char billing code | Condition | ICD10CM |
| 37200970 | Severe pre-eclampsia, complicating the puerperium | 5-char billing code | Condition | ICD10CM |
| 3409733 | Secondary diastolic hypertension | Clinical Finding | Condition | Nebraska Lexicon |
| 45937061 | Transient Hypertension of Pregnancy - Delivered with Postnatal Complication | Diagnosis | Condition | CIEL |
| 4058534 | Severe pre-eclampsia unspecified | Clinical Finding | Condition | SNOMED |
| 4062811 | Benign essential hypertension complicating pregnancy, childbirth and the puerperium with postnatal complication | Clinical Finding | Condition | SNOMED |
| 4062812 | Other pre-existing hypertension complicating pregnancy, childbirth and the puerperium - delivered | Clinical Finding | Condition | SNOMED |
| 3321959 | Rebound hypertension | Clinical Finding | Condition | Nebraska Lexicon |
| 40394323 | Severe proteinuric hypertension of pregnancy | Clinical Finding | Condition | SNOMED |
| 3535481 | Other pre-existing hypertension complicating pregnancy, childbirth and the puerperium | Clinical Finding | Condition | SNOMED |
| 3105646 | Essential hypertension | Clinical Finding | Condition | Nebraska Lexicon |
| 37200972 | HELLP syndrome, complicating the puerperium | 5-char billing code | Condition | ICD10CM |
| 45558202 | Pre-existing hypertensive heart and chronic kidney disease complicating childbirth | 5-char billing code | Condition | ICD10CM |
| 37200966 | Gestational [pregnancy-induced] hypertension without significant proteinuria, complicating the puerperium | 4-char billing code | Condition | ICD10CM |
| 3244126 | Hypertension in chronic kidney disease stage 5 due to type 2 diabetes mellitus | Clinical Finding | Condition | Nebraska Lexicon |
| 3386388 | Transient hypertension | Clinical Finding | Condition | Nebraska Lexicon |
| 3337772 | Transient hypertension of pregnancy - delivered | Clinical Finding | Condition | Nebraska Lexicon |
| 45582443 | Pre-existing hypertensive renal disease complicating pregnancy, childbirth and the puerperium | ICD10 code | Condition | ICD10 |
| 45908380 | Renal Sclerosis with Hypertension | Diagnosis | Condition | CIEL |
| 3153655 | White coat hypertension | Clinical Finding | Condition | Nebraska Lexicon |
| 3536733 | Unspecified hypertension complicating pregnancy, childbirth and the puerperium - delivered with postnatal complication | Clinical Finding | Condition | SNOMED |
| 3402341 | Hypertension secondary to renal disease in obstetric context | Clinical Finding | Condition | Nebraska Lexicon |
| 45558204 | Unspecified pre-eclampsia, third trimester | 5-char billing code | Condition | ICD10CM |
| 45925219 | Severe pre-eclampsia | Diagnosis | Condition | CIEL |
| 44784483 | Pregnancy induced hypertension with pulmonary edema | Clinical Finding | Condition | SNOMED |
| 45757788 | Postpartum pregnancy-induced hypertension | Clinical Finding | Condition | SNOMED |
| 44831234 | Malignant renovascular hypertension | 5-dig billing code | Condition | ICD9CM |
| 4057976 | Severe pre-eclampsia with postnatal complication | Clinical Finding | Condition | SNOMED |
| 3126725 | Other pre-existing hypertension complicating pregnancy, childbirth and the puerperium NOS | Clinical Finding | Condition | Nebraska Lexicon |
| 136743 | Pre-eclampsia or eclampsia with pre-existing hypertension - not delivered | Clinical Finding | Condition | SNOMED |
| 37200494 | Hypertensive emergency | 4-char billing code | Condition | ICD10CM |
| 3310519 | Renal hypertension complicating pregnancy, childbirth and the puerperium - delivered with postnatal complication | Clinical Finding | Condition | Nebraska Lexicon |
| 439393 | Pre-eclampsia | Clinical Finding | Condition | SNOMED |
| 44830211 | Other pre-existing hypertension, complicating pregnancy, childbirth, and the puerperium, antepartum condition or complication | 5-dig billing code | Condition | ICD9CM |
| 3138229 | Toxaemia NOS | Clinical Finding | Condition | Nebraska Lexicon |
| 1571653 | Pre-eclampsia | 3-char nonbill code | Condition | ICD10CM |
| 45953665 | Transient Hypertension | Diagnosis | Condition | CIEL |
| 44830210 | Benign essential hypertension complicating pregnancy, childbirth, and the puerperium, postpartum condition or complication | 5-dig billing code | Condition | ICD9CM |
| 3335853 | Hypertensive crisis | Clinical Finding | Condition | Nebraska Lexicon |
| 44783644 | Labile hypertension due to being in a clinical environment | Clinical Finding | Condition | SNOMED |
| 3398326 | Malignant hypertension complicating AND/OR reason for care during pregnancy | Clinical Finding | Condition | Nebraska Lexicon |
| 3124281 | Systolic hypertension | Clinical Finding | Condition | Nebraska Lexicon |
| 3126757 | Unspecified hypertension complicating pregnancy, childbirth and the puerperium - delivered with postnatal complication | Clinical Finding | Condition | Nebraska Lexicon |
| 3143786 | Non-proteinuric hypertension of pregnancy | Clinical Finding | Condition | Nebraska Lexicon |
| 3422085 | Benign essential hypertension complicating pregnancy, childbirth and the puerperium with postnatal complication | Clinical Finding | Condition | Nebraska Lexicon |
| 45563053 | HELLP syndrome (HELLP), third trimester | 5-char billing code | Condition | ICD10CM |
| 317898 | Malignant essential hypertension | Clinical Finding | Condition | SNOMED |
| 40318659 | Severe proteinuric hypertension of pregnancy | Clinical Finding | Condition | SNOMED |
| 3321322 | Sustained diastolic hypertension | Clinical Finding | Condition | Nebraska Lexicon |
| 4146816 | Pre-existing hypertension complicating AND/OR reason for care during childbirth | Clinical Finding | Condition | SNOMED |
| 1571655 | Severe pre-eclampsia | 4-char nonbill code | Condition | ICD10CM |
| 44837235 | Unspecified hypertension complicating pregnancy, childbirth, or the puerperium, unspecified as to episode of care or not applicable | 5-dig billing code | Condition | ICD9CM |
| 3287433 | Hypertension due to gain-of-function mutation in mineralocorticoid receptor | Clinical Finding | Condition | Nebraska Lexicon |
| 42538697 | Diastolic hypertension and systolic hypertension | Clinical Finding | Condition | SNOMED |
| 45924034 | High blood pressure | Diagnosis | Condition | CIEL |
| 4057972 | Benign essential hypertension complicating pregnancy, childbirth and the puerperium NOS | Clinical Finding | Condition | SNOMED |
| 3259587 | Essential hypertension complicating AND/OR reason for care during puerperium | Clinical Finding | Condition | Nebraska Lexicon |
| 40323436 | (Hypertensive disease) or (hypertension) | Clinical Finding | Condition | SNOMED |
| 433536 | Severe pre-eclampsia | Clinical Finding | Condition | SNOMED |
| 3169253 | Hypertension with apparent mineralocorticoid excess | Clinical Finding | Condition | Nebraska Lexicon |
| 3070738 | Hypertension NOS (& [essential]) | Clinical Finding | Condition | Nebraska Lexicon |
| 3146763 | Gestational hypertension | Clinical Finding | Condition | Nebraska Lexicon |
| 4057971 | Benign essential hypertension complicating pregnancy, childbirth and the puerperium unspecified | Clinical Finding | Condition | SNOMED |
| 4276511 | Labile diastolic hypertension | Clinical Finding | Condition | SNOMED |
| 44836076 | Transient hypertension of pregnancy, unspecified as to episode of care or not applicable | 5-dig billing code | Condition | ICD9CM |
| 3337417 | Hypertension induced by oral contraceptive pill | Clinical Finding | Condition | Nebraska Lexicon |
| 3290389 | Exertional hypertension | Clinical Finding | Condition | Nebraska Lexicon |
| 44826774 | Transient hypertension of pregnancy | 4-dig nonbill code | Condition | ICD9CM |
| 4006325 | Hypertension secondary to renal disease in obstetric context | Clinical Finding | Condition | SNOMED |
| 43020424 | Hypertensive emergency | Clinical Finding | Condition | SNOMED |
| 3126733 | Mild or unspecified pre-eclampsia - delivered | Clinical Finding | Condition | Nebraska Lexicon |
| 44795761 | [X]Other secondary hypertension | Clinical Finding | Condition | SNOMED |
| 45592191 | Unspecified maternal hypertension, second trimester | 4-char billing code | Condition | ICD10CM |
| 44822082 | Mild or unspecified pre-eclampsia, delivered, with or without mention of antepartum condition | 5-dig billing code | Condition | ICD9CM |
| 45944170 | Pre-eclampsia | Diagnosis | Condition | CIEL |
| 3105652 | Hypertensive disease NOS | Clinical Finding | Condition | Nebraska Lexicon |
| 3287690 | Chronic hypertension in obstetric context | Clinical Finding | Condition | Nebraska Lexicon |
| 3126753 | Proteinuric hypertension of pregnancy | Clinical Finding | Condition | Nebraska Lexicon |
| 45548703 | Gestational [pregnancy-induced] hypertension without significant proteinuria, second trimester | 4-char billing code | Condition | ICD10CM |
| 45577702 | Neonatal hypertension | ICD10 code | Condition | ICD10 |
| 3071766 | Transient hypertension of pregnancy | Clinical Finding | Condition | Nebraska Lexicon |
| 3126730 | Transient hypertension of pregnancy NOS | Clinical Finding | Condition | Nebraska Lexicon |
| 45927463 | Paroxysmal Hypertension | Diagnosis | Condition | CIEL |
| 3217809 | Malignant hypertension in obstetric context | Clinical Finding | Condition | Nebraska Lexicon |
| 3106384 | Transient hypertension of pregnancy | Clinical Finding | Condition | Nebraska Lexicon |
| 4108667 | Secondary renovascular hypertension NOS | Clinical Finding | Condition | SNOMED |
| 4094374 | Hypertension secondary to renal disease complicating AND/OR reason for care during childbirth | Clinical Finding | Condition | SNOMED |
| 3185553 | Benign essential hypertension complicating pregnancy, childbirth and the puerperium - delivered | Clinical Finding | Condition | Nebraska Lexicon |
| 3142113 | Hypertension of pregnancy | Clinical Finding | Condition | Nebraska Lexicon |
| 44824394 | Pre-eclampsia or eclampsia superimposed on pre-existing hypertension | 4-dig nonbill code | Condition | ICD9CM |
| 45933332 | Benign Essential Hypertension Complicating Pregnancy, Childbirth and the Puerperium - Delivered | Diagnosis | Condition | CIEL |
| 3541588 | Transient hypertension of pregnancy unspecified | Clinical Finding | Condition | SNOMED |
| 45929018 | Eclampsia Added to Pre-Existing Hypertension | Diagnosis | Condition | CIEL |
| 4180283 | Systolic essential hypertension | Clinical Finding | Condition | SNOMED |
| 45909952 | Essential Hypertension Complicating or Reason for Care During Puerperium | Diagnosis | Condition | CIEL |
| 40394320 | Pre-eclampsia NOS | Clinical Finding | Condition | SNOMED |
| 37208172 | Resistant hypertensive disorder | Clinical Finding | Condition | SNOMED |
| 4062424 | Renal hypertension complicating pregnancy, childbirth and the puerperium unspecified | Clinical Finding | Condition | SNOMED |
| 3307436 | Hypertension associated with transplantation | Clinical Finding | Condition | Nebraska Lexicon |
| 45918974 | Pregnancy Induced Hypertension | Diagnosis | Condition | CIEL |
| 40398392 | High blood pressure (& [essential hypertension]) | Clinical Finding | Condition | SNOMED |
| 40398424 | Secondary benign renovascular hypertension | Clinical Finding | Condition | SNOMED |
| 3126748 | Pre-eclampsia or eclampsia with pre-existing hypertension unspecified | Clinical Finding | Condition | Nebraska Lexicon |
| 40318655 | Hypertension of pregnancy (& [pre-eclampsia]) | Clinical Finding | Condition | SNOMED |
| 3126719 | Other pre-existing hypertension in preg/childbirth/puerp | Clinical Finding | Condition | Nebraska Lexicon |
| 4062554 | Unspecified hypertension complicating pregnancy, childbirth and the puerperium - not delivered | Clinical Finding | Condition | SNOMED |
| 45933331 | Benign Essential Hypertension in Obstetric Context | Diagnosis | Condition | CIEL |
| 4150005 | Hypertension of pregnancy NOS | Clinical Finding | Condition | SNOMED |
| 44831376 | Benign essential hypertension complicating pregnancy, childbirth, and the puerperium | 4-dig nonbill code | Condition | ICD9CM |
| 45940518 | Mild Pre-Eclampsia | Diagnosis | Condition | CIEL |
| 45596917 | Pre-existing hypertension with pre-eclampsia, unspecified trimester | 4-char billing code | Condition | ICD10CM |
| 40322494 | Maternal hypertension | Clinical Finding | Condition | SNOMED |
| 45944846 | Benign Essential Hypertension Complicating or Reason for Care During Puerperium | Diagnosis | Condition | CIEL |
| 3545667 | Stage 2 hypertension (NICE - National Institute for Health and Clinical Excellence 2011) | Clinical Finding | Condition | SNOMED |
| 40373519 | Malignant hypertension | Clinical Finding | Condition | SNOMED |
| 3243079 | Malignant hypertension | Clinical Finding | Condition | Nebraska Lexicon |
| 44834878 | Severe pre-eclampsia, delivered, with mention of postpartum complication | 5-dig billing code | Condition | ICD9CM |
| 132685 | Severe pre-eclampsia - not delivered | Clinical Finding | Condition | SNOMED |
| 3405241 | Benign essential hypertension complicating pregnancy, childbirth and the puerperium - not delivered | Clinical Finding | Condition | Nebraska Lexicon |
| 3271124 | Renal hypertension complicating pregnancy, childbirth and the puerperium with postnatal complication | Clinical Finding | Condition | Nebraska Lexicon |
| 4108213 | Hypertension secondary to drug | Clinical Finding | Condition | SNOMED |
| 40318662 | Hypertension of pregnancy NOS | Clinical Finding | Condition | SNOMED |
| 3145096 | Malignant hypertension | Clinical Finding | Condition | Nebraska Lexicon |
| 37200969 | Severe pre-eclampsia complicating childbirth | 5-char billing code | Condition | ICD10CM |
| 201912 | Renal hypertension complicating pregnancy, childbirth and the puerperium - delivered with postnatal complication | Clinical Finding | Condition | SNOMED |
| 45596916 | Pre-existing hypertension with pre-eclampsia, first trimester | 4-char billing code | Condition | ICD10CM |
| 3137408 | Malignant secondary renovascular hypertension | Clinical Finding | Condition | Nebraska Lexicon |
| 3344473 | Severe proteinuric hypertension of pregnancy | Clinical Finding | Condition | Nebraska Lexicon |
| 37200958 | Pre-existing hypertension with pre-eclampsia, complicating the puerperium | 4-char billing code | Condition | ICD10CM |
| 40619422 | Pre-eclampsia | Clinical Finding | Condition | SNOMED |

Table 3. Diabetes (any).

| **Concept ID** | **Concept Name** | **Class** | **Domain** | **Vocabulary** | |
| --- | --- | --- | --- | --- | --- |
| 4252356 | O/E - left eye proliferative diabetic retinopathy | Clinical Finding | Condition | SNOMED |
| 4270049 | Nodular type diabetic glomerulosclerosis | Clinical Finding | Condition | SNOMED |
| 4044391 | Neuropathy due to diabetes mellitus | Clinical Finding | Condition | SNOMED |
| 4174977 | Retinopathy due to diabetes mellitus | Clinical Finding | Condition | SNOMED |
| 377821 | Disorder of nervous system due to type 1 diabetes mellitus | Clinical Finding | Condition | SNOMED |
| 4131907 | Lipoatrophic diabetes | Clinical Finding | Condition | SNOMED |
| 3181307 | Iatrogenic diabetes insipidus | Clinical Finding | Condition | Nebraska Lexicon |
| 4200875 | Type 2 diabetes mellitus with peripheral angiopathy | Clinical Finding | Condition | SNOMED |
| 45757432 | Hyperlipidemia due to type 1 diabetes mellitus | Clinical Finding | Condition | SNOMED |
| 43531019 | Diabetes mellitus, transient neonatal 2 | Clinical Finding | Condition | SNOMED |
| 4297627 | Idiopathic diabetes insipidus | Clinical Finding | Condition | SNOMED |
| 4178452 | Diabetes mellitus associated with pancreatic disease | Clinical Finding | Condition | SNOMED |
| 4144583 | Diabetes mellitus due to cystic fibrosis | Clinical Finding | Condition | SNOMED |
| 318712 | Peripheral circulatory disorder due to type 1 diabetes mellitus | Clinical Finding | Condition | SNOMED |
| 4228112 | Hypoglycemic coma due to type 1 diabetes mellitus | Clinical Finding | Condition | SNOMED |
| 45773688 | Chronic kidney disease due to type 1 diabetes mellitus | Clinical Finding | Condition | SNOMED |
| 4016047 | Loss of hypoglycemic warning due to diabetes mellitus | Clinical Finding | Condition | SNOMED |
| 37016356 | Mild nonproliferative retinopathy due to secondary diabetes mellitus | Clinical Finding | Condition | SNOMED |
| 35625724 | Bilateral iritis due to diabetes mellitus | Clinical Finding | Condition | SNOMED |
| 35626038 | Mild nonproliferative retinopathy of right eye due to diabetes mellitus | Clinical Finding | Condition | SNOMED |
| 4224419 | Disorder of eye due to malnutrition related diabetes mellitus | Clinical Finding | Condition | SNOMED |
| 4294429 | Wet gangrene of foot due to diabetes mellitus | Clinical Finding | Condition | SNOMED |
| 4079850 | Diabetes mellitus in neonate small for gestational age | Clinical Finding | Condition | SNOMED |
| 765373 | Severe nonproliferative retinopathy due to diabetes mellitus type 1 | Clinical Finding | Condition | SNOMED |
| 44787902 | Lipoatrophic diabetes mellitus without complication | Clinical Finding | Condition | SNOMED |
| 443727 | Diabetic ketoacidosis | Clinical Finding | Condition | SNOMED |
| 4221962 | Very severe nonproliferative retinopathy without macular edema due to diabetes mellitus | Clinical Finding | Condition | SNOMED |
| 37116379 | Stimmler syndrome | Clinical Finding | Condition | SNOMED |
| 35626039 | Mild nonproliferative retinopathy of left eye due to diabetes mellitus | Clinical Finding | Condition | SNOMED |
| 4327944 | Malnutrition related diabetes mellitus | Clinical Finding | Condition | SNOMED |
| 4227210 | Retinopathy due to type 1 diabetes mellitus | Clinical Finding | Condition | SNOMED |
| 4161671 | Non-high-risk proliferative retinopathy with no macular edema due to diabetes mellitus | Clinical Finding | Condition | SNOMED |
| 45763584 | Proliferative retinopathy due to type 1 diabetes mellitus | Clinical Finding | Condition | SNOMED |
| 43530690 | Foot ulcer due to type 2 diabetes mellitus | Clinical Finding | Condition | SNOMED |
| 37018728 | Gastroparesis due to type 2 diabetes mellitus | Clinical Finding | Condition | SNOMED |
| 4102176 | Iritis due to diabetes mellitus | Clinical Finding | Condition | SNOMED |
| 4034963 | Megaloblastic anemia, thiamine-responsive, with diabetes mellitus and sensorineural deafness | Clinical Finding | Condition | SNOMED |
| 46274058 | Skin ulcer of toe due to diabetes mellitus type 2 | Clinical Finding | Condition | SNOMED |
| 4224709 | Multiple complications due to type 1 diabetes mellitus | Clinical Finding | Condition | SNOMED |
| 45770831 | Proliferative retinopathy with retinal edema due to type 2 diabetes mellitus | Clinical Finding | Condition | SNOMED |
| 4034964 | Abnormal metabolic state due to diabetes mellitus | Clinical Finding | Condition | SNOMED |
| 45757266 | Retinal edema due to type 1 diabetes mellitus | Clinical Finding | Condition | SNOMED |
| 4237068 | Diabetes mellitus due to structurally abnormal insulin | Clinical Finding | Condition | SNOMED |
| 43531015 | Maturity-onset diabetes of the young, type 7 | Clinical Finding | Condition | SNOMED |
| 3192955 | Poorly controlled type I diabetes with neuropathy | Clinical Finding | Condition | Nebraska Lexicon |
| 3172958 | Diabetic ulcer of right wrist | Clinical Finding | Condition | Nebraska Lexicon |
| 442793 | Complication due to diabetes mellitus | Clinical Finding | Condition | SNOMED |
| 43531566 | Chronic kidney disease stage 2 due to type 2 diabetes mellitus | Clinical Finding | Condition | SNOMED |
| 4044392 | Chronic painful polyneuropathy due to diabetes mellitus | Clinical Finding | Condition | SNOMED |
| 43531645 | Diabetes mellitus, transient neonatal 1 | Clinical Finding | Condition | SNOMED |
| 45763583 | Nonproliferative diabetic retinopathy due to type 1 diabetes mellitus | Clinical Finding | Condition | SNOMED |
| 43531577 | Chronic kidney disease stage 4 due to type 2 diabetes mellitus | Clinical Finding | Condition | SNOMED |
| 4223734 | Non-ketotic non-hyperosmolar coma due to diabetes mellitus | Clinical Finding | Condition | SNOMED |
| 4082360 | Hypohidrosis-diabetes insipidus syndrome | Clinical Finding | Condition | SNOMED |
| 43531597 | Disorder due to well controlled type 2 diabetes mellitus | Clinical Finding | Condition | SNOMED |
| 4152858 | Type 1 diabetes mellitus with arthropathy | Clinical Finding | Condition | SNOMED |
| 45757435 | Mild nonproliferative retinopathy due to type 2 diabetes mellitus | Clinical Finding | Condition | SNOMED |
| 42535539 | Disorder of nerve co-occurrent and due to type 1 diabetes mellitus | Clinical Finding | Condition | SNOMED |
| 4290823 | Very severe nonproliferative retinopathy with clinically significant macular edema due to diabetes mellitus | Clinical Finding | Condition | SNOMED |
| 40482801 | Type II diabetes mellitus uncontrolled | Clinical Finding | Condition | SNOMED |
| 4226798 | Hypoglycemic coma due to diabetes mellitus | Clinical Finding | Condition | SNOMED |
| 4095288 | Ketoacidotic coma due to diabetes mellitus | Clinical Finding | Condition | SNOMED |
| 43531009 | Pregnancy and type 1 diabetes mellitus | Clinical Finding | Condition | SNOMED |
| 45769830 | Neuropathic arthropathy due to type 1 diabetes mellitus | Clinical Finding | Condition | SNOMED |
| 44792134 | Maternally inherited diabetes mellitus | Clinical Finding | Condition | SNOMED |
| 4114427 | Neuropathic arthropathy due to diabetes mellitus | Clinical Finding | Condition | SNOMED |
| 44793114 | Pre-existing diabetes mellitus | Clinical Finding | Condition | SNOMED |
| 45757363 | Hypoglycemia due to type 2 diabetes mellitus | Clinical Finding | Condition | SNOMED |
| 37016180 | Moderate nonproliferative retinopathy due to type 1 diabetes mellitus | Clinical Finding | Condition | SNOMED |
| 443732 | Disorder due to type 2 diabetes mellitus | Clinical Finding | Condition | SNOMED |
| 4099653 | Steroid-induced diabetes mellitus without complication | Clinical Finding | Condition | SNOMED |
| 45766051 | Type I diabetes mellitus in remission | Clinical Finding | Condition | SNOMED |
| 45769902 | Chronic kidney disease stage 4 due to type 1 diabetes mellitus | Clinical Finding | Condition | SNOMED |
| 376065 | Disorder of nervous system due to type 2 diabetes mellitus | Clinical Finding | Condition | SNOMED |
| 44789319 | Hypoglycaemic warning good | Clinical Finding | Condition | SNOMED |
| 45757075 | Ischemia of retina due to type 2 diabetes mellitus | Clinical Finding | Condition | SNOMED |
| 37016357 | Non-proliferative retinopathy due to secondary diabetes mellitus | Clinical Finding | Condition | SNOMED |
| 37017432 | Polyneuropathy due to type 2 diabetes mellitus | Clinical Finding | Condition | SNOMED |
| 4159742 | Diabetic foot ulcer | Clinical Finding | Condition | SNOMED |
| 4210874 | Ischemic maculopathy due to diabetes mellitus | Clinical Finding | Condition | SNOMED |
| 4128221 | Microalbuminuric diabetic nephropathy | Clinical Finding | Condition | SNOMED |
| 4044393 | Asymmetric polyneuropathy due to diabetes mellitus | Clinical Finding | Condition | SNOMED |
| 4265913 | Pretibial pigmental patches due to diabetes mellitus | Clinical Finding | Condition | SNOMED |
| 4227657 | Skin ulcer due to diabetes mellitus | Clinical Finding | Condition | SNOMED |
| 45773576 | Chronic kidney disease stage 1 due to type 1 diabetes mellitus | Clinical Finding | Condition | SNOMED |
| 43531016 | Maturity-onset diabetes of the young, type 9 | Clinical Finding | Condition | SNOMED |
| 443735 | Coma due to diabetes mellitus | Clinical Finding | Condition | SNOMED |
| 4266041 | Visually threatening diabetic retinopathy | Clinical Finding | Condition | SNOMED |
| 4266637 | Severe nonproliferative retinopathy without macular edema due to diabetes mellitus | Clinical Finding | Condition | SNOMED |
| 36674766 | Venous beading of left retina due to diabetes mellitus | Clinical Finding | Condition | SNOMED |
| 37018566 | Peripheral neuropathy due to type 1 diabetes mellitus | Clinical Finding | Condition | SNOMED |
| 4033942 | Diabetic dermopathy | Clinical Finding | Condition | SNOMED |
| 45769875 | Insulin reactive hypoglycemia due to type 2 diabetes mellitus | Clinical Finding | Condition | SNOMED |
| 4255401 | O/E - right eye proliferative diabetic retinopathy | Clinical Finding | Condition | SNOMED |
| 35626762 | Cranial nerve palsy due to diabetes mellitus | Clinical Finding | Condition | SNOMED |
| 4130164 | Maturity onset diabetes of the young, type 2 | Clinical Finding | Condition | SNOMED |
| 43531641 | Permanent neonatal diabetes mellitus | Clinical Finding | Condition | SNOMED |
| 45769891 | Ankle ulcer due to type 1 diabetes mellitus | Clinical Finding | Condition | SNOMED |
| 45757065 | Proliferative retinopathy following surgery due to diabetes mellitus | Clinical Finding | Condition | SNOMED |
| 45769905 | Microalbuminuria due to type 2 diabetes mellitus | Clinical Finding | Condition | SNOMED |
| 3178971 | Diabetic ulcer of left great toe | Clinical Finding | Condition | Nebraska Lexicon |
| 37312198 | Sensory polyneuropathy due to diabetes mellitus | Clinical Finding | Condition | SNOMED |
| 45770986 | Retinopathy due to unstable diabetes mellitus type 1 | Clinical Finding | Condition | SNOMED |
| 45769892 | Ulcer of forefoot due to type 1 diabetes mellitus | Clinical Finding | Condition | SNOMED |
| 438476 | Nephrogenic diabetes insipidus | Clinical Finding | Condition | SNOMED |
| 37311833 | Stable treated proliferative retinopathy of right eye due to diabetes mellitus | Clinical Finding | Condition | SNOMED |
| 40482458 | Macroalbuminuric nephropathy due to diabetes mellitus | Clinical Finding | Condition | SNOMED |
| 45769834 | Osteomyelitis due to type 1 diabetes mellitus | Clinical Finding | Condition | SNOMED |
| 37312207 | Ulcer of heel due to diabetes mellitus | Clinical Finding | Condition | SNOMED |
| 4143529 | Diabetes mellitus associated with cystic fibrosis | Clinical Finding | Condition | SNOMED |
| 4177050 | Erectile dysfunction due to type 2 diabetes mellitus | Clinical Finding | Condition | SNOMED |
| 37204232 | Primary microcephaly, mild intellectual disability, young-onset diabetes syndrome | Clinical Finding | Condition | SNOMED |
| 4140808 | Rabson-Mendenhall syndrome | Clinical Finding | Condition | SNOMED |
| 201530 | Hyperosmolar coma due to type 2 diabetes mellitus | Clinical Finding | Condition | SNOMED |
| 45757674 | Diabetes mellitus type 1 without retinopathy | Clinical Finding | Condition | SNOMED |
| 443730 | Disorder of nervous system due to diabetes mellitus | Clinical Finding | Condition | SNOMED |
| 37311253 | Nonproliferative retinopathy of right eye due to diabetes mellitus | Clinical Finding | Condition | SNOMED |
| 4136889 | Diabetes mellitus induced by non-steroid drugs without complication | Clinical Finding | Condition | SNOMED |
| 43531010 | Pre-existing type 2 diabetes mellitus in pregnancy | Clinical Finding | Condition | SNOMED |
| 761051 | Complication due to secondary diabetes mellitus | Clinical Finding | Condition | SNOMED |
| 761062 | Ischemic heel and/or midfoot ulcer due to type 2 diabetes mellitus | Clinical Finding | Condition | SNOMED |
| 36712686 | Ulcer of left foot due to type 2 diabetes mellitus | Clinical Finding | Condition | SNOMED |
| 44793113 | Diabetes mellitus with multiple complications | Clinical Finding | Condition | SNOMED |
| 4131908 | Peripheral angiopathy due to diabetes mellitus | Clinical Finding | Condition | SNOMED |
| 42537681 | Diabetes mellitus caused by chemical | Clinical Finding | Condition | SNOMED |
| 4226354 | Gangrene due to diabetes mellitus | Clinical Finding | Condition | SNOMED |
| 4221933 | Coma due to malnutrition-related diabetes mellitus | Clinical Finding | Condition | SNOMED |
| 4129519 | Pregnancy and type 2 diabetes mellitus | Clinical Finding | Condition | SNOMED |
| 443012 | Diabetes mellitus during pregnancy - baby delivered | Clinical Finding | Condition | SNOMED |
| 4129225 | Motor polyneuropathy due to diabetes mellitus | Clinical Finding | Condition | SNOMED |
| 45770832 | Stasis ulcer due to type 2 diabetes mellitus | Clinical Finding | Condition | SNOMED |
| 37018765 | Gestational diabetes mellitus complicating pregnancy | Clinical Finding | Condition | SNOMED |
| 4199039 | Diffuse exudative maculopathy due to diabetes mellitus | Clinical Finding | Condition | SNOMED |
| 43530656 | Nonproliferative retinopathy due to type 2 diabetes mellitus | Clinical Finding | Condition | SNOMED |
| 4216968 | Acquired nephrogenic diabetes insipidus | Clinical Finding | Condition | SNOMED |
| 36712687 | Ulcer of right foot due to type 2 diabetes mellitus | Clinical Finding | Condition | SNOMED |
| 45772019 | Glaucoma due to type 2 diabetes mellitus | Clinical Finding | Condition | SNOMED |
| 4099216 | Multiple complications due to type 2 diabetes mellitus | Clinical Finding | Condition | SNOMED |
| 201254 | Type 1 diabetes mellitus | Clinical Finding | Condition | SNOMED |
| 4099215 | Type 1 diabetes mellitus maturity onset | Clinical Finding | Condition | SNOMED |
| 4096042 | Malnutrition-related diabetes mellitus without complications | Clinical Finding | Condition | SNOMED |
| 42536400 | Absence of lower limb due to diabetes mellitus | Clinical Finding | Condition | SNOMED |
| 37311329 | Woodhouse Sakati syndrome | Clinical Finding | Condition | SNOMED |
| 45771064 | Hypertension in chronic kidney disease due to type 2 diabetes mellitus | Clinical Finding | Condition | SNOMED |
| 4326434 | Gestational diabetes mellitus, class A>1< | Clinical Finding | Condition | SNOMED |
| 4137220 | Diabetic glomerulonephritis | Clinical Finding | Condition | SNOMED |
| 43531643 | Maturity-onset diabetes of the young, type 6 | Clinical Finding | Condition | SNOMED |
| 4066936 | Mixed diabetic ulcer - foot | Clinical Finding | Condition | SNOMED |
| 443412 | Type 1 diabetes mellitus without complication | Clinical Finding | Condition | SNOMED |
| 4143857 | Lumbosacral radiculoplexus neuropathy due to type 1 diabetes mellitus | Clinical Finding | Condition | SNOMED |
| 380097 | Macular edema due to diabetes mellitus | Clinical Finding | Condition | SNOMED |
| 4235260 | O/E - left eye background diabetic retinopathy | Clinical Finding | Condition | SNOMED |
| 4336000 | Proliferative retinopathy with neovascularization elsewhere than the optic disc due to diabetes mellitus | Clinical Finding | Condition | SNOMED |
| 30968 | Diabetes insipidus | Clinical Finding | Condition | SNOMED |
| 4169240 | Bullosis diabeticorum | Clinical Finding | Condition | SNOMED |
| 35626904 | Retinal edema due to diabetes mellitus | Clinical Finding | Condition | SNOMED |
| 36716853 | Renal papillary necrosis due to diabetes mellitus | Clinical Finding | Condition | SNOMED |
| 43531651 | Mixed hyperlipidemia due to type 2 diabetes mellitus | Clinical Finding | Condition | SNOMED |
| 45757447 | Hypertension in chronic kidney disease stage 2 due to type 2 diabetes mellitus | Clinical Finding | Condition | SNOMED |
| 4142579 | Small vessel disease due to type 2 diabetes mellitus | Clinical Finding | Condition | SNOMED |
| 45757507 | Ulcer of foot due to type 1 diabetes mellitus | Clinical Finding | Condition | SNOMED |
| 37312019 | Diabetic cardiomyopathy | Clinical Finding | Condition | SNOMED |
| 3180411 | Preulcerative calluses | Clinical Finding | Condition | Nebraska Lexicon |
| 4193704 | Type 2 diabetes mellitus without complication | Clinical Finding | Condition | SNOMED |
| 4221532 | Dipsogenic diabetes insipidus | Clinical Finding | Condition | SNOMED |
| 45769829 | Nephrotic syndrome due to type 1 diabetes mellitus | Clinical Finding | Condition | SNOMED |
| 3194119 | Poorly controlled type 1 diabetes | Clinical Finding | Condition | Nebraska Lexicon |
| 45769904 | End stage renal disease on dialysis due to type 1 diabetes mellitus | Clinical Finding | Condition | SNOMED |
| 4102018 | Insulin dependent diabetes mellitus type 1B | Clinical Finding | Condition | SNOMED |
| 35626042 | Moderate nonproliferative diabetic retinopathy of left eye | Clinical Finding | Condition | SNOMED |
| 35625717 | Cataract of right eye due to diabetes mellitus | Clinical Finding | Condition | SNOMED |
| 43530685 | Proliferative retinopathy due to type 2 diabetes mellitus | Clinical Finding | Condition | SNOMED |
| 4099334 | Steroid-induced diabetes | Clinical Finding | Condition | SNOMED |
| 4105639 | Chronic painful neuropathy due to diabetes mellitus | Clinical Finding | Condition | SNOMED |
| 376114 | Severe nonproliferative retinopathy due to diabetes mellitus | Clinical Finding | Condition | SNOMED |
| 4215719 | Hyperosmolar non-ketotic state due to type 2 diabetes mellitus | Clinical Finding | Condition | SNOMED |
| 4195498 | Mixed maculopathy due to diabetes mellitus | Clinical Finding | Condition | SNOMED |
| 4242528 | Diarrhea due to diabetes mellitus | Clinical Finding | Condition | SNOMED |
| 4164730 | Neurohypophyseal diabetes insipidus | Clinical Finding | Condition | SNOMED |
| 376683 | Nonproliferative retinopathy due to diabetes mellitus | Clinical Finding | Condition | SNOMED |
| 4252384 | Insulin autoimmune syndrome | Clinical Finding | Condition | SNOMED |
| 42536604 | Lactic acidosis due to diabetes mellitus | Clinical Finding | Condition | SNOMED |
| 37309630 | Coronary artery disease due to type 2 diabetes mellitus | Clinical Finding | Condition | SNOMED |
| 4171406 | Infection of foot due to diabetes mellitus | Clinical Finding | Condition | SNOMED |
| 37110041 | Permanent neonatal diabetes mellitus with cerebellar agenesis syndrome | Clinical Finding | Condition | SNOMED |
| 36717215 | Intellectual disability, craniofacial dysmorphism, hypogonadism, diabetes mellitus syndrome | Clinical Finding | Condition | SNOMED |
| 45773064 | Traction detachment of retina due to type 2 diabetes mellitus | Clinical Finding | Condition | SNOMED |
| 3192052 | Poorly controlled type I diabetes with renal complication | Clinical Finding | Condition | Nebraska Lexicon |
| 3194082 | Poorly controlled type II diabetes with complications | Clinical Finding | Condition | Nebraska Lexicon |
| 45772914 | Glomerulosclerosis of kidney due to diabetes mellitus | Clinical Finding | Condition | SNOMED |
| 4226121 | Retinopathy due to type 2 diabetes mellitus | Clinical Finding | Condition | SNOMED |
| 4131117 | Sensory neuropathy due to diabetes mellitus | Clinical Finding | Condition | SNOMED |
| 44805628 | Diabetic retinopathy detected by national screening programme | Clinical Finding | Condition | SNOMED |
| 4225656 | Cataract due to diabetes mellitus type 1 | Clinical Finding | Condition | SNOMED |
| 37016353 | Dyslipidemia due to type 1 diabetes mellitus | Clinical Finding | Condition | SNOMED |
| 45769835 | Severe malnutrition due to type 2 diabetes mellitus | Clinical Finding | Condition | SNOMED |
| 4334884 | Disorder of macula due to diabetes mellitus | Clinical Finding | Condition | SNOMED |
| 43531559 | Chronic kidney disease stage 1 due to type 2 diabetes mellitus | Clinical Finding | Condition | SNOMED |
| 45757077 | Diabetes mellitus due to pancreatic injury | Clinical Finding | Condition | SNOMED |
| 37016768 | Autonomic neuropathy due to type 2 diabetes mellitus | Clinical Finding | Condition | SNOMED |
| 35626764 | Clinically significant macular edema due to diabetes mellitus | Clinical Finding | Condition | SNOMED |
| 46269764 | Skin ulcer of toe due to diabetes mellitus type 1 | Clinical Finding | Condition | SNOMED |
| 4029420 | Severe hyperglycemia due to diabetes mellitus | Clinical Finding | Condition | SNOMED |
| 36714116 | Hypoglycemic coma due to type 2 diabetes mellitus | Clinical Finding | Condition | SNOMED |
| 760977 | Peripheral vascular disease due to secondary diabetes mellitus | Clinical Finding | Condition | SNOMED |
| 43531578 | Chronic kidney disease due to type 2 diabetes mellitus | Clinical Finding | Condition | SNOMED |
| 4202383 | Drug-induced diabetes mellitus | Clinical Finding | Condition | SNOMED |
| 45757535 | Microalbuminuria due to type 1 diabetes mellitus | Clinical Finding | Condition | SNOMED |
| 377552 | Moderate nonproliferative retinopathy due to diabetes mellitus | Clinical Finding | Condition | SNOMED |
| 4196141 | Arthropathy due to type 2 diabetes mellitus | Clinical Finding | Condition | SNOMED |
| 3656195 | Non centrally involved macular edema due to diabetes mellitus | Clinical Finding | Condition | SNOMED |
| 4265337 | Hereditary nephrogenic diabetes insipidus | Clinical Finding | Condition | SNOMED |
| 439770 | Ketoacidosis due to type 1 diabetes mellitus | Clinical Finding | Condition | SNOMED |
| 3191208 | Poorly controlled type II diabetes with neuropathy | Clinical Finding | Condition | Nebraska Lexicon |
| 376979 | Cataract due to diabetes mellitus | Clinical Finding | Condition | SNOMED |
| 4269871 | Very severe nonproliferative retinopathy due to diabetes mellitus | Clinical Finding | Condition | SNOMED |
| 37311673 | Hyperglycemia due to diabetes mellitus | Clinical Finding | Condition | SNOMED |
| 4058243 | Diabetes mellitus during pregnancy, childbirth and the puerperium | Clinical Finding | Condition | SNOMED |
| 45757362 | Hypoglycemic unawareness due to type 1 diabetes mellitus | Clinical Finding | Condition | SNOMED |
| 3193274 | Poorly controlled type 2 diabetes | Clinical Finding | Condition | Nebraska Lexicon |
| 36674200 | Proliferative retinopathy of left eye due to diabetes mellitus | Clinical Finding | Condition | SNOMED |
| 4099652 | Multiple complications due to malnutrition related diabetes | Clinical Finding | Condition | SNOMED |
| 4226238 | Hyperosmolar coma due to diabetes mellitus | Clinical Finding | Condition | SNOMED |
| 4024659 | Gestational diabetes mellitus | Clinical Finding | Condition | SNOMED |
| 45769837 | Chronic ulcer of skin due to type 1 diabetes mellitus | Clinical Finding | Condition | SNOMED |
| 4215961 | O/E - left eye stable treated proliferative diabetic retinopathy | Clinical Finding | Condition | SNOMED |
| 35625718 | Cataract of left eye due to diabetes mellitus | Clinical Finding | Condition | SNOMED |
| 4147504 | Myasthenic syndrome due to diabetic amyotrophy | Clinical Finding | Condition | SNOMED |
| 35626043 | Severe nonproliferative retinopathy of right eye due to diabetes mellitus | Clinical Finding | Condition | SNOMED |
| 4247107 | O/E - right eye background diabetic retinopathy | Clinical Finding | Condition | SNOMED |
| 4027121 | Nephrotic syndrome due to diabetes mellitus | Clinical Finding | Condition | SNOMED |
| 193323 | Neonatal diabetes mellitus | Clinical Finding | Condition | SNOMED |
| 43531565 | Erectile dysfunction due to type 1 diabetes mellitus | Clinical Finding | Condition | SNOMED |
| 37016354 | Neuropathy due to type 2 diabetes mellitus | Clinical Finding | Condition | SNOMED |
| 200687 | Renal disorder due to type 1 diabetes mellitus | Clinical Finding | Condition | SNOMED |
| 4338900 | Proliferative retinopathy with optic disc neovascularization due to diabetes mellitus | Clinical Finding | Condition | SNOMED |
| 4030061 | Malnutrition-related diabetes mellitus - fibrocalculous | Clinical Finding | Condition | SNOMED |
| 4082346 | Disorder of soft tissue due to diabetes mellitus | Clinical Finding | Condition | SNOMED |
| 4206115 | Glomerulopathy due to diabetes mellitus | Clinical Finding | Condition | SNOMED |
| 45757604 | Proteinuria due to type 1 diabetes mellitus | Clinical Finding | Condition | SNOMED |
| 435216 | Disorder due to type 1 diabetes mellitus | Clinical Finding | Condition | SNOMED |
| 4087682 | Diabetic foot | Clinical Finding | Condition | SNOMED |
| 194700 | Diabetes mellitus in mother complicating pregnancy, childbirth AND/OR puerperium | Clinical Finding | Condition | SNOMED |
| 4023792 | Pseudotabes due to diabetes mellitus | Clinical Finding | Condition | SNOMED |
| 3183485 | Diabetic ulcer right great toe | Clinical Finding | Condition | Nebraska Lexicon |
| 4223739 | Persistent proteinuria due to type 2 diabetes mellitus | Clinical Finding | Condition | SNOMED |
| 195771 | Secondary diabetes mellitus | Clinical Finding | Condition | SNOMED |
| 4245270 | Diabetes mellitus associated with genetic syndrome | Clinical Finding | Condition | SNOMED |
| 45769894 | Cranial nerve palsy due to type 2 diabetes mellitus | Clinical Finding | Condition | SNOMED |
| 4195043 | High risk proliferative retinopathy due to diabetes mellitus | Clinical Finding | Condition | SNOMED |
| 43531642 | Diabetes mellitus due to genetic defect in insulin action | Clinical Finding | Condition | SNOMED |
| 4222553 | Persistent proteinuria due to type 1 diabetes mellitus | Clinical Finding | Condition | SNOMED |
| 4194970 | Asymmetric proximal motor neuropathy due to diabetes mellitus | Clinical Finding | Condition | SNOMED |
| 3655914 | Cystoid macular edema due to diabetes mellitus | Clinical Finding | Condition | SNOMED |
| 376112 | Polyneuropathy due to diabetes mellitus | Clinical Finding | Condition | SNOMED |
| 4101892 | Asymptomatic neuropathy due to diabetes mellitus | Clinical Finding | Condition | SNOMED |
| 321822 | Peripheral vascular disorder due to diabetes mellitus | Clinical Finding | Condition | SNOMED |
| 37396524 | Gingival disease co-occurrent with diabetes mellitus | Clinical Finding | Condition | SNOMED |
| 4290822 | Severe nonproliferative retinopathy with clinically significant macular edema due to diabetes mellitus | Clinical Finding | Condition | SNOMED |
| 760980 | Retinopathy due to secondary diabetes mellitus | Clinical Finding | Condition | SNOMED |
| 37312218 | Neuropathy due to type 1 diabetes mellitus | Clinical Finding | Condition | SNOMED |
| 4212441 | O/E - right eye stable treated proliferative diabetic retinopathy | Clinical Finding | Condition | SNOMED |
| 45769873 | Traction detachment of retina due to type 1 diabetes mellitus | Clinical Finding | Condition | SNOMED |
| 4143689 | Small vessel disease due to type 1 diabetes mellitus | Clinical Finding | Condition | SNOMED |
| 4307319 | Symmetric proximal motor neuropathy due to diabetes mellitus | Clinical Finding | Condition | SNOMED |
| 45770881 | Moderate nonproliferative retinopathy due to type 2 diabetes mellitus | Clinical Finding | Condition | SNOMED |
| 45757124 | Gestational diabetes mellitus in childbirth | Clinical Finding | Condition | SNOMED |
| 201820 | Diabetes mellitus | Clinical Finding | Condition | SNOMED |
| 4145827 | Latent autoimmune diabetes mellitus in adult | Clinical Finding | Condition | SNOMED |
| 3655269 | Glaucoma due to diabetes mellitus | Clinical Finding | Condition | SNOMED |
| 4129520 | Nocturnal hypoglycemia due to diabetes mellitus | Clinical Finding | Condition | SNOMED |
| 4147577 | Intracapillary glomerulosclerosis of kidney due to diabetes mellitus | Clinical Finding | Condition | SNOMED |
| 44809809 | Hypoglycaemic warning absent | Clinical Finding | Condition | SNOMED |
| 43531008 | Pre-existing type 1 diabetes mellitus in pregnancy | Clinical Finding | Condition | SNOMED |
| 4321756 | Acanthosis nigricans due to type 2 diabetes mellitus | Clinical Finding | Condition | SNOMED |
| 37116960 | Wolfram-like syndrome | Clinical Finding | Condition | SNOMED |
| 37110068 | Diabetic mastopathy | Clinical Finding | Condition | SNOMED |
| 37311453 | Armanni-Ebstein kidney due to diabetes mellitus | Clinical Finding | Condition | SNOMED |
| 43531020 | Diabetes mellitus, transient neonatal 3 | Clinical Finding | Condition | SNOMED |
| 35626087 | Disorder of right macula due to diabetes mellitus | Clinical Finding | Condition | SNOMED |
| 3655385 | Chronic ulcer of left foot due to diabetes mellitus | Clinical Finding | Condition | SNOMED |
| 4047906 | Insulin dependent diabetes mellitus type 1A | Clinical Finding | Condition | SNOMED |
| 45770830 | Macular edema and retinopathy due to type 2 diabetes mellitus | Clinical Finding | Condition | SNOMED |
| 35626037 | Preproliferative retinopathy of left eye due to diabetes mellitus | Clinical Finding | Condition | SNOMED |
| 4096041 | Malnutrition-related diabetes mellitus with ketoacidosis | Clinical Finding | Condition | SNOMED |
| 4062686 | Diabetes mellitus in the puerperium - baby delivered during previous episode of care | Clinical Finding | Condition | SNOMED |
| 45771067 | Hypertension in chronic kidney disease due to type 1 diabetes mellitus | Clinical Finding | Condition | SNOMED |
| 37312205 | Ulcer of heel due to type 2 diabetes mellitus | Clinical Finding | Condition | SNOMED |
| 37017431 | Polyneuropathy due to type 1 diabetes mellitus | Clinical Finding | Condition | SNOMED |
| 43531562 | Chronic kidney disease stage 5 due to type 2 diabetes mellitus | Clinical Finding | Condition | SNOMED |
| 37016348 | Hyperglycemia due to type 1 diabetes mellitus | Clinical Finding | Condition | SNOMED |
| 37312200 | Ulcer of midfoot due to type 1 diabetes mellitus | Clinical Finding | Condition | SNOMED |
| 4008576 | Diabetes mellitus without complication | Clinical Finding | Condition | SNOMED |
| 45770880 | Hyperlipidemia due to type 2 diabetes mellitus | Clinical Finding | Condition | SNOMED |
| 40484648 | Type 1 diabetes mellitus uncontrolled | Clinical Finding | Condition | SNOMED |
| 4129524 | Insulin resistance - type A | Clinical Finding | Condition | SNOMED |
| 4307799 | Anemia due to diabetes mellitus | Clinical Finding | Condition | SNOMED |
| 37204277 | Primary microcephaly, epilepsy, permanent neonatal diabetes syndrome | Clinical Finding | Condition | SNOMED |
| 3655382 | Ulcer of right foot due to diabetes mellitus | Clinical Finding | Condition | SNOMED |
| 35625719 | Diabetic cataract of bilateral eyes | Clinical Finding | Condition | SNOMED |
| 35626069 | Macular edema due to type 1 diabetes mellitus | Clinical Finding | Condition | SNOMED |
| 3655948 | Persistent macular edema due to diabetes mellitus | Clinical Finding | Condition | SNOMED |
| 37016349 | Hyperglycemia due to type 2 diabetes mellitus | Clinical Finding | Condition | SNOMED |
| 380096 | Proliferative retinopathy due to diabetes mellitus | Clinical Finding | Condition | SNOMED |
| 3183244 | Gestational diabetes during pregnancy | Clinical Finding | Condition | Nebraska Lexicon |
| 35626071 | Clinically significant macular edema of right eye due to diabetes mellitus | Clinical Finding | Condition | SNOMED |
| 4048202 | Houssay's syndrome | Clinical Finding | Condition | SNOMED |
| 37017429 | Gastroparesis due to type 1 diabetes mellitus | Clinical Finding | Condition | SNOMED |
| 45757277 | Ulcer of lower limb due to type 2 diabetes mellitus | Clinical Finding | Condition | SNOMED |
| 4222415 | Mononeuropathy due to type 2 diabetes mellitus | Clinical Finding | Condition | SNOMED |
| 4311708 | Diabetic peripheral neuropathy | Clinical Finding | Condition | SNOMED |
| 37018912 | Diabetic hand syndrome due to type 2 diabetes mellitus | Clinical Finding | Condition | SNOMED |
| 36674652 | Vitreous hemorrhage of left eye due to diabetes mellitus | Clinical Finding | Condition | SNOMED |
| 45771072 | Ischemic ulcer of foot due to type 2 diabetes mellitus | Clinical Finding | Condition | SNOMED |
| 4161670 | Venous beading of retina due to diabetes mellitus | Clinical Finding | Condition | SNOMED |
| 4063043 | Pre-existing type 2 diabetes mellitus | Clinical Finding | Condition | SNOMED |
| 45773567 | Sensory neuropathy due to type 1 diabetes mellitus | Clinical Finding | Condition | SNOMED |
| 36674651 | Vitreous hemorrhage of right eye due to diabetes mellitus | Clinical Finding | Condition | SNOMED |
| 42536603 | Hyperosmolar hyperglycemic coma due to diabetes mellitus without ketoacidosis | Clinical Finding | Condition | SNOMED |
| 45757449 | Ulcer of toe due to type 2 diabetes mellitus | Clinical Finding | Condition | SNOMED |
| 45772060 | Hypoglycemia unawareness due to type 2 diabetes mellitus | Clinical Finding | Condition | SNOMED |
| 3198118 | Poorly controlled diabetes mellitus | Clinical Finding | Condition | Nebraska Lexicon |
| 4129378 | Transient neonatal diabetes mellitus | Clinical Finding | Condition | SNOMED |
| 4304701 | Mononeuropathy simplex due to diabetes mellitus | Clinical Finding | Condition | SNOMED |
| 4164175 | Diabetic intraretinal microvascular anomaly | Clinical Finding | Condition | SNOMED |
| 4034962 | Hyperproinsulinemia | Clinical Finding | Condition | SNOMED |
| 4263902 | Gestational diabetes mellitus, class A>2< | Clinical Finding | Condition | SNOMED |
| 4230254 | Type 2 diabetes mellitus in nonobese | Clinical Finding | Condition | SNOMED |
| 45769903 | Chronic kidney disease stage 5 due to type 1 diabetes mellitus | Clinical Finding | Condition | SNOMED |
| 4224879 | Disorder of nervous system due to malnutrition related diabetes mellitus | Clinical Finding | Condition | SNOMED |
| 36685758 | Ketosis-prone diabetes mellitus | Clinical Finding | Condition | SNOMED |
| 3655913 | Severe nonproliferative diabetic retinopathy with venous beading | Clinical Finding | Condition | SNOMED |
| 4105173 | Advanced maculopathy due to diabetes mellitus | Clinical Finding | Condition | SNOMED |
| 36674765 | Venous beading of right retina due to diabetes mellitus | Clinical Finding | Condition | SNOMED |
| 36715051 | Atherosclerosis, deafness, diabetes, epilepsy, nephropathy syndrome | Clinical Finding | Condition | SNOMED |
| 765478 | Diabetes mellitus caused by drug without complication | Clinical Finding | Condition | SNOMED |
| 36712670 | Skin ulcer due to type 2 diabetes mellitus | Clinical Finding | Condition | SNOMED |
| 4061725 | Cellulitis of foot due to diabetes mellitus | Clinical Finding | Condition | SNOMED |
| 45769876 | Hypoglycemia due to type 1 diabetes mellitus | Clinical Finding | Condition | SNOMED |
| 4209538 | Advanced retinal disease due to diabetes mellitus | Clinical Finding | Condition | SNOMED |
| 45771533 | Rubeosis iridis due to type 1 diabetes mellitus | Clinical Finding | Condition | SNOMED |
| 4269870 | Non-high-risk proliferative retinopathy with clinically significant macular edema due to diabetes mellitus | Clinical Finding | Condition | SNOMED |
| 43530660 | Mixed hyperlipidemia due to type 1 diabetes mellitus | Clinical Finding | Condition | SNOMED |
| 40480000 | Multiple complications due to diabetes mellitus | Clinical Finding | Condition | SNOMED |
| 4129516 | Diabetes-deafness syndrome maternally transmitted | Clinical Finding | Condition | SNOMED |
| 4223463 | Exudative maculopathy due to type 2 diabetes mellitus | Clinical Finding | Condition | SNOMED |
| 36684827 | Diabetes mellitus type 2 with periodontal disease | Clinical Finding | Condition | SNOMED |
| 35626067 | Macular edema of right eye due to diabetes mellitus | Clinical Finding | Condition | SNOMED |
| 4027848 | Secondary diabetes insipidus | Clinical Finding | Condition | SNOMED |
| 45769888 | Ankle ulcer due to type 2 diabetes mellitus | Clinical Finding | Condition | SNOMED |
| 4054812 | Mixed sensorimotor polyneuropathy due to diabetes mellitus | Clinical Finding | Condition | SNOMED |
| 45769906 | End stage renal disease on dialysis due to type 2 diabetes mellitus | Clinical Finding | Condition | SNOMED |
| 43531608 | Vitreous hemorrhage due to type 2 diabetes mellitus | Clinical Finding | Condition | SNOMED |
| 3178281 | Diabetes mellitus complicating Cystic fibrosis | Clinical Finding | Condition | Nebraska Lexicon |
| 43531653 | Chronic kidney disease stage 3 due to type 2 diabetes mellitus | Clinical Finding | Condition | SNOMED |
| 43530689 | Peripheral neuropathy due to type 2 diabetes mellitus | Clinical Finding | Condition | SNOMED |
| 4029440 | Familial central diabetes insipidus | Clinical Finding | Condition | SNOMED |
| 4164176 | High risk proliferative retinopathy not amenable to photocoagulation due to diabetes mellitus | Clinical Finding | Condition | SNOMED |
| 4101887 | Mononeuritis multiplex with diabetes mellitus | Clinical Finding | Condition | SNOMED |
| 36674199 | Proliferative retinopathy of right eye with diabetes mellitus | Clinical Finding | Condition | SNOMED |
| 35626046 | Very severe nonproliferative retinopathy of right eye due to diabetes mellitus | Clinical Finding | Condition | SNOMED |
| 4164632 | Very severe proliferative retinopathy due to diabetes mellitus | Clinical Finding | Condition | SNOMED |
| 45769890 | Ulcer of forefoot due to type 2 diabetes mellitus | Clinical Finding | Condition | SNOMED |
| 4099741 | Fibrocalculous pancreatic diabetes | Clinical Finding | Condition | SNOMED |
| 36713094 | Peripheral angiopathy due to type 1 diabetes mellitus | Clinical Finding | Condition | SNOMED |
| 43531007 | Pre-existing diabetes mellitus in pregnancy | Clinical Finding | Condition | SNOMED |
| 35626072 | Clinically significant macular edema of left eye due to diabetes mellitus | Clinical Finding | Condition | SNOMED |
| 36716258 | Pancreatic hypoplasia, diabetes mellitus, congenital heart disease syndrome | Clinical Finding | Condition | SNOMED |
| 45769901 | Chronic kidney disease stage 2 due to type 1 diabetes mellitus | Clinical Finding | Condition | SNOMED |
| 4266042 | High risk proliferative retinopathy with clinically significant macula edema due to diabetes mellitus | Clinical Finding | Condition | SNOMED |
| 37017430 | Gastroparesis due to diabetes mellitus | Clinical Finding | Condition | SNOMED |
| 43531644 | Maturity-onset diabetes of the young, type 8 | Clinical Finding | Condition | SNOMED |
| 3656194 | Centrally involved macular edema due to diabetes mellitus | Clinical Finding | Condition | SNOMED |
| 35626044 | Severe nonproliferative retinopathy of left eye due to diabetes mellitus | Clinical Finding | Condition | SNOMED |
| 45757771 | Partial nephrogenic diabetes insipidus | Clinical Finding | Condition | SNOMED |
| 4030066 | Photomyoclonus, diabetes mellitus, deafness, nephropathy and cerebral dysfunction | Clinical Finding | Condition | SNOMED |
| 45757278 | Peripheral sensory neuropathy due to type 2 diabetes mellitus | Clinical Finding | Condition | SNOMED |
| 45757074 | Vitreous hemorrhage due to type 1 diabetes mellitus | Clinical Finding | Condition | SNOMED |
| 4084643 | Extreme insulin resistance with acanthosis nigricans, hirsutism AND abnormal insulin receptors | Clinical Finding | Condition | SNOMED |
| 45757129 | Diabetes mellitus in mother complicating childbirth | Clinical Finding | Condition | SNOMED |
| 36715417 | DEND syndrome | Clinical Finding | Condition | SNOMED |
| 35625722 | Iritis of right eye due to diabetes mellitus | Clinical Finding | Condition | SNOMED |
| 45757073 | Ischemia of retina due to type 1 diabetes mellitus | Clinical Finding | Condition | SNOMED |
| 4175440 | Autonomic neuropathy due to diabetes mellitus | Clinical Finding | Condition | SNOMED |
| 42538169 | Disorder of eye due to type 1 diabetes mellitus | Clinical Finding | Condition | SNOMED |
| 45757445 | Hypertension in chronic kidney disease stage 4 due to type 2 diabetes mellitus | Clinical Finding | Condition | SNOMED |
| 4221487 | Persistent microalbuminuria due to type 2 diabetes mellitus | Clinical Finding | Condition | SNOMED |
| 35626070 | Macular edema due to type 2 diabetes mellitus | Clinical Finding | Condition | SNOMED |
| 36674753 | Microaneurysm of left retinal artery due to diabetes mellitus | Clinical Finding | Condition | SNOMED |
| 4046332 | Diabetic acute painful polyneuropathy | Clinical Finding | Condition | SNOMED |
| 37109305 | Hyperosmolarity due to drug induced diabetes mellitus | Clinical Finding | Condition | SNOMED |
| 40480031 | Hyperglycemic crisis due to diabetes mellitus | Clinical Finding | Condition | SNOMED |
| 45769828 | Nephrotic syndrome due to type 2 diabetes mellitus | Clinical Finding | Condition | SNOMED |
| 45757446 | Hypertension in chronic kidney disease stage 3 due to type 2 diabetes mellitus | Clinical Finding | Condition | SNOMED |
| 761053 | Severe nonproliferative retinopathy due to diabetes mellitus type 2 | Clinical Finding | Condition | SNOMED |
| 4082348 | Rubeosis faciei due to diabetes mellitus | Clinical Finding | Condition | SNOMED |
| 45769836 | Osteomyelitis due to type 2 diabetes mellitus | Clinical Finding | Condition | SNOMED |
| 43531563 | Neuropathic arthropathy due to type 2 diabetes mellitus | Clinical Finding | Condition | SNOMED |
| 37016767 | Autonomic neuropathy due to type 1 diabetes mellitus | Clinical Finding | Condition | SNOMED |
| 4096671 | Malnutrition-related diabetes mellitus with peripheral circulatory complications | Clinical Finding | Condition | SNOMED |
| 3655235 | Disorder of macula of bilateral eyes due to diabetes mellitus | Clinical Finding | Condition | SNOMED |
| 4210128 | Non-high-risk proliferative retinopathy due to diabetes mellitus | Clinical Finding | Condition | SNOMED |
| 3655383 | Ulcer of left foot due to diabetes mellitus | Clinical Finding | Condition | SNOMED |
| 37016350 | Dermatitis due to drug induced diabetes mellitus | Clinical Finding | Condition | SNOMED |
| 4164174 | High risk proliferative retinopathy without macular edema due to diabetes mellitus | Clinical Finding | Condition | SNOMED |
| 3194332 | Poorly controlled type II diabetes with renal complications | Clinical Finding | Condition | Nebraska Lexicon |
| 45766050 | Diabetes mellitus in remission | Clinical Finding | Condition | SNOMED |
| 4105016 | Acute painful diabetic neuropathy | Clinical Finding | Condition | SNOMED |
| 4043348 | Drug-induced nephrogenic diabetes insipidus | Clinical Finding | Condition | SNOMED |
| 4234742 | Diabetic neuropathy with neurologic complication | Clinical Finding | Condition | SNOMED |
| 4063569 | Ischemic ulcer of foot due to diabetes mellitus | Clinical Finding | Condition | SNOMED |
| 4195045 | Vitreous hemorrhage due to diabetes mellitus | Clinical Finding | Condition | SNOMED |
| 4195044 | Proliferative retinopathy with iris neovascularization due to diabetes mellitus | Clinical Finding | Condition | SNOMED |
| 4221495 | Cataract due to diabetes mellitus type 2 | Clinical Finding | Condition | SNOMED |
| 46274096 | Gingivitis co-occurrent with diabetes mellitus | Clinical Finding | Condition | SNOMED |
| 4147719 | Hyperosmolar non-ketotic state due to diabetes mellitus | Clinical Finding | Condition | SNOMED |
| 35626088 | Disorder of left macula due to diabetes mellitus | Clinical Finding | Condition | SNOMED |
| 45763585 | Blindness due to type 1 diabetes mellitus | Clinical Finding | Condition | SNOMED |
| 42535540 | Hyperosmolarity due to type 1 diabetes mellitus | Clinical Finding | Condition | SNOMED |
| 761048 | Disorder of vision due to secondary diabetes mellitus | Clinical Finding | Condition | SNOMED |
| 44805212 | Symptomatic diabetic peripheral neuropathy | Clinical Finding | Condition | SNOMED |
| 35626036 | Preproliferative retinopathy of right eye due to diabetes mellitus | Clinical Finding | Condition | SNOMED |
| 192691 | Diabetes mellitus during pregnancy - baby not yet delivered | Clinical Finding | Condition | SNOMED |
| 37204818 | Myopathy and diabetes mellitus | Clinical Finding | Condition | SNOMED |
| 4048029 | Thoracic radiculopathy due to diabetes mellitus | Clinical Finding | Condition | SNOMED |
| 4099651 | Type 2 diabetes mellitus with ulcer | Clinical Finding | Condition | SNOMED |
| 4009303 | Diabetic ketoacidosis without coma | Clinical Finding | Condition | SNOMED |
| 45763617 | Partial diabetes insipidus | Clinical Finding | Condition | SNOMED |
| 4189418 | Radiculoplexus neuropathy due to diabetes mellitus | Clinical Finding | Condition | SNOMED |
| 37017221 | Neovascular glaucoma due to diabetes mellitus | Clinical Finding | Condition | SNOMED |
| 4151453 | Diabetic optic papillopathy | Clinical Finding | Condition | SNOMED |
| 45757499 | Proteinuria due to type 2 diabetes mellitus | Clinical Finding | Condition | SNOMED |
| 4162095 | Macular edema not clinically significant due to diabetes mellitus | Clinical Finding | Condition | SNOMED |
| 37016355 | Hyperosmolar coma due to secondary diabetes mellitus | Clinical Finding | Condition | SNOMED |
| 35626905 | Ischemia of retina due to diabetes mellitus | Clinical Finding | Condition | SNOMED |
| 45757444 | Hypertension in chronic kidney disease stage 5 due to type 2 diabetes mellitus | Clinical Finding | Condition | SNOMED |
| 4129525 | Insulin resistance - type B | Clinical Finding | Condition | SNOMED |
| 4029423 | Hypoglycemia due to diabetes mellitus | Clinical Finding | Condition | SNOMED |
| 4114426 | Diabetic hand syndrome | Clinical Finding | Condition | SNOMED |
| 43531014 | Maturity-onset diabetes of the young, type 5 | Clinical Finding | Condition | SNOMED |
| 35626068 | Macular edema of left eye due to diabetes mellitus | Clinical Finding | Condition | SNOMED |
| 4192852 | Diabetes mellitus due to insulin receptor antibodies | Clinical Finding | Condition | SNOMED |
| 4171246 | Familial diabetes insipidus | Clinical Finding | Condition | SNOMED |
| 35626047 | Very severe nonproliferative retinopathy of left eye due to diabetes mellitus | Clinical Finding | Condition | SNOMED |
| 4224254 | Ketoacidotic coma due to type 1 diabetes mellitus | Clinical Finding | Condition | SNOMED |
| 37312204 | Ulcer of midfoot due to diabetes mellitus | Clinical Finding | Condition | SNOMED |
| 4304377 | Type 2 diabetes mellitus in obese | Clinical Finding | Condition | SNOMED |
| 4151946 | Kimmelstiel-Wilson syndrome | Clinical Finding | Condition | SNOMED |
| 4099214 | Type 1 diabetes mellitus with ulcer | Clinical Finding | Condition | SNOMED |
| 43531640 | Maturity-onset diabetes of the young | Clinical Finding | Condition | SNOMED |
| 4030664 | Proteinuric nephropathy due to diabetes mellitus | Clinical Finding | Condition | SNOMED |
| 45757474 | Diabetes mellitus type 2 without retinopathy | Clinical Finding | Condition | SNOMED |
| 43531018 | Maturity-onset diabetes of the young, type 11 | Clinical Finding | Condition | SNOMED |
| 4222876 | Gangrene due to type 2 diabetes mellitus | Clinical Finding | Condition | SNOMED |
| 43531013 | Maturity-onset diabetes of the young, type 4 | Clinical Finding | Condition | SNOMED |
| 4101478 | Microaneurysm of retinal artery due to diabetes mellitus | Clinical Finding | Condition | SNOMED |
| 4096670 | Malnutrition-related diabetes mellitus with renal complications | Clinical Finding | Condition | SNOMED |
| 36713275 | Atypical diabetes mellitus | Clinical Finding | Condition | SNOMED |
| 35626041 | Moderate nonproliferative diabetic retinopathy of right eye | Clinical Finding | Condition | SNOMED |
| 4063042 | Pre-existing type 1 diabetes mellitus | Clinical Finding | Condition | SNOMED |
| 443733 | Disorder of eye due to type 2 diabetes mellitus | Clinical Finding | Condition | SNOMED |
| 36715571 | Acidosis due to type 1 diabetes mellitus | Clinical Finding | Condition | SNOMED |
| 4006979 | Leprechaunism syndrome | Clinical Finding | Condition | SNOMED |
| 443767 | Disorder of eye due to diabetes mellitus | Clinical Finding | Condition | SNOMED |
| 37110593 | Lesion of skin due to diabetes mellitus | Clinical Finding | Condition | SNOMED |
| 4240589 | Diabetes mellitus associated with hormonal etiology | Clinical Finding | Condition | SNOMED |
| 45770928 | Retinal edema due to type 2 diabetes mellitus | Clinical Finding | Condition | SNOMED |
| 4210129 | Quiescent proliferative retinopathy due to diabetes mellitus | Clinical Finding | Condition | SNOMED |
| 45757280 | Dyslipidemia with high density lipoprotein below reference range and triglyceride above reference range due to type 2 diabetes mellitus | Clinical Finding | Condition | SNOMED |
| 4225055 | Mononeuropathy due to type 1 diabetes mellitus | Clinical Finding | Condition | SNOMED |
| 45766052 | Type II diabetes mellitus in remission | Clinical Finding | Condition | SNOMED |
| 4338901 | Traction detachment of retina due to diabetes mellitus | Clinical Finding | Condition | SNOMED |
| 42538715 | Acute complication due to diabetes mellitus | Clinical Finding | Condition | SNOMED |
| 3196797 | Poorly controlled type I diabetes with complication | Clinical Finding | Condition | Nebraska Lexicon |
| 4210872 | Focal exudative maculopathy due to diabetes mellitus | Clinical Finding | Condition | SNOMED |
| 40482883 | Posttransplant diabetes mellitus | Clinical Finding | Condition | SNOMED |
| 3169474 | Diabetic hypoglycemia w anger outbursts | Clinical Finding | Condition | Nebraska Lexicon |
| 35626765 | Cranial nerve palsy due to type 1 diabetes mellitus | Clinical Finding | Condition | SNOMED |
| 4062687 | Pre-existing malnutrition-related diabetes mellitus | Clinical Finding | Condition | SNOMED |
| 4034960 | Secondary endocrine diabetes mellitus | Clinical Finding | Condition | SNOMED |
| 4176925 | Ophthalmoplegia due to diabetes mellitus | Clinical Finding | Condition | SNOMED |
| 43531564 | Dyslipidemia due to type 2 diabetes mellitus | Clinical Finding | Condition | SNOMED |
| 43531588 | Angina associated with type 2 diabetes mellitus | Clinical Finding | Condition | SNOMED |
| 3198350 | Poorly controlled type I diabetes with circulatory disorder | Clinical Finding | Condition | Nebraska Lexicon |
| 37311832 | Stable treated proliferative retinopathy of left eye due to diabetes mellitus | Clinical Finding | Condition | SNOMED |
| 42536605 | Metabolic acidosis due to diabetes mellitus | Clinical Finding | Condition | SNOMED |
| 443734 | Ketoacidosis due to type 2 diabetes mellitus | Clinical Finding | Condition | SNOMED |
| 45771075 | Chronic kidney disease stage 3 due to type 1 diabetes mellitus | Clinical Finding | Condition | SNOMED |
| 4218499 | O/E - sight threatening diabetic retinopathy | Clinical Finding | Condition | SNOMED |
| 37311254 | Nonproliferative retinopathy of left eye due to diabetes mellitus | Clinical Finding | Condition | SNOMED |
| 45770902 | Ulcer of lower limb due to type 1 diabetes mellitus | Clinical Finding | Condition | SNOMED |
| 4322638 | Diabetes mellitus AND insipidus with optic atrophy AND deafness | Clinical Finding | Condition | SNOMED |
| 45757789 | Postpartum gestational diabetes mellitus | Clinical Finding | Condition | SNOMED |
| 201531 | Hyperosmolar coma due to type 1 diabetes mellitus | Clinical Finding | Condition | SNOMED |
| 43531012 | Maturity-onset diabetes of the young, type 3 | Clinical Finding | Condition | SNOMED |
| 4105172 | Preproliferative retinopathy due to diabetes mellitus | Clinical Finding | Condition | SNOMED |
| 4243625 | Diffuse glomerulosclerosis of kidney due to diabetes mellitus | Clinical Finding | Condition | SNOMED |
| 4255399 | O/E - right eye preproliferative diabetic retinopathy | Clinical Finding | Condition | SNOMED |
| 43531017 | Maturity-onset diabetes of the young, type 10 | Clinical Finding | Condition | SNOMED |
| 443592 | Hyperosmolality due to uncontrolled type 1 diabetes mellitus | Clinical Finding | Condition | SNOMED |
| 3192767 | Poorly controlled type II diabetes with circulatory disease | Clinical Finding | Condition | Nebraska Lexicon |
| 45763582 | Blindness due to type 2 diabetes mellitus | Clinical Finding | Condition | SNOMED |
| 45757508 | Type 2 diabetes mellitus controlled by diet | Clinical Finding | Condition | SNOMED |
| 4263090 | Femoral mononeuropathy due to diabetes mellitus | Clinical Finding | Condition | SNOMED |
| 4228443 | Ketoacidotic coma due to type 2 diabetes mellitus | Clinical Finding | Condition | SNOMED |
| 443729 | Peripheral circulatory disorder due to type 2 diabetes mellitus | Clinical Finding | Condition | SNOMED |
| 37016358 | Moderate nonproliferative retinopathy due to secondary diabetes mellitus | Clinical Finding | Condition | SNOMED |
| 443731 | Renal disorder due to type 2 diabetes mellitus | Clinical Finding | Condition | SNOMED |
| 4082347 | Diabetic thick skin syndrome | Clinical Finding | Condition | SNOMED |
| 43531616 | Dermopathy due to type 2 diabetes mellitus | Clinical Finding | Condition | SNOMED |
| 192279 | Disorder of kidney due to diabetes mellitus | Clinical Finding | Condition | SNOMED |
| 37396398 | Schofer Beetz Bohl syndrome | Clinical Finding | Condition | SNOMED |
| 43531011 | Diabetes mellitus due to genetic defect in beta cell function | Clinical Finding | Condition | SNOMED |
| 378743 | Mild nonproliferative retinopathy due to diabetes mellitus | Clinical Finding | Condition | SNOMED |
| 37016179 | Mild nonproliferative retinopathy due to type 1 diabetes mellitus | Clinical Finding | Condition | SNOMED |
| 44789318 | Hypoglycaemic warning impaired | Clinical Finding | Condition | SNOMED |
| 4166381 | Maternal diabetes mellitus with hypoglycemia affecting fetus OR newborn | Clinical Finding | Condition | SNOMED |
| 4178790 | Diabetes mellitus associated with receptor abnormality | Clinical Finding | Condition | SNOMED |
| 45769832 | Dermopathy due to type 1 diabetes mellitus | Clinical Finding | Condition | SNOMED |
| 43531006 | Maturity onset diabetes of the young, type 1 | Clinical Finding | Condition | SNOMED |
| 4222687 | Persistent microalbuminuria due to type 1 diabetes mellitus | Clinical Finding | Condition | SNOMED |
| 4062685 | Diabetes mellitus in the puerperium - baby delivered during current episode of care | Clinical Finding | Condition | SNOMED |
| 45757079 | Pre-existing diabetes mellitus in mother complicating childbirth | Clinical Finding | Condition | SNOMED |
| 4221344 | Exudative maculopathy due to type 1 diabetes mellitus | Clinical Finding | Condition | SNOMED |
| 4130162 | Insulin treated type 2 diabetes mellitus | Clinical Finding | Condition | SNOMED |
| 4162239 | Somogyi phenomenon | Clinical Finding | Condition | SNOMED |
| 201826 | Type 2 diabetes mellitus | Clinical Finding | Condition | SNOMED |
| 4191611 | Lumbosacral radiculoplexus neuropathy due to diabetes mellitus | Clinical Finding | Condition | SNOMED |
| 35626761 | Cervical radiculoplexus neuropathy due to diabetes mellitus | Clinical Finding | Condition | SNOMED |
| 4029422 | Hypoglycemic event due to diabetes | Clinical Finding | Condition | SNOMED |
| 4212631 | Protein-deficient diabetes mellitus | Clinical Finding | Condition | SNOMED |
| 36674752 | Microaneurysm of right retinal artery due to diabetes mellitus | Clinical Finding | Condition | SNOMED |
| 4255400 | O/E - left eye preproliferative diabetic retinopathy | Clinical Finding | Condition | SNOMED |
| 4140466 | Lumbosacral radiculoplexus neuropathy due to type 2 diabetes mellitus | Clinical Finding | Condition | SNOMED |
| 4223303 | Gangrene due to type 1 diabetes mellitus | Clinical Finding | Condition | SNOMED |
| 4048028 | Diabetic mononeuropathy | Clinical Finding | Condition | SNOMED |
| 45769833 | Severe malnutrition due to type 1 diabetes mellitus | Clinical Finding | Condition | SNOMED |
| 36717156 | Acidosis due to type 2 diabetes mellitus | Clinical Finding | Condition | SNOMED |
| 4235410 | Diabetes mellitus induced by non-steroid drugs | Clinical Finding | Condition | SNOMED |
| 4262282 | Diabetic mononeuropathy multiplex | Clinical Finding | Condition | SNOMED |
| 3655384 | Chronic ulcer of right foot due to diabetes mellitus | Clinical Finding | Condition | SNOMED |
| 35625723 | Iritis of left eye due to diabetes mellitus | Clinical Finding | Condition | SNOMED |
| 40485020 | Type 2 diabetes mellitus well controlled | Clinical Finding | Condition | SNOMED |
| 45769872 | Rubeosis iridis due to type 2 diabetes mellitus | Clinical Finding | Condition | SNOMED |
| 37312201 | Ulcer of heel due to type 1 diabetes mellitus | Clinical Finding | Condition | SNOMED |
| 37115758 | X-linked intellectual disability, limb spasticity, retinal dystrophy, diabetes insipidus syndrome | Clinical Finding | Condition | SNOMED |
| 35626763 | Erectile dysfunction due to diabetes mellitus | Clinical Finding | Condition | SNOMED |

Table 4. Chronic obstructive pulmonary disease (COPD).

| **Concept ID** | **Concept Name** | **Class** | **Domain** | **Vocabulary** | |
| --- | --- | --- | --- | --- | --- |
| 3105906 | Acute exacerbation of chronic obstructive airways disease | Clinical Finding | Condition | Nebraska Lexicon |
| 35208022 | Emphysema, unspecified | 4-char billing code | Condition | ICD10CM |
| 4050961 | Giant bullous emphysema | Clinical Finding | Condition | SNOMED |
| 4056405 | Obstructive emphysema | Clinical Finding | Condition | SNOMED |
| 4209097 | Severe chronic obstructive pulmonary disease | Clinical Finding | Condition | SNOMED |
| 44820889 | Chronic obstructive asthma with status asthmaticus | 5-dig billing code | Condition | ICD9CM |
| 3240129 | Obstructive emphysema | Clinical Finding | Condition | Nebraska Lexicon |
| 45917671 | moderate chronic obstructive pulmonary disease | Diagnosis | Condition | CIEL |
| 40316538 | Acute exacerbation of chronic obstructive airways disease | Clinical Finding | Condition | SNOMED |
| 45921028 | Centriacinar Emphysema | Diagnosis | Condition | CIEL |
| 4051464 | Chronic obstructive pulmonary disease with acute exacerbation, unspecified | Clinical Finding | Condition | SNOMED |
| 3375330 | Acute exacerbation of chronic obstructive airways disease with asthma | Clinical Finding | Condition | Nebraska Lexicon |
| 35208019 | Panlobular emphysema | 4-char billing code | Condition | ICD10CM |
| 3410488 | Segmental bullous emphysema | Clinical Finding | Condition | Nebraska Lexicon |
| 40316540 | Acute exacerbation of chronic obstructive airways disease | Clinical Finding | Condition | SNOMED |
| 3261420 | Giant bullous emphysema | Clinical Finding | Condition | Nebraska Lexicon |
| 44807895 | Acute non-infective exacerbation of chronic obstructive pulmonary disease | Clinical Finding | Condition | SNOMED |
| 3105916 | Moderate chronic obstructive pulmonary disease | Clinical Finding | Condition | Nebraska Lexicon |
| 45950153 | Chronic Obliterative Bronchiolitis | Diagnosis | Condition | CIEL |
| 3105904 | Chronic obstructive lung disease | Clinical Finding | Condition | Nebraska Lexicon |
| 3150335 | Infantile lobar emphysema | Clinical Finding | Condition | Nebraska Lexicon |
| 40395905 | Mild chronic obstructive pulmonary disease | Clinical Finding | Condition | SNOMED |
| 3274947 | Chronic emphysema caused by chemical fumes | Clinical Finding | Condition | Nebraska Lexicon |
| 45605888 | Panlobular emphysema | ICD10 code | Condition | ICD10 |
| 44827822 | Emphysematous bleb | 4-dig billing code | Condition | ICD9CM |
| 3308437 | CLE - Congenital lobar emphysema | Clinical Finding | Condition | Nebraska Lexicon |
| 45543268 | Other chronic obstructive pulmonary disease | ICD10 Hierarchy | Condition | ICD10 |
| 4046986 | End stage chronic obstructive airways disease | Clinical Finding | Condition | SNOMED |
| 3267799 | Asthma-chronic obstructive pulmonary disease overlap syndrome | Clinical Finding | Condition | Nebraska Lexicon |
| 3389102 | Chronic bronchiolitis obliterans caused by chemical fumes | Clinical Finding | Condition | Nebraska Lexicon |
| 3322063 | Cystic-bullous disease of the lung | Clinical Finding | Condition | Nebraska Lexicon |
| 45906202 | CHRONIC OBSTRUCTIVE PULMONARY DISEASE | Diagnosis | Condition | CIEL |
| 259043 | Emphysematous bleb of lung | Clinical Finding | Condition | SNOMED |
| 45956480 | mild chronic obstructive pulmonary disease | Diagnosis | Condition | CIEL |
| 44829011 | Emphysema | 3-dig nonbill code | Condition | ICD9CM |
| 4110056 | Chronic obstructive pulmonary disease with acute lower respiratory infection | Clinical Finding | Condition | SNOMED |
| 46274062 | Asthma-chronic obstructive pulmonary disease overlap syndrome | Clinical Finding | Condition | SNOMED |
| 4050732 | Pulmonary emphysema in alpha-1 PI deficiency | Clinical Finding | Condition | SNOMED |
| 45586673 | Other specified chronic obstructive pulmonary disease | ICD10 code | Condition | ICD10 |
| 3125011 | Chronic obstructive pulmonary disease with acute exacerbation, unspecified | Clinical Finding | Condition | Nebraska Lexicon |
| 44826677 | Other emphysema | 4-dig billing code | Condition | ICD9CM |
| 3184997 | Alveolar emphysema of lung | Clinical Finding | Condition | Nebraska Lexicon |
| 45596291 | Compensatory emphysema | ICD10 code | Condition | ICD10 |
| 4112674 | Other specified chronic obstructive airways disease | Clinical Finding | Condition | SNOMED |
| 44829013 | Chronic airway obstruction, not elsewhere classified | 3-dig billing code | Condition | ICD9CM |
| 3408420 | Chronic obstructive pulmonary disease with acute lower respiratory infection | Clinical Finding | Condition | Nebraska Lexicon |
| 45937316 | Giant Bullous Emphysema | Diagnosis | Condition | CIEL |
| 1569486 | Emphysema | 3-char nonbill code | Condition | ICD10CM |
| 3124983 | Chronic bullous emphysema NOS | Clinical Finding | Condition | Nebraska Lexicon |
| 35208021 | Other emphysema | 4-char billing code | Condition | ICD10CM |
| 4278831 | Chronic diffuse emphysema due to inhalation of chemical fumes AND/OR vapors | Clinical Finding | Condition | SNOMED |
| 40316555 | Chronic obstructive airways disease NOS | Clinical Finding | Condition | SNOMED |
| 3105915 | Mild chronic obstructive pulmonary disease | Clinical Finding | Condition | Nebraska Lexicon |
| 45567264 | Emphysema, unspecified | ICD10 code | Condition | ICD10 |
| 4188974 | Chronic obstructive pulmonary disease finding | Clinical Finding | Condition | SNOMED |
| 45930910 | Vanishing Lung | Diagnosis | Condition | CIEL |
| 4050734 | Scar emphysema | Clinical Finding | Condition | SNOMED |
| 3318720 | Ruptured emphysematous bleb of lung | Clinical Finding | Condition | Nebraska Lexicon |
| 3379105 | Chronic irreversible airway obstruction | Clinical Finding | Condition | Nebraska Lexicon |
| 44837136 | Chronic obstructive asthma with (acute) exacerbation | 5-dig billing code | Condition | ICD9CM |
| 45601132 | Chronic obstructive pulmonary disease with acute exacerbation, unspecified | ICD10 code | Condition | ICD10 |
| 45950152 | Chronic Obliterative Bronchiolitis due to Inhalation of Chemical Fumes and Vapors | Diagnosis | Condition | CIEL |
| 3219740 | Bullous emphysema with collapse | Clinical Finding | Condition | Nebraska Lexicon |
| 3125008 | Other specified chronic obstructive pulmonary disease | Clinical Finding | Condition | Nebraska Lexicon |
| 4110635 | Segmental bullous emphysema | Clinical Finding | Condition | SNOMED |
| 3130624 | Perinatal interstitial emphysema or related condition NOS | Clinical Finding | Condition | Nebraska Lexicon |
| 4050733 | Toxic emphysema | Clinical Finding | Condition | SNOMED |
| 4193588 | Moderate chronic obstructive pulmonary disease | Clinical Finding | Condition | SNOMED |
| 3130622 | Other specified perinatal interstitial emphysema or related condition | Clinical Finding | Condition | Nebraska Lexicon |
| 4246105 | Hemolytic anemia with emphysema AND cutis laxa | Clinical Finding | Condition | SNOMED |
| 45548115 | Chronic obstructive pulmonary disease, unspecified | ICD10 code | Condition | ICD10 |
| 3278438 | Pulmonary emphysema in alpha-1 primary immunodeficiency deficiency | Clinical Finding | Condition | Nebraska Lexicon |
| 3105948 | Chronic obstructive airways disease NOS | Clinical Finding | Condition | Nebraska Lexicon |
| 3115991 | Chronic obstructive airway disease with asthma | Clinical Finding | Condition | Nebraska Lexicon |
| 4177944 | Panacinar emphysema | Clinical Finding | Condition | SNOMED |
| 45927292 | Obstructive Chronic Bronchitis with Acute Bronchitis | Diagnosis | Condition | CIEL |
| 3214333 | Chronic diffuse emphysema caused by inhalation of chemical fumes AND/OR vapours | Clinical Finding | Condition | Nebraska Lexicon |
| 40538605 | Congenital emphysema | Clinical Finding | Condition | SNOMED |
| 4110046 | Obstructive chronic bronchitis NOS | Clinical Finding | Condition | SNOMED |
| 3105905 | Acute exacerbation of chronic obstructive airways disease | Clinical Finding | Condition | Nebraska Lexicon |
| 45552890 | Centrilobular emphysema | ICD10 code | Condition | ICD10 |
| 4104506 | Cystic-bullous disease of the lung | Clinical Finding | Condition | SNOMED |
| 4115044 | Acute infective exacerbation of chronic obstructive airways disease | Clinical Finding | Condition | SNOMED |
| 3411364 | Zonal bullous emphysema | Clinical Finding | Condition | Nebraska Lexicon |
| 3310749 | Vanishing lung | Clinical Finding | Condition | Nebraska Lexicon |
| 3327847 | Pulmonary emphysema | Clinical Finding | Condition | Nebraska Lexicon |
| 3414592 | Chemical emphysema | Clinical Finding | Condition | Nebraska Lexicon |
| 45925335 | Panacinar Emphysema | Diagnosis | Condition | CIEL |
| 3105900 | Chronic obstructive lung disease | Clinical Finding | Condition | Nebraska Lexicon |
| 3124985 | Emphysema: [acute interstitial] or [atrophic - senile] | Clinical Finding | Condition | Nebraska Lexicon |
| 44834775 | Interstitial emphysema | 4-dig billing code | Condition | ICD9CM |
| 4048151 | Other specified perinatal interstitial emphysema or related condition | Clinical Finding | Condition | SNOMED |
| 40316552 | Mild chronic obstructive pulmonary disease | Clinical Finding | Condition | SNOMED |
| 45949452 | severe chronic obstructive pulmonary disease | Diagnosis | Condition | CIEL |
| 3125006 | Moderate chronic obstructive pulmonary disease | Clinical Finding | Condition | Nebraska Lexicon |
| 3242416 | Chronic obstructive lung disease co-occurrent with acute bronchitis | Clinical Finding | Condition | Nebraska Lexicon |
| 4286497 | Centriacinar emphysema | Clinical Finding | Condition | SNOMED |
| 3130621 | Perinatal interstitial emphysema and related conditions | Clinical Finding | Condition | Nebraska Lexicon |
| 45941119 | Pulmonary Emphysema | Diagnosis | Condition | CIEL |
| 3155005 | Interstitial pulmonary emphysema | Clinical Finding | Condition | Nebraska Lexicon |
| 3363972 | Tension pneumatocele of lung | Clinical Finding | Condition | Nebraska Lexicon |
| 3124978 | Obstructive chronic bronchitis NOS | Clinical Finding | Condition | Nebraska Lexicon |
| 45943866 | Ruptured Emphysematous Bleb of Lung | Diagnosis | Condition | CIEL |
| 40395852 | Chronic obstructive lung disease | Clinical Finding | Condition | SNOMED |
| 3124976 | Chronic obstructive lung disease | Clinical Finding | Condition | Nebraska Lexicon |
| 3137827 | Chronic obstructive pulmonary disease with acute exacerbation, unspecified | Clinical Finding | Condition | Nebraska Lexicon |
| 3185561 | Chronic bullous emphysema | Clinical Finding | Condition | Nebraska Lexicon |
| 44831280 | Chronic obstructive asthma, unspecified | 5-dig billing code | Condition | ICD9CM |
| 3319488 | Pulmonary emphysema co-occurrent with fibrosis of lung | Clinical Finding | Condition | Nebraska Lexicon |
| 4166517 | Chronic obliterative bronchiolitis | Clinical Finding | Condition | SNOMED |
| 4112828 | Zonal bullous emphysema | Clinical Finding | Condition | SNOMED |
| 3365475 | Compensatory emphysema | Clinical Finding | Condition | Nebraska Lexicon |
| 45586672 | Emphysema | ICD10 Hierarchy | Condition | ICD10 |
| 40395906 | Moderate chronic obstructive pulmonary disease | Clinical Finding | Condition | SNOMED |
| 4136683 | Paraseptal emphysema | Clinical Finding | Condition | SNOMED |
| 3245473 | Atrophic (senile) emphysema | Clinical Finding | Condition | Nebraska Lexicon |
| 3341426 | Interstitial pulmonary emphysema | Clinical Finding | Condition | Nebraska Lexicon |
| 261325 | Pulmonary emphysema | Clinical Finding | Condition | SNOMED |
| 45769389 | Pulmonary emphysema co-occurrent with fibrosis of lung | Clinical Finding | Condition | SNOMED |
| 45950378 | Emphysematous Bleb of Lung | Diagnosis | Condition | CIEL |
| 44821995 | Compensatory emphysema | 4-dig billing code | Condition | ICD9CM |
| 4138392 | Chronic obliterative bronchiolitis due to inhalation of chemical fumes AND/OR vapors | Clinical Finding | Condition | SNOMED |
| 45928382 | Haemolytic Anaemia with Emphysema and Cutis Laxa | Diagnosis | Condition | CIEL |
| 3353242 | Acute vesicular emphysema | Clinical Finding | Condition | Nebraska Lexicon |
| 40395907 | Severe chronic obstructive pulmonary disease | Clinical Finding | Condition | SNOMED |
| 3158478 | Chronic obstructive pulmonary disease finding | Clinical Finding | Condition | Nebraska Lexicon |
| 1569487 | Other chronic obstructive pulmonary disease | 3-char nonbill code | Condition | ICD10CM |
| 4148124 | Atrophic (senile) emphysema | Clinical Finding | Condition | SNOMED |
| 43530693 | Acute exacerbation of chronic obstructive airways disease with asthma | Clinical Finding | Condition | SNOMED |
| 3237311 | Acute infective exacerbation of chronic obstructive airways disease | Clinical Finding | Condition | Nebraska Lexicon |
| 45601131 | Chronic obstructive pulmonary disease with acute lower respiratory infection | ICD10 code | Condition | ICD10 |
| 257004 | Acute exacerbation of chronic obstructive airways disease | Clinical Finding | Condition | SNOMED |
| 3332190 | Moderate chronic obstructive pulmonary disease | Clinical Finding | Condition | Nebraska Lexicon |
| 46269701 | Chronic obstructive lung disease co-occurrent with acute bronchitis | Clinical Finding | Condition | SNOMED |
| 3247896 | Chronic obliterative bronchiolitis | Clinical Finding | Condition | Nebraska Lexicon |
| 35208104 | Compensatory emphysema | 4-char billing code | Condition | ICD10CM |
| 40316533 | Chronic obstructive lung disease | Clinical Finding | Condition | SNOMED |
| 45928055 | Interstitial Emphysema of Lung | Diagnosis | Condition | CIEL |
| 35208020 | Centrilobular emphysema | 4-char billing code | Condition | ICD10CM |
| 3392939 | End stage chronic obstructive pulmonary disease | Clinical Finding | Condition | Nebraska Lexicon |
| 440748 | Interstitial emphysema of lung | Clinical Finding | Condition | SNOMED |
| 3466897 | Haemolytic anaemia with emphysema AND cutis laxa | Clinical Finding | Condition | Nebraska Lexicon |
| 4196712 | Mild chronic obstructive pulmonary disease | Clinical Finding | Condition | SNOMED |
| 45931272 | Pulmonary Emphysema in Alpha-1 Pi Deficiency | Diagnosis | Condition | CIEL |
| 4110048 | Chronic bullous emphysema | Clinical Finding | Condition | SNOMED |
| 45562454 | Other emphysema | ICD10 code | Condition | ICD10 |
| 3400716 | Scar emphysema | Clinical Finding | Condition | Nebraska Lexicon |
| 40395913 | Chronic obstructive pulmonary disease with acute exacerbation, unspecified | Clinical Finding | Condition | SNOMED |
| 43530700 | Chronic obstructive airway disease with asthma | Clinical Finding | Condition | SNOMED |
| 35208025 | Chronic obstructive pulmonary disease, unspecified | 4-char billing code | Condition | ICD10CM |
| 44829012 | Chronic obstructive asthma | 4-dig nonbill code | Condition | ICD9CM |
| 4166508 | Congenital emphysema | Clinical Finding | Condition | SNOMED |
| 40395870 | Emphysema: [acute interstitial] or [atrophic - senile] | Clinical Finding | Condition | SNOMED |
| 4145496 | Bullous emphysema with collapse | Clinical Finding | Condition | SNOMED |
| 3402468 | Mild chronic obstructive pulmonary disease | Clinical Finding | Condition | Nebraska Lexicon |
| 3185549 | Bronchiectasis with acute exacerbation | Clinical Finding | Condition | Nebraska Lexicon |
| 3235894 | Peripheral lobular emphysema | Clinical Finding | Condition | Nebraska Lexicon |
| 3125005 | Mild chronic obstructive pulmonary disease | Clinical Finding | Condition | Nebraska Lexicon |
| 3125007 | Severe chronic obstructive pulmonary disease | Clinical Finding | Condition | Nebraska Lexicon |
| 40316542 | Emphysema | Clinical Finding | Condition | SNOMED |
| 3353524 | Centriacinar emphysema | Clinical Finding | Condition | Nebraska Lexicon |
| 45944263 | Paraseptal Emphysema | Diagnosis | Condition | CIEL |
| 4083395 | Vanishing lung | Clinical Finding | Condition | SNOMED |
| 4112829 | Chronic bullous emphysema NOS | Clinical Finding | Condition | SNOMED |
| 4112836 | Chronic emphysema due to chemical fumes | Clinical Finding | Condition | SNOMED |
| 3415340 | Severe chronic obstructive pulmonary disease | Clinical Finding | Condition | Nebraska Lexicon |
| 3253499 | Acute exacerbation of chronic obstructive pulmonary disease | Clinical Finding | Condition | Nebraska Lexicon |
| 255573 | Chronic obstructive lung disease | Clinical Finding | Condition | SNOMED |
| 45948428 | Congenital Emphysema | Diagnosis | Condition | CIEL |
| 35208023 | Chronic obstructive pulmonary disease with (acute) lower respiratory infection | 4-char billing code | Condition | ICD10CM |
| 3105908 | Emphysema | Clinical Finding | Condition | Nebraska Lexicon |
| 45913847 | Chronic Airway Obstruction | Diagnosis | Condition | CIEL |
| 45934870 | chronic bullous emphysema | Diagnosis | Condition | CIEL |
| 44796286 | [X]Other specified chronic obstructive pulmonary disease | Clinical Finding | Condition | SNOMED |
| 3105917 | Severe chronic obstructive pulmonary disease | Clinical Finding | Condition | Nebraska Lexicon |
| 40561762 | Interstitial pulmonary emphysema | Clinical Finding | Condition | SNOMED |
| 4315386 | Ruptured emphysematous bleb of lung | Clinical Finding | Condition | SNOMED |
| 40316537 | Chronic obstructive lung disease | Clinical Finding | Condition | SNOMED |
| 45946309 | Chronic Obstructive Pulmonary Disease with Acute Lower Respiratory Infection | Diagnosis | Condition | CIEL |
| 45930249 | Chronic Diffuse Emphysema due to Inhalation of Chemical Fumes and Vapors | Diagnosis | Condition | CIEL |
| 44791725 | Very severe chronic obstructive pulmonary disease | Clinical Finding | Condition | SNOMED |
| 3125012 | Chronic obstructive pulmonary disease NOS | Clinical Finding | Condition | Nebraska Lexicon |
| 35208024 | Chronic obstructive pulmonary disease with (acute) exacerbation | 4-char billing code | Condition | ICD10CM |
| 4110049 | Acute vesicular emphysema | Clinical Finding | Condition | SNOMED |
| 40316553 | Moderate chronic obstructive pulmonary disease | Clinical Finding | Condition | SNOMED |
| 4110175 | Chronic obstructive airways disease NOS | Clinical Finding | Condition | SNOMED |
| 45877605 | COPD | Answer | Meas Value | LOINC |
| 40316554 | Severe chronic obstructive pulmonary disease | Clinical Finding | Condition | SNOMED |
| 261895 | Compensatory emphysema | Clinical Finding | Condition | SNOMED |
| 45921408 | Compensatory Emphysema | Diagnosis | Condition | CIEL |
| 40316591 | Chronic obstructive airways disease NOS | Clinical Finding | Condition | SNOMED |
| 3105918 | Chronic obstructive airways disease NOS | Clinical Finding | Condition | Nebraska Lexicon |
| 45908973 | Obstructive Emphysema | Diagnosis | Condition | CIEL |

Table 5. Respiratory distress syndrome (ARDS) or acute respiratory failure (ARF).

| **Concept ID** | **Concept Name** | **Class** | **Domain** | **Vocabulary** |
| --- | --- | --- | --- | --- |
| 765888 | Acute-on-chronic respiratory failure following trauma | Clinical Finding | Condition | SNOMED |
| 46273719 | Acute hypercapnic respiratory failure | Clinical Finding | Condition | SNOMED |
| 319049 | Acute respiratory failure | Clinical Finding | Condition | SNOMED |
| 37016114 | Acute on chronic hypoxemic respiratory failure | Clinical Finding | Condition | SNOMED |
| 36716978 | Acute on chronic hypercapnic respiratory failure | Clinical Finding | Condition | SNOMED |
| 4232327 | Postimmersion-submersion syndrome | Clinical Finding | Condition | SNOMED |
| 4024117 | Pulmonary insufficiency following surgery | Clinical Finding | Condition | SNOMED |
| 4195694 | Acute respiratory distress syndrome | Clinical Finding | Condition | SNOMED |
| 42539533 | Acute hypoxemic and hypercapnic respiratory failure | Clinical Finding | Condition | SNOMED |
| 37399087 | Post extubation acute respiratory failure requiring reintubation | Clinical Finding | Condition | SNOMED |
| 4024118 | Pulmonary insufficiency following trauma | Clinical Finding | Condition | SNOMED |
| 4025173 | Pulmonary insufficiency following shock | Clinical Finding | Condition | SNOMED |
| 312940 | Acute-on-chronic respiratory failure | Clinical Finding | Condition | SNOMED |
| 4266019 | Acute respiratory failure requiring reintubation | Clinical Finding | Condition | SNOMED |
| 35615340 | Acute on chronic hypoxemic and hypercapnic respiratory failure | Clinical Finding | Condition | SNOMED |
| 46269690 | Acute hypercapnic respiratory failure due to obstructive sleep apnea | Clinical Finding | Condition | SNOMED |
| 46271075 | Acute hypoxemic respiratory failure | Clinical Finding | Condition | SNOMED |

Table 6. Myocardial infraction.

| **Concept ID** | **Concept Name** | **Class** | **Domain** | **Vocabulary** |
| --- | --- | --- | --- | --- |
| 314666 | Old myocardial infarction | Clinical Finding | Condition | SNOMED |
| 45766116 | Acute ST segment elevation myocardial infarction of inferior wall | Clinical Finding | Condition | SNOMED |
| 45766114 | Subsequent ST segment elevation myocardial infarction | Clinical Finding | Condition | SNOMED |
| 45766241 | Subsequent non-ST segment elevation myocardial infarction | Clinical Finding | Condition | SNOMED |
| 43020460 | Acute ST segment elevation myocardial infarction involving left anterior descending coronary artery | Clinical Finding | Condition | SNOMED |
| 46270163 | Acute ST segment elevation myocardial infarction due to right coronary artery occlusion | Clinical Finding | Condition | SNOMED |
| 4108218 | Subsequent myocardial infarction of inferior wall | Clinical Finding | Condition | SNOMED |
| 4270024 | Acute non-ST segment elevation myocardial infarction | Clinical Finding | Condition | SNOMED |
| 4296653 | Acute ST segment elevation myocardial infarction | Clinical Finding | Condition | SNOMED |
| 46270162 | Acute ST segment elevation myocardial infarction due to left coronary artery occlusion | Clinical Finding | Condition | SNOMED |
| 312327 | Acute myocardial infarction | Clinical Finding | Condition | SNOMED |
| 4108677 | Subsequent myocardial infarction of anterior wall | Clinical Finding | Condition | SNOMED |
| 4108217 | Subsequent myocardial infarction | Clinical Finding | Condition | SNOMED |
| 45766075 | Acute anterior ST segment elevation myocardial infarction | Clinical Finding | Condition | SNOMED |
| 4329847 | Myocardial infarction | Clinical Finding | Condition | SNOMED |

Table 7. Congestive heart failure (CHF).

| **Concept ID** | **Concept Name** | **Class** | | **Domain** | | **Vocabulary** |
| --- | --- | --- | --- | --- | --- | --- |
| 40482727 | Combined systolic and diastolic dysfunction | Clinical Finding | | Condition | | SNOMED |
| 40480602 | Acute on chronic systolic heart failure | Clinical Finding | Condition | | SNOMED | |
| 40479576 | Chronic diastolic heart failure | Clinical Finding | Condition | | SNOMED | |
| 4163710 | Dilated cardiomyopathy | Clinical Finding | Condition | | SNOMED | |
| 4229440 | Chronic congestive heart failure | Clinical Finding | Condition | | SNOMED | |
| 4110961 | Generalized ischemic myocardial dysfunction | Clinical Finding | Condition | | SNOMED | |
| 44782718 | Acute combined systolic and diastolic heart failure | Clinical Finding | Condition | | SNOMED | |
| 4004279 | High output heart failure | Clinical Finding | Condition | | SNOMED | |
| 316139 | Heart failure | Clinical Finding | Condition | | SNOMED | |
| 40479192 | Chronic systolic heart failure | Clinical Finding | Condition | | SNOMED | |
| 4233424 | Acute right-sided heart failure | Clinical Finding | Condition | | SNOMED | |
| 44782719 | Chronic combined systolic and diastolic heart failure | Clinical Finding | Condition | | SNOMED | |
| 37309625 | Acute on chronic right-sided congestive heart failure | Clinical Finding | Condition | | SNOMED | |
| 320746 | Cardiomyopathy associated with another disorder | Clinical Finding | Condition | | SNOMED | |
| 443580 | Systolic heart failure | Clinical Finding | Condition | | SNOMED | |
| 4273632 | Right ventricular failure | Clinical Finding | Condition | | SNOMED | |
| 443587 | Diastolic heart failure | Clinical Finding | Condition | | SNOMED | |
| 444101 | Hypertensive heart failure | Clinical Finding | Condition | | SNOMED | |
| 40481042 | Acute diastolic heart failure | Clinical Finding | Condition | | SNOMED | |
| 40481043 | Acute on chronic diastolic heart failure | Clinical Finding | Condition | | SNOMED | |
| 321319 | Cardiomyopathy | Clinical Finding | Condition | | SNOMED | |
| 439696 | Hypertensive heart and renal disease with (congestive) heart failure | Clinical Finding | Condition | | SNOMED | |
| 4172864 | Neonatal cardiac failure | Clinical Finding | Condition | | SNOMED | |
| 439846 | Left heart failure | Clinical Finding | Condition | | SNOMED | |
| 318773 | Dilated cardiomyopathy secondary to alcohol | Clinical Finding | Condition | | SNOMED | |
| 4190773 | Restrictive cardiomyopathy | Clinical Finding | Condition | | SNOMED | |
| 4195785 | Right heart failure secondary to left heart failure | Clinical Finding | Condition | | SNOMED | |
| 319835 | Congestive heart failure | Clinical Finding | Condition | | SNOMED | |
| 4242669 | Biventricular congestive heart failure | Clinical Finding | Condition | | SNOMED | |
| 44782733 | Acute on chronic combined systolic and diastolic heart failure | Clinical Finding | Condition | | SNOMED | |
| 40480603 | Acute systolic heart failure | Clinical Finding | Condition | | SNOMED | |
| 4014159 | Chronic right-sided heart failure | Clinical Finding | Condition | | SNOMED | |

Table 8. AIDS.

| **Concept ID** | **Concept Name** | **Class** | **Domain** | **Vocabulary** |
| --- | --- | --- | --- | --- |
| 41208473 | abacavir Oral Tablet [Abacavir Hexal] | Branded Drug Form | Drug | RxNorm Extension |
| 44045833 | dasabuvir 250 MG / ombitasvir 12.5 MG / paritaprevir 75 MG / Ritonavir 50 MG Oral Tablet | Clinical Drug | Drug | RxNorm Extension |
| 45892739 | atazanavir 50 MG | Clinical Drug Comp | Drug | RxNorm |
| 41201623 | Nevirapine 0.0417 MG/ML | Clinical Drug Comp | Drug | RxNorm Extension |
| 43026166 | emtricitabine 200 MG / tenofovir disoproxil 250 MG Delayed Release Oral Tablet Box of 30 by KRKA | Marketed Product | Drug | RxNorm Extension |
| 19081175 | didanosine 50 MG | Clinical Drug Comp | Drug | RxNorm |
| 36061908 | lopinavir 200 MG / ritonavir 50 MG Delayed Release Oral Tablet by Mylan | Marketed Product | Drug | RxNorm Extension |
| 21033712 | efavirenz 50 MG Oral Capsule [Sustiva] Box of 30 by Bristol Myers Squibb | Marketed Product | Drug | RxNorm Extension |
| 41260971 | emtricitabine 200 MG Oral Capsule [Emtriva] Box of 30 by Emra-Med | Marketed Product | Drug | RxNorm Extension |
| 36249788 | dolutegravir 25 MG Oral Tablet [Tivicay] | Branded Drug | Drug | RxNorm |
| 21110708 | Indinavir 333 MG Oral Capsule [Crixivan] Box of 135 by Merck | Marketed Product | Drug | RxNorm Extension |
| 36061971 | atazanavir 200 MG Oral Capsule by Accord | Marketed Product | Drug | RxNorm Extension |
| 21023723 | Lamivudine 300 MG Oral Tablet by A A H | Marketed Product | Drug | RxNorm Extension |
| 43169082 | tenofovir disoproxil 250 MG Delayed Release Oral Tablet | Clinical Drug | Drug | RxNorm Extension |
| 45892214 | {2 (dasabuvir 250 MG Oral Tablet) / 2 (ombitasvir 12.5 MG / paritaprevir 75 MG / ritonavir 50 MG Oral Tablet) } Pack [Viekira Pak] | Branded Pack | Drug | RxNorm |
| 21026616 | darunavir 150 MG Oral Tablet [Prezista] by Janssen | Marketed Product | Drug | RxNorm Extension |
| 44062950 | Nelfinavir 250 MG Oral Tablet [Viracept] by Pfizer | Marketed Product | Drug | RxNorm Extension |
| 41257682 | Ritonavir 100 MG Oral Tablet [Norvir] Box of 30 by Orifarm Leverkus | Marketed Product | Drug | RxNorm Extension |
| 21121799 | Lamivudine 300 MG Oral Tablet Box of 30 by A A H | Marketed Product | Drug | RxNorm Extension |
| 44128617 | Delavirdine 100 MG Oral Tablet [Rescriptor] by ViiV | Marketed Product | Drug | RxNorm Extension |
| 43177133 | Didanosine 250 MG Extended Release Oral Tablet Box of 30 | Clinical Drug Box | Drug | RxNorm Extension |
| 40730818 | abacavir 600 MG / Lamivudine 300 MG Oral Tablet Box of 30 by Mylan | Marketed Product | Drug | RxNorm Extension |
| 36261256 | Didanosine 250 MG Delayed Release Oral Capsule [Videx] Box of 30 by Bristol Myers Squibb | Marketed Product | Drug | RxNorm Extension |
| 43661062 | Atazanavir 228 MG Oral Capsule [Atazanavir-Ratiopharm] | Branded Drug | Drug | RxNorm Extension |
| 41209226 | Ritonavir 0.889 MG/ML Oral Solution Box of 1 | Clinical Drug Box | Drug | RxNorm Extension |
| 40919110 | Atazanavir 300 MG Oral Capsule [Reyataz] Box of 30 by Aca Mueller | Marketed Product | Drug | RxNorm Extension |
| 36257452 | Saquinavir 500 MG Oral Capsule [Invirase] | Branded Drug | Drug | RxNorm Extension |
| 41228124 | Nevirapine 400 MG Oral Tablet [Viramune] Box of 30 by Gerke | Marketed Product | Drug | RxNorm Extension |
| 43146908 | Rilpivirine Delayed Release Oral Tablet | Clinical Drug Form | Drug | RxNorm Extension |
| 43022435 | darunavir 400 MG Delayed Release Oral Tablet Box of 60 by Sandoz | Marketed Product | Drug | RxNorm Extension |
| 44159913 | Lamivudine 150 MG / Zidovudine 300 MG Oral Tablet [Combivir] Box of 60 by European | Marketed Product | Drug | RxNorm Extension |
| 44058013 | abacavir / Lamivudine Oral Tablet [Pms-Abacavir-Lamivudine] | Branded Drug Form | Drug | RxNorm Extension |
| 40724596 | Ritonavir 100 MG Oral Tablet Box of 30 by Mylan | Marketed Product | Drug | RxNorm Extension |
| 36227427 | tenofovir disoproxil Oral Product | Clinical Dose Group | Drug | RxNorm |
| 40753349 | Nevirapine 400 MG Extended Release Oral Tablet Box of 30 by TEVA | Marketed Product | Drug | RxNorm Extension |
| 2052801 | tenofovir disoproxil Oral Tablet [TENOFREE] | Branded Drug Form | Drug | RxNorm Extension |
| 21121991 | Amprenavir 50 MG Oral Capsule [Agenerase] Box of 480 by Glaxosmithkline | Marketed Product | Drug | RxNorm Extension |
| 36062188 | tenofovir disoproxil 250 MG Delayed Release Oral Tablet by Zentiva | Marketed Product | Drug | RxNorm Extension |
| 36061611 | darunavir 400 MG Oral Tablet by Dr Reddy's | Marketed Product | Drug | RxNorm Extension |
| 35858572 | stavudine Oral Capsule [Xinfuda] | Branded Drug Form | Drug | RxNorm Extension |
| 41252913 | Didanosine 125 MG Extended Release Oral Capsule [Videx] Box of 60 | Branded Drug Box | Drug | RxNorm Extension |
| 36248761 | atazanavir / cobicistat Pill | Clinical Dose Group | Drug | RxNorm |
| 41259821 | Lamivudine 100 MG Oral Tablet [Zeffix] Box of 84 by Kohlpharma | Marketed Product | Drug | RxNorm Extension |
| 40709680 | 454 ML Ritonavir 80 MG/ML Oral Solution [Norvir] by Abbvie | Marketed Product | Drug | RxNorm Extension |
| 21131575 | Lamivudine 150 MG Oral Tablet Box of 60 | Clinical Drug Box | Drug | RxNorm Extension |
| 42706894 | etravirine 25 MG [Intelence] | Branded Drug Comp | Drug | RxNorm |
| 41011265 | efavirenz 600 MG Oral Tablet [Sustiva] Box of 90 by Aca Mueller | Marketed Product | Drug | RxNorm Extension |
| 41199396 | Didanosine 200 MG Oral Capsule [Videx] Box of 60 by Eurim-Pharm | Marketed Product | Drug | RxNorm Extension |
| 19006257 | didanosine 400 MG [Videx EC] | Branded Drug Comp | Drug | RxNorm |
| 40961370 | Didanosine 250 MG Oral Capsule | Clinical Drug | Drug | RxNorm Extension |
| 42918908 | 240 ML Ritonavir 80 MG/ML Oral Solution [Norvir] | Quant Branded Drug | Drug | RxNorm Extension |
| 41288583 | Saquinavir 500 MG Delayed Release Oral Tablet [Invirase] Box of 120 by Cc | Marketed Product | Drug | RxNorm Extension |
| 35862031 | zidovudine Oral Capsule [Qiluoke] | Branded Drug Form | Drug | RxNorm Extension |
| 43204732 | ombitasvir / paritaprevir / Ritonavir Delayed Release Oral Tablet [Viekirax] | Branded Drug Form | Drug | RxNorm Extension |
| 44042631 | abacavir 600 MG / Lamivudine 300 MG [Mylan-Abacavir/Lamivudine] | Branded Drug Comp | Drug | RxNorm Extension |
| 44114706 | Lamivudine 150 MG Oral Tablet [Auro-Lamivudine] by Auro | Marketed Product | Drug | RxNorm Extension |
| 36894603 | Nevirapine 400 MG Oral Tablet | Clinical Drug | Drug | RxNorm Extension |
| 43177134 | Didanosine 150 MG Chewable Tablet [Videx] Box of 60 by Bristol Myers Squibb | Marketed Product | Drug | RxNorm Extension |
| 43022451 | darunavir 600 MG Oral Tablet Box of 60 by KRKA | Marketed Product | Drug | RxNorm Extension |
| 42683461 | DORAVIRINE 100 MG / Lamivudine 300 MG / tenofovir disoproxil 250 MG Oral Tablet | Clinical Drug | Drug | RxNorm Extension |
| 40943476 | Atazanavir 300 MG Oral Capsule [Reyataz] Box of 90 | Branded Drug Box | Drug | RxNorm Extension |
| 21063033 | Stavudine 40 MG Oral Capsule [Zerit] by Bristol Myers Squibb | Marketed Product | Drug | RxNorm Extension |
| 40882846 | tenofovir disoproxil 250 MG Oral Tablet [Viread] Box of 30 by Inopha | Marketed Product | Drug | RxNorm Extension |
| 41103894 | Lamivudine 150 MG / Zidovudine 300 MG Oral Tablet [Combivir] Box of 60 by Aca Mueller | Marketed Product | Drug | RxNorm Extension |
| 1781410 | stavudine 40 MG Oral Capsule [Zerit] | Branded Drug | Drug | RxNorm |
| 44089547 | Didanosine 50 MG Oral Tablet [Videx] by Bristol Myers Squibb | Marketed Product | Drug | RxNorm Extension |
| 2052767 | tenofovir disoproxil 300 MG [TENOVA] | Branded Drug Comp | Drug | RxNorm Extension |
| 43160868 | lopinavir 80 MG/ML / Ritonavir 20 MG/ML Oral Solution Box of 5 | Clinical Drug Box | Drug | RxNorm Extension |
| 42708096 | tenofovir disoproxil fumarate 250 MG Oral Tablet | Clinical Drug | Drug | RxNorm |
| 36226561 | efavirenz / lamivudine / tenofovir disoproxil Oral Product | Clinical Dose Group | Drug | RxNorm |
| 43268522 | Stavudine 40 MG Oral Capsule Box of 60 | Clinical Drug Box | Drug | RxNorm Extension |
| 40875590 | Lamivudine 150 MG Oral Tablet [LAMIVUDINE MYLAN] Box of 60 | Branded Drug Box | Drug | RxNorm Extension |
| 43187466 | 240 ML Lamivudine 5 MG/ML Oral Solution Box of 1 | Quant Clinical Box | Drug | RxNorm Extension |
| 43023778 | Nevirapine 200 MG Oral Tablet Box of 60 by Arrow | Marketed Product | Drug | RxNorm Extension |
| 21121987 | efavirenz 30 MG/ML Oral Solution [Sustiva] | Branded Drug | Drug | RxNorm Extension |
| 1511082 | dolutegravir / lamivudine Oral Product | Clinical Dose Group | Drug | RxNorm |
| 21043415 | Ritonavir 100 MG Oral Tablet by Abbvie | Marketed Product | Drug | RxNorm Extension |
| 19102831 | emtricitabine 200 MG Oral Capsule [Emtriva] | Branded Drug | Drug | RxNorm |
| 35412092 | emtricitabine 200 MG / tenofovir alafenamide 10 MG Delayed Release Oral Tablet | Clinical Drug | Drug | RxNorm Extension |
| 40720869 | dolutegravir 10 MG Oral Tablet [Tivicay] Box of 30 by ViiV | Marketed Product | Drug | RxNorm Extension |
| 41381586 | 20 ML Zidovudine 10 MG/ML Injectable Solution Box of 5 | Quant Clinical Box | Drug | RxNorm Extension |
| 43202075 | darunavir 75 MG Delayed Release Oral Tablet [Prezista] Box of 480 | Branded Drug Box | Drug | RxNorm Extension |
| 36785881 | efavirenz 200 MG Delayed Release Oral Tablet Box of 90 | Clinical Drug Box | Drug | RxNorm Extension |
| 36890547 | darunavir Oral Solution | Clinical Drug Form | Drug | RxNorm Extension |
| 43158209 | cobicistat / elvitegravir / emtricitabine / tenofovir disoproxil Delayed Release Oral Tablet [Stribild] | Branded Drug Form | Drug | RxNorm Extension |
| 40097200 | abacavir / lamivudine / zidovudine Oral Tablet | Clinical Drug Form | Drug | RxNorm |
| 41273222 | efavirenz 0.167 MG/ML Oral Solution | Clinical Drug | Drug | RxNorm Extension |
| 36419264 | raltegravir 600 MG Oral Tablet Box of 180 | Clinical Drug Box | Drug | RxNorm Extension |
| 43144917 | efavirenz 30 MG/ML Oral Solution Box of 1 | Clinical Drug Box | Drug | RxNorm Extension |
| 36225762 | enfuvirtide Injectable Product | Clinical Dose Group | Drug | RxNorm |
| 21075763 | darunavir 75 MG Oral Tablet [Prezista] Box of 480 by Janssen | Marketed Product | Drug | RxNorm Extension |
| 41046666 | Nevirapine 200 MG [Nevirapin Ratiopharm] | Branded Drug Comp | Drug | RxNorm Extension |
| 36276926 | Nevirapine 400 MG [NEVIRAPINE MYLAN] | Branded Drug Comp | Drug | RxNorm Extension |
| 36891879 | Zidovudine 10 MG/ML Prefilled Syringe | Clinical Drug | Drug | RxNorm Extension |
| 40847307 | Didanosine 400 MG Delayed Release Oral Capsule [Videx] Box of 30 | Branded Drug Box | Drug | RxNorm Extension |
| 45775747 | abacavir 600 MG / dolutegravir 50 MG / lamivudine 300 MG Oral Tablet | Clinical Drug | Drug | RxNorm |
| 43031551 | maraviroc 75 MG/ML | Clinical Drug Comp | Drug | RxNorm Extension |
| 44171834 | abacavir 703 MG / Lamivudine 300 MG Oral Tablet [Kivexa] Box of 90 by Abacus Medicine | Marketed Product | Drug | RxNorm Extension |
| 41071598 | Nelfinavir 250 MG Oral Tablet [Viracept] Box of 270 by Roche | Marketed Product | Drug | RxNorm Extension |
| 21053215 | lopinavir 100 MG / Ritonavir 25 MG Oral Tablet [Kaletra] Box of 60 | Branded Drug Box | Drug | RxNorm Extension |
| 40741180 | dolutegravir 50 MG / Rilpivirine 25 MG Oral Tablet [Juluca] Box of 30 | Branded Drug Box | Drug | RxNorm Extension |
| 19127304 | maraviroc 300 MG [Selzentry] | Branded Drug Comp | Drug | RxNorm |
| 1781407 | stavudine 15 MG Oral Capsule [Zerit] | Branded Drug | Drug | RxNorm |
| 21173974 | maraviroc 300 MG Oral Tablet [Celsentri] Box of 60 by ViiV | Marketed Product | Drug | RxNorm Extension |
| 41226698 | Saquinavir 200 MG Oral Capsule [Fortovase] Box of 540 by Roche | Marketed Product | Drug | RxNorm Extension |
| 43145150 | Lamivudine 150 MG Delayed Release Oral Tablet [Epivir] | Branded Drug | Drug | RxNorm Extension |
| 41245640 | tenofovir disoproxil 0.04 MG/MG Oral Powder [Viread] Box of 1 | Branded Drug Box | Drug | RxNorm Extension |
| 44205180 | 4030 MG Didanosine 1 MG/MG Oral Solution [Videx] | Quant Branded Drug | Drug | RxNorm Extension |
| 19079870 | zalcitabine 0.75 MG Oral Tablet | Clinical Drug | Drug | RxNorm |
| 36277507 | Nevirapine 400 MG Extended Release Oral Capsule Box of 30 | Clinical Drug Box | Drug | RxNorm Extension |
| 21033496 | Lamivudine 150 MG Oral Tablet [Epivir] Box of 60 | Branded Drug Box | Drug | RxNorm Extension |
| 44167743 | Didanosine 250 MG Oral Capsule [Videx] Box of 30 by Gerke | Marketed Product | Drug | RxNorm Extension |
| 43786180 | Ritonavir 100 MG [Ritonavir Hexal] | Branded Drug Comp | Drug | RxNorm Extension |
| 36242594 | Viramune Oral Liquid Product | Branded Dose Group | Drug | RxNorm |
| 41133284 | raltegravir 400 MG Delayed Release Oral Tablet [Isentress] Box of 60 by Kohlpharma | Marketed Product | Drug | RxNorm Extension |
| 36225757 | emtricitabine / tenofovir disoproxil Oral Product | Clinical Dose Group | Drug | RxNorm |
| 43032210 | Didanosine 2000 MG Powder for Oral Solution [Videx] by Bristol Myers Squibb | Marketed Product | Drug | RxNorm Extension |
| 21024319 | Zidovudine 100 MG Oral Capsule Box of 60 by A A H | Marketed Product | Drug | RxNorm Extension |
| 35768846 | Lamivudine 300 MG Delayed Release Oral Tablet by TEVA | Marketed Product | Drug | RxNorm Extension |
| 782823 | emtricitabine / Tenofovir Oral Tablet [Emtricitabine / Tenofovir disoproxil Krka] | Branded Drug Form | Drug | RxNorm Extension |
| 43134209 | abacavir 600 MG / dolutegravir 50 MG / Lamivudine 300 MG Delayed Release Oral Tablet [Triumeq] Box of 30 | Branded Drug Box | Drug | RxNorm Extension |
| 41290902 | Lamivudine 150 MG / Zidovudine 300 MG Oral Tablet [Combivir] Box of 60 by Abacus Medicine | Marketed Product | Drug | RxNorm Extension |
| 41072413 | Lamivudine 100 MG Oral Tablet [Lamivudin Hexal] Box of 84 by Novartis | Marketed Product | Drug | RxNorm Extension |
| 35862178 | didanosine 100 MG [Hate] | Branded Drug Comp | Drug | RxNorm Extension |
| 1738171 | lopinavir 133 MG | Clinical Drug Comp | Drug | RxNorm |
| 40710120 | 230 ML maraviroc 20 MG/ML Oral Solution [Celsentri] by ViiV | Marketed Product | Drug | RxNorm Extension |
| 41264435 | Zidovudine 0.25 MG/ML [Retrovir] | Branded Drug Comp | Drug | RxNorm Extension |
| 21147823 | 144000 MG Nelfinavir 0.05 MG/MG Injectable Solution [Viracept] by Roche | Marketed Product | Drug | RxNorm Extension |
| 40746925 | efavirenz 600 MG / emtricitabine 200 MG / tenofovir disoproxil 250 MG Oral Tablet Box of 30 by Mylan | Marketed Product | Drug | RxNorm Extension |
| 1748955 | lopinavir 133 MG / ritonavir 33.3 MG Oral Capsule | Clinical Drug | Drug | RxNorm |
| 35860837 | ritonavir 100 MG [Aizhiwei] | Branded Drug Comp | Drug | RxNorm Extension |
| 36248986 | atazanavir Oral Powder Product | Clinical Dose Group | Drug | RxNorm |
| 44061790 | abacavir 600 MG / Lamivudine 300 MG Oral Tablet [Pms-Abacavir-Lamivudine] | Branded Drug | Drug | RxNorm Extension |
| 2031819 | 1 ML lamivudine 5 MG/ML Oral Suspension [Zeffix] | Quant Branded Drug | Drug | RxNorm Extension |
| 41412005 | 1.1 ML enfuvirtide 90 MG/ML Injection [Fuzeon] | Quant Branded Drug | Drug | RxNorm Extension |
| 40241982 | emtricitabine 200 MG / rilpivirine 25 MG / tenofovir disoproxil fumarate 300 MG Oral Tablet | Clinical Drug | Drug | RxNorm |
| 36505730 | dolutegravir 50 MG / Rilpivirine 25 MG Oral Tablet [Juluca] Box of 30 by Orifarm Leverkus | Marketed Product | Drug | RxNorm Extension |
| 42707688 | raltegravir 25 MG Chewable Tablet | Clinical Drug | Drug | RxNorm |
| 21141532 | Lamivudine 150 MG / Zidovudine 300 MG Oral Tablet Box of 60 by Milpharm | Marketed Product | Drug | RxNorm Extension |
| 42655678 | Nevirapine 400 MG Extended Release Oral Capsule Box of 30 by Mylan | Marketed Product | Drug | RxNorm Extension |
| 40162285 | delavirdine mesylate 100 MG | Clinical Drug Comp | Drug | RxNorm |
| 44175087 | fosamprenavir 700 MG Delayed Release Oral Tablet [Telzir] Box of 60 by Beragena | Marketed Product | Drug | RxNorm Extension |
| 21101476 | Nelfinavir 625 MG Oral Tablet Box of 120 | Clinical Drug Box | Drug | RxNorm Extension |
| 40746912 | emtricitabine 200 MG / tenofovir disoproxil 250 MG Oral Tablet Box of 30 by TEVA | Marketed Product | Drug | RxNorm Extension |
| 36882760 | emtricitabine / Tenofovir Oral Tablet | Clinical Drug Form | Drug | RxNorm Extension |
| 21131569 | Stavudine 30 MG Oral Capsule [Zerit] Box of 56 by Bristol Myers Squibb | Marketed Product | Drug | RxNorm Extension |
| 44108421 | Nevirapine Oral Tablet [Auro-Nevirapine] | Branded Drug Form | Drug | RxNorm Extension |
| 21072949 | Nevirapine 200 MG Oral Tablet Box of 60 by Creo | Marketed Product | Drug | RxNorm Extension |
| 43174017 | maraviroc 300 MG Delayed Release Oral Tablet | Clinical Drug | Drug | RxNorm Extension |
| 995225 | tenofovir disoproxil 250 MG Oral Tablet [Tenofovir Cipla] by Cipla | Marketed Product | Drug | RxNorm Extension |
| 43294619 | Nevirapine 200 MG [Nevipin] | Branded Drug Comp | Drug | RxNorm Extension |
| 43026171 | emtricitabine 200 MG / tenofovir disoproxil 250 MG Oral Tablet Box of 30 by KRKA | Marketed Product | Drug | RxNorm Extension |
| 1736996 | abacavir 20 MG/ML | Clinical Drug Comp | Drug | RxNorm |
| 19125940 | stavudine 37.5 MG [Zerit] | Branded Drug Comp | Drug | RxNorm |
| 35862180 | didanosine 50 MG Oral Granules | Clinical Drug | Drug | RxNorm Extension |
| 21055213 | Tenofovir / tenofovir disoproxil Oral Tablet | Clinical Drug Form | Drug | RxNorm Extension |
| 42902283 | darunavir 800 MG Oral Tablet [Prezista] | Branded Drug | Drug | RxNorm |
| 44161262 | Didanosine 1 MG/MG Oral Solution Box of 1 | Clinical Drug Box | Drug | RxNorm Extension |
| 44106041 | Lamivudine 300 MG [Auro-Lamivudine] | Branded Drug Comp | Drug | RxNorm Extension |
| 41190787 | Didanosine 50 MG Oral Tablet [Videx] Box of 60 | Branded Drug Box | Drug | RxNorm Extension |
| 36249786 | dolutegravir 25 MG Oral Tablet | Clinical Drug | Drug | RxNorm |
| 36239227 | Truvada Pill | Branded Dose Group | Drug | RxNorm |
| 43143367 | 240 ML Lamivudine 10 MG/ML Oral Solution Box of 1 | Quant Clinical Box | Drug | RxNorm Extension |
| 36062382 | efavirenz 600 MG / emtricitabine 200 MG / tenofovir disoproxil 250 MG Oral Tablet by Zentiva | Marketed Product | Drug | RxNorm Extension |
| 40990913 | Nelfinavir 250 MG Delayed Release Oral Tablet Box of 270 | Clinical Drug Box | Drug | RxNorm Extension |
| 35606589 | emtricitabine 167 MG / tenofovir disoproxil fumarate 250 MG Oral Tablet | Clinical Drug | Drug | RxNorm |
| 43268385 | ombitasvir 12.5 MG / paritaprevir 75 MG / Ritonavir 50 MG [Viekira Pak] | Branded Drug Comp | Drug | RxNorm Extension |
| 35788645 | 226 ML fosamprenavir 50 MG/ML Oral Suspension [Telzir] by ViiV | Marketed Product | Drug | RxNorm Extension |
| 2053067 | lamivudine Oral Tablet [HEPACT] | Branded Drug Form | Drug | RxNorm Extension |
| 43158158 | Saquinavir 500 MG Delayed Release Oral Tablet | Clinical Drug | Drug | RxNorm Extension |
| 43212907 | darunavir 800 MG Delayed Release Oral Tablet [Prezista] Box of 30 | Branded Drug Box | Drug | RxNorm Extension |
| 1748987 | lopinavir 100 MG / ritonavir 25 MG Oral Tablet [Kaletra] | Branded Drug | Drug | RxNorm |
| 35752148 | lopinavir 200 MG / Ritonavir 50 MG Delayed Release Oral Tablet [Kaletra] by Abbvie | Marketed Product | Drug | RxNorm Extension |
| 40874083 | Nevirapine 400 MG Extended Release Oral Tablet [Viramune] Box of 90 | Branded Drug Box | Drug | RxNorm Extension |
| 35603888 | enfuvirtide Injection | Clinical Drug Form | Drug | RxNorm |
| 36784667 | abacavir 600 MG / Lamivudine 300 MG Delayed Release Oral Tablet [Abacavir / Lamivudin Sandoz] Box of 30 | Branded Drug Box | Drug | RxNorm Extension |
| 40049811 | indinavir Oral Tablet | Clinical Drug Form | Drug | RxNorm |
| 36782726 | Nevirapine 400 MG Extended Release Oral Tablet [NEVIRAPINE SANDOZ] Box of 30 | Branded Drug Box | Drug | RxNorm Extension |
| 43138382 | raltegravir 400 MG Delayed Release Oral Tablet | Clinical Drug | Drug | RxNorm Extension |
| 40885494 | Lamivudine 100 MG Oral Tablet [Zeffix] Box of 84 by Aca Mueller | Marketed Product | Drug | RxNorm Extension |
| 36785878 | efavirenz 200 MG Delayed Release Oral Tablet [Stocrin] Box of 90 by MSD | Marketed Product | Drug | RxNorm Extension |
| 43713465 | Zidovudine 100 MG [Zidovudin Aurobindo] | Branded Drug Comp | Drug | RxNorm Extension |
| 1592273 | raltegravir 600 MG | Clinical Drug Comp | Drug | RxNorm |
| 41304479 | Didanosine 200 MG Extended Release Oral Capsule | Clinical Drug | Drug | RxNorm Extension |
| 40930058 | Didanosine 200 MG Oral Capsule | Clinical Drug | Drug | RxNorm Extension |
| 43291040 | maraviroc 300 MG Oral Tablet Box of 180 | Clinical Drug Box | Drug | RxNorm Extension |
| 37592144 | Nevirapine 400 MG Extended Release Oral Tablet [Nevirapin Hexal] by Novartis | Marketed Product | Drug | RxNorm Extension |
| 19098330 | tenofovir disoproxil fumarate 300 MG Oral Tablet [Viread] | Branded Drug | Drug | RxNorm |
| 41150671 | Amprenavir 15 MG/ML Oral Solution Box of 1 | Clinical Drug Box | Drug | RxNorm Extension |
| 1718489 | maraviroc Oral Solution [Selzentry] | Branded Drug Form | Drug | RxNorm |
| 43039286 | 2 ML enfuvirtide 90 MG/ML Injectable Solution [Fuzeon] | Quant Branded Drug | Drug | RxNorm Extension |
| 40996625 | Ritonavir 100 MG Delayed Release Oral Tablet [Norvir] Box of 90 | Branded Drug Box | Drug | RxNorm Extension |
| 40714440 | 20 ML Zidovudine 10 MG/ML Injectable Solution [Retrovir] Box of 5 by ViiV | Marketed Product | Drug | RxNorm Extension |
| 19038141 | efavirenz 100 MG [Sustiva] | Branded Drug Comp | Drug | RxNorm |
| 19091081 | darunavir 400 MG [Prezista] | Branded Drug Comp | Drug | RxNorm |
| 36238740 | Retrovir Oral Liquid Product | Branded Dose Group | Drug | RxNorm |
| 19122306 | abacavir 600 MG / lamivudine 300 MG Oral Tablet | Clinical Drug | Drug | RxNorm |
| 43293565 | efavirenz 30 MG/ML [Stocrin] | Branded Drug Comp | Drug | RxNorm Extension |
| 21063039 | Lamivudine 100 MG Oral Tablet Box of 28 by A A H | Marketed Product | Drug | RxNorm Extension |
| 40821843 | efavirenz 600 MG Delayed Release Oral Tablet [Efavirenz Aurobindo] Box of 90 | Branded Drug Box | Drug | RxNorm Extension |
| 43526311 | nevirapine 100 MG Extended Release Oral Tablet [Viramune] | Branded Drug | Drug | RxNorm |
| 40736230 | efavirenz 600 MG Oral Tablet Box of 30 by Mylan | Marketed Product | Drug | RxNorm Extension |
| 43149294 | raltegravir 400 MG Delayed Release Oral Tablet [Isentress] | Branded Drug | Drug | RxNorm Extension |
| 36788885 | Zidovudine 10 MG/ML Intravenous Solution Box of 5 | Clinical Drug Box | Drug | RxNorm Extension |
| 40978758 | Lamivudine 300 MG Oral Tablet [Epivir] Box of 30 by Eurim-Pharm | Marketed Product | Drug | RxNorm Extension |
| 43138732 | lopinavir 100 MG / Ritonavir 25 MG Delayed Release Oral Tablet [Kaletra] | Branded Drug | Drug | RxNorm Extension |
| 36062179 | tenofovir disoproxil 250 MG Delayed Release Oral Tablet by Macleods | Marketed Product | Drug | RxNorm Extension |
| 44076530 | efavirenz 600 MG Oral Tablet [Auro-Efavirenz] by Auro | Marketed Product | Drug | RxNorm Extension |
| 36242591 | Viracept Pill | Branded Dose Group | Drug | RxNorm |
| 43026156 | efavirenz 600 MG / emtricitabine 200 MG / tenofovir disoproxil 250 MG Oral Tablet Box of 30 by Biogaran | Marketed Product | Drug | RxNorm Extension |
| 21171507 | Didanosine 125 MG Delayed Release Oral Capsule Box of 30 | Clinical Drug Box | Drug | RxNorm Extension |
| 41169651 | abacavir 703 MG / Lamivudine 300 MG Oral Tablet [Kivexa] Box of 30 by Beragena | Marketed Product | Drug | RxNorm Extension |
| 36061608 | darunavir 600 MG Oral Tablet by Zentiva | Marketed Product | Drug | RxNorm Extension |
| 43256643 | Nevirapine 200 MG Oral Tablet Box of 60 by Alphapharm | Marketed Product | Drug | RxNorm Extension |
| 43180130 | darunavir 75 MG Delayed Release Oral Tablet Box of 480 | Clinical Drug Box | Drug | RxNorm Extension |
| 43155130 | Didanosine 400 MG Extended Release Oral Tablet [Videx] Box of 30 by Bristol Myers Squibb | Marketed Product | Drug | RxNorm Extension |
| 35862020 | zidovudine 10 MG/ML Injectable Solution [Weinuo] | Branded Drug | Drug | RxNorm Extension |
| 36779743 | 20 ML Zidovudine 10 MG/ML Intravenous Solution [Retrovir] Box of 5 | Quant Branded Box | Drug | RxNorm Extension |
| 21112306 | efavirenz 600 MG Oral Tablet [Sustiva] Box of 30 | Branded Drug Box | Drug | RxNorm Extension |
| 40950313 | Atazanavir 200 MG Oral Capsule [Reyataz] Box of 60 by Aca Mueller | Marketed Product | Drug | RxNorm Extension |
| 43133976 | efavirenz 600 MG Delayed Release Oral Tablet [EFAVIRENZ SANDOZ] | Branded Drug | Drug | RxNorm Extension |
| 21120401 | Saquinavir 200 MG Oral Capsule [Fortovase] Box of 180 by Roche | Marketed Product | Drug | RxNorm Extension |
| 35200460 | Delstrigo Oral Product | Branded Dose Group | Drug | RxNorm |
| 36889217 | Atazanavir 342 MG | Clinical Drug Comp | Drug | RxNorm Extension |
| 782826 | emtricitabine 200 MG / Tenofovir 245 MG Oral Tablet [Emtricitabine / Tenofovir disoproxil Mylan] | Branded Drug | Drug | RxNorm Extension |
| 36245658 | Viracept Oral Powder Product | Branded Dose Group | Drug | RxNorm |
| 40730827 | Lamivudine 100 MG Oral Tablet [Zeffix] by Sigma | Marketed Product | Drug | RxNorm Extension |
| 41136273 | etravirine 100 MG Oral Tablet [Intelence] Box of 120 by Gerke | Marketed Product | Drug | RxNorm Extension |
| 41435736 | 240 ML Nevirapine 0.0417 MG/ML Oral Solution | Quant Clinical Drug | Drug | RxNorm Extension |
| 41294029 | abacavir 351 MG / Lamivudine 150 MG / Zidovudine 300 MG Oral Tablet [Trizivir] Box of 60 by Bb Farma | Marketed Product | Drug | RxNorm Extension |
| 21100250 | cobicistat 150 MG / elvitegravir 150 MG / emtricitabine 200 MG / tenofovir disoproxil 250 MG Oral Tablet | Clinical Drug | Drug | RxNorm Extension |
| 41101949 | raltegravir 400 MG Oral Tablet [Isentress] Box of 180 by Merck | Marketed Product | Drug | RxNorm Extension |
| 21171050 | Lamivudine 100 MG Oral Tablet Box of 28 by TEVA | Marketed Product | Drug | RxNorm Extension |
| 36249080 | Odefsey Pill | Branded Dose Group | Drug | RxNorm |
| 43187468 | 240 ML abacavir 20 MG/ML Oral Solution Box of 1 | Quant Clinical Box | Drug | RxNorm Extension |
| 44178254 | raltegravir 400 MG Oral Tablet [Isentress] Box of 180 by Kohlpharma | Marketed Product | Drug | RxNorm Extension |
| 41404350 | 90 ML Ritonavir 0.889 MG/ML Oral Solution | Quant Clinical Drug | Drug | RxNorm Extension |
| 40746917 | emtricitabine 200 MG / tenofovir disoproxil 250 MG Oral Tablet by Aristo | Marketed Product | Drug | RxNorm Extension |
| 43522868 | {56 (dasabuvir 250 MG Oral Tablet) / 56 (ombitasvir 12.5 MG / paritaprevir 75 MG / Ritonavir 50 MG Oral Tablet) / 168 (Ribavirin 200 MG Oral Tablet) } Pack [Viekira Pak-Rbv] | Branded Pack | Drug | RxNorm Extension |
| 44205225 | 4030 MG Didanosine 1 MG/MG Oral Solution | Quant Clinical Drug | Drug | RxNorm Extension |
| 19124377 | darunavir 300 MG [Prezista] | Branded Drug Comp | Drug | RxNorm |
| 35859795 | nevirapine 200 MG Oral Tablet [Aitai] | Branded Drug | Drug | RxNorm Extension |
| 35411785 | raltegravir 100 MG Granules for Oral Suspension [Isentress] Box of 120 by Merck | Marketed Product | Drug | RxNorm Extension |
| 41135304 | Lamivudine 100 MG Oral Tablet [Zeffix] Box of 28 by Emra-Med | Marketed Product | Drug | RxNorm Extension |
| 41187846 | Lamivudine 0.0208 MG/ML Oral Solution [Zeffix] | Branded Drug | Drug | RxNorm Extension |
| 36248699 | cobicistat / darunavir Oral Product | Clinical Dose Group | Drug | RxNorm |
| 40971747 | Didanosine 200 MG Extended Release Oral Capsule [Videx] Box of 30 | Branded Drug Box | Drug | RxNorm Extension |
| 43151360 | Nevirapine 100 MG Oral Tablet Box of 90 | Clinical Drug Box | Drug | RxNorm Extension |
| 19123440 | didanosine 400 MG Delayed Release Oral Capsule [Viden] | Branded Drug | Drug | RxNorm |
| 19022896 | lamivudine 150 MG / zidovudine 300 MG Oral Tablet | Clinical Drug | Drug | RxNorm |
| 19045249 | fosamprenavir 50 MG/ML [Lexiva] | Branded Drug Comp | Drug | RxNorm |
| 40918006 | darunavir 800 MG Oral Tablet [Prezista] Box of 30 by Abacus Medicine | Marketed Product | Drug | RxNorm Extension |
| 43180074 | emtricitabine 200 MG / Rilpivirine 25 MG / tenofovir disoproxil 250 MG Delayed Release Oral Tablet [Eviplera] | Branded Drug | Drug | RxNorm Extension |
| 41197044 | Nevirapine 400 MG Extended Release Oral Tablet [Viramune] Box of 30 by Gerke | Marketed Product | Drug | RxNorm Extension |
| 42656090 | emtricitabine 200 MG / Tenofovir 245 MG Oral Tablet Box of 30 by Mylan | Marketed Product | Drug | RxNorm Extension |
| 36224276 | tipranavir Oral Liquid Product | Clinical Dose Group | Drug | RxNorm |
| 21157929 | 240 ML Lamivudine 5 MG/ML Oral Solution [Zeffix] by Glaxosmithkline | Marketed Product | Drug | RxNorm Extension |
| 42543870 | lamivudine / tenofovir disoproxil Pill | Clinical Dose Group | Drug | RxNorm |
| 43163199 | fosamprenavir Delayed Release Oral Tablet | Clinical Drug Form | Drug | RxNorm Extension |
| 43200283 | Lamivudine Delayed Release Oral Tablet [Epivir] | Branded Drug Form | Drug | RxNorm Extension |
| 43180173 | tenofovir disoproxil 200 MG Delayed Release Oral Tablet [Viread] Box of 30 by Gilead | Marketed Product | Drug | RxNorm Extension |
| 43189263 | Lamivudine / Zidovudine Delayed Release Oral Tablet | Clinical Drug Form | Drug | RxNorm Extension |
| 41502054 | 60000 MG tenofovir disoproxil 0.04 MG/MG Oral Powder | Quant Clinical Drug | Drug | RxNorm Extension |
| 21118438 | 200 ML Zidovudine 10 MG/ML Oral Solution [Retrovir] by ViiV | Marketed Product | Drug | RxNorm Extension |
| 43185065 | fosamprenavir 700 MG Delayed Release Oral Tablet [Telzir] Box of 60 | Branded Drug Box | Drug | RxNorm Extension |
| 21131570 | Stavudine 40 MG Oral Capsule Box of 56 | Clinical Drug Box | Drug | RxNorm Extension |
| 40165045 | nelfinavir Oral Powder [Viracept] | Branded Drug Form | Drug | RxNorm |
| 35773215 | Lamivudine 100 MG Delayed Release Oral Tablet [Zeffix] by Glaxosmithkline | Marketed Product | Drug | RxNorm Extension |
| 36271282 | Lamivudine 150 MG / Zidovudine 300 MG Oral Tablet [LAMIVUDINE/ZIDOVUDINE MYLAN] Box of 60 by Mylan | Marketed Product | Drug | RxNorm Extension |
| 43026751 | DORAVIRINE / Lamivudine / tenofovir disoproxil Delayed Release Oral Tablet | Clinical Drug Form | Drug | RxNorm Extension |
| 36784670 | abacavir / Lamivudine Oral Tablet [Abacavir / Lamivudin Sandoz] | Branded Drug Form | Drug | RxNorm Extension |
| 19125469 | lamivudine 150 MG / nevirapine 200 MG / zidovudine 300 MG Oral Tablet | Clinical Drug | Drug | RxNorm |
| 2052591 | lamivudine 100 MG [LAMIDINE] | Branded Drug Comp | Drug | RxNorm Extension |
| 44196810 | 95 ML tipranavir 100 MG/ML Oral Solution [Aptivus] Box of 1 | Quant Branded Box | Drug | RxNorm Extension |
| 21149333 | Atazanavir 300 MG / cobicistat 150 MG Oral Tablet Box of 30 | Clinical Drug Box | Drug | RxNorm Extension |
| 36238998 | Zerit Oral Liquid Product | Branded Dose Group | Drug | RxNorm |
| 782836 | efavirenz / emtricitabine / tenofovir disoproxil Oral Tablet [Efavirenz / Emtricitabine / Tenofovir disoproxil Mylan] | Branded Drug Form | Drug | RxNorm Extension |
| 41270318 | Zidovudine 300 MG Oral Tablet Box of 60 | Clinical Drug Box | Drug | RxNorm Extension |
| 40733519 | maraviroc Oral Solution [Celsentri] | Branded Drug Form | Drug | RxNorm Extension |
| 21112608 | Didanosine 200 MG Delayed Release Oral Capsule Box of 30 | Clinical Drug Box | Drug | RxNorm Extension |
| 41221906 | Didanosine 125 MG Extended Release Oral Capsule [Videx] | Branded Drug | Drug | RxNorm Extension |
| 21072940 | Ritonavir 100 MG Oral Tablet Box of 30 | Clinical Drug Box | Drug | RxNorm Extension |
| 36784660 | abacavir 600 MG / Lamivudine 300 MG Oral Tablet [Abacavir / Lamivudin Sandoz] Box of 30 by Sandoz | Marketed Product | Drug | RxNorm Extension |
| 19102250 | fosamprenavir 700 MG | Clinical Drug Comp | Drug | RxNorm |
| 35415360 | 226 ML fosamprenavir 50 MG/ML Oral Suspension [Telzir] | Quant Branded Drug | Drug | RxNorm Extension |
| 21147041 | 20 ML Zidovudine 10 MG/ML Injection [Retrovir] Box of 5 by ViiV | Marketed Product | Drug | RxNorm Extension |
| 40851568 | tenofovir disoproxil 250 MG Oral Tablet [Viread] Box of 30 by Haematogmbh | Marketed Product | Drug | RxNorm Extension |
| 36404012 | Ritonavir 100 MG Oral Capsule [Norvir] Box of 84 by Abbott | Marketed Product | Drug | RxNorm Extension |
| 21069538 | 240 ML Lamivudine 5 MG/ML Oral Solution [Zeffix] | Quant Branded Drug | Drug | RxNorm Extension |
| 21144478 | maraviroc Oral Tablet [Celsentri] | Branded Drug Form | Drug | RxNorm Extension |
| 21072947 | Nevirapine 400 MG Extended Release Oral Tablet [Viramune] Box of 30 | Branded Drug Box | Drug | RxNorm Extension |
| 43189254 | Lamivudine 150 MG Delayed Release Oral Tablet [Epivir] Box of 60 by ViiV | Marketed Product | Drug | RxNorm Extension |
| 36062303 | lamivudine 100 MG Oral Tablet [Zeffix] Box of 28 by CST | Marketed Product | Drug | RxNorm Extension |
| 37592830 | dolutegravir 50 MG / Rilpivirine 25 MG Oral Tablet [Juluca] Box of 90 by ViiV | Marketed Product | Drug | RxNorm Extension |
| 44186404 | emtricitabine 200 MG / Rilpivirine 25 MG / tenofovir disoproxil 300 MG Oral Tablet [Eviplera] Box of 90 by Canoma | Marketed Product | Drug | RxNorm Extension |
| 43026143 | bictegravir 50 MG / emtricitabine 200 MG / tenofovir alafenamide 25 MG Delayed Release Oral Tablet Box of 30 | Clinical Drug Box | Drug | RxNorm Extension |
| 19113114 | didanosine 250 MG Extended Release Oral Capsule | Clinical Drug | Drug | RxNorm |
| 21033498 | Lamivudine 100 MG Oral Tablet Box of 28 by Creo | Marketed Product | Drug | RxNorm Extension |
| 40930180 | darunavir 600 MG Oral Tablet Box of 90 | Clinical Drug Box | Drug | RxNorm Extension |
| 40879056 | cobicistat 150 MG Oral Tablet [Tybost] Box of 90 | Branded Drug Box | Drug | RxNorm Extension |
| 41430784 | 180 ML efavirenz 0.167 MG/ML Oral Solution [Sustiva] | Quant Branded Drug | Drug | RxNorm Extension |
| 35860787 | lopinavir Oral Tablet [Kelizhi] | Branded Drug Form | Drug | RxNorm Extension |
| 21122327 | Zidovudine 250 MG Oral Capsule Box of 60 by A A H | Marketed Product | Drug | RxNorm Extension |
| 19027669 | 20 ML zidovudine 10 MG/ML Injection | Quant Clinical Drug | Drug | RxNorm |
| 35860452 | ritonavir 25 MG Oral Tablet [Kelizhi] | Branded Drug | Drug | RxNorm Extension |
| 44045755 | enfuvirtide 108 MG Injectable Solution | Clinical Drug | Drug | RxNorm Extension |
| 1511230 | lamivudine / tenofovir disoproxil Oral Tablet [Temixys] | Branded Drug Form | Drug | RxNorm |
| 40855382 | efavirenz 600 MG / emtricitabine 200 MG / tenofovir disoproxil 300 MG Oral Tablet [Atripla] Box of 30 by Aaston | Marketed Product | Drug | RxNorm Extension |
| 43219711 | 90 ML Ritonavir 80 MG/ML Oral Solution Box of 5 | Quant Clinical Box | Drug | RxNorm Extension |
| 21131574 | lopinavir 100 MG / Ritonavir 25 MG Oral Tablet [Kaletra] Box of 60 by Abbvie | Marketed Product | Drug | RxNorm Extension |
| 1738162 | efavirenz 100 MG | Clinical Drug Comp | Drug | RxNorm |
| 41168002 | Didanosine 250 MG Oral Capsule [Videx] Box of 60 by Kohlpharma | Marketed Product | Drug | RxNorm Extension |
| 44044297 | efavirenz Oral Tablet [EFAVIRENZ MYLAN] | Branded Drug Form | Drug | RxNorm Extension |
| 43144916 | efavirenz 600 MG Delayed Release Oral Tablet [Sustiva] Box of 30 | Branded Drug Box | Drug | RxNorm Extension |
| 40234303 | etravirine 200 MG [Intelence] | Branded Drug Comp | Drug | RxNorm |
| 41103003 | Nevirapine 200 MG Oral Tablet [Viramune] Box of 60 by Gerke | Marketed Product | Drug | RxNorm Extension |
| 41435748 | 240 ML Nevirapine 10 MG/ML Oral Solution [Viramune] Box of 1 | Quant Branded Box | Drug | RxNorm Extension |
| 44129866 | abacavir 600 MG / Lamivudine 300 MG Oral Tablet [Apo-Abacavir-Lamivudine] by Apotex | Marketed Product | Drug | RxNorm Extension |
| 42918909 | 240 ML Ritonavir 80 MG/ML Oral Solution | Quant Clinical Drug | Drug | RxNorm Extension |
| 19125938 | stavudine 100 MG [Zerit] | Branded Drug Comp | Drug | RxNorm |
| 37592828 | dolutegravir 50 MG / Rilpivirine 25 MG Oral Tablet Box of 90 | Clinical Drug Box | Drug | RxNorm Extension |
| 2052718 | zidovudine 250 MG Oral Capsule [AZIDOMINE] | Branded Drug | Drug | RxNorm Extension |
| 41089766 | Stavudine 1 MG/ML Oral Suspension [Zerit] | Branded Drug | Drug | RxNorm Extension |
| 43134211 | Lamivudine 150 MG / Zidovudine 300 MG Delayed Release Oral Tablet [LAMIVUDINE/ZIDOVUDINE MYLAN] Box of 60 | Branded Drug Box | Drug | RxNorm Extension |
| 42874705 | raltegravir 400 MG Oral Tablet [Isentress] Box of 180 by Aaha | Marketed Product | Drug | RxNorm Extension |
| 43195889 | maraviroc 150 MG Delayed Release Oral Tablet Box of 60 | Clinical Drug Box | Drug | RxNorm Extension |
| 44111050 | Zidovudine 100 MG Oral Capsule [Apo-Zidovudine] | Branded Drug | Drug | RxNorm Extension |
| 44121653 | Lamivudine Oral Tablet [Apo-Lamivudine] | Branded Drug Form | Drug | RxNorm Extension |
| 41003135 | Didanosine 200 MG Oral Tablet [Videx] Box of 60 | Branded Drug Box | Drug | RxNorm Extension |
| 43133085 | Didanosine 125 MG Extended Release Oral Tablet [Videx] | Branded Drug | Drug | RxNorm Extension |
| 41239851 | tenofovir disoproxil 250 MG Delayed Release Oral Tablet Box of 90 | Clinical Drug Box | Drug | RxNorm Extension |
| 43270867 | cobicistat 150 MG / darunavir 800 MG Oral Tablet [Prezcobix] Box of 30 | Branded Drug Box | Drug | RxNorm Extension |
| 40947772 | Lamivudine 150 MG / Zidovudine 300 MG Delayed Release Oral Tablet Box of 60 by Aurobindo | Marketed Product | Drug | RxNorm Extension |
| 21023722 | lopinavir 200 MG / Ritonavir 50 MG Oral Tablet [Kaletra] Box of 120 by Abbvie | Marketed Product | Drug | RxNorm Extension |
| 21151341 | Lamivudine 300 MG Oral Tablet [Epivir] Box of 30 by ViiV | Marketed Product | Drug | RxNorm Extension |
| 43261494 | Lamivudine 150 MG / Zidovudine 300 MG Oral Tablet by Alphapharm | Marketed Product | Drug | RxNorm Extension |
| 2053047 | lamivudine 100 MG Oral Tablet [HEPTOLIVER] | Branded Drug | Drug | RxNorm Extension |
| 41306667 | Zidovudine 250 MG Oral Capsule [Retrovir] Box of 80 | Branded Drug Box | Drug | RxNorm Extension |
| 35200465 | doravirine / lamivudine / tenofovir disoproxil Oral Tablet [Delstrigo] | Branded Drug Form | Drug | RxNorm |
| 21069464 | 180 ML efavirenz 30 MG/ML Oral Solution [Sustiva] | Quant Branded Drug | Drug | RxNorm Extension |
| 36248702 | Prezcobix Pill | Branded Dose Group | Drug | RxNorm |
| 36783451 | cobicistat / darunavir Delayed Release Oral Tablet | Clinical Drug Form | Drug | RxNorm Extension |
| 42927739 | tenofovir disoproxil 250 MG [TENOLID] | Branded Drug Comp | Drug | RxNorm Extension |
| 42927758 | tenofovir disoproxil Oral Tablet [PREAD] | Branded Drug Form | Drug | RxNorm Extension |
| 36061604 | darunavir 600 MG Oral Tablet by A A H | Marketed Product | Drug | RxNorm Extension |
| 40010553 | atazanavir Oral Capsule [Reyataz] | Branded Drug Form | Drug | RxNorm |
| 19121070 | fosamprenavir 700 MG [Lexiva] | Branded Drug Comp | Drug | RxNorm |
| 40730819 | abacavir 600 MG / Lamivudine 300 MG Oral Tablet by TEVA | Marketed Product | Drug | RxNorm Extension |
| 42708114 | tenofovir disoproxil fumarate 150 MG Oral Tablet | Clinical Drug | Drug | RxNorm |
| 21092413 | Ritonavir 80 MG/ML Oral Solution by Abbvie | Marketed Product | Drug | RxNorm Extension |
| 45892974 | cobicistat 150 MG / darunavir 800 MG Oral Tablet | Clinical Drug | Drug | RxNorm |
| 41037971 | Zidovudine 250 MG Oral Capsule [Retrovir] Box of 40 by Aca Mueller | Marketed Product | Drug | RxNorm Extension |
| 2011837 | tenofovir disoproxil 300 MG Oral Tablet [TENOLID-F] | Branded Drug | Drug | RxNorm Extension |
| 43156112 | efavirenz 30 MG/ML Oral Solution [Sustiva] Box of 1 | Branded Drug Box | Drug | RxNorm Extension |
| 782438 | darunavir 800 MG [Darunavir Krka] | Branded Drug Comp | Drug | RxNorm Extension |
| 42874953 | Nevirapine Delayed Release Oral Tablet | Clinical Drug Form | Drug | RxNorm Extension |
| 40928353 | Nevirapine 10 MG/ML Oral Solution Box of 1 | Clinical Drug Box | Drug | RxNorm Extension |
| 41234056 | Ritonavir 0.889 MG/ML [Norvir] | Branded Drug Comp | Drug | RxNorm Extension |
| 36074451 | abacavir 300 MG Delayed Release Oral Tablet Box of 60 by Dr Reddy's | Marketed Product | Drug | RxNorm Extension |
| 40917580 | emtricitabine 200 MG / tenofovir disoproxil 300 MG Oral Tablet [Truvada] Box of 30 by Axicorp | Marketed Product | Drug | RxNorm Extension |
| 21145007 | Rilpivirine 25 MG Oral Tablet [Edurant] Box of 30 | Branded Drug Box | Drug | RxNorm Extension |
| 43860998 | 2000 MG Didanosine 1 MG/MG Oral Powder | Quant Clinical Drug | Drug | RxNorm Extension |
| 41280887 | Lamivudine 150 MG Delayed Release Oral Tablet [Lamivudin Aurobindo] Box of 30 | Branded Drug Box | Drug | RxNorm Extension |
| 41083974 | Ritonavir 0.889 MG/ML Oral Solution Box of 5 | Clinical Drug Box | Drug | RxNorm Extension |
| 41132956 | Saquinavir 500 MG Oral Tablet [Invirase] Box of 120 by Aca Mueller | Marketed Product | Drug | RxNorm Extension |
| 43211097 | Lamivudine 300 MG Delayed Release Oral Tablet [Epivir] Box of 30 by ViiV | Marketed Product | Drug | RxNorm Extension |
| 41435058 | 200 ML darunavir 0.5 MG/ML Oral Solution [Prezista] Box of 1 | Quant Branded Box | Drug | RxNorm Extension |
| 1710612 | zidovudine | Ingredient | Drug | RxNorm |
| 19129965 | darunavir 600 MG Oral Tablet [Prezista] | Branded Drug | Drug | RxNorm |
| 1758537 | etravirine 100 MG | Clinical Drug Comp | Drug | RxNorm |
| 44067769 | emtricitabine 200 MG / tenofovir alafenamide 10 MG [Descovy] | Branded Drug Comp | Drug | RxNorm Extension |
| 43146977 | darunavir 300 MG Delayed Release Oral Tablet | Clinical Drug | Drug | RxNorm Extension |
| 44119366 | efavirenz 600 MG [Auro-Efavirenz] | Branded Drug Comp | Drug | RxNorm Extension |
| 40825250 | Lamivudine 300 MG Oral Tablet [LAMIVUDINE MYLAN] Box of 30 | Branded Drug Box | Drug | RxNorm Extension |
| 36249552 | emtricitabine / tenofovir alafenamide Oral Product | Clinical Dose Group | Drug | RxNorm |
| 36219237 | zidovudine Pill | Clinical Dose Group | Drug | RxNorm |
| 35858570 | stavudine Inhalation Powder | Clinical Drug Form | Drug | RxNorm Extension |
| 35415358 | 226 ML fosamprenavir 50 MG/ML Oral Suspension [Telzir] Box of 1 | Quant Branded Box | Drug | RxNorm Extension |
| 1724993 | didanosine 200 MG Chewable Tablet | Clinical Drug | Drug | RxNorm |
| 44065245 | abacavir 300 MG / Lamivudine 150 MG / Zidovudine 300 MG Oral Tablet [Trizivir] by ViiV | Marketed Product | Drug | RxNorm Extension |
| 21154304 | darunavir 75 MG Oral Tablet Box of 480 | Clinical Drug Box | Drug | RxNorm Extension |
| 21134119 | enfuvirtide 108 MG Injection | Clinical Drug | Drug | RxNorm Extension |
| 21169052 | cobicistat 150 MG / elvitegravir 150 MG / emtricitabine 200 MG / Tenofovir 10 MG Oral Tablet | Clinical Drug | Drug | RxNorm Extension |
| 36407004 | raltegravir 25 MG Oral Tablet [Isentress] Box of 60 by Merck | Marketed Product | Drug | RxNorm Extension |
| 2052770 | tenofovir disoproxil 300 MG [VIRIVA] | Branded Drug Comp | Drug | RxNorm Extension |
| 41179980 | enfuvirtide 90 MG/ML Injectable Solution Box of 60 | Clinical Drug Box | Drug | RxNorm Extension |
| 964218 | efavirenz 400 MG / lamivudine 300 MG / tenofovir disoproxil fumarate 300 MG Oral Tablet | Clinical Drug | Drug | RxNorm |
| 35862764 | lamivudine 10 MG/ML [Yipingwei] | Branded Drug Comp | Drug | RxNorm Extension |
| 40238459 | nevirapine 400 MG Extended Release Oral Tablet [Viramune] | Branded Drug | Drug | RxNorm |
| 41206774 | Nevirapine Oral Tablet [Nevirapin Hormosan] | Branded Drug Form | Drug | RxNorm Extension |
| 43212951 | tenofovir disoproxil 123 MG Delayed Release Oral Tablet | Clinical Drug | Drug | RxNorm Extension |
| 43149972 | cobicistat / elvitegravir / emtricitabine / tenofovir alafenamide Delayed Release Oral Tablet | Clinical Drug Form | Drug | RxNorm Extension |
| 41135083 | lopinavir 200 MG / Ritonavir 50 MG Delayed Release Oral Tablet [Kaletra] Box of 360 by European | Marketed Product | Drug | RxNorm Extension |
| 21026702 | maraviroc 150 MG [Celsentri] | Branded Drug Comp | Drug | RxNorm Extension |
| 21164103 | darunavir 150 MG Oral Tablet [Prezista] Box of 240 | Branded Drug Box | Drug | RxNorm Extension |
| 19122565 | tipranavir 250 MG Oral Capsule [Aptivus] | Branded Drug | Drug | RxNorm |
| 41013502 | Ritonavir 0.889 MG/ML | Clinical Drug Comp | Drug | RxNorm Extension |
| 41432040 | 200 ML Stavudine 1 MG/ML Oral Suspension Box of 1 | Quant Clinical Box | Drug | RxNorm Extension |
| 42927714 | tenofovir disoproxil 250 MG Oral Tablet [PREAD] | Branded Drug | Drug | RxNorm Extension |
| 41165668 | Nevirapine 400 MG Extended Release Oral Tablet [Viramune] Box of 90 by Gerke | Marketed Product | Drug | RxNorm Extension |
| 36062174 | tenofovir disoproxil 250 MG Delayed Release Oral Tablet Box of 30 by A A H | Marketed Product | Drug | RxNorm Extension |
| 35750794 | Rilpivirine 25 MG Delayed Release Oral Tablet [Edurant] by Janssen | Marketed Product | Drug | RxNorm Extension |
| 36420271 | maraviroc 20 MG/ML Oral Solution [Celsentri] Box of 1 | Branded Drug Box | Drug | RxNorm Extension |
| 36783449 | cobicistat 150 MG / darunavir 800 MG Delayed Release Oral Tablet | Clinical Drug | Drug | RxNorm Extension |
| 40127987 | emtricitabine Oral Solution | Clinical Drug Form | Drug | RxNorm |
| 21151898 | Zidovudine 100 MG Oral Capsule [Retrovir] Box of 100 | Branded Drug Box | Drug | RxNorm Extension |
| 35760526 | Lamivudine 100 MG Delayed Release Oral Tablet Box of 28 by Sandoz | Marketed Product | Drug | RxNorm Extension |
| 41054831 | Didanosine 1 MG/MG Oral Suspension | Clinical Drug | Drug | RxNorm Extension |
| 36788886 | Zidovudine 10 MG/ML Intravenous Solution | Clinical Drug | Drug | RxNorm Extension |
| 40720638 | tenofovir disoproxil 250 MG Oral Tablet by Aristo | Marketed Product | Drug | RxNorm Extension |
| 43522795 | {56 (dasabuvir 250 MG Oral Tablet) / 56 (ombitasvir 12.5 MG / paritaprevir 75 MG / Ritonavir 50 MG Oral Tablet) / 56 (Ribavirin 600 MG Oral Tablet) } Pack [Viekira Pak-Rbv] box of 1 by Abbvie | Marketed Product | Drug | RxNorm Extension |
| 43258430 | maraviroc 150 MG Oral Tablet [Celsentri] Box of 180 | Branded Drug Box | Drug | RxNorm Extension |
| 782839 | efavirenz 600 MG / emtricitabine 200 MG / tenofovir disoproxil 250 MG Oral Tablet [Efavirenz / Emtricitabine / Tenofovir disoproxil Krka] Box of 30 | Branded Drug Box | Drug | RxNorm Extension |
| 40955101 | Nelfinavir Oral Solution | Clinical Drug Form | Drug | RxNorm Extension |
| 2052765 | tenofovir disoproxil 300 MG [TENOFOVIL] | Branded Drug Comp | Drug | RxNorm Extension |
| 41280885 | Lamivudine 300 MG Delayed Release Oral Tablet [Lamivudin Hexal] | Branded Drug | Drug | RxNorm Extension |
| 35743671 | efavirenz 600 MG Delayed Release Oral Tablet by Creo | Marketed Product | Drug | RxNorm Extension |
| 43589260 | tenofovir disoproxil 300 MG [Tenofovirdisoproxil-Ratiopharm] | Branded Drug Comp | Drug | RxNorm Extension |
| 36261384 | abacavir 600 MG / Lamivudine 300 MG Oral Tablet [Mylan-Abacavir/Lamivudine] Box of 30 by Mylan | Marketed Product | Drug | RxNorm Extension |
| 35858569 | stavudine Oral Tablet [Maisiting] | Branded Drug Form | Drug | RxNorm Extension |
| 35411994 | efavirenz 600 MG [EFAVIRENZ CRISTERS] | Branded Drug Comp | Drug | RxNorm Extension |
| 40945509 | Saquinavir 500 MG Delayed Release Oral Tablet [Invirase] Box of 120 by Beragena | Marketed Product | Drug | RxNorm Extension |
| 42967484 | Zidovudine 100 MG Oral Capsule [AZIDOMINE] | Branded Drug | Drug | RxNorm Extension |
| 19065536 | didanosine 10 MG/ML Oral Solution | Clinical Drug | Drug | RxNorm |
| 1725064 | didanosine 1 MG/ML | Clinical Drug Comp | Drug | RxNorm |
| 36267870 | Nevirapine 400 MG Extended Release Oral Capsule [Viramune] | Branded Drug | Drug | RxNorm Extension |
| 35410895 | efavirenz 600 MG Delayed Release Oral Tablet [Efavirenz Teva] Box of 30 | Branded Drug Box | Drug | RxNorm Extension |
| 19041909 | atazanavir 300 MG [Reyataz] | Branded Drug Comp | Drug | RxNorm |
| 36249079 | Odefsey Oral Product | Branded Dose Group | Drug | RxNorm |
| 41315122 | Didanosine 1 MG/MG Oral Suspension [Videx] Box of 1 | Branded Drug Box | Drug | RxNorm Extension |
| 40948924 | emtricitabine 200 MG / tenofovir disoproxil 300 MG Oral Tablet [Truvada] Box of 30 by Orifarm Leverkus | Marketed Product | Drug | RxNorm Extension |
| 44048810 | abacavir 600 MG / Lamivudine 300 MG Oral Tablet [Apo-Abacavir-Lamivudine] | Branded Drug | Drug | RxNorm Extension |
| 40730838 | Lamivudine 300 MG Oral Tablet Box of 30 by Milpharm | Marketed Product | Drug | RxNorm Extension |
| 45892117 | atazanavir / cobicistat Oral Tablet [Evotaz] | Branded Drug Form | Drug | RxNorm |
| 41464361 | 2020 MG Didanosine 1 MG/MG Oral Suspension [Videx] Box of 1 | Quant Branded Box | Drug | RxNorm Extension |
| 21082608 | Lamivudine 150 MG / Zidovudine 300 MG Oral Tablet Box of 60 | Clinical Drug Box | Drug | RxNorm Extension |
| 40909781 | darunavir 800 MG Oral Tablet [Prezista] Box of 90 | Branded Drug Box | Drug | RxNorm Extension |
| 1725063 | didanosine 0.847 MG/ML | Clinical Drug Comp | Drug | RxNorm |
| 43515541 | tenofovir disoproxil 300 MG Oral Tablet Box of 30 | Clinical Drug Box | Drug | RxNorm Extension |
| 21082831 | efavirenz 600 MG Oral Tablet [Sustiva] by Bristol Myers Squibb | Marketed Product | Drug | RxNorm Extension |
| 35858553 | stavudine 40 MG Oral Tablet | Clinical Drug | Drug | RxNorm Extension |
| 21102497 | efavirenz 600 MG / emtricitabine 200 MG / tenofovir disoproxil 250 MG Oral Tablet [Atripla] Box of 30 | Branded Drug Box | Drug | RxNorm Extension |
| 43840728 | efavirenz Oral Tablet [Efavirenz Accord] | Branded Drug Form | Drug | RxNorm Extension |
| 35862767 | lamivudine Oral Tablet [Keweizi] | Branded Drug Form | Drug | RxNorm Extension |
| 43290082 | Ritonavir 100 MG Oral Tablet [Norvir] Box of 30 | Branded Drug Box | Drug | RxNorm Extension |
| 44106040 | Lamivudine 5 MG/ML [Heptovir] | Branded Drug Comp | Drug | RxNorm Extension |
| 43291041 | maraviroc 150 MG Oral Tablet Box of 30 | Clinical Drug Box | Drug | RxNorm Extension |
| 40977858 | Nevirapine 400 MG Extended Release Oral Tablet [Viramune] Box of 30 by Medicopharm | Marketed Product | Drug | RxNorm Extension |
| 19123901 | nelfinavir 200 MG Oral Tablet [Viracept] | Branded Drug | Drug | RxNorm |
| 40753344 | Nevirapine 200 MG Oral Tablet Box of 60 by Milpharm | Marketed Product | Drug | RxNorm Extension |
| 1592433 | ritonavir Oral Powder Product | Clinical Dose Group | Drug | RxNorm |
| 19124374 | efavirenz 600 MG / emtricitabine 200 MG / tenofovir disoproxil fumarate 300 MG Oral Tablet [Atripla] | Branded Drug | Drug | RxNorm |
| 2052776 | tenofovir disoproxil 250 MG Oral Tablet [TENOF] by Eden Parma | Marketed Product | Drug | RxNorm Extension |
| 35409733 | efavirenz Delayed Release Oral Tablet [Efavirenz Teva] | Branded Drug Form | Drug | RxNorm Extension |
| 43262883 | ombitasvir 12.5 MG / paritaprevir 75 MG / Ritonavir 50 MG Oral Tablet [Viekira Pak] | Branded Drug | Drug | RxNorm Extension |
| 40918004 | darunavir 600 MG Oral Tablet [Prezista] Box of 60 by Gerke | Marketed Product | Drug | RxNorm Extension |
| 43211100 | abacavir / dolutegravir / Lamivudine Delayed Release Oral Tablet | Clinical Drug Form | Drug | RxNorm Extension |
| 41435685 | 226 ML fosamprenavir 0.222 MG/ML Oral Solution [Telzir] Box of 1 | Quant Branded Box | Drug | RxNorm Extension |
| 40945667 | Ritonavir 100 MG Oral Capsule [Norvir] Box of 84 by Eurim-Pharm | Marketed Product | Drug | RxNorm Extension |
| 44057005 | Lamivudine / Nevirapine / Zidovudine Oral Tablet [Apo-Zidovudine-Lamivudine-Nevirapine] | Branded Drug Form | Drug | RxNorm Extension |
| 44086573 | Didanosine 4000 MG Oral Powder [Videx Pediatric] | Branded Drug | Drug | RxNorm Extension |
| 41290665 | lopinavir 200 MG / Ritonavir 50 MG Oral Tablet [Kaletra] Box of 360 by European | Marketed Product | Drug | RxNorm Extension |
| 44070910 | abacavir / Lamivudine Oral Tablet [Auro-Abacavir/Lamivudine] | Branded Drug Form | Drug | RxNorm Extension |
| 40708526 | 1000 MG tenofovir disoproxil 0.033 MG/MG Oral Granules [Viread] | Quant Branded Drug | Drug | RxNorm Extension |
| 42543878 | lamivudine 300 MG / tenofovir disoproxil fumarate 300 MG Oral Tablet [Cimduo] | Branded Drug | Drug | RxNorm |
| 40886688 | efavirenz 600 MG / emtricitabine 200 MG / tenofovir disoproxil 300 MG Oral Tablet [Atripla] Box of 30 by Kohlpharma | Marketed Product | Drug | RxNorm Extension |
| 41199572 | darunavir 400 MG Delayed Release Oral Tablet [Prezista] Box of 60 by Aca Mueller | Marketed Product | Drug | RxNorm Extension |
| 36407765 | raltegravir 100 MG Oral Tablet [Isentress] by Merck | Marketed Product | Drug | RxNorm Extension |
| 44071629 | Didanosine 50 MG Oral Tablet | Clinical Drug | Drug | RxNorm Extension |
| 1738202 | lopinavir 80 MG/ML | Clinical Drug Comp | Drug | RxNorm |
| 35859812 | nevirapine 200 MG [Weilesi] | Branded Drug Comp | Drug | RxNorm Extension |
| 21033960 | Didanosine 200 MG Delayed Release Oral Capsule [Videx EC] Box of 30 by Bristol Myers Squibb | Marketed Product | Drug | RxNorm Extension |
| 40976777 | raltegravir 400 MG Oral Tablet [Isentress] Box of 60 by Veron | Marketed Product | Drug | RxNorm Extension |
| 40720643 | tenofovir disoproxil 250 MG Oral Tablet by Mylan | Marketed Product | Drug | RxNorm Extension |
| 35408211 | raltegravir 100 MG Granules for Oral Suspension Box of 120 | Clinical Drug Box | Drug | RxNorm Extension |
| 36785885 | efavirenz 600 MG Delayed Release Oral Tablet [Stocrin] Box of 30 by PI-Pharma | Marketed Product | Drug | RxNorm Extension |
| 41164638 | raltegravir 400 MG Oral Tablet [Isentress] Box of 60 by Eurim-Pharm | Marketed Product | Drug | RxNorm Extension |
| 1727223 | atazanavir | Ingredient | Drug | RxNorm |
| 36259364 | Lamivudine / Zidovudine Oral Tablet [LAMIVUDINE/ZIDOVUDINE MYLAN] | Branded Drug Form | Drug | RxNorm Extension |
| 43143990 | Didanosine 200 MG Extended Release Oral Tablet | Clinical Drug | Drug | RxNorm Extension |
| 587054 | Zidovudine 10 MG/ML Injection Box of 10 | Clinical Drug Box | Drug | RxNorm Extension |
| 43193750 | Ritonavir 80 MG/ML Oral Solution [Norvir] Box of 5 | Branded Drug Box | Drug | RxNorm Extension |
| 36219270 | abacavir Oral Liquid Product | Clinical Dose Group | Drug | RxNorm |
| 40032839 | didanosine Chewable Tablet [Videx] | Branded Drug Form | Drug | RxNorm |
| 40874082 | Nevirapine 0.0417 MG/ML Oral Solution [Viramune] Box of 1 | Branded Drug Box | Drug | RxNorm Extension |
| 19047928 | stavudine 1 MG/ML [Zerit] | Branded Drug Comp | Drug | RxNorm |
| 21121234 | Delavirdine 100 MG Oral Tablet [Rescriptor] Box of 360 | Branded Drug Box | Drug | RxNorm Extension |
| 21085486 | darunavir 150 MG Oral Tablet [Prezista] Box of 240 by Janssen | Marketed Product | Drug | RxNorm Extension |
| 36223704 | stavudine Oral Product | Clinical Dose Group | Drug | RxNorm |
| 21026709 | fosamprenavir 115 MG Injection [Ivemend] | Branded Drug | Drug | RxNorm Extension |
| 36061898 | lopinavir 200 MG / ritonavir 50 MG Oral Tablet Box of 120 by Accord | Marketed Product | Drug | RxNorm Extension |
| 43298482 | {14 (dasabuvir 250 MG Oral Tablet) / 14 (ombitasvir 12.5 MG / paritaprevir 75 MG / Ritonavir 50 MG Oral Tablet) } Pack | Clinical Pack | Drug | RxNorm Extension |
| 41123761 | Nevirapine 400 MG Oral Tablet [Nevirapin Ratiopharm] Box of 30 | Branded Drug Box | Drug | RxNorm Extension |
| 19102469 | stavudine 75 MG Extended Release Oral Capsule | Clinical Drug | Drug | RxNorm |
| 35411824 | emtricitabine 200 MG / tenofovir alafenamide 25 MG Delayed Release Oral Tablet [Descovy] Box of 30 by Gilead | Marketed Product | Drug | RxNorm Extension |
| 21063034 | lopinavir 200 MG / Ritonavir 50 MG Oral Tablet Box of 120 | Clinical Drug Box | Drug | RxNorm Extension |
| 41041200 | Lamivudine 150 MG / Zidovudine 300 MG Oral Tablet [Combivir] Box of 60 by Docpharm | Marketed Product | Drug | RxNorm Extension |
| 36257626 | Nevirapine 400 MG Extended Release Oral Capsule [NEVIRAPINE MYLAN] Box of 30 | Branded Drug Box | Drug | RxNorm Extension |
| 782827 | emtricitabine 200 MG / Tenofovir 245 MG Oral Tablet [Emtricitabine / Tenofovir disoproxil Mylan] Box of 30 | Branded Drug Box | Drug | RxNorm Extension |
| 40746926 | efavirenz 600 MG / emtricitabine 200 MG / tenofovir disoproxil 250 MG Oral Tablet by TEVA | Marketed Product | Drug | RxNorm Extension |
| 42927761 | tenofovir disoproxil Oral Tablet [TENOLID] | Branded Drug Form | Drug | RxNorm Extension |
| 964018 | bictegravir / emtricitabine / tenofovir alafenamide Oral Tablet [Biktarvy] | Branded Drug Form | Drug | RxNorm |
| 41365231 | 10 ML Zidovudine 20 MG/ML Injection [Retrovir] Box of 10 | Quant Branded Box | Drug | RxNorm Extension |
| 40080338 | ritonavir Oral Solution [Norvir] | Branded Drug Form | Drug | RxNorm |
| 41292362 | Didanosine 250 MG Delayed Release Oral Capsule [Videx] Box of 60 by Orifarm Leverkus | Marketed Product | Drug | RxNorm Extension |
| 44127513 | Nevirapine 400 MG Extended Release Oral Tablet [Apo-Nevirapine] by Apotex | Marketed Product | Drug | RxNorm Extension |
| 43169046 | darunavir 600 MG Delayed Release Oral Tablet [Prezista] Box of 60 | Branded Drug Box | Drug | RxNorm Extension |
| 43026758 | DORAVIRINE 100 MG / Lamivudine 300 MG / tenofovir disoproxil 250 MG Delayed Release Oral Tablet [Delstrigo] Box of 30 by Merck | Marketed Product | Drug | RxNorm Extension |
| 36062290 | doravirine 100 MG / lamivudine 300 MG / tenofovir disoproxil 250 MG Oral Tablet [Delstrigo] by Merck | Marketed Product | Drug | RxNorm Extension |
| 35757504 | cobicistat 150 MG Delayed Release Oral Tablet [Tybost] Box of 30 by Gilead | Marketed Product | Drug | RxNorm Extension |
| 40095221 | zidovudine Oral Capsule | Clinical Drug Form | Drug | RxNorm |
| 21023721 | lopinavir 80 MG/ML / Ritonavir 20 MG/ML Oral Solution [Kaletra] by Abbvie | Marketed Product | Drug | RxNorm Extension |
| 21167670 | 200 ML Stavudine 1 MG/ML Oral Solution | Quant Clinical Drug | Drug | RxNorm Extension |
| 36249782 | dolutegravir 10 MG Oral Tablet | Clinical Drug | Drug | RxNorm |
| 36242993 | Sustiva Oral Product | Branded Dose Group | Drug | RxNorm |
| 41321319 | Nevirapine 200 MG Oral Tablet [Viramune] Box of 120 by Orifarm Leverkus | Marketed Product | Drug | RxNorm Extension |
| 41148899 | emtricitabine 200 MG / Rilpivirine 25 MG / tenofovir alafenamide 25 MG Oral Tablet Box of 90 | Clinical Drug Box | Drug | RxNorm Extension |
| 44086143 | Lamivudine 100 MG Oral Tablet [Heptovir] | Branded Drug | Drug | RxNorm Extension |
| 44178717 | Lamivudine 100 MG Delayed Release Oral Tablet [Zeffix] Box of 84 by Cc | Marketed Product | Drug | RxNorm Extension |
| 19102470 | stavudine 100 MG Extended Release Oral Capsule | Clinical Drug | Drug | RxNorm |
| 21032701 | Nelfinavir 250 MG Oral Tablet [Viracept] Box of 300 | Branded Drug Box | Drug | RxNorm Extension |
| 41159231 | efavirenz 600 MG Delayed Release Oral Tablet [Stocrin] | Branded Drug | Drug | RxNorm Extension |
| 19125943 | stavudine 50 MG Extended Release Oral Capsule [Zerit] | Branded Drug | Drug | RxNorm |
| 43037164 | tenofovir disoproxil 250 MG Delayed Release Oral Tablet Box of 30 by Sandoz | Marketed Product | Drug | RxNorm Extension |
| 41011683 | darunavir 600 MG Oral Tablet [Prezista] Box of 60 by Kohlpharma | Marketed Product | Drug | RxNorm Extension |
| 43189252 | Lamivudine 100 MG Delayed Release Oral Tablet Box of 28 | Clinical Drug Box | Drug | RxNorm Extension |
| 21069539 | 240 ML Amprenavir 15 MG/ML Oral Solution | Quant Clinical Drug | Drug | RxNorm Extension |
| 36503871 | darunavir 800 MG Oral Tablet by Betapharm | Marketed Product | Drug | RxNorm Extension |
| 21072950 | Nevirapine 200 MG Oral Tablet [Viramune] Box of 60 by Dowelhurst | Marketed Product | Drug | RxNorm Extension |
| 19120186 | lamivudine 150 MG / zidovudine 300 MG [Combivir] | Branded Drug Comp | Drug | RxNorm |
| 36880598 | abacavir 351 MG | Clinical Drug Comp | Drug | RxNorm Extension |
| 43162536 | Nevirapine 400 MG Oral Tablet Box of 30 | Clinical Drug Box | Drug | RxNorm Extension |
| 40898935 | Didanosine 125 MG Extended Release Oral Capsule Box of 60 | Clinical Drug Box | Drug | RxNorm Extension |
| 41312203 | Lamivudine 100 MG Delayed Release Oral Tablet [Lamivudin Teva] Box of 28 | Branded Drug Box | Drug | RxNorm Extension |
| 40978751 | Lamivudine 150 MG / Zidovudine 300 MG Delayed Release Oral Tablet [Combivir] Box of 60 by Bb Farma | Marketed Product | Drug | RxNorm Extension |
| 40166596 | efavirenz 600 MG / lamivudine 300 MG / tenofovir disoproxil fumarate 300 MG Oral Tablet | Clinical Drug | Drug | RxNorm |
| 43146909 | Rilpivirine 25 MG Delayed Release Oral Tablet [Edurant] Box of 30 | Branded Drug Box | Drug | RxNorm Extension |
| 43140379 | Nevirapine 200 MG Oral Tablet [NEVIRAPINE MYLAN] Box of 60 by Mylan | Marketed Product | Drug | RxNorm Extension |
| 41211615 | Didanosine 250 MG Delayed Release Oral Capsule Box of 60 | Clinical Drug Box | Drug | RxNorm Extension |
| 40950315 | Atazanavir 300 MG Oral Capsule [Reyataz] Box of 30 by Veron | Marketed Product | Drug | RxNorm Extension |
| 21053213 | lopinavir 133 MG / Ritonavir 33.3 MG Oral Capsule Box of 180 | Clinical Drug Box | Drug | RxNorm Extension |
| 41411931 | 1.1 ML enfuvirtide 90 MG/ML Injection [Fuzeon] Box of 60 | Quant Branded Box | Drug | RxNorm Extension |
| 36074449 | abacavir 300 MG Oral Tablet Box of 60 by Mylan | Marketed Product | Drug | RxNorm Extension |
| 41117641 | Didanosine 400 MG Extended Release Oral Capsule Box of 60 | Clinical Drug Box | Drug | RxNorm Extension |
| 1704183 | lamivudine | Ingredient | Drug | RxNorm |
| 1704217 | lamivudine 300 MG Oral Tablet | Clinical Drug | Drug | RxNorm |
| 41250074 | Lamivudine 100 MG Oral Tablet [LAMIVUDINE MYLAN] Box of 84 | Branded Drug Box | Drug | RxNorm Extension |
| 19015459 | nelfinavir 250 MG [Viracept] | Branded Drug Comp | Drug | RxNorm |
| 43191063 | cobicistat 150 MG / elvitegravir 150 MG / emtricitabine 200 MG / tenofovir disoproxil 250 MG Delayed Release Oral Tablet | Clinical Drug | Drug | RxNorm Extension |
| 41469748 | 4000 MG Didanosine 1 MG/MG Oral Suspension | Quant Clinical Drug | Drug | RxNorm Extension |
| 41197042 | Nevirapine 200 MG Oral Tablet [Viramune] Box of 120 by Haematogmbh | Marketed Product | Drug | RxNorm Extension |
| 40864786 | Zidovudine 250 MG Oral Capsule Box of 80 | Clinical Drug Box | Drug | RxNorm Extension |
| 1756831 | darunavir | Ingredient | Drug | RxNorm |
| 44096044 | dasabuvir / ombitasvir / paritaprevir / Ritonavir Oral Tablet [Holkira] | Branded Drug Form | Drug | RxNorm Extension |
| 44120244 | abacavir 600 MG / Lamivudine 300 MG [Pms-Abacavir-Lamivudine] | Branded Drug Comp | Drug | RxNorm Extension |
| 40844389 | Lamivudine 300 MG Oral Tablet [Lamivudin Teva] Box of 80 | Branded Drug Box | Drug | RxNorm Extension |
| 42874225 | cobicistat 150 MG / elvitegravir 150 MG / emtricitabine 200 MG / tenofovir disoproxil fumarate 300 MG Oral Tablet [Stribild] | Branded Drug | Drug | RxNorm |
| 42967486 | Zidovudine Oral Capsule [AZIDOMINE] | Branded Drug Form | Drug | RxNorm Extension |
| 36404666 | fosamprenavir 115 MG Injectable Solution [Ivemend] by Merck | Marketed Product | Drug | RxNorm Extension |
| 19078497 | indinavir 333 MG Oral Capsule | Clinical Drug | Drug | RxNorm |
| 44188518 | Lamivudine 150 MG Delayed Release Oral Tablet [Lamivudin Hexal] | Branded Drug | Drug | RxNorm Extension |
| 21102496 | efavirenz 200 MG Oral Capsule [Sustiva] by Bristol Myers Squibb | Marketed Product | Drug | RxNorm Extension |
| 42705416 | raltegravir Chewable Tablet [Isentress] | Branded Drug Form | Drug | RxNorm |
| 21157930 | 240 ML Amprenavir 15 MG/ML Oral Solution [Agenerase] by Glaxosmithkline | Marketed Product | Drug | RxNorm Extension |
| 43032456 | abacavir 600 MG / Lamivudine 300 MG Oral Tablet Box of 30 by Sandoz | Marketed Product | Drug | RxNorm Extension |
| 1738165 | efavirenz 600 MG Oral Tablet | Clinical Drug | Drug | RxNorm |
| 40708525 | 1000 MG tenofovir disoproxil 0.033 MG/MG Oral Granules [Viread] by Gilead | Marketed Product | Drug | RxNorm Extension |
| 43296720 | 1 ML Didanosine 5 MG/ML Powder for Oral Solution | Quant Clinical Drug | Drug | RxNorm Extension |
| 41435605 | 225 ML fosamprenavir 50 MG/ML Oral Solution [Telzir] Box of 1 | Quant Branded Box | Drug | RxNorm Extension |
| 1710659 | zidovudine 10 MG/ML Oral Solution [Retrovir] | Branded Drug | Drug | RxNorm |
| 44205179 | 4030 MG Didanosine 1 MG/MG Oral Solution [Videx] Box of 1 | Quant Branded Box | Drug | RxNorm Extension |
| 43285658 | maraviroc 300 MG Oral Tablet Box of 90 | Clinical Drug Box | Drug | RxNorm Extension |
| 40162290 | delavirdine mesylate 200 MG | Clinical Drug Comp | Drug | RxNorm |
| 41107092 | Zidovudine 0.25 MG/ML | Clinical Drug Comp | Drug | RxNorm Extension |
| 40720649 | tenofovir disoproxil 150 MG Oral Tablet [Viread] by Gilead | Marketed Product | Drug | RxNorm Extension |
| 43037165 | tenofovir disoproxil 250 MG Delayed Release Oral Tablet Box of 30 by Arrow | Marketed Product | Drug | RxNorm Extension |
| 45892744 | atazanavir 50 MG Oral Powder [Reyataz] | Branded Drug | Drug | RxNorm |
| 21092417 | Lamivudine 150 MG / Zidovudine 300 MG Oral Tablet [Combivir] Box of 60 by Mawdsley-Brooks | Marketed Product | Drug | RxNorm Extension |
| 41106649 | Atazanavir 200 MG Oral Capsule [Reyataz] Box of 60 by Kohlpharma | Marketed Product | Drug | RxNorm Extension |
| 995224 | tenofovir disoproxil 250 MG Oral Tablet [Tenofovir Cipla] | Branded Drug | Drug | RxNorm Extension |
| 41412003 | 1.1 ML enfuvirtide 90 MG/ML Injectable Solution Box of 60 | Quant Clinical Box | Drug | RxNorm Extension |
| 994687 | emtricitabine / tenofovir disoproxil Oral Tablet [Emtenovo] | Branded Drug Form | Drug | RxNorm Extension |
| 43294113 | Indinavir 400 MG Oral Capsule Box of 84 | Clinical Drug Box | Drug | RxNorm Extension |
| 43026152 | efavirenz 600 MG / emtricitabine 200 MG / tenofovir disoproxil 250 MG Delayed Release Oral Tablet Box of 30 by Sandoz | Marketed Product | Drug | RxNorm Extension |
| 1510226 | Symfi Pill | Branded Dose Group | Drug | RxNorm |
| 42927736 | tenofovir disoproxil 250 MG [PREAD] | Branded Drug Comp | Drug | RxNorm Extension |
| 40947776 | Lamivudine 100 MG Oral Tablet [Zeffix] Box of 28 by Mtk | Marketed Product | Drug | RxNorm Extension |
| 36272454 | etravirine 200 MG Disintegrating Oral Tablet | Clinical Drug | Drug | RxNorm Extension |
| 41261212 | Didanosine 100 MG Chewable Tablet [Videx] Box of 60 by Mtk | Marketed Product | Drug | RxNorm Extension |
| 41434399 | 200 ML Zidovudine 0.25 MG/ML Oral Solution [Retrovir] Box of 1 by ViiV | Marketed Product | Drug | RxNorm Extension |
| 43678859 | efavirenz 600 MG Oral Tablet [Efavirenz Aurobindo] | Branded Drug | Drug | RxNorm Extension |
| 41280882 | Lamivudine 150 MG / Zidovudine 300 MG Oral Tablet [Combivir Cmj] Box of 60 | Branded Drug Box | Drug | RxNorm Extension |
| 19113609 | indinavir 100 MG Oral Capsule [Crixivan] | Branded Drug | Drug | RxNorm |
| 36275044 | emtricitabine 200 MG / tenofovir disoproxil 250 MG Oral Tablet Box of 30 | Clinical Drug Box | Drug | RxNorm Extension |
| 36890656 | Saquinavir 572 MG Oral Tablet | Clinical Drug | Drug | RxNorm Extension |
| 36219268 | abacavir / lamivudine Oral Product | Clinical Dose Group | Drug | RxNorm |
| 37592142 | Nevirapine 400 MG Extended Release Oral Tablet [Viramune] by Aca Mueller | Marketed Product | Drug | RxNorm Extension |
| 42543875 | lamivudine / tenofovir disoproxil Oral Tablet [Cimduo] | Branded Drug Form | Drug | RxNorm |
| 36784672 | abacavir / Lamivudine Delayed Release Oral Tablet [Abacavir / Lamivudin Sandoz] | Branded Drug Form | Drug | RxNorm Extension |
| 21075859 | maraviroc 150 MG Oral Tablet [Celsentri] | Branded Drug | Drug | RxNorm Extension |
| 43178347 | Lamivudine 150 MG / Zidovudine 300 MG [LAMIVUDINE/ZIDOVUDINE SANDOZ] | Branded Drug Comp | Drug | RxNorm Extension |
| 41299919 | Lamivudine Delayed Release Oral Tablet [Lamivir] | Branded Drug Form | Drug | RxNorm Extension |
| 41041201 | Lamivudine 300 MG Oral Tablet [Lamivudin Hexal] Box of 30 by Novartis | Marketed Product | Drug | RxNorm Extension |
| 44040584 | Zidovudine 100 MG [Apo-Zidovudine] | Branded Drug Comp | Drug | RxNorm Extension |
| 21030087 | 180 ML efavirenz 30 MG/ML Oral Solution | Quant Clinical Drug | Drug | RxNorm Extension |
| 40712269 | 120 ML lopinavir 80 MG/ML / Ritonavir 20 MG/ML Oral Solution [Kaletra] by Abbvie | Marketed Product | Drug | RxNorm Extension |
| 19048073 | lopinavir 100 MG / ritonavir 25 MG [Kaletra] | Branded Drug Comp | Drug | RxNorm |
| 43283254 | Lamivudine 100 MG Oral Tablet Box of 84 | Clinical Drug Box | Drug | RxNorm Extension |
| 21082603 | Lamivudine 5 MG/ML [Zeffix] | Branded Drug Comp | Drug | RxNorm Extension |
| 41195630 | Saquinavir 500 MG Oral Tablet [Invirase] Box of 120 by Beragena | Marketed Product | Drug | RxNorm Extension |
| 40927355 | tenofovir disoproxil 123 MG Oral Tablet Box of 30 | Clinical Drug Box | Drug | RxNorm Extension |
| 40241981 | emtricitabine / rilpivirine / tenofovir disoproxil Oral Tablet [Complera] | Branded Drug Form | Drug | RxNorm |
| 35861975 | indinavir Oral Tablet [Youxin] | Branded Drug Form | Drug | RxNorm Extension |
| 41232113 | abacavir 703 MG / Lamivudine 300 MG Oral Tablet [Kivexa] Box of 30 by ViiV | Marketed Product | Drug | RxNorm Extension |
| 21154397 | raltegravir 100 MG Oral Solution [Isentress] by Merck | Marketed Product | Drug | RxNorm Extension |
| 36257199 | emtricitabine 200 MG / tenofovir disoproxil 250 MG Oral Tablet | Clinical Drug | Drug | RxNorm Extension |
| 41323443 | efavirenz 600 MG / emtricitabine 200 MG / tenofovir disoproxil 300 MG Oral Tablet [Atripla] Box of 90 by Cc | Marketed Product | Drug | RxNorm Extension |
| 42705554 | darunavir Oral Suspension [Prezista] | Branded Drug Form | Drug | RxNorm |
| 21159260 | cobicistat 150 MG / darunavir 800 MG [Rezolsta] | Branded Drug Comp | Drug | RxNorm Extension |
| 41009206 | Nevirapine 200 MG Oral Tablet [Nevirapin Aurobindo] Box of 120 by Aurobindo | Marketed Product | Drug | RxNorm Extension |
| 40977854 | Nevirapine 200 MG Oral Tablet [Viramune] Box of 60 by Orifarm Leverkus | Marketed Product | Drug | RxNorm Extension |
| 36261828 | tenofovir disoproxil Delayed Release Oral Tablet [Tenofovir Disoproxil Mylan] | Branded Drug Form | Drug | RxNorm Extension |
| 21065836 | darunavir 300 MG Oral Tablet Box of 120 | Clinical Drug Box | Drug | RxNorm Extension |
| 2052738 | tenofovir disoproxil 300 MG Oral Tablet [HEPALID] by Guju | Marketed Product | Drug | RxNorm Extension |
| 44028387 | Lamivudine 300 MG [Apo-Lamivudine] | Branded Drug Comp | Drug | RxNorm Extension |
| 43026168 | emtricitabine 200 MG / tenofovir disoproxil 250 MG Oral Tablet Box of 30 by Eurogenerics | Marketed Product | Drug | RxNorm Extension |
| 40051188 | lamivudine Oral Tablet [Epivir HBV] | Branded Drug Form | Drug | RxNorm |
| 43696024 | Didanosine 1 MG/MG Oral Powder | Clinical Drug | Drug | RxNorm Extension |
| 40160002 | lopinavir / ritonavir Oral Solution [Kaletra] | Branded Drug Form | Drug | RxNorm |
| 41088350 | abacavir 703 MG / Lamivudine 300 MG Oral Tablet Box of 30 | Clinical Drug Box | Drug | RxNorm Extension |
| 36062293 | lamivudine 300 MG / tenofovir disoproxil 250 MG Oral Tablet Box of 90 | Clinical Drug Box | Drug | RxNorm Extension |
| 40932068 | abacavir 703 MG / dolutegravir 50 MG / Lamivudine 300 MG Oral Tablet Box of 90 | Clinical Drug Box | Drug | RxNorm Extension |
| 41256602 | Zidovudine 250 MG Oral Capsule [Retrovir] Box of 40 by Gerke | Marketed Product | Drug | RxNorm Extension |
| 2052763 | tenofovir disoproxil 300 MG [TENOFODE] | Branded Drug Comp | Drug | RxNorm Extension |
| 35412534 | efavirenz 600 MG Delayed Release Oral Tablet [EFAVIRENZ CRISTERS] Box of 30 by Cristers | Marketed Product | Drug | RxNorm Extension |
| 43169042 | Saquinavir 500 MG Delayed Release Oral Tablet [Invirase] | Branded Drug | Drug | RxNorm Extension |
| 43167287 | Lamivudine 150 MG / Zidovudine 300 MG [LAMIVUDINE/ZIDOVUDINE MYLAN] | Branded Drug Comp | Drug | RxNorm Extension |
| 1748989 | lopinavir 133 MG / ritonavir 33.3 MG Oral Capsule [Kaletra] | Branded Drug | Drug | RxNorm |
| 40956688 | Lamivudine Oral Tablet [Lamivudin Teva] | Branded Drug Form | Drug | RxNorm Extension |
| 21164210 | fosamprenavir 150 MG Injection Box of 1 | Clinical Drug Box | Drug | RxNorm Extension |
| 42941332 | Lamivudine 100 MG [ZERAFFIC] | Branded Drug Comp | Drug | RxNorm Extension |
| 43178343 | Lamivudine 150 MG Delayed Release Oral Tablet | Clinical Drug | Drug | RxNorm Extension |
| 35774329 | cobicistat 150 MG Delayed Release Oral Tablet [Tybost] by Gilead | Marketed Product | Drug | RxNorm Extension |
| 36062284 | abacavir 600 MG / lamivudine 300 MG Oral Tablet Box of 30 by Sun | Marketed Product | Drug | RxNorm Extension |
| 41190788 | Didanosine 200 MG Oral Tablet [Videx] | Branded Drug | Drug | RxNorm Extension |
| 41235209 | efavirenz 600 MG [Efavirenz Teva] | Branded Drug Comp | Drug | RxNorm Extension |
| 42927724 | tenofovir disoproxil 250 MG [VIRREAL] | Branded Drug Comp | Drug | RxNorm Extension |
| 2052737 | tenofovir disoproxil 300 MG Oral Tablet [HEPALID] | Branded Drug | Drug | RxNorm Extension |
| 43804510 | Nevirapine 200 MG Oral Tablet [Nevirapin Hexal] | Branded Drug | Drug | RxNorm Extension |
| 21023938 | Amprenavir 150 MG Oral Capsule [Agenerase] by Glaxosmithkline | Marketed Product | Drug | RxNorm Extension |
| 43202117 | tenofovir disoproxil 0.033 MG/MG | Clinical Drug Comp | Drug | RxNorm Extension |
| 41026831 | tenofovir disoproxil 150 MG Oral Tablet [Viread] Box of 30 | Branded Drug Box | Drug | RxNorm Extension |
| 21134575 | fosamprenavir 115 MG Injection | Clinical Drug | Drug | RxNorm Extension |
| 35861966 | indinavir 200 MG Oral Tablet | Clinical Drug | Drug | RxNorm Extension |
| 21051386 | ombitasvir 12.5 MG / paritaprevir 75 MG / Ritonavir 50 MG Oral Tablet [Viekirax] by Abbvie | Marketed Product | Drug | RxNorm Extension |
| 43166158 | Didanosine 125 MG Extended Release Oral Tablet [Videx] Box of 30 | Branded Drug Box | Drug | RxNorm Extension |
| 1736999 | tipranavir | Ingredient | Drug | RxNorm |
| 1718486 | maraviroc Oral Solution | Clinical Drug Form | Drug | RxNorm |
| 21053214 | lopinavir 133 MG / Ritonavir 33.3 MG Oral Capsule [Kaletra] by Abbott | Marketed Product | Drug | RxNorm Extension |
| 41235195 | enfuvirtide 90 MG/ML [Fuzeon] | Branded Drug Comp | Drug | RxNorm Extension |
| 21164209 | fosamprenavir Injection [Ivemend] | Branded Drug Form | Drug | RxNorm Extension |
| 19102211 | fosamprenavir 700 MG Oral Tablet | Clinical Drug | Drug | RxNorm |
| 36061613 | darunavir 400 MG Delayed Release Oral Tablet by Dr Reddy's | Marketed Product | Drug | RxNorm Extension |
| 36419263 | raltegravir 600 MG Oral Tablet [Isentress] Box of 180 | Branded Drug Box | Drug | RxNorm Extension |
| 35743804 | Lamivudine 150 MG / Zidovudine 300 MG Delayed Release Oral Tablet Box of 60 by Sandoz | Marketed Product | Drug | RxNorm Extension |
| 35770801 | Didanosine 25 MG Oral Tablet Box of 60 | Clinical Drug Box | Drug | RxNorm Extension |
| 43178349 | Lamivudine 150 MG / Zidovudine 300 MG Delayed Release Oral Tablet [LAMIVUDINE/ZIDOVUDINE SANDOZ] | Branded Drug | Drug | RxNorm Extension |
| 44167259 | Nevirapine 400 MG Extended Release Oral Tablet [Viramune] Box of 90 by Haematogmbh | Marketed Product | Drug | RxNorm Extension |
| 36062299 | lamivudine 300 MG Delayed Release Oral Tablet Box of 30 by Accord | Marketed Product | Drug | RxNorm Extension |
| 41040936 | lopinavir 133 MG / Ritonavir 33.3 MG Oral Capsule [Kaletra] Box of 180 by Orifarm Leverkus | Marketed Product | Drug | RxNorm Extension |
| 36062297 | lamivudine 300 MG / tenofovir disoproxil 250 MG Oral Tablet | Clinical Drug | Drug | RxNorm Extension |
| 35200457 | doravirine 100 MG Oral Tablet [Pifeltro] | Branded Drug | Drug | RxNorm |
| 35415364 | 226 ML fosamprenavir 50 MG/ML Oral Suspension Box of 1 | Quant Clinical Box | Drug | RxNorm Extension |
| 41136963 | darunavir 400 MG Oral Tablet [Prezista] Box of 60 by Haematogmbh | Marketed Product | Drug | RxNorm Extension |
| 35414451 | 90 ML Ritonavir 80 MG/ML Oral Solution [Norvir] Box of 1 by Abbvie | Marketed Product | Drug | RxNorm Extension |
| 40706773 | 1010 MG tenofovir disoproxil 0.033 MG/MG Oral Granules [Viread] by Gilead | Marketed Product | Drug | RxNorm Extension |
| 41302143 | Ritonavir 100 MG Delayed Release Oral Tablet Box of 90 | Clinical Drug Box | Drug | RxNorm Extension |
| 1711524 | indinavir 200 MG Oral Capsule [Crixivan] | Branded Drug | Drug | RxNorm |
| 35756375 | Stavudine 37.5 MG Extended Release Oral Capsule [Zerit] by Bristol Myers Squibb | Marketed Product | Drug | RxNorm Extension |
| 43272621 | lopinavir 133 MG / Ritonavir 33.3 MG Oral Capsule Box of 90 | Clinical Drug Box | Drug | RxNorm Extension |
| 40976102 | tenofovir disoproxil 250 MG Oral Tablet [Viread] Box of 90 by Gerke | Marketed Product | Drug | RxNorm Extension |
| 40979945 | efavirenz 600 MG Delayed Release Oral Tablet [Sustiva] Box of 30 by Gerke | Marketed Product | Drug | RxNorm Extension |
| 40142130 | lamivudine / stavudine Oral Tablet | Clinical Drug Form | Drug | RxNorm |
| 43588591 | efavirenz 600 MG Oral Tablet [EFAVIRENZ SANDOZ] by Hexal | Marketed Product | Drug | RxNorm Extension |
| 1736923 | fosamprenavir 50 MG/ML | Clinical Drug Comp | Drug | RxNorm |
| 41228645 | maraviroc 300 MG Oral Tablet [Celsentri] Box of 60 by Orifarm Leverkus | Marketed Product | Drug | RxNorm Extension |
| 43189261 | abacavir 600 MG / dolutegravir 50 MG / Lamivudine 300 MG Delayed Release Oral Tablet | Clinical Drug | Drug | RxNorm Extension |
| 40749146 | Zidovudine 10 MG/ML Injectable Solution [Retrovir] | Branded Drug | Drug | RxNorm Extension |
| 1756840 | darunavir 400 MG Oral Tablet [Prezista] | Branded Drug | Drug | RxNorm |
| 41430636 | 170 ML emtricitabine 0.0588 MG/ML Oral Solution | Quant Clinical Drug | Drug | RxNorm Extension |
| 41221905 | Didanosine 400 MG Extended Release Oral Capsule [Videx] | Branded Drug | Drug | RxNorm Extension |
| 43022445 | darunavir 600 MG Oral Tablet Box of 60 by Mylan | Marketed Product | Drug | RxNorm Extension |
| 44107532 | Didanosine Oral Tablet | Clinical Drug Form | Drug | RxNorm Extension |
| 2011766 | nelfinavir 50 MG Oral Powder [Viracept] by Dong-a | Marketed Product | Drug | RxNorm Extension |
| 36272121 | Nevirapine Extended Release Oral Capsule [Viramune] | Branded Drug Form | Drug | RxNorm Extension |
| 35858556 | stavudine 30 MG Oral Tablet | Clinical Drug | Drug | RxNorm Extension |
| 41435606 | 225 ML fosamprenavir 50 MG/ML Oral Solution [Telzir] | Quant Branded Drug | Drug | RxNorm Extension |
| 41117647 | Didanosine 200 MG Oral Tablet | Clinical Drug | Drug | RxNorm Extension |
| 44127860 | Lamivudine 300 MG Oral Tablet [Apo-Lamivudine] by Apotex | Marketed Product | Drug | RxNorm Extension |
| 43288724 | Lamivudine 100 MG [Zetlam] | Branded Drug Comp | Drug | RxNorm Extension |
| 40909013 | emtricitabine 200 MG / Rilpivirine 25 MG / tenofovir disoproxil 300 MG Oral Tablet [Eviplera] | Branded Drug | Drug | RxNorm Extension |
| 43186772 | 60 ML lopinavir 80 MG/ML / Ritonavir 20 MG/ML Oral Solution [Kaletra] | Quant Branded Drug | Drug | RxNorm Extension |
| 35750352 | Stavudine 37.5 MG Extended Release Oral Capsule [Zerit] Box of 56 | Branded Drug Box | Drug | RxNorm Extension |
| 35860458 | ritonavir Oral Solution [Maikexin] | Branded Drug Form | Drug | RxNorm Extension |
| 37594090 | 1500 MG Atazanavir 0.0333 MG/MG Oral Granules | Quant Clinical Drug | Drug | RxNorm Extension |
| 40730843 | Lamivudine 300 MG Oral Tablet by Accord | Marketed Product | Drug | RxNorm Extension |
| 43659804 | Lamivudine / Zidovudine Oral Tablet [Lamizido] | Branded Drug Form | Drug | RxNorm Extension |
| 40916369 | Lamivudine 300 MG Oral Tablet [Lamivudin Hexal] Box of 60 by Novartis | Marketed Product | Drug | RxNorm Extension |
| 2053071 | lamivudine Oral Tablet [LAMIDINE] | Branded Drug Form | Drug | RxNorm Extension |
| 40835037 | Nevirapine 50 MG Oral Tablet Box of 180 | Clinical Drug Box | Drug | RxNorm Extension |
| 21034051 | Zidovudine 250 MG Oral Capsule Box of 60 | Clinical Drug Box | Drug | RxNorm Extension |
| 40979421 | fosamprenavir 700 MG Oral Tablet [Telzir] Box of 60 by Aca Mueller | Marketed Product | Drug | RxNorm Extension |
| 21161276 | lopinavir 100 MG / Ritonavir 25 MG Oral Tablet Box of 60 | Clinical Drug Box | Drug | RxNorm Extension |
| 43195890 | maraviroc 150 MG Delayed Release Oral Tablet [Celsentri] | Branded Drug | Drug | RxNorm Extension |
| 41222274 | darunavir 600 MG Delayed Release Oral Tablet [Prezista] Box of 90 | Branded Drug Box | Drug | RxNorm Extension |
| 36062291 | doravirine 100 MG / lamivudine 300 MG / tenofovir disoproxil 250 MG Delayed Release Oral Tablet [Delstrigo] by Merck | Marketed Product | Drug | RxNorm Extension |
| 42705918 | tenofovir disoproxil fumarate 200 MG [Viread] | Branded Drug Comp | Drug | RxNorm |
| 41324936 | Atazanavir 300 MG Oral Capsule [Reyataz] Box of 90 by Orifarm Leverkus | Marketed Product | Drug | RxNorm Extension |
| 44185709 | raltegravir 400 MG Oral Tablet [Isentress] Box of 60 by Cambridge Major | Marketed Product | Drug | RxNorm Extension |
| 41101257 | tenofovir disoproxil 250 MG Oral Tablet [Viread] Box of 90 by Orifarm Leverkus | Marketed Product | Drug | RxNorm Extension |
| 35771278 | Didanosine 200 MG Extended Release Oral Capsule [Videx EC] Box of 30 | Branded Drug Box | Drug | RxNorm Extension |
| 35414412 | 60 ML lopinavir 80 MG/ML / Ritonavir 20 MG/ML Oral Solution [Kaletra] Box of 5 by Abbvie | Marketed Product | Drug | RxNorm Extension |
| 42927749 | tenofovir disoproxil Oral Tablet [LINOPHED] | Branded Drug Form | Drug | RxNorm Extension |
| 41404323 | 90 ML Ritonavir 0.889 MG/ML Oral Solution [Norvir] Box of 1 | Quant Branded Box | Drug | RxNorm Extension |
| 793027 | dolutegravir / rilpivirine Oral Tablet | Clinical Drug Form | Drug | RxNorm |
| 36062298 | lamivudine 300 MG Delayed Release Oral Tablet Box of 30 by Milpharm | Marketed Product | Drug | RxNorm Extension |
| 41006884 | Zidovudine 250 MG Oral Capsule [Retrovir] Box of 40 by Beragena | Marketed Product | Drug | RxNorm Extension |
| 43858805 | efavirenz 600 MG Oral Tablet [EFAVIRENZ SANDOZ] | Branded Drug | Drug | RxNorm Extension |
| 43587495 | Zidovudine 250 MG [Zidovudin Aurobindo] | Branded Drug Comp | Drug | RxNorm Extension |
| 21033495 | Lamivudine 150 MG Oral Tablet [Epivir] by ViiV | Marketed Product | Drug | RxNorm Extension |
| 36785883 | efavirenz 600 MG Oral Tablet [Stocrin] Box of 30 by PI-Pharma | Marketed Product | Drug | RxNorm Extension |
| 43026157 | efavirenz 600 MG / emtricitabine 200 MG / tenofovir disoproxil 250 MG Oral Tablet Box of 30 by Sandoz | Marketed Product | Drug | RxNorm Extension |
| 21108714 | 200 ML darunavir 100 MG/ML Oral Suspension | Quant Clinical Drug | Drug | RxNorm Extension |
| 19088562 | ritonavir 20 MG/ML | Clinical Drug Comp | Drug | RxNorm |
| 40733278 | maraviroc 25 MG Oral Tablet [Celsentri] Box of 120 | Branded Drug Box | Drug | RxNorm Extension |
| 35773217 | Lamivudine 100 MG Delayed Release Oral Tablet by TEVA | Marketed Product | Drug | RxNorm Extension |
| 40720766 | raltegravir 100 MG Oral Granules [Isentress] | Branded Drug | Drug | RxNorm Extension |
| 43155129 | Didanosine Extended Release Oral Tablet | Clinical Drug Form | Drug | RxNorm Extension |
| 43206300 | Nevirapine Oral Tablet [NEVIRAPINE CRISTERS] | Branded Drug Form | Drug | RxNorm Extension |
| 1758539 | etravirine 100 MG Oral Tablet [Intelence] | Branded Drug | Drug | RxNorm |
| 36226566 | efavirenz Oral Product | Clinical Dose Group | Drug | RxNorm |
| 36214321 | indinavir Pill | Clinical Dose Group | Drug | RxNorm |
| 41228646 | maraviroc 300 MG Oral Tablet [Celsentri] Box of 60 by Gerke | Marketed Product | Drug | RxNorm Extension |
| 43821524 | abacavir 300 MG / Lamivudine 150 MG / Zidovudine 300 MG [Triplead-Ratiopharm] | Branded Drug Comp | Drug | RxNorm Extension |
| 1746244 | saquinavir | Ingredient | Drug | RxNorm |
| 43277959 | Lamivudine 100 MG Oral Tablet [Zetlam] | Branded Drug | Drug | RxNorm Extension |
| 44087733 | tenofovir disoproxil 300 MG Oral Tablet [Viread] by Gilead | Marketed Product | Drug | RxNorm Extension |
| 43178344 | Lamivudine 150 MG Delayed Release Oral Tablet [LAMIVUDINE MYLAN] Box of 60 | Branded Drug Box | Drug | RxNorm Extension |
| 41288585 | Saquinavir 500 MG Oral Tablet [Invirase] Box of 120 by Gerke | Marketed Product | Drug | RxNorm Extension |
| 41148915 | efavirenz 600 MG / emtricitabine 200 MG / tenofovir disoproxil 300 MG Oral Tablet Box of 90 | Clinical Drug Box | Drug | RxNorm Extension |
| 41201042 | abacavir 351 MG / Lamivudine 150 MG / Zidovudine 300 MG Oral Tablet [Trizivir] Box of 60 by Cc | Marketed Product | Drug | RxNorm Extension |
| 36220596 | fosamprenavir Oral Product | Clinical Dose Group | Drug | RxNorm |
| 43200288 | abacavir 600 MG / Lamivudine 300 MG Delayed Release Oral Tablet [Kivexa] | Branded Drug | Drug | RxNorm Extension |
| 44114794 | Indinavir 400 MG Oral Capsule [Crixivan] by Merck | Marketed Product | Drug | RxNorm Extension |
| 41250072 | Lamivudine 100 MG Delayed Release Oral Tablet [Zeffix] Box of 84 | Branded Drug Box | Drug | RxNorm Extension |
| 44168094 | abacavir 703 MG / Lamivudine 300 MG Oral Tablet Box of 30 by Novartis | Marketed Product | Drug | RxNorm Extension |
| 36236511 | Atripla Pill | Branded Dose Group | Drug | RxNorm |
| 2053060 | lamivudine 100 MG Oral Tablet [ZAVUDIN] by Alvogen | Marketed Product | Drug | RxNorm Extension |
| 44127082 | raltegravir 400 MG Oral Tablet [Isentress] by Merck | Marketed Product | Drug | RxNorm Extension |
| 36242596 | Viramune Pill | Branded Dose Group | Drug | RxNorm |
| 35858575 | stavudine Oral Capsule [Sairuite] | Branded Drug Form | Drug | RxNorm Extension |
| 35861965 | indinavir 400 MG [Jiaxihuan] | Branded Drug Comp | Drug | RxNorm Extension |
| 36220376 | Complera Oral Product | Branded Dose Group | Drug | RxNorm |
| 41117644 | Didanosine 200 MG Extended Release Oral Capsule Box of 60 | Clinical Drug Box | Drug | RxNorm Extension |
| 36277450 | Ritonavir 100 MG Oral Suspension | Clinical Drug | Drug | RxNorm Extension |
| 42874220 | cobicistat | Ingredient | Drug | RxNorm |
| 40844391 | Lamivudine 150 MG Delayed Release Oral Tablet [Lamivudin Teva] | Branded Drug | Drug | RxNorm Extension |
| 41433119 | 200 ML darunavir 0.5 MG/ML Oral Solution [Prezista] Box of 1 by Janssen | Marketed Product | Drug | RxNorm Extension |
| 35862029 | zidovudine Oral Solution [Lituowei] | Branded Drug Form | Drug | RxNorm Extension |
| 41195247 | tenofovir disoproxil 250 MG Oral Tablet [Viread] Box of 90 by Emra-Med | Marketed Product | Drug | RxNorm Extension |
| 43022448 | darunavir 600 MG Oral Tablet Box of 60 by TEVA | Marketed Product | Drug | RxNorm Extension |
| 43160866 | lopinavir / Ritonavir Delayed Release Oral Tablet [Kaletra] | Branded Drug Form | Drug | RxNorm Extension |
| 40897794 | Lamivudine 150 MG Delayed Release Oral Tablet Box of 80 | Clinical Drug Box | Drug | RxNorm Extension |
| 41312204 | Lamivudine 100 MG Delayed Release Oral Tablet [Lamivudin Teva] | Branded Drug | Drug | RxNorm Extension |
| 40733289 | maraviroc 75 MG Oral Tablet [Celsentri] | Branded Drug | Drug | RxNorm Extension |
| 40885492 | Lamivudine 150 MG / Zidovudine 300 MG Oral Tablet [Combivir] Box of 60 by Orifarm Leverkus | Marketed Product | Drug | RxNorm Extension |
| 43605578 | abacavir 351 MG / Lamivudine 150 MG / Zidovudine 300 MG Oral Tablet by Mylan | Marketed Product | Drug | RxNorm Extension |
| 43133974 | efavirenz Delayed Release Oral Tablet [EFAVIRENZ SANDOZ] | Branded Drug Form | Drug | RxNorm Extension |
| 44037965 | cobicistat 150 MG / darunavir 800 MG Oral Tablet [Prezcobix] by Janssen | Marketed Product | Drug | RxNorm Extension |
| 41430634 | 170 ML emtricitabine 0.0588 MG/ML Oral Solution [Emtriva] Box of 1 by Gilead | Marketed Product | Drug | RxNorm Extension |
| 793028 | dolutegravir 50 MG / rilpivirine 25 MG Oral Tablet | Clinical Drug | Drug | RxNorm |
| 35858561 | stavudine 20 MG [Shazhi] | Branded Drug Comp | Drug | RxNorm Extension |
| 40008594 | amprenavir Oral Solution | Clinical Drug Form | Drug | RxNorm |
| 40898933 | Didanosine 1 MG/MG Oral Suspension Box of 1 | Clinical Drug Box | Drug | RxNorm Extension |
| 43158447 | abacavir 20 MG/ML Oral Solution [Ziagen] Box of 1 | Branded Drug Box | Drug | RxNorm Extension |
| 1718462 | zidovudine 10 MG/ML Injection [Retrovir] | Branded Drug | Drug | RxNorm |
| 35767613 | darunavir 75 MG Delayed Release Oral Tablet [Prezista] by Janssen | Marketed Product | Drug | RxNorm Extension |
| 44183046 | abacavir 703 MG / Lamivudine 300 MG Oral Tablet [Kivexa] Box of 30 by Abacus Medicine | Marketed Product | Drug | RxNorm Extension |
| 1748985 | lopinavir 100 MG / ritonavir 25 MG Oral Tablet | Clinical Drug | Drug | RxNorm |
| 42927716 | tenofovir disoproxil 250 MG Oral Tablet [TENOFORIN] | Branded Drug | Drug | RxNorm Extension |
| 36235768 | Hivid Oral Product | Branded Dose Group | Drug | RxNorm |
| 43182790 | Ritonavir 100 MG Delayed Release Oral Tablet | Clinical Drug | Drug | RxNorm Extension |
| 40992055 | fosamprenavir 50 MG/ML Oral Solution | Clinical Drug | Drug | RxNorm Extension |
| 43022460 | darunavir 800 MG Oral Tablet Box of 30 by Zentiva | Marketed Product | Drug | RxNorm Extension |
| 41207012 | Lamivudine Delayed Release Oral Tablet [Lamivudin Hexal] | Branded Drug Form | Drug | RxNorm Extension |
| 41321875 | maraviroc 300 MG Oral Tablet [Celsentri] Box of 60 by Cc | Marketed Product | Drug | RxNorm Extension |
| 35860603 | saquinavir 200 MG Oral Capsule [Yinfulei] | Branded Drug | Drug | RxNorm Extension |
| 1511233 | lamivudine 300 MG / tenofovir disoproxil fumarate 300 MG Oral Tablet [Temixys] | Branded Drug | Drug | RxNorm |
| 21134572 | raltegravir Oral Solution | Clinical Drug Form | Drug | RxNorm Extension |
| 43180400 | abacavir 300 MG Delayed Release Oral Tablet [Ziagen] | Branded Drug | Drug | RxNorm Extension |
| 44061791 | abacavir 600 MG / Lamivudine 300 MG Oral Tablet [Mylan-Abacavir/Lamivudine] | Branded Drug | Drug | RxNorm Extension |
| 21033493 | lopinavir 133 MG / Ritonavir 33.3 MG Oral Capsule [Kaletra] Box of 180 | Branded Drug Box | Drug | RxNorm Extension |
| 21102875 | Zidovudine 100 MG Oral Capsule by Aurobindo | Marketed Product | Drug | RxNorm Extension |
| 35752147 | Lamivudine 150 MG Delayed Release Oral Tablet by A A H | Marketed Product | Drug | RxNorm Extension |
| 43732231 | Ritonavir 100 MG Oral Tablet [Ritonavir Mylan] | Branded Drug | Drug | RxNorm Extension |
| 44121980 | Didanosine Oral Powder [Videx Pediatric] | Branded Drug Form | Drug | RxNorm Extension |
| 42963129 | ombitasvir 12.5 MG / paritaprevir 75 MG / Ritonavir 50 MG [VIEKIRA] | Branded Drug Comp | Drug | RxNorm Extension |
| 2052736 | tenofovir disoproxil 300 MG Oral Tablet [FOVIED] by Korea United Pharm | Marketed Product | Drug | RxNorm Extension |
| 44204772 | 4000 MG Didanosine 1 MG/MG Oral Solution | Quant Clinical Drug | Drug | RxNorm Extension |
| 43028257 | dolutegravir / Rilpivirine Delayed Release Oral Tablet | Clinical Drug Form | Drug | RxNorm Extension |
| 44164477 | raltegravir Oral Powder | Clinical Drug Form | Drug | RxNorm Extension |
| 36248168 | Technivie Oral Product | Branded Dose Group | Drug | RxNorm |
| 1736971 | abacavir | Ingredient | Drug | RxNorm |
| 43136093 | tenofovir disoproxil Oral Granules [Viread] | Branded Drug Form | Drug | RxNorm Extension |
| 2052748 | tenofovir disoproxil 300 MG Oral Tablet [TENOFREE] | Branded Drug | Drug | RxNorm Extension |
| 19078496 | indinavir 200 MG Oral Capsule | Clinical Drug | Drug | RxNorm |
| 41435735 | 240 ML Lamivudine 0.0417 MG/ML Oral Solution [Epivir] | Quant Branded Drug | Drug | RxNorm Extension |
| 36897682 | {112 (dasabuvir 250 MG Oral Tablet) / 112 (ombitasvir 12.5 MG / paritaprevir 75 MG / Ritonavir 50 MG Oral Tablet) / 56 (Ribavirin 600 MG Oral Tablet) } Pack [Viekira Pak-Rbv] | Branded Pack | Drug | RxNorm Extension |
| 40855385 | efavirenz 600 MG Oral Tablet [Sustiva] Box of 30 by Gerke | Marketed Product | Drug | RxNorm Extension |
| 44091881 | Didanosine 4000 MG | Clinical Drug Comp | Drug | RxNorm Extension |
| 1715476 | nelfinavir 0.05 MG/MG Oral Powder | Clinical Drug | Drug | RxNorm |
| 41272807 | fosamprenavir 0.222 MG/ML Oral Solution Box of 1 | Clinical Drug Box | Drug | RxNorm Extension |
| 21169144 | abacavir 600 MG / dolutegravir 50 MG / Lamivudine 300 MG Oral Tablet Box of 30 | Clinical Drug Box | Drug | RxNorm Extension |
| 36889986 | Didanosine 100 MG Chewable Tablet [Videx] Box of 60 | Branded Drug Box | Drug | RxNorm Extension |
| 43039287 | 2 ML enfuvirtide 90 MG/ML Injectable Solution [Fuzeon] Box of 60 | Quant Branded Box | Drug | RxNorm Extension |
| 41211441 | efavirenz 600 MG Delayed Release Oral Tablet Box of 90 | Clinical Drug Box | Drug | RxNorm Extension |
| 43202118 | tenofovir disoproxil 123 MG | Clinical Drug Comp | Drug | RxNorm Extension |
| 994686 | emtricitabine / tenofovir disoproxil Delayed Release Oral Tablet [Emtenovo] | Branded Drug Form | Drug | RxNorm Extension |
| 40874080 | Nevirapine 200 MG Oral Tablet [Nevirapin Hexal] Box of 14 | Branded Drug Box | Drug | RxNorm Extension |
| 41471034 | 4040 MG Didanosine 1 MG/MG Oral Suspension Box of 1 | Quant Clinical Box | Drug | RxNorm Extension |
| 35743803 | Lamivudine 150 MG / Zidovudine 300 MG Delayed Release Oral Tablet [Combivir] by Mawdsley-Brooks | Marketed Product | Drug | RxNorm Extension |
| 43028263 | dolutegravir 50 MG / Rilpivirine 25 MG Delayed Release Oral Tablet [Juluca] Box of 30 by ViiV | Marketed Product | Drug | RxNorm Extension |
| 21072948 | Nevirapine 200 MG Oral Tablet by Morningside | Marketed Product | Drug | RxNorm Extension |
| 2047892 | stavudine 40 MG Oral Capsule [Zerit] by BMS | Marketed Product | Drug | RxNorm Extension |
| 35862023 | zidovudine Oral Tablet [Sanxiewei] | Branded Drug Form | Drug | RxNorm Extension |
| 35200454 | doravirine Oral Tablet [Pifeltro] | Branded Drug Form | Drug | RxNorm |
| 2050980 | didanosine 100 MG Oral Tablet [Videx] by BMS | Marketed Product | Drug | RxNorm Extension |
| 35860840 | ritonavir 50 MG [Kelizhi] | Branded Drug Comp | Drug | RxNorm Extension |
| 40975405 | abacavir 300 MG Delayed Release Oral Tablet [Abacavir Hexal] Box of 180 | Branded Drug Box | Drug | RxNorm Extension |
| 21060948 | cobicistat 150 MG Oral Tablet [Tybost] Box of 30 by Gilead | Marketed Product | Drug | RxNorm Extension |
| 44166954 | Ritonavir 100 MG Oral Tablet [Norvir] Box of 30 by Cambridge Major | Marketed Product | Drug | RxNorm Extension |
| 42875456 | DORAVIRINE 100 MG Oral Tablet [Pifeltro] Box of 90 | Branded Drug Box | Drug | RxNorm Extension |
| 41226850 | Ritonavir 100 MG Oral Capsule [Norvir] Box of 336 by Kohlpharma | Marketed Product | Drug | RxNorm Extension |
| 782648 | Nevirapine Extended Release Oral Capsule [NEVIRAPINE SANDOZ] | Branded Drug Form | Drug | RxNorm Extension |
| 40162291 | delavirdine mesylate 200 MG Oral Tablet | Clinical Drug | Drug | RxNorm |
| 2053040 | lamivudine 100 MG Oral Tablet [LAMIFFIX] by Kyungdong | Marketed Product | Drug | RxNorm Extension |
| 40925729 | fosamprenavir Oral Solution [Telzir] | Branded Drug Form | Drug | RxNorm Extension |
| 43034923 | Atazanavir 150 MG Oral Capsule Box of 60 by Mylan | Marketed Product | Drug | RxNorm Extension |
| 35768845 | Lamivudine 150 MG / Zidovudine 300 MG Delayed Release Oral Tablet Box of 60 by TEVA | Marketed Product | Drug | RxNorm Extension |
| 45892978 | cobicistat 150 MG / darunavir 800 MG Oral Tablet [Prezcobix] | Branded Drug | Drug | RxNorm |
| 44167258 | Nevirapine 400 MG Oral Tablet [Viramune] Box of 90 by Abacus Medicine | Marketed Product | Drug | RxNorm Extension |
| 41170647 | enfuvirtide 90 MG/ML | Clinical Drug Comp | Drug | RxNorm Extension |
| 21082604 | Lamivudine 100 MG Oral Tablet by TEVA | Marketed Product | Drug | RxNorm Extension |
| 35200466 | doravirine 100 MG / lamivudine 300 MG / tenofovir disoproxil fumarate 300 MG Oral Tablet | Clinical Drug | Drug | RxNorm |
| 43156338 | Lamivudine 150 MG / Zidovudine 300 MG Delayed Release Oral Tablet [LAMIVUDINE/ZIDOVUDINE MYLAN] Box of 60 by Mylan | Marketed Product | Drug | RxNorm Extension |
| 36895800 | 20 ML Zidovudine 10 MG/ML Prefilled Syringe Box of 5 | Quant Clinical Box | Drug | RxNorm Extension |
| 2011839 | tenofovir disoproxil 300 MG Oral Tablet [TENO-V] | Branded Drug | Drug | RxNorm Extension |
| 35410515 | emtricitabine 200 MG / tenofovir alafenamide 25 MG Delayed Release Oral Tablet Box of 30 | Clinical Drug Box | Drug | RxNorm Extension |
| 35200450 | doravirine Oral Tablet | Clinical Drug Form | Drug | RxNorm |
| 21132158 | Zidovudine 250 MG Oral Capsule by A A H | Marketed Product | Drug | RxNorm Extension |
| 40855383 | efavirenz 600 MG / emtricitabine 200 MG / tenofovir disoproxil 300 MG Oral Tablet [Atripla] Box of 90 by Beragena | Marketed Product | Drug | RxNorm Extension |
| 40945664 | Ritonavir 100 MG Oral Tablet [Norvir] Box of 90 by Canoma | Marketed Product | Drug | RxNorm Extension |
| 41211425 | emtricitabine 200 MG / tenofovir alafenamide 10 MG Oral Tablet Box of 30 | Clinical Drug Box | Drug | RxNorm Extension |
| 19129167 | stavudine 12 MG | Clinical Drug Comp | Drug | RxNorm |
| 1725065 | didanosine 2.5 MG/ML | Clinical Drug Comp | Drug | RxNorm |
| 21121798 | Ritonavir 100 MG Oral Capsule by Abbott | Marketed Product | Drug | RxNorm Extension |
| 41042360 | emtricitabine 200 MG Oral Capsule [Emtriva] Box of 30 by Gerke | Marketed Product | Drug | RxNorm Extension |
| 43204906 | Nelfinavir 250 MG Delayed Release Oral Tablet [Viracept] | Branded Drug | Drug | RxNorm Extension |
| 19010081 | didanosine 100 MG Chewable Tablet [Videx] | Branded Drug | Drug | RxNorm |
| 36222515 | Epivir HBV Pill | Branded Dose Group | Drug | RxNorm |
| 35200464 | doravirine / lamivudine / tenofovir disoproxil Oral Tablet | Clinical Drug Form | Drug | RxNorm |
| 35858565 | stavudine 15 MG Oral Capsule [Shazhi] | Branded Drug | Drug | RxNorm Extension |
| 21104934 | Atazanavir 200 MG Oral Capsule [Reyataz] Box of 60 | Branded Drug Box | Drug | RxNorm Extension |
| 41430811 | 180 ML efavirenz 0.167 MG/ML Oral Solution Box of 1 | Quant Clinical Box | Drug | RxNorm Extension |
| 41111364 | Nevirapine Oral Solution | Clinical Drug Form | Drug | RxNorm Extension |
| 43189265 | Lamivudine 150 MG / Zidovudine 300 MG [LAMIVUDINE/ZIDOVUDINE CRISTERS] | Branded Drug Comp | Drug | RxNorm Extension |
| 41277370 | Ritonavir 0.889 MG/ML Oral Solution [Norvir] Box of 1 | Branded Drug Box | Drug | RxNorm Extension |
| 43263864 | maraviroc 150 MG Oral Tablet Box of 180 | Clinical Drug Box | Drug | RxNorm Extension |
| 40825359 | efavirenz 600 MG / emtricitabine 200 MG / tenofovir disoproxil 300 MG Oral Tablet [Atripla] Box of 30 by Emra-Med | Marketed Product | Drug | RxNorm Extension |
| 41197948 | Lamivudine 150 MG / Zidovudine 300 MG Oral Tablet Box of 60 by Novartis | Marketed Product | Drug | RxNorm Extension |
| 40916125 | lopinavir 200 MG / Ritonavir 50 MG Oral Tablet [Kaletra] Box of 120 by Bb Farma | Marketed Product | Drug | RxNorm Extension |
| 21100338 | abacavir 600 MG / dolutegravir 50 MG / Lamivudine 300 MG Oral Tablet [Triumeq] Box of 30 by ViiV | Marketed Product | Drug | RxNorm Extension |
| 44095715 | Lamivudine / Zidovudine Oral Tablet [LAMIVUDINE/ZIDOVUDINE TEVA] | Branded Drug Form | Drug | RxNorm Extension |
| 21080573 | cobicistat 150 MG / elvitegravir 150 MG / emtricitabine 200 MG / tenofovir disoproxil 250 MG Oral Tablet [Stribild] Box of 30 | Branded Drug Box | Drug | RxNorm Extension |
| 40996626 | Ritonavir 100 MG Oral Tablet [Norvir] Box of 90 | Branded Drug Box | Drug | RxNorm Extension |
| 1715473 | nelfinavir 250 MG Oral Tablet [Viracept] | Branded Drug | Drug | RxNorm |
| 21161282 | Lamivudine 100 MG Oral Tablet [Zeffix] Box of 28 by Mawdsley-Brooks | Marketed Product | Drug | RxNorm Extension |
| 35862593 | emtricitabine 200 MG Oral Tablet [Huierding] | Branded Drug | Drug | RxNorm Extension |
| 40842858 | Nevirapine 400 MG Extended Release Oral Tablet [Nevirapin Ratiopharm] Box of 90 | Branded Drug Box | Drug | RxNorm Extension |
| 42965765 | emtricitabine 200 MG / Rilpivirine 25 MG / tenofovir disoproxil 250 MG Oral Tablet [Complera] | Branded Drug | Drug | RxNorm Extension |
| 1560081 | cobicistat 150 MG / darunavir 800 MG / emtricitabine 200 MG / tenofovir alafenamide 10 MG [Symtuza] | Branded Drug Comp | Drug | RxNorm |
| 40937841 | Lamivudine 300 MG Delayed Release Oral Tablet [Lamivudin Teva] Box of 30 | Branded Drug Box | Drug | RxNorm Extension |
| 19102236 | atazanavir 200 MG | Clinical Drug Comp | Drug | RxNorm |
| 41288584 | Saquinavir 500 MG Oral Tablet [Invirase] Box of 120 by Cc | Marketed Product | Drug | RxNorm Extension |
| 21124650 | maraviroc 300 MG Oral Tablet Box of 60 | Clinical Drug Box | Drug | RxNorm Extension |
| 35771931 | maraviroc 150 MG Delayed Release Oral Tablet [Celsentri] by ViiV | Marketed Product | Drug | RxNorm Extension |
| 40730836 | Lamivudine 150 MG Oral Tablet by Mylan | Marketed Product | Drug | RxNorm Extension |
| 21033503 | Nevirapine 200 MG Oral Tablet Box of 60 by Sandoz | Marketed Product | Drug | RxNorm Extension |
| 44785499 | raltegravir 100 MG Granules for Oral Suspension [Isentress] | Branded Drug | Drug | RxNorm |
| 43180399 | abacavir Delayed Release Oral Tablet | Clinical Drug Form | Drug | RxNorm Extension |
| 40733288 | maraviroc 75 MG Oral Tablet [Celsentri] by ViiV | Marketed Product | Drug | RxNorm Extension |
| 43178348 | Lamivudine 150 MG / Zidovudine 300 MG Delayed Release Oral Tablet [LAMIVUDINE/ZIDOVUDINE CRISTERS] Box of 60 | Branded Drug Box | Drug | RxNorm Extension |
| 41038514 | tenofovir disoproxil 250 MG Delayed Release Oral Tablet [Viread] Box of 90 by Beragena | Marketed Product | Drug | RxNorm Extension |
| 35862011 | zidovudine 100 MG [Kedu] | Branded Drug Comp | Drug | RxNorm Extension |
| 43146976 | darunavir 600 MG Delayed Release Oral Tablet [Prezista] Box of 60 by Janssen | Marketed Product | Drug | RxNorm Extension |
| 36061970 | atazanavir 200 MG Oral Capsule by A A H | Marketed Product | Drug | RxNorm Extension |
| 44182584 | fosamprenavir 700 MG Delayed Release Oral Tablet [Telzir] Box of 60 by Eurim-Pharm | Marketed Product | Drug | RxNorm Extension |
| 35146811 | tenofovir disoproxil 300 MG Oral Tablet [Tenozet] | Branded Drug | Drug | RxNorm Extension |
| 42941336 | Lamivudine Oral Tablet [HANMI VUDINE] | Branded Drug Form | Drug | RxNorm Extension |
| 41435384 | 200 ML Zidovudine 0.25 MG/ML Oral Solution [Retrovir] | Quant Branded Drug | Drug | RxNorm Extension |
| 40733285 | maraviroc 20 MG/ML [Celsentri] | Branded Drug Comp | Drug | RxNorm Extension |
| 40855106 | etravirine 200 MG Oral Tablet [Intelence] Box of 60 by Kohlpharma | Marketed Product | Drug | RxNorm Extension |
| 42705418 | raltegravir 25 MG [Isentress] | Branded Drug Comp | Drug | RxNorm |
| 41156729 | Lamivudine 150 MG Delayed Release Oral Tablet [Lamivudin Teva] Box of 30 | Branded Drug Box | Drug | RxNorm Extension |
| 43032450 | abacavir 600 MG / Lamivudine 300 MG Delayed Release Oral Tablet Box of 30 by Eurogenerics | Marketed Product | Drug | RxNorm Extension |
| 41280884 | Lamivudine 300 MG Oral Tablet [Lamivudin Teva] | Branded Drug | Drug | RxNorm Extension |
| 41435662 | 226 ML fosamprenavir 50 MG/ML Oral Solution [Telzir] Box of 1 | Quant Branded Box | Drug | RxNorm Extension |
| 21041342 | cobicistat 150 MG / elvitegravir 150 MG / emtricitabine 200 MG / Tenofovir 10 MG Oral Tablet Box of 30 | Clinical Drug Box | Drug | RxNorm Extension |
| 41136534 | emtricitabine 200 MG / Rilpivirine 25 MG / tenofovir disoproxil 300 MG Oral Tablet [Eviplera] Box of 30 by Canoma | Marketed Product | Drug | RxNorm Extension |
| 21140070 | Indinavir 333 MG Oral Capsule [Crixivan] Box of 135 | Branded Drug Box | Drug | RxNorm Extension |
| 40730828 | Lamivudine 100 MG Oral Tablet Box of 28 by Mylan | Marketed Product | Drug | RxNorm Extension |
| 40875589 | Lamivudine 0.0417 MG/ML Oral Solution [Epivir] | Branded Drug | Drug | RxNorm Extension |
| 41239951 | Stavudine 1 MG/ML Oral Suspension | Clinical Drug | Drug | RxNorm Extension |
| 44171218 | Lamivudine 150 MG / Zidovudine 300 MG Delayed Release Oral Tablet [Combivir] Box of 60 by European | Marketed Product | Drug | RxNorm Extension |
| 43146978 | darunavir 300 MG Delayed Release Oral Tablet [Prezista] | Branded Drug | Drug | RxNorm Extension |
| 42927742 | tenofovir disoproxil Oral Tablet [NEXREAD] | Branded Drug Form | Drug | RxNorm Extension |
| 40864787 | Zidovudine 0.25 MG/ML Oral Solution | Clinical Drug | Drug | RxNorm Extension |
| 43180174 | cobicistat / elvitegravir / emtricitabine / tenofovir disoproxil Delayed Release Oral Tablet | Clinical Drug Form | Drug | RxNorm Extension |
| 43023780 | Nevirapine 400 MG Extended Release Oral Tablet Box of 30 by Cristers | Marketed Product | Drug | RxNorm Extension |
| 36784669 | abacavir 600 MG / Lamivudine 300 MG [Abacavir / Lamivudin Sandoz] | Branded Drug Comp | Drug | RxNorm Extension |
| 793026 | dolutegravir / rilpivirine Pill | Clinical Dose Group | Drug | RxNorm |
| 21131779 | Amprenavir 150 MG Oral Capsule [Agenerase] Box of 240 | Branded Drug Box | Drug | RxNorm Extension |
| 40706774 | 1010 MG tenofovir disoproxil 0.033 MG/MG Oral Granules [Viread] | Quant Branded Drug | Drug | RxNorm Extension |
| 41165671 | Nevirapine 400 MG Oral Tablet [Viramune] Box of 30 by Medicopharm | Marketed Product | Drug | RxNorm Extension |
| 21169055 | cobicistat 150 MG / darunavir 800 MG Oral Tablet [Rezolsta] by Janssen | Marketed Product | Drug | RxNorm Extension |
| 36404479 | raltegravir 25 MG Oral Tablet [Isentress] | Branded Drug | Drug | RxNorm Extension |
| 42705925 | tenofovir disoproxil fumarate 0.04 MG/MG | Clinical Drug Comp | Drug | RxNorm |
| 40171778 | ritonavir 100 MG Oral Tablet | Clinical Drug | Drug | RxNorm |
| 43167286 | abacavir / dolutegravir / Lamivudine Delayed Release Oral Tablet [Triumeq] | Branded Drug Form | Drug | RxNorm Extension |
| 21039999 | 240 ML abacavir 20 MG/ML Oral Solution [Ziagen] by ViiV | Marketed Product | Drug | RxNorm Extension |
| 35861030 | abacavir Oral Solution [Saijin] | Branded Drug Form | Drug | RxNorm Extension |
| 36217381 | amprenavir Oral Liquid Product | Clinical Dose Group | Drug | RxNorm |
| 19116946 | zidovudine 300 MG [Retrovir] | Branded Drug Comp | Drug | RxNorm |
| 43696022 | Didanosine Delayed Release Oral Capsule [Didanosin Aurobindo] | Branded Drug Form | Drug | RxNorm Extension |
| 35771967 | darunavir 300 MG Delayed Release Oral Tablet [Prezista] by Janssen | Marketed Product | Drug | RxNorm Extension |
| 41088351 | abacavir 703 MG / Lamivudine 300 MG Oral Tablet | Clinical Drug | Drug | RxNorm Extension |
| 43171965 | Nelfinavir 250 MG Delayed Release Oral Tablet [Viracept] Box of 300 | Branded Drug Box | Drug | RxNorm Extension |
| 44171220 | Lamivudine 150 MG Delayed Release Oral Tablet [Epivir] Box of 60 by Mtk | Marketed Product | Drug | RxNorm Extension |
| 44082743 | Lamivudine / Zidovudine Oral Tablet [Auro-Lamivudine/Zidovudine] | Branded Drug Form | Drug | RxNorm Extension |
| 1592435 | ritonavir 100 MG Oral Powder | Clinical Drug | Drug | RxNorm |
| 36257399 | tenofovir disoproxil 250 MG Delayed Release Oral Tablet [Tenofovir Disoproxil Mylan] Box of 30 | Branded Drug Box | Drug | RxNorm Extension |
| 41435638 | 225 ML fosamprenavir 50 MG/ML Oral Solution | Quant Clinical Drug | Drug | RxNorm Extension |
| 41096914 | Didanosine 250 MG Extended Release Oral Capsule [Videx] Box of 30 | Branded Drug Box | Drug | RxNorm Extension |
| 43200295 | Lamivudine 150 MG / Zidovudine 300 MG Delayed Release Oral Tablet [LAMIVUDINE/ZIDOVUDINE SANDOZ] Box of 60 | Branded Drug Box | Drug | RxNorm Extension |
| 44101047 | Ritonavir 100 MG Oral Capsule [Norvir] Box of 2 by Abbvie | Marketed Product | Drug | RxNorm Extension |
| 43156337 | abacavir 300 MG / Lamivudine 150 MG / Zidovudine 300 MG Delayed Release Oral Tablet [Trizivir] Box of 60 by ViiV | Marketed Product | Drug | RxNorm Extension |
| 21100884 | Indinavir 200 MG Oral Capsule [Crixivan] Box of 360 by Merck | Marketed Product | Drug | RxNorm Extension |
| 21149923 | Indinavir 400 MG Oral Capsule Box of 180 | Clinical Drug Box | Drug | RxNorm Extension |
| 36062294 | lamivudine 300 MG / tenofovir disoproxil 250 MG Oral Tablet Box of 30 by Cipla | Marketed Product | Drug | RxNorm Extension |
| 41006780 | abacavir 703 MG / dolutegravir 50 MG / Lamivudine 300 MG Oral Tablet [Triumeq] | Branded Drug | Drug | RxNorm Extension |
| 43026161 | emtricitabine 200 MG / tenofovir disoproxil 250 MG Delayed Release Oral Tablet Box of 30 by Mylan | Marketed Product | Drug | RxNorm Extension |
| 35136179 | Lamivudine Oral Tablet [Zefix] | Branded Drug Form | Drug | RxNorm Extension |
| 41411994 | 1.1 ML enfuvirtide 90 MG/ML Injection [Fuzeon] Box of 60 by Gerke | Marketed Product | Drug | RxNorm Extension |
| 40972100 | darunavir 600 MG Oral Tablet [Prezista] Box of 30 | Branded Drug Box | Drug | RxNorm Extension |
| 40720634 | tenofovir disoproxil 250 MG Oral Tablet Box of 30 by Mylan | Marketed Product | Drug | RxNorm Extension |
| 21154084 | Atazanavir 100 MG Oral Capsule [Reyataz] Box of 60 by Bristol Myers Squibb | Marketed Product | Drug | RxNorm Extension |
| 36062159 | tenofovir disoproxil 163 MG Oral Tablet [Viread] Box of 30 | Branded Drug Box | Drug | RxNorm Extension |
| 40730815 | abacavir 600 MG / Lamivudine 300 MG Oral Tablet Box of 30 by Lupin | Marketed Product | Drug | RxNorm Extension |
| 43261490 | Lamivudine 300 MG [3tc] | Branded Drug Comp | Drug | RxNorm Extension |
| 43026752 | DORAVIRINE / Lamivudine / tenofovir disoproxil Delayed Release Oral Tablet [Delstrigo] | Branded Drug Form | Drug | RxNorm Extension |
| 35860796 | efavirenz 50 MG [Shiduoning] | Branded Drug Comp | Drug | RxNorm Extension |
| 36074476 | tenofovir disoproxil 250 MG Oral Tablet Box of 30 by Macleods | Marketed Product | Drug | RxNorm Extension |
| 40221116 | {3 (24 HR dasabuvir 200 MG / ombitasvir 8.33 MG / paritaprevir 50 MG / ritonavir 33.33 MG Extended Release Oral Tablet) } Pack | Clinical Pack | Drug | RxNorm |
| 41042359 | emtricitabine 200 MG / tenofovir disoproxil 300 MG Oral Tablet [Truvada] Box of 90 by Abacus Medicine | Marketed Product | Drug | RxNorm Extension |
| 2052749 | tenofovir disoproxil 300 MG Oral Tablet [TENOFREE] by Seoul | Marketed Product | Drug | RxNorm Extension |
| 21139717 | ombitasvir 12.5 MG / paritaprevir 75 MG / Ritonavir 50 MG Oral Tablet Box of 56 | Clinical Drug Box | Drug | RxNorm Extension |
| 19102201 | atazanavir 200 MG Oral Capsule [Reyataz] | Branded Drug | Drug | RxNorm |
| 42705927 | tenofovir disoproxil fumarate 0.04 MG/MG [Viread] | Branded Drug Comp | Drug | RxNorm |
| 2031631 | 1 ML ritonavir 80 MG/ML Oral Solution | Quant Clinical Drug | Drug | RxNorm Extension |
| 2052731 | tenofovir disoproxil 300 MG Oral Tablet [Viread] by Yuhan | Marketed Product | Drug | RxNorm Extension |
| 21161289 | Nevirapine 200 MG Oral Tablet by Creo | Marketed Product | Drug | RxNorm Extension |
| 36243648 | Stribild Pill | Branded Dose Group | Drug | RxNorm |
| 35605554 | cobicistat 150 MG / elvitegravir 150 MG / emtricitabine 200 MG / tenofovir alafenamide 10 MG [Genvoya] | Branded Drug Comp | Drug | RxNorm |
| 35606580 | emtricitabine 100 MG | Clinical Drug Comp | Drug | RxNorm |
| 41290901 | Lamivudine 150 MG / Zidovudine 300 MG Oral Tablet [Combivir] Box of 60 by Emra-Med | Marketed Product | Drug | RxNorm Extension |
| 40051184 | lamivudine Oral Solution [Epivir HBV] | Branded Drug Form | Drug | RxNorm |
| 43178107 | efavirenz / emtricitabine / tenofovir disoproxil Delayed Release Oral Tablet [Atripla] | Branded Drug Form | Drug | RxNorm Extension |
| 19042159 | emtricitabine 200 MG [Emtriva] | Branded Drug Comp | Drug | RxNorm |
| 40051187 | lamivudine Oral Tablet | Clinical Drug Form | Drug | RxNorm |
| 43211099 | Lamivudine 300 MG Delayed Release Oral Tablet [LAMIVUDINE MYLAN] Box of 30 by Mylan | Marketed Product | Drug | RxNorm Extension |
| 21043418 | abacavir 600 MG / Lamivudine 300 MG Oral Tablet [Kivexa] Box of 30 by ViiV | Marketed Product | Drug | RxNorm Extension |
| 44216219 | 144000 MG Nelfinavir 0.0585 MG/MG Oral Powder | Quant Clinical Drug | Drug | RxNorm Extension |
| 44171511 | Didanosine 400 MG Extended Release Oral Capsule [Videx] Box of 60 by Orifarm Leverkus | Marketed Product | Drug | RxNorm Extension |
| 42705414 | raltegravir Chewable Tablet | Clinical Drug Form | Drug | RxNorm |
| 1718279 | Isentress Granule Product | Branded Dose Group | Drug | RxNorm |
| 44205300 | 4030 MG Didanosine 1 MG/MG Oral Solution Box of 1 | Quant Clinical Box | Drug | RxNorm Extension |
| 41010082 | Lamivudine 150 MG Oral Tablet [Epivir] Box of 60 by Eurim-Pharm | Marketed Product | Drug | RxNorm Extension |
| 1511090 | Dovato Pill | Branded Dose Group | Drug | RxNorm |
| 40842857 | Nevirapine 200 MG Oral Tablet [Nevirapin Ratiopharm] Box of 200 | Branded Drug Box | Drug | RxNorm Extension |
| 36224278 | tipranavir Pill | Clinical Dose Group | Drug | RxNorm |
| 36272195 | etravirine Disintegrating Oral Tablet [Intelence] | Branded Drug Form | Drug | RxNorm Extension |
| 35746160 | Didanosine 150 MG Oral Tablet [Videx] Box of 60 | Branded Drug Box | Drug | RxNorm Extension |
| 43191283 | abacavir 300 MG Delayed Release Oral Tablet | Clinical Drug | Drug | RxNorm Extension |
| 44096910 | Ritonavir 100 MG Oral Capsule Box of 2 | Clinical Drug Box | Drug | RxNorm Extension |
| 21033494 | Lamivudine 150 MG Oral Tablet by TEVA | Marketed Product | Drug | RxNorm Extension |
| 21171239 | efavirenz 600 MG / emtricitabine 200 MG / tenofovir disoproxil 250 MG Oral Tablet [Atripla] by Gilead | Marketed Product | Drug | RxNorm Extension |
| 41178959 | Lamivudine 300 MG Oral Tablet Box of 80 | Clinical Drug Box | Drug | RxNorm Extension |
| 1769415 | nevirapine 200 MG | Clinical Drug Comp | Drug | RxNorm |
| 36230093 | Emtriva Oral Product | Branded Dose Group | Drug | RxNorm |
| 40975511 | Zidovudine 300 MG Oral Tablet [Retrovir] Box of 60 by Emra-Med | Marketed Product | Drug | RxNorm Extension |
| 43642065 | Didanosine 200 MG [Didanosin Aurobindo] | Branded Drug Comp | Drug | RxNorm Extension |
| 44175550 | abacavir 703 MG / dolutegravir 50 MG / Lamivudine 300 MG Oral Tablet [Triumeq] Box of 30 by Abacus Medicine | Marketed Product | Drug | RxNorm Extension |
| 36263870 | Nevirapine 100 MG Extended Release Oral Capsule [Viramune] Box of 90 by Boehringer Ingelheim | Marketed Product | Drug | RxNorm Extension |
| 21171053 | Lamivudine 150 MG / Zidovudine 300 MG Oral Tablet [Combivir] by ViiV | Marketed Product | Drug | RxNorm Extension |
| 41166551 | Lamivudine 100 MG Oral Tablet [LAMIVUDINE MYLAN] Box of 84 by Mylan | Marketed Product | Drug | RxNorm Extension |
| 45892973 | cobicistat / darunavir Oral Tablet | Clinical Drug Form | Drug | RxNorm |
| 36237503 | Edurant Pill | Branded Dose Group | Drug | RxNorm |
| 44178253 | raltegravir 400 MG Oral Tablet [Isentress] Box of 180 by Paranova Pack | Marketed Product | Drug | RxNorm Extension |
| 35764659 | Lamivudine 150 MG / Zidovudine 300 MG Delayed Release Oral Tablet by TEVA | Marketed Product | Drug | RxNorm Extension |
| 40835547 | Lamivudine 0.0208 MG/ML Oral Solution | Clinical Drug | Drug | RxNorm Extension |
| 35860789 | lopinavir Oral Solution [Kelizhi] | Branded Drug Form | Drug | RxNorm Extension |
| 41003134 | Didanosine 125 MG Delayed Release Oral Capsule [Videx] Box of 30 | Branded Drug Box | Drug | RxNorm Extension |
| 2052766 | tenofovir disoproxil 300 MG [TENOFREE] | Branded Drug Comp | Drug | RxNorm Extension |
| 35861989 | zidovudine 300 MG Oral Tablet [Kedu] | Branded Drug | Drug | RxNorm Extension |
| 35861968 | indinavir 200 MG Oral Tablet [Ouzhi] | Branded Drug | Drug | RxNorm Extension |
| 41205554 | fosamprenavir Oral Solution | Clinical Drug Form | Drug | RxNorm Extension |
| 44177533 | Didanosine 1 MG/MG Oral Solution [Videx] | Branded Drug | Drug | RxNorm Extension |
| 36061952 | atazanavir 300 MG Oral Capsule Box of 30 by Sandoz | Marketed Product | Drug | RxNorm Extension |
| 42927715 | tenofovir disoproxil 250 MG Oral Tablet [S-HEPA] | Branded Drug | Drug | RxNorm Extension |
| 21169667 | Indinavir 400 MG Oral Capsule Box of 90 | Clinical Drug Box | Drug | RxNorm Extension |
| 43180131 | darunavir 75 MG Delayed Release Oral Tablet [Prezista] Box of 480 by Janssen | Marketed Product | Drug | RxNorm Extension |
| 21140072 | Indinavir 100 MG Oral Capsule [Crixivan] Box of 180 by Merck | Marketed Product | Drug | RxNorm Extension |
| 40746915 | emtricitabine 200 MG / tenofovir disoproxil 250 MG Oral Tablet Box of 30 by A A H | Marketed Product | Drug | RxNorm Extension |
| 35861972 | indinavir 200 MG [Ouzhi] | Branded Drug Comp | Drug | RxNorm Extension |
| 42941334 | Lamivudine Oral Tablet [LAMIFFIX] | Branded Drug Form | Drug | RxNorm Extension |
| 41117646 | Didanosine 200 MG Oral Tablet Box of 60 | Clinical Drug Box | Drug | RxNorm Extension |
| 36409071 | fosamprenavir 115 MG Injectable Solution [Ivemend] | Branded Drug | Drug | RxNorm Extension |
| 41294032 | abacavir 703 MG / dolutegravir 50 MG / Lamivudine 300 MG Oral Tablet [Triumeq] Box of 30 by Cc | Marketed Product | Drug | RxNorm Extension |
| 36411418 | 95 ML tipranavir 100 MG/ML Oral Solution [Aptivus] by Boehringer Ingelheim | Marketed Product | Drug | RxNorm Extension |
| 21104772 | emtricitabine 200 MG Oral Capsule [Emtriva] by Gilead | Marketed Product | Drug | RxNorm Extension |
| 41435786 | 240 ML Nevirapine 0.0417 MG/ML Oral Solution [Viramune] Box of 1 | Quant Branded Box | Drug | RxNorm Extension |
| 36061599 | darunavir 800 MG Oral Tablet by Zentiva | Marketed Product | Drug | RxNorm Extension |
| 36274244 | Lamivudine 100 MG Oral Tablet [Zeffix] Box of 84 by GSK | Marketed Product | Drug | RxNorm Extension |
| 43269333 | maraviroc 150 MG Oral Tablet [Celsentri] Box of 90 | Branded Drug Box | Drug | RxNorm Extension |
| 43147024 | cobicistat 150 MG / elvitegravir 150 MG / emtricitabine 200 MG / tenofovir disoproxil 250 MG Delayed Release Oral Tablet Box of 30 | Clinical Drug Box | Drug | RxNorm Extension |
| 37592829 | dolutegravir 50 MG / Rilpivirine 25 MG Oral Tablet [Juluca] Box of 90 | Branded Drug Box | Drug | RxNorm Extension |
| 2052750 | tenofovir disoproxil 300 MG Oral Tablet [TENOVA] | Branded Drug | Drug | RxNorm Extension |
| 2053075 | lamivudine Oral Tablet [ZELAVUDINE] | Branded Drug Form | Drug | RxNorm Extension |
| 35860790 | efavirenz 600 MG Oral Tablet [Shiduoning] | Branded Drug | Drug | RxNorm Extension |
| 42927721 | tenofovir disoproxil 250 MG [TEFOVIR] | Branded Drug Comp | Drug | RxNorm Extension |
| 43151359 | Nevirapine 200 MG Oral Tablet [NEVIRAPINE SANDOZ] | Branded Drug | Drug | RxNorm Extension |
| 40130705 | emtricitabine Oral Solution [Emtriva] | Branded Drug Form | Drug | RxNorm |
| 35860780 | lopinavir 200 MG Oral Tablet [Kelizhi] | Branded Drug | Drug | RxNorm Extension |
| 35860839 | ritonavir 50 MG Oral Tablet [Kelizhi] | Branded Drug | Drug | RxNorm Extension |
| 45774772 | cobicistat 150 MG Oral Tablet [Tybost] | Branded Drug | Drug | RxNorm |
| 40720283 | raltegravir 100 MG Oral Granules [Isentress] Box of 60 | Branded Drug Box | Drug | RxNorm Extension |
| 43187422 | 200 ML darunavir 100 MG/ML Oral Solution [Prezista] Box of 1 | Quant Branded Box | Drug | RxNorm Extension |
| 43145152 | Lamivudine 150 MG Delayed Release Oral Tablet [LAMIVUDINE MYLAN] Box of 60 by Mylan | Marketed Product | Drug | RxNorm Extension |
| 43623448 | Lamivudine 150 MG / Zidovudine 300 MG [Lamizido] | Branded Drug Comp | Drug | RxNorm Extension |
| 35862766 | lamivudine 5 MG/ML [Hepuding] | Branded Drug Comp | Drug | RxNorm Extension |
| 21051767 | Indinavir 200 MG Oral Capsule [Crixivan] Box of 360 | Branded Drug Box | Drug | RxNorm Extension |
| 44178718 | Lamivudine 300 MG Delayed Release Oral Tablet [Epivir] Box of 30 by Axicorp | Marketed Product | Drug | RxNorm Extension |
| 21073134 | Amprenavir 150 MG Oral Capsule [Agenerase] Box of 240 by Glaxosmithkline | Marketed Product | Drug | RxNorm Extension |
| 35859811 | nevirapine 200 MG Oral Capsule [Liweier] | Branded Drug | Drug | RxNorm Extension |
| 40720280 | raltegravir 100 MG Granules for Oral Suspension [Isentress] by Merck | Marketed Product | Drug | RxNorm Extension |
| 40917581 | emtricitabine 200 MG / tenofovir alafenamide 25 MG Oral Tablet [Descovy] Box of 90 by Gilead | Marketed Product | Drug | RxNorm Extension |
| 43560387 | dolutegravir 50 MG Oral Tablet | Clinical Drug | Drug | RxNorm |
| 43785857 | Didanosine 200 MG Delayed Release Oral Capsule [Videx] by Bristol Myers Squibb | Marketed Product | Drug | RxNorm Extension |
| 44182209 | Nevirapine 400 MG Oral Tablet [Viramune] Box of 90 by Haematogmbh | Marketed Product | Drug | RxNorm Extension |
| 36507139 | darunavir 400 MG Oral Tablet by Betapharm | Marketed Product | Drug | RxNorm Extension |
| 35861964 | indinavir 400 MG Oral Capsule [Jiaxihuan] | Branded Drug | Drug | RxNorm Extension |
| 42874956 | Nevirapine 400 MG Delayed Release Oral Tablet Box of 90 by Accord | Marketed Product | Drug | RxNorm Extension |
| 44161263 | Didanosine 1 MG/MG Oral Solution | Clinical Drug | Drug | RxNorm Extension |
| 43140377 | Nevirapine 200 MG [NEVIRAPINE CRISTERS] | Branded Drug Comp | Drug | RxNorm Extension |
| 44174952 | Lamivudine 100 MG Delayed Release Oral Tablet [Zeffix] Box of 28 by Mtk | Marketed Product | Drug | RxNorm Extension |
| 43297764 | 240 ML Lamivudine 10 MG/ML Oral Solution [3tc] | Quant Branded Drug | Drug | RxNorm Extension |
| 21060951 | cobicistat 150 MG / darunavir 800 MG Oral Tablet Box of 30 | Clinical Drug Box | Drug | RxNorm Extension |
| 35751888 | Didanosine 250 MG Extended Release Oral Capsule [Videx EC] Box of 30 by Bristol Myers Squibb | Marketed Product | Drug | RxNorm Extension |
| 43156335 | abacavir / Lamivudine Delayed Release Oral Tablet | Clinical Drug Form | Drug | RxNorm Extension |
| 1710616 | zidovudine 100 MG Oral Capsule | Clinical Drug | Drug | RxNorm |
| 36217482 | delavirdine Pill | Clinical Dose Group | Drug | RxNorm |
| 40753356 | Nevirapine 400 MG Extended Release Oral Tablet by A A H | Marketed Product | Drug | RxNorm Extension |
| 43277958 | Lamivudine 300 MG Oral Tablet [3tc] | Branded Drug | Drug | RxNorm Extension |
| 43025640 | Lamivudine 150 MG / Zidovudine 300 MG Delayed Release Oral Tablet Box of 60 by Arrow | Marketed Product | Drug | RxNorm Extension |
| 41435809 | 240 ML Lamivudine 10 MG/ML Oral Solution [Epivir] Box of 1 by Gerke | Marketed Product | Drug | RxNorm Extension |
| 36248535 | cobicistat Oral Product | Clinical Dose Group | Drug | RxNorm |
| 44163577 | maraviroc 300 MG Oral Tablet [Celsentri] Box of 60 by Kohlpharma | Marketed Product | Drug | RxNorm Extension |
| 44113270 | abacavir 300 MG Oral Tablet [Apo-Abacavir] | Branded Drug | Drug | RxNorm Extension |
| 41367239 | 10 ML Zidovudine 20 MG/ML Injectable Solution | Quant Clinical Drug | Drug | RxNorm Extension |
| 40965072 | Saquinavir 500 MG Oral Tablet [Saquinavir Hexal] Box of 120 | Branded Drug Box | Drug | RxNorm Extension |
| 21134576 | fosamprenavir 115 MG Injection [Ivemend] by Merck | Marketed Product | Drug | RxNorm Extension |
| 43749411 | Zidovudine 250 MG Oral Capsule [Zidovudin Aurobindo] by Aurobindo | Marketed Product | Drug | RxNorm Extension |
| 35411828 | efavirenz 600 MG Delayed Release Oral Tablet [Efavirenz Teva] Box of 30 by TEVA | Marketed Product | Drug | RxNorm Extension |
| 40724591 | Ritonavir 100 MG Powder for Oral Suspension [Norvir] by Abbvie | Marketed Product | Drug | RxNorm Extension |
| 36061596 | darunavir 800 MG Oral Tablet by A A H | Marketed Product | Drug | RxNorm Extension |
| 41382002 | 20 ML Zidovudine 10 MG/ML Injectable Solution Box of 10 | Quant Clinical Box | Drug | RxNorm Extension |
| 21173675 | Atazanavir 100 MG Oral Capsule [Reyataz] Box of 60 | Branded Drug Box | Drug | RxNorm Extension |
| 21112314 | abacavir 300 MG Oral Tablet [Ziagen] by ViiV | Marketed Product | Drug | RxNorm Extension |
| 44170968 | ombitasvir 12.5 MG / paritaprevir 75 MG / Ritonavir 50 MG Oral Tablet [Viekirax] Box of 56 by Abacus Medicine | Marketed Product | Drug | RxNorm Extension |
| 35748011 | Lamivudine 150 MG / Zidovudine 300 MG Delayed Release Oral Tablet Box of 60 by Mylan | Marketed Product | Drug | RxNorm Extension |
| 43175843 | 60000 MG tenofovir disoproxil 0.033 MG/MG Oral Granules [Viread] | Quant Branded Drug | Drug | RxNorm Extension |
| 43202122 | emtricitabine 200 MG / tenofovir disoproxil 250 MG Delayed Release Oral Tablet [Truvada] Box of 30 | Branded Drug Box | Drug | RxNorm Extension |
| 41041202 | Lamivudine 150 MG Oral Tablet [Epivir] Box of 60 by Orifarm Leverkus | Marketed Product | Drug | RxNorm Extension |
| 2052721 | zidovudine 100 MG Oral Capsule [AZIDOMINE] by Chunggei | Marketed Product | Drug | RxNorm Extension |
| 41435790 | 240 ML abacavir 23.4 MG/ML Oral Solution [Ziagen] Box of 1 by ViiV | Marketed Product | Drug | RxNorm Extension |
| 41176026 | Didanosine Oral Capsule [Videx] | Branded Drug Form | Drug | RxNorm Extension |
| 44101098 | raltegravir 25 MG Chewable Tablet [Isentress] by Merck | Marketed Product | Drug | RxNorm Extension |
| 1715472 | nelfinavir | Ingredient | Drug | RxNorm |
| 44170585 | tenofovir disoproxil 250 MG Oral Tablet [Viread] Box of 90 by Abacus Medicine | Marketed Product | Drug | RxNorm Extension |
| 21147949 | 240 ML Lamivudine 10 MG/ML Oral Solution | Quant Clinical Drug | Drug | RxNorm Extension |
| 41086183 | emtricitabine 200 MG / tenofovir alafenamide 25 MG Oral Tablet Box of 30 | Clinical Drug Box | Drug | RxNorm Extension |
| 41404313 | 90 ML Ritonavir 0.889 MG/ML Oral Solution [Norvir] | Quant Branded Drug | Drug | RxNorm Extension |
| 40051182 | lamivudine Oral Capsule | Clinical Drug Form | Drug | RxNorm |
| 40097505 | abacavir Oral Tablet [Ziagen] | Branded Drug Form | Drug | RxNorm |
| 2052768 | tenofovir disoproxil 300 MG [TENOVIR] | Branded Drug Comp | Drug | RxNorm Extension |
| 41288218 | tenofovir disoproxil 250 MG Delayed Release Oral Tablet [Viread] Box of 30 by Adequapharm | Marketed Product | Drug | RxNorm Extension |
| 21075762 | darunavir 400 MG Oral Tablet [Prezista] Box of 60 | Branded Drug Box | Drug | RxNorm Extension |
| 21065614 | Atazanavir 200 MG Oral Capsule [Reyataz] by Bristol Myers Squibb | Marketed Product | Drug | RxNorm Extension |
| 41252912 | Didanosine 250 MG Delayed Release Oral Capsule [Videx] Box of 30 | Branded Drug Box | Drug | RxNorm Extension |
| 41190329 | efavirenz 600 MG Oral Tablet [Efavirenz Teva] | Branded Drug | Drug | RxNorm Extension |
| 43206860 | enfuvirtide 54 MG/ML [Fuzeon] | Branded Drug Comp | Drug | RxNorm Extension |
| 41197949 | Lamivudine 100 MG Delayed Release Oral Tablet [Lamivudin Teva] Box of 84 by Bb Farma | Marketed Product | Drug | RxNorm Extension |
| 43209113 | 144000 MG Nelfinavir 0.05 MG/MG Oral Powder [Viracept] by Roche | Marketed Product | Drug | RxNorm Extension |
| 40724604 | Ritonavir 100 MG Oral Capsule [Norvir] by Abbott | Marketed Product | Drug | RxNorm Extension |
| 41069115 | abacavir 300 MG Delayed Release Oral Tablet [Abacavir Hexal] | Branded Drug | Drug | RxNorm Extension |
| 36788487 | emtricitabine 200 MG / Rilpivirine 25 MG / tenofovir alafenamide 25 MG Delayed Release Oral Tablet [Odefsey] Box of 30 by Gilead | Marketed Product | Drug | RxNorm Extension |
| 21041343 | Atazanavir 300 MG / cobicistat 150 MG Oral Tablet [Evotaz] by Bristol Myers Squibb | Marketed Product | Drug | RxNorm Extension |
| 43189028 | efavirenz 600 MG Delayed Release Oral Tablet | Clinical Drug | Drug | RxNorm Extension |
| 782846 | efavirenz 600 MG / emtricitabine 200 MG / tenofovir disoproxil 250 MG Oral Tablet [Efavirenz / Emtricitabine / Tenofovir disoproxil Mylan] Box of 90 by Mylan | Marketed Product | Drug | RxNorm Extension |
| 42709025 | etravirine 25 MG Oral Tablet | Clinical Drug | Drug | RxNorm |
| 40937840 | Lamivudine 300 MG Oral Tablet [Lamivudin Teva] Box of 30 | Branded Drug Box | Drug | RxNorm Extension |
| 19112067 | stavudine 5 MG | Clinical Drug Comp | Drug | RxNorm |
| 42875915 | lopinavir 200 MG / Ritonavir 50 MG Oral Tablet [Kaletra] Box of 120 by Aaha | Marketed Product | Drug | RxNorm Extension |
| 43174803 | 2 ML enfuvirtide 54 MG/ML Prefilled Syringe | Quant Clinical Drug | Drug | RxNorm Extension |
| 964008 | bictegravir | Ingredient | Drug | RxNorm |
| 19131468 | tipranavir 100 MG/ML | Clinical Drug Comp | Drug | RxNorm |
| 44185984 | Nevirapine 400 MG Extended Release Oral Tablet [Viramune] Box of 30 by Abacus Medicine | Marketed Product | Drug | RxNorm Extension |
| 41290564 | maraviroc 300 MG Oral Tablet [Celsentri] Box of 60 by Cambridge | Marketed Product | Drug | RxNorm Extension |
| 40921138 | abacavir 23.4 MG/ML | Clinical Drug Comp | Drug | RxNorm Extension |
| 21082599 | Stavudine 15 MG Oral Capsule [Zerit] Box of 56 by Bristol Myers Squibb | Marketed Product | Drug | RxNorm Extension |
| 2052740 | tenofovir disoproxil 300 MG Oral Tablet [LIVERFORD] by Jinyang | Marketed Product | Drug | RxNorm Extension |
| 43153842 | 60 ML lopinavir 80 MG/ML / Ritonavir 20 MG/ML Oral Solution Box of 5 | Quant Clinical Box | Drug | RxNorm Extension |
| 19047768 | efavirenz 200 MG Oral Capsule [Sustiva] | Branded Drug | Drug | RxNorm |
| 21085166 | enfuvirtide 108 MG Injection Box of 60 | Clinical Drug Box | Drug | RxNorm Extension |
| 35772093 | emtricitabine 200 MG / Rilpivirine 25 MG / tenofovir disoproxil 250 MG Delayed Release Oral Tablet [Eviplera] by Gilead | Marketed Product | Drug | RxNorm Extension |
| 21080574 | cobicistat / darunavir Oral Tablet [Rezolsta] | Branded Drug Form | Drug | RxNorm Extension |
| 44186353 | etravirine 200 MG Oral Tablet [Intelence] Box of 60 by Abacus Medicine | Marketed Product | Drug | RxNorm Extension |
| 43026163 | emtricitabine 200 MG / tenofovir disoproxil 250 MG Delayed Release Oral Tablet Box of 30 by Eurogenerics | Marketed Product | Drug | RxNorm Extension |
| 1710654 | zidovudine 100 MG | Clinical Drug Comp | Drug | RxNorm |
| 19079684 | stavudine 20 MG Oral Capsule | Clinical Drug | Drug | RxNorm |
| 41101256 | tenofovir disoproxil 250 MG Oral Tablet [Viread] Box of 90 by Aca Mueller | Marketed Product | Drug | RxNorm Extension |
| 43191020 | darunavir 150 MG Delayed Release Oral Tablet [Prezista] Box of 240 by Janssen | Marketed Product | Drug | RxNorm Extension |
| 43028258 | dolutegravir / Rilpivirine Delayed Release Oral Tablet [Juluca] | Branded Drug Form | Drug | RxNorm Extension |
| 36061974 | atazanavir 150 MG Oral Capsule Box of 60 by Zentiva | Marketed Product | Drug | RxNorm Extension |
| 35859799 | nevirapine 200 MG Oral Capsule [Aiweining] | Branded Drug | Drug | RxNorm Extension |
| 43660865 | Nevirapine 200 MG Oral Tablet [Nevirapin Aurobindo] | Branded Drug | Drug | RxNorm Extension |
| 40720770 | raltegravir 600 MG Oral Tablet [Isentress] Box of 60 | Branded Drug Box | Drug | RxNorm Extension |
| 36219234 | zidovudine Injectable Product | Clinical Dose Group | Drug | RxNorm |
| 41282493 | fosamprenavir 0.222 MG/ML Oral Solution [Telzir] | Branded Drug | Drug | RxNorm Extension |
| 40742423 | enfuvirtide 108 MG Injectable Solution [Fuzeon] by Roche | Marketed Product | Drug | RxNorm Extension |
| 36261430 | tenofovir disoproxil 250 MG Delayed Release Oral Tablet [Tenofovir Disoproxil Mylan] Box of 30 by Mylan | Marketed Product | Drug | RxNorm Extension |
| 43200291 | abacavir 300 MG / Lamivudine 150 MG / Zidovudine 300 MG Delayed Release Oral Tablet [Trizivir] | Branded Drug | Drug | RxNorm Extension |
| 41136962 | darunavir 400 MG Delayed Release Oral Tablet [Prezista] Box of 60 by Tisida | Marketed Product | Drug | RxNorm Extension |
| 40978759 | Lamivudine 150 MG Oral Tablet [Epivir] Box of 60 by Emra-Med | Marketed Product | Drug | RxNorm Extension |
| 36788485 | cobicistat / darunavir / emtricitabine / tenofovir alafenamide Delayed Release Oral Tablet [Symtuza] | Branded Drug Form | Drug | RxNorm Extension |
| 21142103 | Zidovudine 10 MG/ML Oral Solution [Retrovir] by ViiV | Marketed Product | Drug | RxNorm Extension |
| 41191169 | darunavir 0.5 MG/ML Oral Solution [Prezista] | Branded Drug | Drug | RxNorm Extension |
| 43218581 | 2 ML enfuvirtide 54 MG/ML Prefilled Syringe Box of 60 | Quant Clinical Box | Drug | RxNorm Extension |
| 40071866 | nelfinavir Oral Powder | Clinical Drug Form | Drug | RxNorm |
| 41290898 | Lamivudine 150 MG / Zidovudine 300 MG Oral Tablet [Combivir] Box of 60 by Tisida | Marketed Product | Drug | RxNorm Extension |
| 35862015 | zidovudine 100 MG [Naxinde] | Branded Drug Comp | Drug | RxNorm Extension |
| 44180760 | Nelfinavir 0.0585 MG/MG Oral Powder [Viracept] | Branded Drug | Drug | RxNorm Extension |
| 44036307 | Ritonavir 80 MG/ML Oral Solution [Norvir] by Abbvie | Marketed Product | Drug | RxNorm Extension |
| 35606582 | emtricitabine 100 MG / tenofovir disoproxil fumarate 150 MG [Truvada] | Branded Drug Comp | Drug | RxNorm |
| 40720282 | raltegravir 100 MG Oral Granules [Isentress] Box of 60 by Merck | Marketed Product | Drug | RxNorm Extension |
| 41237236 | Stavudine Oral Suspension [Zerit] | Branded Drug Form | Drug | RxNorm Extension |
| 43022432 | darunavir 400 MG Delayed Release Oral Tablet Box of 60 by Mylan | Marketed Product | Drug | RxNorm Extension |
| 43022444 | darunavir 600 MG Oral Tablet Box of 60 by Zentiva | Marketed Product | Drug | RxNorm Extension |
| 41177701 | Ritonavir 100 MG Oral Tablet Box of 90 | Clinical Drug Box | Drug | RxNorm Extension |
| 21131578 | Nevirapine 10 MG/ML Oral Suspension [Viramune] by Boehringer Ingelheim | Marketed Product | Drug | RxNorm Extension |
| 41136544 | efavirenz 600 MG / emtricitabine 200 MG / tenofovir disoproxil 300 MG Oral Tablet [Atripla] Box of 30 by Medicopharm | Marketed Product | Drug | RxNorm Extension |
| 41463278 | 2000 MG Didanosine 1 MG/MG Oral Suspension [Videx] | Quant Branded Drug | Drug | RxNorm Extension |
| 21112109 | Lamivudine Oral Solution [Zeffix] | Branded Drug Form | Drug | RxNorm Extension |
| 36788473 | efavirenz 600 MG / emtricitabine 200 MG / tenofovir disoproxil 250 MG Delayed Release Oral Tablet [Efavirenz / Emtricitabine / Tenofovir disoproxil Mylan] Box of 90 | Branded Drug Box | Drug | RxNorm Extension |
| 1718478 | maraviroc 25 MG [Selzentry] | Branded Drug Comp | Drug | RxNorm |
| 41100704 | Zidovudine 250 MG Oral Capsule [Retrovir] Box of 40 by A+S Unicare | Marketed Product | Drug | RxNorm Extension |
| 41139245 | Lamivudine 0.0417 MG/ML | Clinical Drug Comp | Drug | RxNorm Extension |
| 21161280 | Lamivudine 100 MG Oral Tablet by Sandoz | Marketed Product | Drug | RxNorm Extension |
| 43642243 | Lamivudine 150 MG Oral Tablet [Lamivudin Hexal] | Branded Drug | Drug | RxNorm Extension |
| 41325360 | abacavir 703 MG / dolutegravir 50 MG / Lamivudine 300 MG Oral Tablet [Triumeq] Box of 90 by ViiV | Marketed Product | Drug | RxNorm Extension |
| 35862763 | lamivudine 10 MG/ML Oral Solution [Yipingwei] | Branded Drug | Drug | RxNorm Extension |
| 21051155 | cobicistat 150 MG / elvitegravir 150 MG / emtricitabine 200 MG / tenofovir disoproxil 250 MG [Stribild] | Branded Drug Comp | Drug | RxNorm Extension |
| 36236162 | Agenerase Oral Liquid Product | Branded Dose Group | Drug | RxNorm |
| 43839615 | Zidovudine 250 MG Oral Capsule [Zidovudin Aurobindo] | Branded Drug | Drug | RxNorm Extension |
| 43154372 | 180 ML efavirenz 30 MG/ML Oral Solution [Sustiva] Box of 1 | Quant Branded Box | Drug | RxNorm Extension |
| 35411296 | emtricitabine 200 MG / tenofovir alafenamide 10 MG Delayed Release Oral Tablet Box of 30 | Clinical Drug Box | Drug | RxNorm Extension |
| 41168140 | darunavir 600 MG Oral Tablet [Prezista] Box of 60 by Orifarm Leverkus | Marketed Product | Drug | RxNorm Extension |
| 1738167 | efavirenz 600 MG Oral Tablet [Sustiva] | Branded Drug | Drug | RxNorm |
| 43168991 | Rilpivirine 25 MG Delayed Release Oral Tablet [Edurant] Box of 30 by Janssen | Marketed Product | Drug | RxNorm Extension |
| 43140836 | Zidovudine 300 MG Delayed Release Oral Tablet [Retrovir] Box of 60 | Branded Drug Box | Drug | RxNorm Extension |
| 35860795 | efavirenz 50 MG Oral Tablet [Shiduoning] | Branded Drug | Drug | RxNorm Extension |
| 21032700 | Nelfinavir 625 MG Oral Tablet [Viracept] Box of 120 | Branded Drug Box | Drug | RxNorm Extension |
| 41072090 | maraviroc 150 MG Delayed Release Oral Tablet [Celsentri] Box of 60 by Emra-Med | Marketed Product | Drug | RxNorm Extension |
| 43202073 | darunavir Delayed Release Oral Tablet [Prezista] | Branded Drug Form | Drug | RxNorm Extension |
| 35752146 | Lamivudine 150 MG Delayed Release Oral Tablet Box of 60 by TEVA | Marketed Product | Drug | RxNorm Extension |
| 43167284 | Lamivudine 5 MG/ML Oral Solution [Zeffix] Box of 1 | Branded Drug Box | Drug | RxNorm Extension |
| 36062292 | lamivudine 300 MG / tenofovir disoproxil 250 MG Oral Tablet Box of 90 by Cipla | Marketed Product | Drug | RxNorm Extension |
| 21138091 | 240 ML Nevirapine 10 MG/ML Oral Suspension [Viramune] | Quant Branded Drug | Drug | RxNorm Extension |
| 42927717 | tenofovir disoproxil 250 MG Oral Tablet [TENOLID] | Branded Drug | Drug | RxNorm Extension |
| 41258928 | Nevirapine 200 MG Oral Tablet [Nevirapin Hexal] Box of 120 by Novartis | Marketed Product | Drug | RxNorm Extension |
| 1769413 | nevirapine 10 MG/ML Oral Suspension | Clinical Drug | Drug | RxNorm |
| 41435782 | 240 ML Nevirapine 0.0417 MG/ML Oral Solution Box of 1 | Quant Clinical Box | Drug | RxNorm Extension |
| 1781438 | stavudine 20 MG | Clinical Drug Comp | Drug | RxNorm |
| 35861997 | zidovudine 250 MG Oral Capsule [Lituowei] | Branded Drug | Drug | RxNorm Extension |
| 35862598 | emtricitabine 200 MG [Xinluoshu] | Branded Drug Comp | Drug | RxNorm Extension |
| 45892742 | atazanavir 50 MG [Reyataz] | Branded Drug Comp | Drug | RxNorm |
| 21061185 | ombitasvir 12.5 MG / paritaprevir 75 MG / Ritonavir 50 MG [Viekirax] | Branded Drug Comp | Drug | RxNorm Extension |
| 41071596 | Nevirapine 200 MG Oral Tablet [Nevirapin Hexal] Box of 60 by Novartis | Marketed Product | Drug | RxNorm Extension |
| 35860788 | lopinavir Oral Solution | Clinical Drug Form | Drug | RxNorm Extension |
| 41293605 | Atazanavir 300 MG Oral Capsule [Reyataz] Box of 90 by European | Marketed Product | Drug | RxNorm Extension |
| 2052719 | zidovudine 250 MG Oral Capsule [AZIDOMINE] by Aprogen | Marketed Product | Drug | RxNorm Extension |
| 43189029 | efavirenz 600 MG Delayed Release Oral Tablet [EFAVIRENZ SANDOZ] Box of 30 by Sandoz | Marketed Product | Drug | RxNorm Extension |
| 19124992 | atazanavir 300 MG | Clinical Drug Comp | Drug | RxNorm |
| 1711526 | indinavir 333 MG Oral Capsule [Crixivan] | Branded Drug | Drug | RxNorm |
| 40730826 | Lamivudine 100 MG Oral Tablet [Zeffix] by Necessity Supplies | Marketed Product | Drug | RxNorm Extension |
| 37593310 | Atazanavir Oral Granules [Reyataz] | Branded Drug Form | Drug | RxNorm Extension |
| 40825633 | tenofovir disoproxil 250 MG Oral Tablet [Viread] Box of 30 by Gerke | Marketed Product | Drug | RxNorm Extension |
| 41040308 | Nevirapine 200 MG Oral Tablet [Viramune] Box of 120 by Eurim-Pharm | Marketed Product | Drug | RxNorm Extension |
| 36061594 | darunavir 800 MG Oral Tablet Box of 30 by Accord | Marketed Product | Drug | RxNorm Extension |
| 21053218 | Nevirapine 100 MG Extended Release Oral Tablet [Viramune] Box of 90 by Boehringer Ingelheim | Marketed Product | Drug | RxNorm Extension |
| 21056141 | etravirine 100 MG Oral Tablet [Intelence] Box of 120 | Branded Drug Box | Drug | RxNorm Extension |
| 40945268 | Stavudine 40 MG Oral Capsule [Zerit] Box of 56 by Kohlpharma | Marketed Product | Drug | RxNorm Extension |
| 41303337 | Lamivudine 0.0208 MG/ML Oral Solution Box of 1 | Clinical Drug Box | Drug | RxNorm Extension |
| 43146979 | darunavir 300 MG Delayed Release Oral Tablet [Prezista] Box of 120 by Janssen | Marketed Product | Drug | RxNorm Extension |
| 1510224 | efavirenz / lamivudine / tenofovir disoproxil Oral Tablet [Symfi] | Branded Drug Form | Drug | RxNorm |
| 41135084 | lopinavir 200 MG / Ritonavir 50 MG Oral Tablet [Kaletra] Box of 120 by Docpharm | Marketed Product | Drug | RxNorm Extension |
| 41186293 | Nevirapine 200 MG Oral Tablet [Nevirapin Aurobindo] Box of 14 | Branded Drug Box | Drug | RxNorm Extension |
| 40978755 | Lamivudine 100 MG Oral Tablet [Zeffix] Box of 28 by Eurim-Pharm | Marketed Product | Drug | RxNorm Extension |
| 41062397 | Lamivudine 150 MG Oral Tablet [Lamivudin Hexal] Box of 60 | Branded Drug Box | Drug | RxNorm Extension |
| 40959675 | Nevirapine 400 MG Extended Release Oral Tablet Box of 60 | Clinical Drug Box | Drug | RxNorm Extension |
| 35200468 | doravirine 100 MG / lamivudine 300 MG / tenofovir disoproxil fumarate 300 MG Oral Tablet [Delstrigo] | Branded Drug | Drug | RxNorm |
| 42874783 | darunavir 800 MG Delayed Release Oral Tablet Box of 30 by Betapharm | Marketed Product | Drug | RxNorm Extension |
| 43200286 | Lamivudine 300 MG Delayed Release Oral Tablet Box of 30 | Clinical Drug Box | Drug | RxNorm Extension |
| 43143988 | Didanosine 250 MG Extended Release Oral Tablet [Videx] Box of 30 by Bristol Myers Squibb | Marketed Product | Drug | RxNorm Extension |
| 2052786 | tenofovir disoproxil 250 MG [TENOFIN] | Branded Drug Comp | Drug | RxNorm Extension |
| 43265387 | cobicistat 150 MG / elvitegravir 150 MG / emtricitabine 200 MG / tenofovir alafenamide 10 MG Oral Tablet Box of 30 | Clinical Drug Box | Drug | RxNorm Extension |
| 2011843 | tenofovir disoproxil Oral Tablet [TENOLID-F] | Branded Drug Form | Drug | RxNorm Extension |
| 21026526 | tipranavir 100 MG/ML Oral Solution by Boehringer Ingelheim | Marketed Product | Drug | RxNorm Extension |
| 43022221 | raltegravir 600 MG Delayed Release Oral Tablet [Isentress] Box of 60 by Merck | Marketed Product | Drug | RxNorm Extension |
| 19082373 | ritonavir 100 MG Oral Capsule | Clinical Drug | Drug | RxNorm |
| 43519084 | 95 ML tipranavir 100 MG/ML Oral Solution [Aptivus] | Quant Branded Drug | Drug | RxNorm Extension |
| 40220794 | dasabuvir / ombitasvir / paritaprevir / ritonavir Pill | Clinical Dose Group | Drug | RxNorm |
| 43515342 | Atazanavir 300 MG Oral Capsule [Reyataz] Box of 60 | Branded Drug Box | Drug | RxNorm Extension |
| 43031552 | maraviroc 75 MG/ML [Celsentri] | Branded Drug Comp | Drug | RxNorm Extension |
| 1769414 | nevirapine 10 MG/ML | Clinical Drug Comp | Drug | RxNorm |
| 43031557 | maraviroc 75 MG Delayed Release Oral Tablet [Celsentri] | Branded Drug | Drug | RxNorm Extension |
| 35860785 | lopinavir 80 MG/ML [Kelizhi] | Branded Drug Comp | Drug | RxNorm Extension |
| 43200289 | abacavir 600 MG / dolutegravir 50 MG / Lamivudine 300 MG Delayed Release Oral Tablet Box of 30 | Clinical Drug Box | Drug | RxNorm Extension |
| 36219267 | abacavir / lamivudine / zidovudine Pill | Clinical Dose Group | Drug | RxNorm |
| 19084909 | didanosine 400 MG | Clinical Drug Comp | Drug | RxNorm |
| 21121986 | efavirenz 100 MG Oral Capsule [Sustiva] Box of 30 by Bristol Myers Squibb | Marketed Product | Drug | RxNorm Extension |
| 45892741 | atazanavir 50 MG Oral Powder | Clinical Drug | Drug | RxNorm |
| 41320002 | Saquinavir 500 MG Oral Tablet [Invirase] Box of 120 by Medicopharm | Marketed Product | Drug | RxNorm Extension |
| 2050590 | lopinavir 100 MG / ritonavir 25 MG Oral Tablet [Kaletra] by Abbott | Marketed Product | Drug | RxNorm Extension |
| 36061904 | lopinavir 200 MG / ritonavir 50 MG Delayed Release Oral Tablet Box of 120 by Accord | Marketed Product | Drug | RxNorm Extension |
| 2052742 | tenofovir disoproxil 300 MG Oral Tablet [TENOFO] by NBK | Marketed Product | Drug | RxNorm Extension |
| 40869878 | abacavir 703 MG / dolutegravir 50 MG / Lamivudine 300 MG Oral Tablet Box of 30 | Clinical Drug Box | Drug | RxNorm Extension |
| 21171052 | Lamivudine 150 MG / Zidovudine 300 MG Oral Tablet Box of 60 by TEVA | Marketed Product | Drug | RxNorm Extension |
| 36404313 | fosamprenavir 150 MG Injectable Solution | Clinical Drug | Drug | RxNorm Extension |
| 21030149 | 200 ML darunavir 100 MG/ML Oral Suspension [Prezista] | Quant Branded Drug | Drug | RxNorm Extension |
| 36506462 | tenofovir disoproxil 250 MG Delayed Release Oral Tablet by Puren | Marketed Product | Drug | RxNorm Extension |
| 43840211 | Lamivudine 100 MG Oral Tablet [Lamivir] | Branded Drug | Drug | RxNorm Extension |
| 40161653 | darunavir 150 MG [Prezista] | Branded Drug Comp | Drug | RxNorm |
| 21092967 | Zidovudine 100 MG Oral Capsule Box of 60 by Aurobindo | Marketed Product | Drug | RxNorm Extension |
| 41135306 | Lamivudine 100 MG Delayed Release Oral Tablet [Lamivir] Box of 28 by Medicopharm | Marketed Product | Drug | RxNorm Extension |
| 40885498 | Lamivudine 150 MG Oral Tablet [Lamivudin Teva] Box of 80 by Ratiopharm | Marketed Product | Drug | RxNorm Extension |
| 40945117 | tenofovir disoproxil 250 MG Oral Tablet [Viread] Box of 90 by Cc | Marketed Product | Drug | RxNorm Extension |
| 21149924 | Saquinavir 500 MG Oral Tablet [Invirase] Box of 120 | Branded Drug Box | Drug | RxNorm Extension |
| 40887121 | darunavir 400 MG Oral Tablet [Prezista] Box of 60 by Cambridge | Marketed Product | Drug | RxNorm Extension |
| 40975404 | abacavir 703 MG / Lamivudine 300 MG Oral Tablet [Kivexa] Box of 90 | Branded Drug Box | Drug | RxNorm Extension |
| 40072742 | nevirapine Oral Tablet | Clinical Drug Form | Drug | RxNorm |
| 21112111 | Lamivudine 100 MG Oral Tablet [Zeffix] Box of 28 by Glaxosmithkline | Marketed Product | Drug | RxNorm Extension |
| 42927704 | tenofovir disoproxil 250 MG Oral Tablet [TECAVIR] | Branded Drug | Drug | RxNorm Extension |
| 35771968 | darunavir 100 MG/ML Oral Solution [Prezista] by Janssen | Marketed Product | Drug | RxNorm Extension |
| 41275457 | abacavir 703 MG / Lamivudine 300 MG Oral Tablet Box of 90 | Clinical Drug Box | Drug | RxNorm Extension |
| 35604228 | emtricitabine / tenofovir alafenamide Oral Tablet [Descovy] | Branded Drug Form | Drug | RxNorm |
| 44159282 | tenofovir disoproxil 250 MG Oral Tablet [Viread] Box of 90 by Cambridge Major | Marketed Product | Drug | RxNorm Extension |
| 21063036 | lopinavir 200 MG / Ritonavir 50 MG Oral Tablet [Kaletra] Box of 120 | Branded Drug Box | Drug | RxNorm Extension |
| 44082744 | Lamivudine / Zidovudine Oral Tablet [Apo-Lamivudine-Zidovudine] | Branded Drug Form | Drug | RxNorm Extension |
| 43180132 | darunavir 300 MG Delayed Release Oral Tablet Box of 120 | Clinical Drug Box | Drug | RxNorm Extension |
| 43144915 | efavirenz 600 MG [EFAVIRENZ SANDOZ] | Branded Drug Comp | Drug | RxNorm Extension |
| 44050940 | Didanosine 25 MG Oral Tablet [Videx] by Bristol Myers Squibb | Marketed Product | Drug | RxNorm Extension |
| 44161036 | raltegravir 100 MG Oral Powder | Clinical Drug | Drug | RxNorm Extension |
| 43288720 | Lamivudine Oral Tablet [Zetlam] | Branded Drug Form | Drug | RxNorm Extension |
| 21102307 | abacavir 600 MG / Lamivudine 300 MG Oral Tablet [Kivexa] | Branded Drug | Drug | RxNorm Extension |
| 40921943 | Nevirapine 0.0417 MG/ML [Viramune] | Branded Drug Comp | Drug | RxNorm Extension |
| 19058557 | abacavir 20 MG/ML Oral Solution | Clinical Drug | Drug | RxNorm |
| 40241980 | emtricitabine / rilpivirine / tenofovir disoproxil Oral Tablet | Clinical Drug Form | Drug | RxNorm |
| 36061901 | lopinavir 200 MG / ritonavir 50 MG Oral Tablet by A A H | Marketed Product | Drug | RxNorm Extension |
| 40724595 | Ritonavir 100 MG Oral Tablet Box of 30 by A A H | Marketed Product | Drug | RxNorm Extension |
| 37593313 | Atazanavir 0.0333 MG/MG Oral Granules [Reyataz] | Branded Drug | Drug | RxNorm Extension |
| 36222272 | etravirine Pill | Clinical Dose Group | Drug | RxNorm |
| 40945669 | Ritonavir 100 MG Oral Tablet [Norvir] Box of 30 by Axicorp | Marketed Product | Drug | RxNorm Extension |
| 21055923 | tipranavir 250 MG Oral Capsule Box of 120 by Boehringer Ingelheim | Marketed Product | Drug | RxNorm Extension |
| 42901583 | darunavir 800 MG [Prezista] | Branded Drug Comp | Drug | RxNorm |
| 43169085 | cobicistat 150 MG / elvitegravir 150 MG / emtricitabine 200 MG / tenofovir disoproxil 250 MG Delayed Release Oral Tablet [Stribild] Box of 30 by Gilead | Marketed Product | Drug | RxNorm Extension |
| 41134412 | Nevirapine 200 MG Oral Tablet [Viramune] Box of 60 by Aca Mueller | Marketed Product | Drug | RxNorm Extension |
| 41008133 | raltegravir 400 MG Oral Tablet [Isentress] Box of 60 by Kohlpharma | Marketed Product | Drug | RxNorm Extension |
| 35862602 | emtricitabine Oral Capsule [Xinluoshu] | Branded Drug Form | Drug | RxNorm Extension |
| 40924860 | Saquinavir Delayed Release Oral Tablet [Saquinavir Hexal] | Branded Drug Form | Drug | RxNorm Extension |
| 42927735 | tenofovir disoproxil 250 MG [TEREAD] | Branded Drug Comp | Drug | RxNorm Extension |
| 1511088 | dolutegravir / lamivudine Oral Tablet [Dovato] | Branded Drug Form | Drug | RxNorm |
| 36273917 | abacavir 300 MG / Lamivudine 150 MG / Zidovudine 300 MG Oral Tablet [Trizivir] Box of 60 by ViiV | Marketed Product | Drug | RxNorm Extension |
| 40140031 | didanosine Delayed Release Oral Capsule [Videx] | Branded Drug Form | Drug | RxNorm |
| 36240291 | Reyataz Pill | Branded Dose Group | Drug | RxNorm |
| 43261491 | Lamivudine 300 MG Oral Tablet by Ranbaxy | Marketed Product | Drug | RxNorm Extension |
| 43202367 | abacavir 300 MG Delayed Release Oral Tablet [Ziagen] Box of 60 | Branded Drug Box | Drug | RxNorm Extension |
| 35764671 | lopinavir 100 MG / Ritonavir 25 MG Delayed Release Oral Tablet [Kaletra] by Abbvie | Marketed Product | Drug | RxNorm Extension |
| 45892977 | cobicistat / darunavir Oral Tablet [Prezcobix] | Branded Drug Form | Drug | RxNorm |
| 36259586 | Nevirapine 100 MG Extended Release Oral Capsule Box of 90 | Clinical Drug Box | Drug | RxNorm Extension |
| 21151824 | Didanosine 150 MG Chewable Tablet Box of 60 | Clinical Drug Box | Drug | RxNorm Extension |
| 19125236 | darunavir 600 MG | Clinical Drug Comp | Drug | RxNorm |
| 19107920 | zidovudine 200 MG Oral Tablet | Clinical Drug | Drug | RxNorm |
| 43803833 | Didanosine 250 MG Delayed Release Oral Capsule [Didanosin Aurobindo] by Aurobindo | Marketed Product | Drug | RxNorm Extension |
| 41239853 | tenofovir disoproxil 250 MG Oral Tablet Box of 30 | Clinical Drug Box | Drug | RxNorm Extension |
| 43146975 | darunavir 600 MG Delayed Release Oral Tablet [Prezista] | Branded Drug | Drug | RxNorm Extension |
| 36061953 | atazanavir 300 MG Oral Capsule Box of 30 by Dr Reddy's | Marketed Product | Drug | RxNorm Extension |
| 19131908 | tipranavir 100 MG/ML Oral Solution [Aptivus] | Branded Drug | Drug | RxNorm |
| 42708121 | tenofovir disoproxil fumarate 0.04 MG/MG Oral Powder [Viread] | Branded Drug | Drug | RxNorm |
| 43209325 | 240 ML Nevirapine 10 MG/ML Oral Suspension [Viramune] Box of 1 | Quant Branded Box | Drug | RxNorm Extension |
| 35746161 | Didanosine 25 MG Oral Tablet [Videx] Box of 60 | Branded Drug Box | Drug | RxNorm Extension |
| 43298357 | {56 (dasabuvir 250 MG Oral Tablet) / 56 (ombitasvir 12.5 MG / paritaprevir 75 MG / Ritonavir 50 MG Oral Tablet) / 56 (Ribavirin 600 MG Oral Tablet) } Pack box of 1 | Clinical Pack Box | Drug | RxNorm Extension |
| 35764670 | Lamivudine 100 MG Delayed Release Oral Tablet by Sigma-Tau | Marketed Product | Drug | RxNorm Extension |
| 1718550 | didanosine 10 MG/ML [Videx] | Branded Drug Comp | Drug | RxNorm |
| 41323598 | dolutegravir 50 MG Oral Tablet [Tivicay] Box of 30 by European | Marketed Product | Drug | RxNorm Extension |
| 36061612 | darunavir 400 MG Oral Tablet by Zentiva | Marketed Product | Drug | RxNorm Extension |
| 41007597 | Stavudine 30 MG Oral Capsule [Zerit] Box of 56 by Eurim-Pharm | Marketed Product | Drug | RxNorm Extension |
| 21092416 | Lamivudine 150 MG / Zidovudine 300 MG Oral Tablet by Sandoz | Marketed Product | Drug | RxNorm Extension |
| 42941327 | Lamivudine 5 MG/ML Oral Suspension | Clinical Drug | Drug | RxNorm Extension |
| 40971274 | efavirenz 600 MG Delayed Release Oral Tablet [Sustiva] Box of 90 | Branded Drug Box | Drug | RxNorm Extension |
| 36273748 | Didanosine 200 MG Delayed Release Oral Capsule [Videx] Box of 30 by Bristol Myers Squibb | Marketed Product | Drug | RxNorm Extension |
| 36239209 | Trizivir Pill | Branded Dose Group | Drug | RxNorm |
| 35862012 | zidovudine 100 MG [Qiluoke] | Branded Drug Comp | Drug | RxNorm Extension |
| 43609440 | 5 ML Zidovudine 10 MG/ML Oral Solution | Quant Clinical Drug | Drug | RxNorm Extension |
| 41086366 | Didanosine 250 MG Extended Release Oral Capsule Box of 30 | Clinical Drug Box | Drug | RxNorm Extension |
| 21082833 | efavirenz 50 MG Oral Capsule [Sustiva] Box of 30 | Branded Drug Box | Drug | RxNorm Extension |
| 21114740 | Atazanavir 150 MG Oral Capsule Box of 60 | Clinical Drug Box | Drug | RxNorm Extension |
| 43184389 | Nevirapine 10 MG/ML Oral Suspension [Viramune] Box of 1 | Branded Drug Box | Drug | RxNorm Extension |
| 41302840 | Nevirapine 200 MG Oral Tablet Box of 180 | Clinical Drug Box | Drug | RxNorm Extension |
| 41236246 | abacavir 351 MG / Lamivudine 150 MG / Zidovudine 300 MG [Trizivir] | Branded Drug Comp | Drug | RxNorm Extension |
| 40882156 | abacavir 300 MG Delayed Release Oral Tablet [Abacavir Hexal] Box of 60 | Branded Drug Box | Drug | RxNorm Extension |
| 40838797 | abacavir 351 MG / Lamivudine 150 MG / Zidovudine 300 MG Oral Tablet Box of 40 | Clinical Drug Box | Drug | RxNorm Extension |
| 41151164 | abacavir 300 MG Oral Tablet Box of 180 | Clinical Drug Box | Drug | RxNorm Extension |
| 36412246 | 450 ML Ritonavir 80 MG/ML Oral Solution [Norvir] | Quant Branded Drug | Drug | RxNorm Extension |
| 40154238 | tipranavir Oral Solution [Aptivus] | Branded Drug Form | Drug | RxNorm |
| 36783447 | cobicistat 150 MG / darunavir 800 MG Delayed Release Oral Tablet [Rezolsta] | Branded Drug | Drug | RxNorm Extension |
| 36218528 | Isentress Pill | Branded Dose Group | Drug | RxNorm |
| 36061903 | lopinavir 200 MG / ritonavir 50 MG Delayed Release Oral Tablet Box of 120 by A A H | Marketed Product | Drug | RxNorm Extension |
| 40906480 | Lamivudine 100 MG Delayed Release Oral Tablet [LAMIVUDINE MYLAN] Box of 28 | Branded Drug Box | Drug | RxNorm Extension |
| 36778127 | 240 ML Lamivudine 5 MG/ML Oral Solution [Zeffix] Box of 1 by GSK | Marketed Product | Drug | RxNorm Extension |
| 41260983 | efavirenz 600 MG Delayed Release Oral Tablet [Stocrin] Box of 30 by Axicorp | Marketed Product | Drug | RxNorm Extension |
| 36269549 | Ritonavir Oral Suspension [Norvir] | Branded Drug Form | Drug | RxNorm Extension |
| 43030473 | efavirenz 600 MG Oral Tablet Box of 30 by TEVA | Marketed Product | Drug | RxNorm Extension |
| 44041760 | Didanosine 4000 MG [Videx Pediatric] | Branded Drug Comp | Drug | RxNorm Extension |
| 1789455 | amprenavir 50 MG | Clinical Drug Comp | Drug | RxNorm |
| 42927755 | tenofovir disoproxil Oral Tablet [VIREHEPA] | Branded Drug Form | Drug | RxNorm Extension |
| 43169047 | darunavir 150 MG Delayed Release Oral Tablet [Prezista] | Branded Drug | Drug | RxNorm Extension |
| 36784665 | abacavir 600 MG / Lamivudine 300 MG Delayed Release Oral Tablet [Mylan-Abacavir/Lamivudine] | Branded Drug | Drug | RxNorm Extension |
| 44121505 | Nevirapine Oral Tablet [Apo-Nevirapine] | Branded Drug Form | Drug | RxNorm Extension |
| 1724912 | didanosine 150 MG Chewable Tablet [Videx] | Branded Drug | Drug | RxNorm |
| 43750550 | efavirenz 600 MG [Efavirenz Accord] | Branded Drug Comp | Drug | RxNorm Extension |
| 35605921 | emtricitabine 200 MG / rilpivirine 25 MG / tenofovir alafenamide 25 MG Oral Tablet | Clinical Drug | Drug | RxNorm |
| 36232127 | Selzentry Pill | Branded Dose Group | Drug | RxNorm |
| 21032078 | Saquinavir 500 MG Oral Tablet [Invirase] Box of 120 by Roche | Marketed Product | Drug | RxNorm Extension |
| 41069222 | Zidovudine 250 MG Oral Capsule [Retrovir] Box of 40 by Emra-Med | Marketed Product | Drug | RxNorm Extension |
| 21121802 | abacavir 600 MG / Lamivudine 300 MG Oral Tablet [Kivexa] Box of 30 | Branded Drug Box | Drug | RxNorm Extension |
| 21151560 | efavirenz 30 MG/ML [Sustiva] | Branded Drug Comp | Drug | RxNorm Extension |
| 2011838 | tenofovir disoproxil 300 MG Oral Tablet [TENOLID-F] by Samjin | Marketed Product | Drug | RxNorm Extension |
| 36213369 | saquinavir Oral Product | Clinical Dose Group | Drug | RxNorm |
| 36236164 | Agenerase Pill | Branded Dose Group | Drug | RxNorm |
| 43212957 | cobicistat 150 MG / elvitegravir 150 MG / emtricitabine 200 MG / tenofovir disoproxil 250 MG Delayed Release Oral Tablet [Stribild] Box of 30 | Branded Drug Box | Drug | RxNorm Extension |
| 43180134 | darunavir 150 MG Delayed Release Oral Tablet [Prezista] Box of 240 | Branded Drug Box | Drug | RxNorm Extension |
| 40987956 | Lamivudine / Zidovudine Delayed Release Oral Tablet [Lamizido] | Branded Drug Form | Drug | RxNorm Extension |
| 37592138 | Nevirapine Extended Release Oral Tablet [Nevirapin Hexal] | Branded Drug Form | Drug | RxNorm Extension |
| 21108650 | 180 ML efavirenz 30 MG/ML Oral Solution [Sustiva] by Bristol Myers Squibb | Marketed Product | Drug | RxNorm Extension |
| 44115232 | emtricitabine 200 MG / Rilpivirine 25 MG / tenofovir disoproxil 300 MG Oral Tablet [Complera] by Gilead | Marketed Product | Drug | RxNorm Extension |
| 46275632 | ombitasvir 12.5 MG / paritaprevir 75 MG / ritonavir 50 MG [Technivie] | Branded Drug Comp | Drug | RxNorm |
| 35858559 | stavudine 20 MG Oral Capsule [Shazhi] | Branded Drug | Drug | RxNorm Extension |
| 43280466 | 1 ML Didanosine 5 MG/ML Powder for Oral Solution [Videx] | Quant Branded Drug | Drug | RxNorm Extension |
| 43822634 | efavirenz 600 MG [Efavirenz Aurobindo] | Branded Drug Comp | Drug | RxNorm Extension |
| 41062398 | Lamivudine 150 MG Oral Tablet [Lamivudin Aurobindo] Box of 60 | Branded Drug Box | Drug | RxNorm Extension |
| 41149219 | darunavir 600 MG Oral Tablet Box of 30 | Clinical Drug Box | Drug | RxNorm Extension |
| 36504283 | Lamivudine 100 MG Delayed Release Oral Tablet [Zeffix] by Axicorp | Marketed Product | Drug | RxNorm Extension |
| 36243992 | Viread Oral Powder Product | Branded Dose Group | Drug | RxNorm |
| 43149975 | cobicistat 150 MG / elvitegravir 150 MG / emtricitabine 200 MG / tenofovir alafenamide 10 MG Delayed Release Oral Tablet [Genvoya] Box of 30 | Branded Drug Box | Drug | RxNorm Extension |
| 35768603 | Didanosine 250 MG Extended Release Oral Capsule [Videx EC] by Bristol Myers Squibb | Marketed Product | Drug | RxNorm Extension |
| 44168401 | raltegravir Oral Powder [Isentress] | Branded Drug Form | Drug | RxNorm Extension |
| 43189256 | Lamivudine 300 MG Delayed Release Oral Tablet | Clinical Drug | Drug | RxNorm Extension |
| 35860786 | lopinavir Oral Tablet | Clinical Drug Form | Drug | RxNorm Extension |
| 43022458 | darunavir 800 MG Delayed Release Oral Tablet Box of 30 by Sandoz | Marketed Product | Drug | RxNorm Extension |
| 43190967 | emtricitabine 200 MG / Rilpivirine 25 MG / tenofovir disoproxil 250 MG Delayed Release Oral Tablet Box of 30 | Clinical Drug Box | Drug | RxNorm Extension |
| 35860836 | ritonavir 100 MG Oral Tablet [Aizhiwei] | Branded Drug | Drug | RxNorm Extension |
| 43840730 | efavirenz Oral Tablet [EFAVIRENZ SANDOZ] | Branded Drug Form | Drug | RxNorm Extension |
| 21023725 | Lamivudine 150 MG Oral Tablet by A A H | Marketed Product | Drug | RxNorm Extension |
| 36504352 | darunavir 600 MG Oral Tablet by Betapharm | Marketed Product | Drug | RxNorm Extension |
| 43184867 | Zidovudine Delayed Release Oral Tablet [Retrovir] | Branded Drug Form | Drug | RxNorm Extension |
| 42927722 | tenofovir disoproxil 250 MG [TENFOVIR] | Branded Drug Comp | Drug | RxNorm Extension |
| 43588573 | Nevirapine 200 MG Oral Tablet [Nevirapin Hexal] by Hexal | Marketed Product | Drug | RxNorm Extension |
| 36214475 | atazanavir Oral Product | Clinical Dose Group | Drug | RxNorm |
| 35770020 | ombitasvir 12.5 MG / paritaprevir 75 MG / Ritonavir 50 MG Delayed Release Oral Tablet [Viekirax] by Abbvie | Marketed Product | Drug | RxNorm Extension |
| 44204751 | 4000 MG Didanosine 1 MG/MG Oral Solution [Videx] | Quant Branded Drug | Drug | RxNorm Extension |
| 43210854 | efavirenz 600 MG / emtricitabine 200 MG / tenofovir disoproxil 250 MG Delayed Release Oral Tablet [Atripla] Box of 30 | Branded Drug Box | Drug | RxNorm Extension |
| 21142013 | Didanosine 25 MG Chewable Tablet [Videx] Box of 60 | Branded Drug Box | Drug | RxNorm Extension |
| 43177135 | Didanosine 200 MG Extended Release Oral Tablet [Videx] | Branded Drug | Drug | RxNorm Extension |
| 40051162 | lamivudine / zidovudine Oral Tablet [Combivir] | Branded Drug Form | Drug | RxNorm |
| 36065920 | dolutegravir 50 MG / lamivudine 300 MG Oral Tablet [Dovato] by ViiV | Marketed Product | Drug | RxNorm Extension |
| 40143572 | raltegravir Oral Tablet [Isentress] | Branded Drug Form | Drug | RxNorm |
| 21053409 | efavirenz 200 MG Oral Capsule Box of 90 | Clinical Drug Box | Drug | RxNorm Extension |
| 21171237 | efavirenz 600 MG Oral Tablet Box of 30 | Clinical Drug Box | Drug | RxNorm Extension |
| 41312206 | Lamivudine 150 MG Oral Tablet [Lamivudin Teva] Box of 60 | Branded Drug Box | Drug | RxNorm Extension |
| 40741181 | dolutegravir 50 MG / Rilpivirine 25 MG Oral Tablet [Juluca] by ViiV | Marketed Product | Drug | RxNorm Extension |
| 41042366 | efavirenz 600 MG Oral Tablet [Stocrin] Box of 30 by Axicorp | Marketed Product | Drug | RxNorm Extension |
| 40720871 | dolutegravir 10 MG Oral Tablet [Tivicay] by ViiV | Marketed Product | Drug | RxNorm Extension |
| 19079686 | stavudine 40 MG Oral Capsule | Clinical Drug | Drug | RxNorm |
| 41430792 | 180 ML efavirenz 0.167 MG/ML Oral Solution | Quant Clinical Drug | Drug | RxNorm Extension |
| 43200065 | efavirenz 600 MG Delayed Release Oral Tablet [EFAVIRENZ MYLAN] | Branded Drug | Drug | RxNorm Extension |
| 36781988 | raltegravir 600 MG Delayed Release Oral Tablet | Clinical Drug | Drug | RxNorm Extension |
| 35862028 | zidovudine Topical Ointment | Clinical Drug Form | Drug | RxNorm Extension |
| 21151343 | Lamivudine 100 MG Oral Tablet Box of 28 by Alliance | Marketed Product | Drug | RxNorm Extension |
| 782652 | Nevirapine 400 MG Extended Release Oral Capsule [NEVIRAPINE SANDOZ] Box of 30 by Sandoz | Marketed Product | Drug | RxNorm Extension |
| 36062288 | abacavir 600 MG / lamivudine 300 MG Oral Tablet by Zentiva | Marketed Product | Drug | RxNorm Extension |
| 35860779 | lopinavir 200 MG Oral Tablet | Clinical Drug | Drug | RxNorm Extension |
| 36785879 | efavirenz 200 MG Delayed Release Oral Tablet [Stocrin] Box of 90 | Branded Drug Box | Drug | RxNorm Extension |
| 21063041 | Lamivudine 150 MG / Zidovudine 300 MG Oral Tablet [Combivir] by Mawdsley-Brooks | Marketed Product | Drug | RxNorm Extension |
| 40730830 | Lamivudine 100 MG Oral Tablet by Mylan | Marketed Product | Drug | RxNorm Extension |
| 40978424 | maraviroc 300 MG Oral Tablet [Celsentri] Box of 60 by Haematogmbh | Marketed Product | Drug | RxNorm Extension |
| 41378269 | 20 ML Zidovudine 10 MG/ML Injection [Retrovir] Box of 10 | Quant Branded Box | Drug | RxNorm Extension |
| 41112903 | Lamivudine Delayed Release Oral Tablet [Lamivudin Aurobindo] | Branded Drug Form | Drug | RxNorm Extension |
| 41290031 | Nevirapine 200 MG Oral Tablet [Viramune] Box of 60 by MZG | Marketed Product | Drug | RxNorm Extension |
| 36062161 | tenofovir disoproxil 163 MG Oral Tablet [Viread] | Branded Drug | Drug | RxNorm Extension |
| 2052751 | tenofovir disoproxil 300 MG Oral Tablet [TENOVA] by Korea Kolmar | Marketed Product | Drug | RxNorm Extension |
| 40854226 | Lamivudine 300 MG Oral Tablet [Epivir] Box of 30 by Medicopharm | Marketed Product | Drug | RxNorm Extension |
| 35605556 | cobicistat 150 MG / elvitegravir 150 MG / emtricitabine 200 MG / tenofovir alafenamide 10 MG Oral Tablet [Genvoya] | Branded Drug | Drug | RxNorm |
| 21071497 | Indinavir 400 MG Oral Capsule [Crixivan] Box of 90 by Merck | Marketed Product | Drug | RxNorm Extension |
| 35156246 | tenofovir disoproxil 300 MG [Tenozet] | Branded Drug Comp | Drug | RxNorm Extension |
| 41096442 | efavirenz 600 MG Delayed Release Oral Tablet [Efavirenz Accord] | Branded Drug | Drug | RxNorm Extension |
| 40947773 | Lamivudine 100 MG Delayed Release Oral Tablet [Zeffix] Box of 28 by Eurim-Pharm | Marketed Product | Drug | RxNorm Extension |
| 40008593 | amprenavir Oral Capsule [Agenerase] | Branded Drug Form | Drug | RxNorm |
| 43172104 | cobicistat 150 MG / elvitegravir 150 MG / emtricitabine 200 MG / tenofovir alafenamide 10 MG Delayed Release Oral Tablet Box of 30 | Clinical Drug Box | Drug | RxNorm Extension |
| 42927718 | tenofovir disoproxil 250 MG Oral Tablet [VIRIP] | Branded Drug | Drug | RxNorm Extension |
| 587311 | 10 ML Zidovudine 20 MG/ML Injection Box of 10 | Quant Clinical Box | Drug | RxNorm Extension |
| 2053051 | lamivudine 100 MG Oral Tablet [LAFIX] by Sinil | Marketed Product | Drug | RxNorm Extension |
| 45774770 | cobicistat 150 MG [Tybost] | Branded Drug Comp | Drug | RxNorm |
| 41169220 | Atazanavir 200 MG Oral Capsule [Reyataz] Box of 60 by Mevita | Marketed Product | Drug | RxNorm Extension |
| 43158206 | tenofovir disoproxil 0.033 MG/MG [Viread] | Branded Drug Comp | Drug | RxNorm Extension |
| 36268199 | efavirenz 600 MG Oral Tablet [EFAVIRENZ MYLAN] Box of 30 | Branded Drug Box | Drug | RxNorm Extension |
| 43138383 | raltegravir 400 MG Delayed Release Oral Tablet [Isentress] Box of 60 by Merck | Marketed Product | Drug | RxNorm Extension |
| 36891509 | Didanosine Oral Powder | Clinical Drug Form | Drug | RxNorm Extension |
| 1736925 | fosamprenavir 50 MG/ML Oral Suspension [Lexiva] | Branded Drug | Drug | RxNorm |
| 41103893 | Lamivudine 150 MG / Zidovudine 300 MG Delayed Release Oral Tablet [Combivir] Box of 60 by Cc | Marketed Product | Drug | RxNorm Extension |
| 40883376 | Ritonavir 100 MG Oral Tablet [Norvir] Box of 30 by Eurim-Pharm | Marketed Product | Drug | RxNorm Extension |
| 35860455 | ritonavir 80 MG/ML [Maikexin] | Branded Drug Comp | Drug | RxNorm Extension |
| 41148917 | efavirenz 600 MG Oral Tablet Box of 90 | Clinical Drug Box | Drug | RxNorm Extension |
| 41105553 | darunavir 300 MG Oral Tablet [Prezista] Box of 120 by Cc | Marketed Product | Drug | RxNorm Extension |
| 40142304 | maraviroc Oral Tablet | Clinical Drug Form | Drug | RxNorm |
| 41064865 | emtricitabine 200 MG / tenofovir disoproxil 300 MG Oral Tablet [Truvada] Box of 30 | Branded Drug Box | Drug | RxNorm Extension |
| 40882848 | tenofovir disoproxil 250 MG Oral Tablet [Viread] Box of 30 by Emra-Med | Marketed Product | Drug | RxNorm Extension |
| 21171248 | abacavir 300 MG Oral Tablet Box of 60 | Clinical Drug Box | Drug | RxNorm Extension |
| 1592436 | ritonavir Oral Powder [Norvir] | Branded Drug Form | Drug | RxNorm |
| 36788477 | efavirenz 600 MG / emtricitabine 200 MG / tenofovir disoproxil 250 MG [Efavirenz / Emtricitabine / Tenofovir disoproxil Mylan] | Branded Drug Comp | Drug | RxNorm Extension |
| 41229015 | Lamivudine 100 MG Oral Tablet [Zeffix] Box of 84 by Axicorp | Marketed Product | Drug | RxNorm Extension |
| 43037156 | tenofovir disoproxil 123 MG Delayed Release Oral Tablet Box of 30 by Arrow | Marketed Product | Drug | RxNorm Extension |
| 1710656 | zidovudine 10 MG/ML | Clinical Drug Comp | Drug | RxNorm |
| 42874784 | darunavir 800 MG Delayed Release Oral Tablet Box of 90 by Mylan | Marketed Product | Drug | RxNorm Extension |
| 21043413 | Stavudine 30 MG Oral Capsule Box of 56 | Clinical Drug Box | Drug | RxNorm Extension |
| 41260968 | emtricitabine 200 MG / tenofovir disoproxil 300 MG Oral Tablet [Truvada] Box of 30 by Canoma | Marketed Product | Drug | RxNorm Extension |
| 40861829 | Didanosine Oral Capsule | Clinical Drug Form | Drug | RxNorm Extension |
| 40896365 | tenofovir disoproxil 200 MG Oral Tablet Box of 30 | Clinical Drug Box | Drug | RxNorm Extension |
| 36218352 | lamivudine / stavudine Pill | Clinical Dose Group | Drug | RxNorm |
| 43733306 | tenofovir disoproxil Oral Tablet [Tenofovirdisoproxil-Ratiopharm] | Branded Drug Form | Drug | RxNorm Extension |
| 37594088 | 1490 MG Atazanavir 0.0333 MG/MG Oral Granules | Quant Clinical Drug | Drug | RxNorm Extension |
| 41075644 | abacavir 300 MG Delayed Release Oral Tablet [Ziagen] Box of 60 by ViiV | Marketed Product | Drug | RxNorm Extension |
| 2052788 | tenofovir disoproxil 250 MG [TENOVIER] | Branded Drug Comp | Drug | RxNorm Extension |
| 782832 | emtricitabine 200 MG / Tenofovir 245 MG Oral Tablet [Emtricitabine / Tenofovir disoproxil Krka] Box of 30 | Branded Drug Box | Drug | RxNorm Extension |
| 43714567 | Nevirapine Oral Tablet [Nevirapin Aurobindo] | Branded Drug Form | Drug | RxNorm Extension |
| 36779000 | 60 ML lopinavir 80 MG/ML / Ritonavir 20 MG/ML Oral Solution [Kaletra] Box of 2 | Quant Branded Box | Drug | RxNorm Extension |
| 21056113 | fosamprenavir 150 MG | Clinical Drug Comp | Drug | RxNorm Extension |
| 44030939 | maraviroc Oral Tablet [Celsentr] | Branded Drug Form | Drug | RxNorm Extension |
| 36230119 | Prezista Pill | Branded Dose Group | Drug | RxNorm |
| 21053663 | Didanosine 400 MG Delayed Release Oral Capsule [Videx EC] by Bristol Myers Squibb | Marketed Product | Drug | RxNorm Extension |
| 19124342 | emtricitabine 200 MG Oral Tablet | Clinical Drug | Drug | RxNorm |
| 44112432 | dasabuvir 250 MG / ombitasvir 12.5 MG / paritaprevir 75 MG / Ritonavir 50 MG Oral Tablet [Holkira] | Branded Drug | Drug | RxNorm Extension |
| 41260970 | emtricitabine 200 MG / Rilpivirine 25 MG / tenofovir disoproxil 300 MG Oral Tablet [Eviplera] Box of 30 by European | Marketed Product | Drug | RxNorm Extension |
| 40982471 | Nelfinavir 0.0585 MG/MG | Clinical Drug Comp | Drug | RxNorm Extension |
| 36061959 | atazanavir 300 MG Oral Capsule by A A H | Marketed Product | Drug | RxNorm Extension |
| 40981883 | abacavir 300 MG Oral Tablet [Ziagen] Box of 60 by Medicopharm | Marketed Product | Drug | RxNorm Extension |
| 21141530 | Lamivudine 100 MG Oral Tablet [Zeffix] by Lexon | Marketed Product | Drug | RxNorm Extension |
| 40730840 | Lamivudine 300 MG Oral Tablet Box of 30 by Accord | Marketed Product | Drug | RxNorm Extension |
| 21083114 | Didanosine 25 MG Chewable Tablet [Videx] by Bristol Myers Squibb | Marketed Product | Drug | RxNorm Extension |
| 41250075 | Lamivudine 300 MG Delayed Release Oral Tablet [Lamivudin Aurobindo] Box of 60 | Branded Drug Box | Drug | RxNorm Extension |
| 41435683 | 226 ML fosamprenavir 0.222 MG/ML Oral Solution Box of 1 | Quant Clinical Box | Drug | RxNorm Extension |
| 41060965 | Nevirapine 10 MG/ML Oral Solution [Viramune] Box of 1 | Branded Drug Box | Drug | RxNorm Extension |
| 1704191 | lamivudine 5 MG/ML Oral Solution [Epivir HBV] | Branded Drug | Drug | RxNorm |
| 36241911 | Videx EC Pill | Branded Dose Group | Drug | RxNorm |
| 40914147 | Saquinavir 500 MG Oral Tablet [Invirase] Box of 120 by Kohlpharma | Marketed Product | Drug | RxNorm Extension |
| 1746248 | saquinavir 200 MG | Clinical Drug Comp | Drug | RxNorm |
| 41304478 | Didanosine 400 MG Delayed Release Oral Capsule Box of 60 | Clinical Drug Box | Drug | RxNorm Extension |
| 41021878 | Nevirapine 400 MG Oral Tablet Box of 60 | Clinical Drug Box | Drug | RxNorm Extension |
| 21134573 | raltegravir 400 MG Oral Tablet [Isentress] Box of 60 by Merck | Marketed Product | Drug | RxNorm Extension |
| 964012 | bictegravir / emtricitabine / tenofovir alafenamide Oral Product | Clinical Dose Group | Drug | RxNorm |
| 19123900 | nelfinavir 200 MG [Viracept] | Branded Drug Comp | Drug | RxNorm |
| 21151342 | Lamivudine 100 MG Oral Tablet by A A H | Marketed Product | Drug | RxNorm Extension |
| 41435283 | 200 ML Stavudine 1 MG/ML Oral Solution [Zerit] Box of 1 by Gerke | Marketed Product | Drug | RxNorm Extension |
| 36403691 | fosamprenavir 150 MG Injectable Solution [Ivemend] Box of 1 | Branded Drug Box | Drug | RxNorm Extension |
| 35858562 | stavudine 20 MG [Sairuite] | Branded Drug Comp | Drug | RxNorm Extension |
| 19103096 | stavudine 100 MG | Clinical Drug Comp | Drug | RxNorm |
| 1724918 | didanosine 125 MG Delayed Release Oral Capsule | Clinical Drug | Drug | RxNorm |
| 41312205 | Lamivudine 300 MG Delayed Release Oral Tablet [Lamivudin Teva] Box of 60 | Branded Drug Box | Drug | RxNorm Extension |
| 1748960 | ritonavir 33.3 MG | Clinical Drug Comp | Drug | RxNorm |
| 43840729 | efavirenz Oral Tablet [Efavirenz Aurobindo] | Branded Drug Form | Drug | RxNorm Extension |
| 36223705 | stavudine Pill | Clinical Dose Group | Drug | RxNorm |
| 1738204 | lopinavir 100 MG | Clinical Drug Comp | Drug | RxNorm |
| 21073128 | efavirenz 600 MG Oral Tablet Box of 30 by A A H | Marketed Product | Drug | RxNorm Extension |
| 21023727 | Nevirapine 200 MG Oral Tablet by A A H | Marketed Product | Drug | RxNorm Extension |
| 36778174 | 200 ML darunavir 100 MG/ML Oral Suspension Box of 1 | Quant Clinical Box | Drug | RxNorm Extension |
| 21149422 | dolutegravir 50 MG Oral Tablet Box of 30 | Clinical Drug Box | Drug | RxNorm Extension |
| 35762188 | cobicistat Delayed Release Oral Tablet [Tybost] | Branded Drug Form | Drug | RxNorm Extension |
| 44067260 | Nevirapine 200 MG [Auro-Nevirapine] | Branded Drug Comp | Drug | RxNorm Extension |
| 43028260 | dolutegravir 50 MG / Rilpivirine 25 MG Delayed Release Oral Tablet Box of 30 | Clinical Drug Box | Drug | RxNorm Extension |
| 42705551 | darunavir 100 MG/ML | Clinical Drug Comp | Drug | RxNorm |
| 21092420 | Nevirapine 50 MG Extended Release Oral Tablet Box of 180 | Clinical Drug Box | Drug | RxNorm Extension |
| 40979666 | etravirine 100 MG Oral Tablet [Intelence] Box of 120 by Cc | Marketed Product | Drug | RxNorm Extension |
| 41221488 | emtricitabine 200 MG / Rilpivirine 25 MG / tenofovir alafenamide 25 MG Oral Tablet [Odefsey] Box of 90 | Branded Drug Box | Drug | RxNorm Extension |
| 40720772 | raltegravir 600 MG Oral Tablet Box of 60 | Clinical Drug Box | Drug | RxNorm Extension |
| 1736997 | abacavir 300 MG | Clinical Drug Comp | Drug | RxNorm |
| 35859814 | nevirapine 200 MG [Aitai] | Branded Drug Comp | Drug | RxNorm Extension |
| 40032845 | didanosine Oral Suspension | Clinical Drug Form | Drug | RxNorm |
| 41135305 | Lamivudine 100 MG Oral Tablet [Lamivudin Teva] Box of 84 by TEVA | Marketed Product | Drug | RxNorm Extension |
| 36061906 | lopinavir 200 MG / ritonavir 50 MG Delayed Release Oral Tablet Box of 30 | Clinical Drug Box | Drug | RxNorm Extension |
| 21124559 | darunavir 300 MG Oral Tablet [Prezista] Box of 120 by Janssen | Marketed Product | Drug | RxNorm Extension |
| 21033961 | Didanosine 200 MG Chewable Tablet Box of 60 | Clinical Drug Box | Drug | RxNorm Extension |
| 35862596 | emtricitabine 200 MG [Shufatai] | Branded Drug Comp | Drug | RxNorm Extension |
| 44111895 | Lamivudine 150 MG / Zidovudine 300 MG Oral Tablet [Apo-Lamivudine-Zidovudine] | Branded Drug | Drug | RxNorm Extension |
| 1703069 | emtricitabine | Ingredient | Drug | RxNorm |
| 40746918 | emtricitabine 200 MG / tenofovir disoproxil 250 MG Oral Tablet by TEVA | Marketed Product | Drug | RxNorm Extension |
| 40945267 | Stavudine 40 MG Oral Capsule [Zerit] Box of 56 by Mtk | Marketed Product | Drug | RxNorm Extension |
| 40971261 | emtricitabine 200 MG / tenofovir alafenamide 25 MG Oral Tablet [Descovy] Box of 30 | Branded Drug Box | Drug | RxNorm Extension |
| 40724600 | Ritonavir 100 MG Oral Tablet by Accord | Marketed Product | Drug | RxNorm Extension |
| 21141531 | Lamivudine 100 MG Oral Tablet [Zeffix] Box of 28 by Waymade | Marketed Product | Drug | RxNorm Extension |
| 43145155 | Lamivudine / Zidovudine Delayed Release Oral Tablet [LAMIVUDINE/ZIDOVUDINE SANDOZ] | Branded Drug Form | Drug | RxNorm Extension |
| 782841 | efavirenz 600 MG / emtricitabine 200 MG / tenofovir disoproxil 250 MG Oral Tablet Box of 90 | Clinical Drug Box | Drug | RxNorm Extension |
| 21033713 | efavirenz 600 MG / emtricitabine 200 MG / tenofovir disoproxil 250 MG Oral Tablet Box of 30 | Clinical Drug Box | Drug | RxNorm Extension |
| 21141535 | Nevirapine 200 MG Oral Tablet by Sandoz | Marketed Product | Drug | RxNorm Extension |
| 21161281 | Lamivudine 100 MG Oral Tablet Box of 28 by Sigma-Tau | Marketed Product | Drug | RxNorm Extension |
| 40720874 | dolutegravir 25 MG Oral Tablet [Tivicay] Box of 30 | Branded Drug Box | Drug | RxNorm Extension |
| 43822108 | Lamivudine 300 MG [Lamivudin Hexal] | Branded Drug Comp | Drug | RxNorm Extension |
| 35860797 | efavirenz Oral Tablet [Shiduoning] | Branded Drug Form | Drug | RxNorm Extension |
| 2052779 | tenofovir disoproxil 250 MG Oral Tablet [TENOVI] | Branded Drug | Drug | RxNorm Extension |
| 44182583 | fosamprenavir 700 MG Delayed Release Oral Tablet [Telzir] Box of 60 by Kohlpharma | Marketed Product | Drug | RxNorm Extension |
| 41072412 | Lamivudine 100 MG Oral Tablet [Zeffix] Box of 84 by Cc | Marketed Product | Drug | RxNorm Extension |
| 36061610 | darunavir 400 MG Oral Tablet Box of 60 by Zentiva | Marketed Product | Drug | RxNorm Extension |
| 42481363 | Nelfinavir 0.05 MG/MG Injectable Solution | Clinical Drug | Drug | RxNorm Extension |
| 2053053 | lamivudine 100 MG Oral Tablet [LAMIDINE] by BC World | Marketed Product | Drug | RxNorm Extension |
| 44171512 | Didanosine 400 MG Extended Release Oral Capsule [Videx] Box of 60 by Axicorp | Marketed Product | Drug | RxNorm Extension |
| 43156110 | efavirenz 600 MG Delayed Release Oral Tablet [EFAVIRENZ MYLAN] Box of 30 | Branded Drug Box | Drug | RxNorm Extension |
| 21139474 | cobicistat 150 MG / darunavir 800 MG Oral Tablet [Rezolsta] Box of 30 by Janssen | Marketed Product | Drug | RxNorm Extension |
| 40720632 | tenofovir disoproxil 250 MG Oral Tablet Box of 30 by Dr Reddy's | Marketed Product | Drug | RxNorm Extension |
| 44093004 | maraviroc 150 MG [Celsentr] | Branded Drug Comp | Drug | RxNorm Extension |
| 21043416 | Lamivudine 100 MG Oral Tablet Box of 28 | Clinical Drug Box | Drug | RxNorm Extension |
| 41011687 | darunavir 400 MG Oral Tablet [Prezista] Box of 60 by Gerke | Marketed Product | Drug | RxNorm Extension |
| 41435672 | 226 ML fosamprenavir 0.222 MG/ML Oral Solution [Telzir] | Quant Branded Drug | Drug | RxNorm Extension |
| 40852101 | Ritonavir 100 MG Oral Tablet [Norvir] Box of 90 by Kohlpharma | Marketed Product | Drug | RxNorm Extension |
| 21085487 | darunavir 300 MG Oral Tablet [Prezista] by Janssen | Marketed Product | Drug | RxNorm Extension |
| 43752639 | 2000 MG Didanosine 1 MG/MG Oral Powder [Videx] | Quant Branded Drug | Drug | RxNorm Extension |
| 2053068 | lamivudine Oral Tablet [HEPAVUDIN] | Branded Drug Form | Drug | RxNorm Extension |
| 43032546 | lopinavir 100 MG / Ritonavir 25 MG Delayed Release Oral Tablet Box of 60 by Mylan | Marketed Product | Drug | RxNorm Extension |
| 43176513 | 200 ML Stavudine 1 MG/ML Powder for Oral Solution [Zerit] | Quant Branded Drug | Drug | RxNorm Extension |
| 36241914 | Videx Pill | Branded Dose Group | Drug | RxNorm |
| 35751886 | Didanosine 200 MG Oral Tablet [Videx] Box of 60 by Bristol Myers Squibb | Marketed Product | Drug | RxNorm Extension |
| 37593206 | Atazanavir 0.0333 MG/MG [Reyataz] | Branded Drug Comp | Drug | RxNorm Extension |
| 40080336 | ritonavir Oral Capsule [Norvir] | Branded Drug Form | Drug | RxNorm |
| 43257628 | Stavudine 40 MG Oral Capsule [Zerit] Box of 60 | Branded Drug Box | Drug | RxNorm Extension |
| 44174956 | Lamivudine 150 MG Delayed Release Oral Tablet [Epivir] Box of 60 by Eurim-Pharm | Marketed Product | Drug | RxNorm Extension |
| 41135085 | lopinavir 133 MG / Ritonavir 33.3 MG Oral Capsule [Kaletra] Box of 180 by Eurim-Pharm | Marketed Product | Drug | RxNorm Extension |
| 21102495 | efavirenz 100 MG Oral Capsule [Sustiva] by Bristol Myers Squibb | Marketed Product | Drug | RxNorm Extension |
| 43167285 | abacavir / Lamivudine Delayed Release Oral Tablet [Kivexa] | Branded Drug Form | Drug | RxNorm Extension |
| 41294031 | abacavir 703 MG / Lamivudine 300 MG Oral Tablet [Kivexa] Box of 30 by Aca Mueller | Marketed Product | Drug | RxNorm Extension |
| 41462731 | 2000 MG Didanosine 1 MG/MG Oral Suspension | Quant Clinical Drug | Drug | RxNorm Extension |
| 35862466 | lamivudine Oral Tablet [Hepuding] | Branded Drug Form | Drug | RxNorm Extension |
| 43166154 | Didanosine 250 MG Extended Release Oral Tablet [Videx] | Branded Drug | Drug | RxNorm Extension |
| 41435822 | 240 ML Nevirapine 0.0417 MG/ML Oral Solution [Viramune] | Quant Branded Drug | Drug | RxNorm Extension |
| 43178108 | efavirenz 600 MG / emtricitabine 200 MG / tenofovir disoproxil 250 MG Delayed Release Oral Tablet [Atripla] Box of 30 by Bristol Myers Squibb | Marketed Product | Drug | RxNorm Extension |
| 21124558 | darunavir 800 MG Oral Tablet [Prezista] by Janssen | Marketed Product | Drug | RxNorm Extension |
| 40058823 | fosamprenavir Oral Tablet [Lexiva] | Branded Drug Form | Drug | RxNorm |
| 43624656 | Atazanavir 228 MG Oral Capsule [Atazanavir-Ratiopharm] by Ratiopharm | Marketed Product | Drug | RxNorm Extension |
| 41435313 | 200 ML Zidovudine 0.25 MG/ML Oral Solution Box of 1 | Quant Clinical Box | Drug | RxNorm Extension |
| 44043480 | Zidovudine Oral Capsule [Novo-Azt] | Branded Drug Form | Drug | RxNorm Extension |
| 43785855 | Didanosine 1 MG/MG | Clinical Drug Comp | Drug | RxNorm Extension |
| 41242524 | Didanosine 200 MG Delayed Release Oral Capsule Box of 60 | Clinical Drug Box | Drug | RxNorm Extension |
| 36061607 | darunavir 600 MG Oral Tablet by Accord | Marketed Product | Drug | RxNorm Extension |
| 41280883 | Lamivudine 100 MG Delayed Release Oral Tablet [Lamivir] Box of 84 | Branded Drug Box | Drug | RxNorm Extension |
| 45892556 | ombitasvir / paritaprevir / ritonavir Oral Tablet | Clinical Drug Form | Drug | RxNorm |
| 19103099 | stavudine 75 MG | Clinical Drug Comp | Drug | RxNorm |
| 36061606 | darunavir 600 MG Oral Tablet by Mylan | Marketed Product | Drug | RxNorm Extension |
| 21161291 | Nevirapine 200 MG Oral Tablet [Viramune] Box of 60 | Branded Drug Box | Drug | RxNorm Extension |
| 43026164 | emtricitabine 200 MG / tenofovir disoproxil 250 MG Delayed Release Oral Tablet Box of 30 by Biogaran | Marketed Product | Drug | RxNorm Extension |
| 41015711 | Lamivudine 150 MG / Zidovudine 300 MG [Combivir Cmj] | Branded Drug Comp | Drug | RxNorm Extension |
| 36893408 | Didanosine 250 MG [Videx] | Branded Drug Comp | Drug | RxNorm Extension |
| 21114741 | Atazanavir 150 MG Oral Capsule [Reyataz] by Bristol Myers Squibb | Marketed Product | Drug | RxNorm Extension |
| 41101768 | Ritonavir 100 MG Delayed Release Oral Tablet [Norvir] Box of 90 by Cambridge | Marketed Product | Drug | RxNorm Extension |
| 1738135 | efavirenz | Ingredient | Drug | RxNorm |
| 43138927 | Nelfinavir 250 MG Delayed Release Oral Tablet [Viracept] Box of 300 by Roche | Marketed Product | Drug | RxNorm Extension |
| 35862770 | lamivudine Oral Tablet [Yipingwei] | Branded Drug Form | Drug | RxNorm Extension |
| 41072414 | Lamivudine 150 MG Oral Tablet [Epivir] Box of 60 by Axicorp | Marketed Product | Drug | RxNorm Extension |
| 41258929 | Nevirapine 400 MG Oral Tablet [Viramune] Box of 90 by Orifarm Leverkus | Marketed Product | Drug | RxNorm Extension |
| 43193907 | Nelfinavir Delayed Release Oral Tablet [Viracept] | Branded Drug Form | Drug | RxNorm Extension |
| 40736229 | efavirenz 600 MG Oral Tablet Box of 30 by Milpharm | Marketed Product | Drug | RxNorm Extension |
| 19081174 | didanosine 25 MG | Clinical Drug Comp | Drug | RxNorm |
| 43023782 | Nevirapine 400 MG Oral Tablet Box of 30 by TEVA | Marketed Product | Drug | RxNorm Extension |
| 40753352 | Nevirapine 400 MG Extended Release Oral Tablet Box of 30 by Mylan | Marketed Product | Drug | RxNorm Extension |
| 41315123 | Didanosine 400 MG Extended Release Oral Capsule [Videx] Box of 60 | Branded Drug Box | Drug | RxNorm Extension |
| 43134208 | abacavir 600 MG / Lamivudine 300 MG Delayed Release Oral Tablet [Kivexa] Box of 30 by ViiV | Marketed Product | Drug | RxNorm Extension |
| 44216221 | 144000 MG Nelfinavir 0.0585 MG/MG Oral Powder [Viracept] Box of 1 | Quant Branded Box | Drug | RxNorm Extension |
| 36238741 | Retrovir Oral Product | Branded Dose Group | Drug | RxNorm |
| 21051153 | cobicistat 150 MG Oral Tablet [Tybost] by Gilead | Marketed Product | Drug | RxNorm Extension |
| 44174954 | Lamivudine 150 MG Oral Tablet [LAMIVUDINE MYLAN] Box of 60 by Mylan | Marketed Product | Drug | RxNorm Extension |
| 35859824 | nevirapine Oral Capsule [Aiweining] | Branded Drug Form | Drug | RxNorm Extension |
| 36275802 | etravirine 25 MG Disintegrating Oral Tablet [Intelence] | Branded Drug | Drug | RxNorm Extension |
| 44163693 | Lamivudine 150 MG Delayed Release Oral Tablet [Epivir] Box of 60 by Aca Mueller | Marketed Product | Drug | RxNorm Extension |
| 44090903 | abacavir 300 MG Oral Tablet [Apo-Abacavir] by Apotex | Marketed Product | Drug | RxNorm Extension |
| 44176187 | Nelfinavir 0.0585 MG/MG Oral Powder Box of 1 | Clinical Drug Box | Drug | RxNorm Extension |
| 44041366 | Lamivudine 100 MG [Apo-Lamivudine] | Branded Drug Comp | Drug | RxNorm Extension |
| 44170738 | raltegravir 400 MG Oral Tablet [Isentress] Box of 180 by Cc | Marketed Product | Drug | RxNorm Extension |
| 40058382 | efavirenz Oral Capsule [Sustiva] | Branded Drug Form | Drug | RxNorm |
| 41288216 | tenofovir disoproxil 250 MG Delayed Release Oral Tablet [Viread] Box of 30 by Canoma | Marketed Product | Drug | RxNorm Extension |
| 43143989 | Didanosine 125 MG Extended Release Oral Tablet [Videx] Box of 30 by Bristol Myers Squibb | Marketed Product | Drug | RxNorm Extension |
| 35773212 | abacavir 600 MG / Lamivudine 300 MG Delayed Release Oral Tablet [Kivexa] by ViiV | Marketed Product | Drug | RxNorm Extension |
| 43166157 | Didanosine 125 MG Extended Release Oral Tablet Box of 30 | Clinical Drug Box | Drug | RxNorm Extension |
| 43155132 | Didanosine 200 MG Extended Release Oral Tablet Box of 30 | Clinical Drug Box | Drug | RxNorm Extension |
| 35862008 | zidovudine 100 MG Oral Capsule [Aijian] | Branded Drug | Drug | RxNorm Extension |
| 43026145 | bictegravir 50 MG / emtricitabine 200 MG / tenofovir alafenamide 25 MG Delayed Release Oral Tablet [Biktarvy] Box of 30 | Branded Drug Box | Drug | RxNorm Extension |
| 36884120 | darunavir 100 MG/ML Oral Solution [Prezista] | Branded Drug | Drug | RxNorm Extension |
| 41074119 | darunavir 800 MG Oral Tablet [Prezista] Box of 90 by Emra-Med | Marketed Product | Drug | RxNorm Extension |
| 42874788 | darunavir 600 MG Oral Tablet Box of 180 | Clinical Drug Box | Drug | RxNorm Extension |
| 19131469 | tipranavir 100 MG/ML Oral Solution | Clinical Drug | Drug | RxNorm |
| 41123757 | Nevirapine 200 MG Oral Tablet [Nevirapin Hormosan] | Branded Drug | Drug | RxNorm Extension |
| 41435652 | 226 ML fosamprenavir 50 MG/ML Oral Solution | Quant Clinical Drug | Drug | RxNorm Extension |
| 36412245 | 450 ML Ritonavir 80 MG/ML Oral Solution [Norvir] by Abbvie | Marketed Product | Drug | RxNorm Extension |
| 41134413 | Nevirapine 400 MG Oral Tablet [Viramune] Box of 90 by Gerke | Marketed Product | Drug | RxNorm Extension |
| 41200616 | Atazanavir 300 MG Oral Capsule [Reyataz] Box of 30 by ADL | Marketed Product | Drug | RxNorm Extension |
| 36062165 | tenofovir disoproxil 163 MG | Clinical Drug Comp | Drug | RxNorm Extension |
| 36062170 | tenofovir disoproxil 250 MG Delayed Release Oral Tablet Box of 30 by Lupin | Marketed Product | Drug | RxNorm Extension |
| 41117412 | enfuvirtide 90 MG/ML Injectable Solution | Clinical Drug | Drug | RxNorm Extension |
| 41027628 | Ritonavir 100 MG Oral Solution [Norvir] | Branded Drug | Drug | RxNorm Extension |
| 41011257 | emtricitabine 200 MG / Rilpivirine 25 MG / tenofovir disoproxil 300 MG Oral Tablet [Eviplera] Box of 90 by European | Marketed Product | Drug | RxNorm Extension |
| 43155133 | Didanosine 200 MG Extended Release Oral Tablet [Videx] Box of 30 | Branded Drug Box | Drug | RxNorm Extension |
| 43149848 | Nelfinavir Delayed Release Oral Tablet | Clinical Drug Form | Drug | RxNorm Extension |
| 21072945 | Lamivudine 100 MG Oral Tablet [Zeffix] by Waymade | Marketed Product | Drug | RxNorm Extension |
| 44036777 | Nevirapine 200 MG Oral Tablet [Auro-Nevirapine] by Aurobindo | Marketed Product | Drug | RxNorm Extension |
| 43199153 | Didanosine 200 MG Extended Release Oral Tablet [Videx] Box of 30 by Bristol Myers Squibb | Marketed Product | Drug | RxNorm Extension |
| 41152923 | Saquinavir 500 MG Oral Tablet [Saquinavir Hexal] | Branded Drug | Drug | RxNorm Extension |
| 43136094 | tenofovir disoproxil 123 MG Delayed Release Oral Tablet [Viread] Box of 30 | Branded Drug Box | Drug | RxNorm Extension |
| 36783255 | Ritonavir 100 MG Delayed Release Oral Tablet [Norvir] Box of 90 by Abbvie | Marketed Product | Drug | RxNorm Extension |
| 35764664 | Lamivudine 100 MG Delayed Release Oral Tablet [Zeffix] Box of 28 by Lexon | Marketed Product | Drug | RxNorm Extension |
| 19129166 | nevirapine 100 MG | Clinical Drug Comp | Drug | RxNorm |
| 36897543 | {112 (dasabuvir 250 MG Oral Tablet) / 112 (ombitasvir 12.5 MG / paritaprevir 75 MG / Ritonavir 50 MG Oral Tablet) / 168 (Ribavirin 200 MG Oral Tablet) } Pack box of 1 | Clinical Pack Box | Drug | RxNorm Extension |
| 40730823 | abacavir 600 MG / Lamivudine 300 MG Oral Tablet by Mylan | Marketed Product | Drug | RxNorm Extension |
| 21159878 | Saquinavir 200 MG Oral Capsule [Invirase] Box of 270 | Branded Drug Box | Drug | RxNorm Extension |
| 44095150 | Zidovudine Oral Capsule [Apo-Zidovudine] | Branded Drug Form | Drug | RxNorm Extension |
| 44093106 | Lamivudine 150 MG / Zidovudine 300 MG [Auro-Lamivudine/Zidovudine] | Branded Drug Comp | Drug | RxNorm Extension |
| 36233245 | Intelence Pill | Branded Dose Group | Drug | RxNorm |
| 44163224 | raltegravir 400 MG Delayed Release Oral Tablet [Isentress] Box of 180 by Paranova Pack | Marketed Product | Drug | RxNorm Extension |
| 41217621 | Nevirapine 200 MG Oral Tablet [Nevirapin Ratiopharm] | Branded Drug | Drug | RxNorm Extension |
| 44075984 | Lamivudine 10 MG/ML Oral Solution by ViiV | Marketed Product | Drug | RxNorm Extension |
| 43023784 | Nevirapine 400 MG Oral Tablet Box of 30 by Cristers | Marketed Product | Drug | RxNorm Extension |
| 41029809 | Nelfinavir 250 MG Delayed Release Oral Tablet [Viracept] Box of 270 | Branded Drug Box | Drug | RxNorm Extension |
| 1704244 | lamivudine 150 MG / stavudine 30 MG Oral Tablet | Clinical Drug | Drug | RxNorm |
| 43022329 | darunavir 800 MG Oral Tablet Box of 30 by Eurogenerics | Marketed Product | Drug | RxNorm Extension |
| 35745445 | Didanosine Extended Release Oral Capsule [Videx EC] | Branded Drug Form | Drug | RxNorm Extension |
| 41096917 | Didanosine 200 MG Oral Capsule [Videx] Box of 60 | Branded Drug Box | Drug | RxNorm Extension |
| 35788655 | 226 ML fosamprenavir 50 MG/ML Oral Solution [Telzir] by ViiV | Marketed Product | Drug | RxNorm Extension |
| 36229805 | Norvir Oral Product | Branded Dose Group | Drug | RxNorm |
| 35862184 | didanosine 25 MG [Hate] | Branded Drug Comp | Drug | RxNorm Extension |
| 36058615 | doravirine 100 MG Oral Tablet [Pifeltro] by Merck | Marketed Product | Drug | RxNorm Extension |
| 2052774 | tenofovir disoproxil 250 MG Oral Tablet [HEPARODIN] by Ahngook New Pharm | Marketed Product | Drug | RxNorm Extension |
| 43039284 | 2 ML enfuvirtide 90 MG/ML Injectable Solution | Quant Clinical Drug | Drug | RxNorm Extension |
| 41166553 | Lamivudine 150 MG Oral Tablet [Lamivudin Hexal] Box of 60 by Novartis | Marketed Product | Drug | RxNorm Extension |
| 37593309 | Atazanavir Oral Granules | Clinical Drug Form | Drug | RxNorm Extension |
| 41011686 | darunavir 800 MG Oral Tablet [Prezista] Box of 30 by Haematogmbh | Marketed Product | Drug | RxNorm Extension |
| 40746935 | bictegravir 50 MG / emtricitabine 200 MG / tenofovir alafenamide 25 MG Oral Tablet [Biktarvy] by Gilead | Marketed Product | Drug | RxNorm Extension |
| 36811149 | emtricitabine 200 MG / tenofovir disoproxil 250 MG Oral Tablet [Ictastan] by Actavis | Marketed Product | Drug | RxNorm Extension |
| 43298296 | {112 (dasabuvir 250 MG Oral Tablet) / 112 (ombitasvir 12.5 MG / paritaprevir 75 MG / Ritonavir 50 MG Oral Tablet) / 56 (Ribavirin 600 MG Oral Tablet) } Pack [Viekira Pak-Rbv] box of 1 by Abbvie | Marketed Product | Drug | RxNorm Extension |
| 1560084 | Symtuza Pill | Branded Dose Group | Drug | RxNorm |
| 40733284 | maraviroc 20 MG/ML Oral Solution [Celsentri] | Branded Drug | Drug | RxNorm Extension |
| 40720631 | tenofovir disoproxil 250 MG Oral Tablet Box of 30 by Lupin | Marketed Product | Drug | RxNorm Extension |
| 44174953 | Lamivudine 300 MG Oral Tablet [Lamivudin Teva] Box of 60 by Ratiopharm | Marketed Product | Drug | RxNorm Extension |
| 41435634 | 225 ML fosamprenavir 0.222 MG/ML Oral Solution [Telzir] Box of 1 | Quant Branded Box | Drug | RxNorm Extension |
| 43022455 | darunavir 800 MG Delayed Release Oral Tablet Box of 30 by TEVA | Marketed Product | Drug | RxNorm Extension |
| 21112113 | Nevirapine 200 MG Oral Tablet Box of 60 by Wockhardt | Marketed Product | Drug | RxNorm Extension |
| 2053052 | lamivudine 100 MG Oral Tablet [LAMIDINE] | Branded Drug | Drug | RxNorm Extension |
| 43804528 | efavirenz Oral Tablet [Efavirenz Hexal] | Branded Drug Form | Drug | RxNorm Extension |
| 40853348 | Nevirapine 400 MG Oral Tablet [Viramune] Box of 90 by Cc | Marketed Product | Drug | RxNorm Extension |
| 41435789 | 240 ML Lamivudine 10 MG/ML Oral Solution [Epivir] Box of 1 by Emra-Med | Marketed Product | Drug | RxNorm Extension |
| 36406645 | raltegravir 25 MG Oral Tablet Box of 60 | Clinical Drug Box | Drug | RxNorm Extension |
| 44101877 | Lamivudine 300 MG Oral Tablet [Auro-Lamivudine] by Auro | Marketed Product | Drug | RxNorm Extension |
| 43171802 | ombitasvir 12.5 MG / paritaprevir 75 MG / Ritonavir 50 MG Delayed Release Oral Tablet Box of 56 | Clinical Drug Box | Drug | RxNorm Extension |
| 1724862 | zalcitabine 0.375 MG | Clinical Drug Comp | Drug | RxNorm |
| 40720768 | raltegravir 100 MG Oral Granules | Clinical Drug | Drug | RxNorm Extension |
| 40825040 | Lamivudine 150 MG / Zidovudine 300 MG Oral Tablet [Combivir] Box of 60 by Adjupharm | Marketed Product | Drug | RxNorm Extension |
| 35756373 | Lamivudine 150 MG / Zidovudine 300 MG Delayed Release Oral Tablet by A A H | Marketed Product | Drug | RxNorm Extension |
[truncated: 457,117 more chars]
