# Supplementary material for: Temporal Events Detector for Pregnancy Care (TED-PC): A rule-based algorithm to infer gestational age and delivery date from electronic health records of pregnant women with and without COVID-19
Source: PLoS One. 2022 Oct 31;17(10):e0276923. doi: 10.1371/journal.pone.0276923 (PMC9621451; doi:10.1371/journal.pone.0276923)
Supplement: S5 Table — (DOCX) [file pone.0276923.s005.docx]

**Supporting information 5**

**Table. Selected demographics and underlying conditions for pregnant women with and without COVID-19 by gestations during pre- and peri-pandemic.**

|  | **Pre-Pandemic^*^** | | |  | **Peri-Pandemic^†^** | | | | | | | | | | | | | | | | | | |  |  |
| --- | --- | --- | --- | --- | --- | --- | --- | --- | --- | --- | --- | --- | --- | --- | --- | --- | --- | --- | --- | --- | --- | --- | --- | --- | --- |
|  | **Total** | | |  | **Total** | | **COVID-19 Infection  Before the DOD** | | | | | **COVID-19 Infection During the First or Second Trimester  (before 28 weeks)** | | | | | **COVID-19 Infection During the Third Trimester  (28 week and after)^‡^** | | | | | | |  |  |
| **Characteristics** | | n=104,791 | |  | n=191,403 | | No (n=174,744) | | Yes (n=16,659) | | No (n=172,237) | | | | Yes (n=4,252) | | No (n=171,442) | | Yes (n=12,697) | | | | |  |  |
| **Age group** |  | |  |  |  | |  | |  | |  | |  |  | |  |  |  |  |  | | | |  |  |
| **15-19** | 3,296 | | 3.1% |  | 5,366 | 2.8% | 4,778 | 2.7% | 588 | 3.5% | 4,773 | | 2.8% | - | | - | 4,695 | 2.7% | 499 | 3.9% | | | |  |  |
| **20-24** | 17,076 | | 16.3% |  | 28,000 | 14.6% | 25,051 | 14.3% | 2,949 | 17.7% | 24,978 | | 14.5% | 679 | | 16.2% | 24,561 | 14.3% | 2,305 | 18.2% | | | |  |  |
| **25-29** | 27,337 | | 26.1% |  | 46,867 | 24.5% | 42,406 | 24.3% | 4,461 | 26.8% | 42,285 | | 24.6% | 1,148 | | 26.8% | 41,616 | 24.3% | 3,400 | 26.8% | | | |  |  |
| **30-34** | 33,590 | | 32.1% |  | 60,815 | 31.8% | 56,057 | 32.1% | 4,758 | 28.6% | 55,916 | | 32.5% | 1,278 | | 29.8% | 55,120 | 32.2% | 3,571 | 28.1% | | | |  |  |
| **35-39** | 18,843 | | 18.0% |  | 39,162 | 20.5% | 36,173 | 20.7% | 2,989 | 17.9% | 36,044 | | 20.9% | 797 | | 18.9% | 35,412 | 20.7% | 2,236 | 17.6% | | | |  |  |
| **40-44** | 4,326 | | 4.1% |  | 10,402 | 5.4% | 9,553 | 5.5% | 849 | 5.1% | 9,519 | | 5.5% | 233 | | 5.5% | 9,332 | 5.4% | 634 | 5.0% | | | |  |  |
| **45-49** | 323 | | 0.3% |  | 791 | 0.4% | 726 | 0.4% | 65 | 0.4% | 722 | | 0.4% | - | | - | 706 | 0.4% | 52 | 0.4% | | | |  |  |
| **Race** | | | | | | | | | | |  | |  |  | |  |  |  |  |  | | | |  |  |
| **White** | 59,842 | | 57.1% |  | 95,517 | 49.9% | 88,898 | 50.9% | 6,619 | 39.7% | 88,680 | | 51.5% | 1,897 | | 44.6% | 87,550 | 51.1% | 4,784 | 37.7% | | | |  |  |
| **Black** | 18,536 | | 17.7% |  | 33,144 | 17.3% | 30,567 | 17.5% | 2,577 | 15.5% | 30,437 | | 17.7% | 669 | | 15.7% | 29,631 | 17.3% | 1,937 | 15.3% | | | |  |  |
| **Hispanic/ Latino** | 15,961 | | 15.2% |  | 37,095 | 19.4% | 31,841 | 18.2% | 5,254 | 31.5% | 31,753 | | 18.4% | 1,160 | | 27.3% | 31,247 | 18.2% | 4,261 | 33.6% | | | |  |  |
| **Asian** | 4,219 | | 4.0% |  | 9,952 | 5.2% | 9,289 | 5.3% | 663 | 4.0% | 9,262 | | 5.4% | 178 | | 4.2% | 9,151 | 5.3% | 496 | 3.9% | | | |  |  |
| **NHOPI** | 182 | | 0.2% |  | 375 | 0.2% | 325 | 0.2% | 50 | 0.3% | 322 | | 0.2% | - | | - | 312 | 0.2% | 35 | 0.3% | | | |  |  |
| **Other/ unknown** | 5,477 | | 5.2% |  | 13,996 | 7.3% | 12,643 | 7.2% | 1,353 | 8.1% | 12,611 | | 7.3% | 300 | | 7.1% | 12,397 | 7.2% | 1,071 | 8.4% | | | |  |  |
| **Multiracial** | 574 | | 0.5% |  | 1,324 | 0.7% | 1,181 | 0.7% | 143 | 0.9% | 1,172 | | 0.7% | - | | - | 1,154 | 0.7% | 113 | 0.9% | | | |  |  |
| **Obesity/overweight** | | | | | | | | | | |  | |  |  | |  |  |  |  |  | | | |  |  |
| **No** | 77,992 | | 74.4% |  | 133,988 | 70.0% | 123,181 | 70.5% | 10,807 | 64.9% | 122,822 | | 70.5% | 2,702 | | 63.5% | 120,957 | 70.6% | 8,179 | 64.4% | | | |  |  |
| **Yes** | 26,799 | | 25.6% |  | 57,415 | 30.0% | 51,563 | 29.5% | 5,852 | 35.1% | 51,415 | | 29.5% | 1,550 | | 36.5% | 50,485 | 29.4% | 4,518 | 35.6% | | | |  |  |
| **Hypertensive disorders (any)^§^** | | | | | | | | | | |  | |  |  | |  |  |  |  |  | | | |  |  |
| **No** | 85,308 | | 81.4% |  | 150,308 | 78.5% | 137,226 | 78.5% | 13,082 | 78.5% | 136,810 | | 78.5% | 3,289 | | 77.4% | 134,710 | 78.6% | 9,992 | 78.7% | | | |  |  |
| **Yes** | 19,483 | | 18.6% |  | 41,095 | 21.5% | 37,518 | 21.5% | 3,577 | 21.5% | 37,427 | | 21.5% | 963 | | 22.6% | 36,732 | 21.4% | 2,705 | 21.3% | | | |  |  |
| **Diabetes (any)^‖^** | | | | | | | | | | |  | |  |  | |  |  |  |  |  | | | |  |  |
| **No** | 94,375 | | 90.1% |  | 167,225 | 87.4% | 152,860 | 87.5% | 14,365 | 86.2% | 152,396 | | 87.5% | 3,641 | | 85.6% | 149,893 | 87.4% | 10,936 | 86.1% | | | |  |  |
| **Yes** | 10,416 | | 9.9% |  | 24,178 | 12.6% | 21,884 | 12.5% | 2,294 | 13.8% | 21,841 | | 12.5% | 611 | | 14.4% | 21,549 | 12.6% | 1,761 | 13.9% | | | |  |  |
| **COPD** | | | | | | | | | | |  | |  |  | |  |  |  |  |  | | | |  |  |
| **No** | 104,683 | | 99.9% |  | 191,120 | 99.9% | 174,499 | 99.9% | 16,621 | 99.8% | 173,995 | | 99.9% | - | | 99.8% | 171,204 | 99.9% | 12,666 | 99.8% | | | |  |  |
| **Yes** | 108 | | 0.1% |  | 283 | 0.1% | 245 | 0.1% | 38 | 0.2% | 242 | | 0.1% | - | | 0.2% | 238 | 0.1% | 31 | 0.2% | | | |  |  |
| **ARDS/ARF** | | | | | | | | | | |  | |  |  | |  |  |  |  |  | | | |  |  |
| **No** | 104,634 | | 99.9% |  | 190,669 | 99.6% | 174,316 | 99.8% | 16,353 | 98.2% | 173,812 | | 99.8% | 4,173 | | 98.1% | 171,043 | 99.8% | 12,448 | 98.0% | | | |  |  |
| **Yes** | 157 | | 0.1% |  | 734 | 0.4% | 428 | 0.2% | 306 | 1.8% | 425 | | 0.2% | 79 | | 1.9% | 399 | 0.2% | 249 | 2.0% | | | |  |  |
| **Myocardial Infarction** | | | | | | | | | | |  | |  |  | |  |  |  |  |  | | | |  |  |
| **No** | 104,662 | | 99.9% |  | 191,132 | 99.9% | 174,494 | 99.9% | 16,638 | 99.9% | 173,989 | | 99.9% | - | | 99.9% | 171,204 | 99.9% | - | 99.9% | | | |  |  |
| **Yes** | 129 | | 0.1% |  | 271 | 0.1% | 250 | 0.1% | 21 | 0.1% | 248 | | 0.1% |  | | 0.1% | 238 | 0.1% | - | 0.1% | | | |  |  |
| **Congestive Heart Failure** | | | | | | | | | | |  | |  |  | |  |  |  |  |  | | | |  |  |
| **No** | 104,410 | | 99.6% |  | 190,673 | 99.6% | 174,081 | 99.6% | 16,592 | 99.6% | 173,579 | | 99.6% | - | | 99.6% | 170,803 | 99.6% | 12,646 | 99.6% | | | |  |  |
| **Yes** | 381 | | 0.4% |  | 730 | 0.4% | 663 | 0.4% | 67 | 0.4% | 658 | | 0.4% | - | | 0.4% | 639 | 0.4% | 51 | 0.4% | | | |  |  |
| **HIV/AIDS** | | | | | | | | | | |  | |  |  | |  |  |  |  |  | | | |  |  |
| **No** | 104,406 | | 99.6% |  | 190,583 | 99.6% | 174,021 | 99.6% | 16,562 | 99.4% | 173,519 | | 99.6% | 4,229 | | 99.5% | 170,737 | 99.6% | 12,620 | 99.4% | | | |  |  |
| **Yes** | 385 | | 0.4% |  | 820 | 0.4% | 723 | 0.4% | 97 | 0.6% | 718 | | 0.4% | 23 | | 0.5% | 705 | 0.4% | 77 | 0.6% | | | |  |  |
| **Placental Abruption** | | | | | | | | | | |  | |  |  | |  |  |  |  |  | | | |  |  |
| **No** | 103,872 | | 99.1% |  | 189,319 | 98.9% | 172,832 | 98.9% | 16,487 | 99.0% | 172,338 | | 98.9% | 4,206 | | 98.9% | 169,707 | 99.0% | 12,572 | 99.0% | | | |  |  |
| **Yes** | 919 | | 0.9% |  | 2,084 | 1.1% | 1,912 | 1.1% | 172 | 1.0% | 1,899 | | 1.1% | 46 | | 1.1% | 1,735 | 1.0% | 125 | 1.0% | | | |  |  |
|  |  | |  |  |  |  |  |  |  |  |  | |  |  | |  |  |  |  |  | | | |  |  |
| ^*^: before the COVID-19 pandemic: June 1^st^, 2018 to February 29^th^, 2020 | | | | | | | | | | | | | | | | | | |  |  | | | |  |  |
| ^†^: during the COVID-19 pandemic: May 1^st^, 2020 to May 31^st^, 2021 | | | | | | | | | | | | | | | | | | |  |  | | | |  |  |
| ^‡^: among those pregnancies with gestational length greater than 27 weeks | | | | | | | | | | | | | | | | | | | |  | | | |  |  |
| ^§^: includes chronic hypertension, gestational hypertension, preeclampsia, eclampsia, and HELLP syndrome | | | | | | | | | | | | | | | | | | |  |  | | | |  |  |
| ^‖^: includes prepregnancy diabetes and gestational diabetes | | | | | | | | | | | | | | | | | |  |  |  | | | |  |  |
| DOD: date of delivery | | | | | | | | | | | | | | | |  |  |  |  |  | | | |  |  |
| NHOPI: Native Hawaiians and other Pacific Islanders | | | | | | | | | | | | | | | | | |  |  |  | | | |  |  |
| COPD: chronic obstructive pulmonary disease | | | | | | | | | | | | | | | | | | | |  | | | |  |  |
| ARDS/ARF: acute respiratory distress syndrome or acute respiratory failure | | | | | | | | | | | | | | | | | |  |  |  | | | |  |  |
| -: cells with patient counts <20 or that have been jittered to obscure back calculation of the <20 cell | | | | | | | | | | | | | |  | |  | | | | |  |  |  | |  |
